# Global, regional, and national burden of epilepsy, 1990–2021: a systematic analysis for the Global Burden of Disease Study 2021

**DOI:** 10.1016/S2468-2667(24)00302-5

**Published:** 2025-02-24

**Authors:** GBD Epilepsy Collaborators, Valery L Feigin, Theo Vos, Balakrishnan Sukumaran Nair, Simon I Hay, Yohannes Habtegiorgis Abate, Abdallah H A Abd Al Magied, Samar Abd ElHafeez, Atef Abdelkader, Mohammad-Amin Abdollahifar, Auwal Abdullahi, Richard Gyan Aboagye, Lucas Guimarães Abreu, Samir Abu Rumeileh, Hasan Abualruz, Salahdein Aburuz, Ahmed Abu-Zaid, Isaac Yeboah Addo, Rufus Adesoji Adedoyin, Abiola Victor Adepoju, Muhammad Sohail Afzal, Saira Afzal, Aqeel Ahmad, Sajjad Ahmad, Tauseef Ahmad, Ali Ahmadi, Amir Mahmoud Ahmadzade, Ayman Ahmed, Haroon Ahmed, Mehrunnisha Sharif Ahmed, Muktar Beshir Ahmed, Salah Al Awaidy, Omar Al Omari, Yazan Al-Ajlouni, Mohammed Albashtawy, Bassam Al-Fatly, Abdelazeem M Algammal, Abid Ali, Mohammed Usman Ali, Syed Shujait Ali, Waad Ali, Sheikh Mohammad Alif, Joseph Uy Almazan, Najim Z Alshahrani, Awais Altaf, Mohammad Al-Wardat, Yaser Mohammed Al-Worafi, Hany Aly, Karem H Alzoubi, Sohrab Amiri, Robert Ancuceanu, Dhanalakshmi Angappan, Mohammed Tahir Ansari, Saeid Anvari, Anayochukwu Edward Anyasodor, Jalal Arabloo, Mosab Arafat, Aleksandr Y Aravkin, Brhane Berhe Aregawi, Abdulfatai Aremu, Maha Moh'd Wahbi Atout, Alok Atreya, Avinash Aujayeb, Setognal Birara Aychiluhm, Shahkaar Aziz, Ahmed Y Azzam, Ashish D Badiye, Ruhai Bai, Atif Amin Baig, Shankar M Bakkannavar, Soham Bandyopadhyay, Indrajit Banerjee, Mainak Bardhan, Suzanne Lyn Barker-Collo, Amadou Barrow, Zarrin Basharat, Azadeh Bashiri, Afisu Basiru, Mohammad-Mahdi Bastan, Sai Batchu, Babak Behnam, Diana Fernanda Bejarano Ramirez, Maryam Bemanalizadeh, Kebede A Beyene, Devidas S Bhagat, Akshaya Srikanth Bhagavathula, Sonu Bhaskar, Ajay Nagesh Bhat, Gurjit Kaur Bhatti, Jasvinder Singh Bhatti, Mohiuddin Ahmed Bhuiyan, Soumitra S Bhuyan, Cem Bilgin, Francesca Bisulli, Archith Boloor, Sri Harsha Boppana, Souad Bouaoud, Yasser Bustanji, Mehtap Çakmak Barsbay, Felix Carvalho, Joao Mauricio Castaldelli-Maia, Rama Mohan Chandika, Vijay Kumar Chattu, Anis Ahmad Chaudhary, Patrick R Ching, Hitesh Chopra, Dinh-Toi Chu, Hongyuan Chu, Samuele Cortese, Paolo Angelo Cortesi, Natalia Cruz-Martins, Omid Dadras, Xiaochen Dai, Emanuele D'Amico, Lalit Dandona, Rakhi Dandona, Samuel Demissie Darcho, Amira Hamed Darwish, Amol S Dhane, Vishal R Dhulipala, Michael J Diaz, Thanh Chi Do, Sushil Dohare, Ojas Prakashbhai Doshi, Haneil Larson Dsouza, Arkadiusz Marian Dziedzic, Alireza Ebrahimi, Negin Eissazade, Michael Ekholuenetale, Rabie Adel El Arab, Ibrahim Farahat El Bayoumy, Omar Abdelsadek Abdou El Meligy, Hala Rashad Elhabashy, Muhammed Elhadi, Chadi Eltaha, Adeniyi Francis Fagbamigbe, Ayesha Fahim, Jawad Fares, Mohsen Farjoud Kouhanjani, Abidemi Omolara Fasanmi, Ali Fatehizadeh, Patrick Fazeli, Timur Fazylov, Ginenus Fekadu, Seyed-Mohammad Fereshtehnejad, Pietro Ferrara, Nuno Ferreira, Getahun Fetensa, Florian Fischer, Matteo Foschi, Muktar A Gadanya, Yaseen Galali, Balasankar Ganesan, Xiang Gao, Ravindra Kumar Garg, Miglas Welay Gebregergis, Fataneh Ghadirian, Seyyed-Hadi Ghamari, Jaleed Ahmed Gilani, Alem Abera Girmay, Giorgia Giussani, Elena V Gnedovskaya, Mahaveer Golechha, Mahdi Gouravani, Ayman Grada, Shi-Yang Guan, Sapna Gupta, Mohammad Haghani Dogahe, Arvin Haj-Mirzaian, Nadia M Hamdy, Netanja I Harlianto, Ahmed I Hasaballah, Hamidreza Hasani, Amr Hassan, Ikrama Ibrahim Hassan, Mahgol Sadat Hassan Zadeh Tabatabaei, Omar E Hegazi, Golnaz Heidari, Mehdi Hemmati, Kamal Hezam, Nguyen Quoc Hoan, Ramesh Holla, Mehdi Hosseinzadeh, Junjie Huang, Hong-Han Huynh, Bing-Fang Hwang, Segun Emmanuel Ibitoye, Adalia Ikiroma, Olayinka Stephen Ilesanmi, Irena M Ilic, Milena D Ilic, Mohammad Tarique Imam, Mustapha Immurana, Arit Inok, Md Rabiul Islam, Chidozie Declan Iwu, Louis Jacob, Abdollah Jafarzadeh, Haitham Jahrami, Ammar Abdulrahman Jairoun, Mihajlo Jakovljevic, Reza Jalilzadeh Yengejeh, Roland Dominic G Jamora, Talha Jawaid, Sathish Kumar Jayapal, Zixiang Ji, Jost B Jonas, Nitin Joseph, Charity Ehimwenma Joshua, Zubair Kabir, Rizwan Kalani, Arun Kamireddy, Kehinde Kazeem Kanmodi, Neeti Kapoor, Faizan Zaffar Kashoo, Harkiran Kaur, Foad Kazemi, Himanshu Khajuria, Alireza Khalilian, Maseer Khan, Haitham Khatatbeh, Hamid Reza Khayat Kashani, Khalid A Kheirallah, Feriha Fatima Khidri, Moein Khormali, Atulya Aman Khosla, Jagdish Khubchandani, Yun Jin Kim, Yun Seo Kim, Ruth W Kimokoti, Hyun Yong Koh, Ali-Asghar Kolahi, Karel Kostev, Kewal Krishan, Vijay Krishnamoorthy, Jera Kruja, Mohammed Kuddus, Mukhtar Kulimbet, G Anil Kumar, Manasi Kumar, Satyajit Kundu, Ville Kytö, Chandrakant Lahariya, Dharmesh Kumar Lal, Judit Lám, Iván Landires, Francesco Lanfranchi, Nhi Huu Hanh Le, Seung Won Lee, Virendra S Ligade, Stephen S Lim, Christine Linehan, Xiaofeng Liu, Xuefeng Liu, José Francisco López-Gil, Giancarlo Lucchetti, Azeem Majeed, Kashish Malhotra, Ahmad Azam Malik, Vahid Mansouri, Hamid Reza Marateb, Miquel Martorell, Roy Rillera Marzo, Yasith Mathangasinghe, Rishi P Mediratta, Man Mohan Mehndiratta, Hadush Negash Meles, Endalkachew Belayneh Melese, George A Mensah, Atte Meretoja, Tomislav Mestrovic, Sachith Mettananda, Giuseppe Minervini, Reza Mirfakhraie, Moonis Mirza, Awoke Misganaw, Arup Kumar Misra, Abdalla Z Mohamed, Nouh Saad Mohamed, Abdollah Mohammadian-Hafshejani, Ibrahim Mohammadzadeh, Syam Mohan, Ali H Mokdad, Lorenzo Monasta, AmirAli Moodi Ghalibaf, Maryam Moradi, Rohith Motappa, Lorenzo Muccioli, Francesk Mulita, Yanjinlkham Munkhsaikhan, Efren Murillo-Zamora, Sathish Muthu, Amin Nabavi, Ganesh R Naik, Shumaila Nargus, Abdulqadir J Nashwan, Zuhair S Natto, Javaid Nauman, Muhammad Naveed, Biswa Prakash Nayak, Athare Nazri-Panjaki, Gaurav Nepal, Henok Biresaw Netsere, Hau Thi Hien Nguyen, Robina Khan Niazi, Ali Nikoobar, Majid Nozari, Chisom Adaobi Nri-Ezedi, Vincent Ebuka Nwatah, Ogochukwu Janet Nzoputam, Bogdan Oancea, Andrew T Olagunju, Oladotun Victor Olalusi, Ahmed Omar Bali, Michal Ordak, Verner N Orish, Esteban Ortiz-Prado, Nikita Otstavnov, Amel Ouyahia, Mayowa O Owolabi, Alicia Padron-Monedero, Jagadish Rao Padubidri, Sujogya Kumar Panda, Songhomitra Panda-Jonas, Deepshikha Pande Katare, Anamika Pandey, Leonidas D Panos, Ioannis Pantazopoulos, Paraskevi Papadopoulou, Utsav Parekh, Romil R Parikh, Nicholas Parsons, Roberto Passera, Shankargouda Patil, Shrikant Pawar, Hamidreza Pazoki Toroudi, Umberto Pensato, Prince Peprah, Mario F P Peres, Simone Perna, Hoang Nhat Pham, Zahra Zahid Piracha, Michael A Piradov, Dimitri Poddighe, Ramesh Poluru, Ahmad Pour-Rashidi, Jalandhar Pradhan, Manya Prasad, Dimas Ria Angga Pribadi, Jagadeesh Puvvula, Nameer Hashim Qasim, Venkatraman Radhakrishnan, Pankaja Raghav, Fakher Rahim, Mosiur Rahman, Amir Masoud Rahmani, Mohammad Rahmanian, Adarsh Raja, Ali Rajabpour Sanati, Pushp Lata Rajpoot, Mahmoud Mohammed Ramadan, Shakthi Kumaran Ramasamy, Nemanja Rancic, Sowmya J Rao, Mohammad-Mahdi Rashidi, Devarajan Rathish, Salman Rawaf, Murali Mohan Rama Krishna Reddy, Elrashdy M Moustafa Mohamed Redwan, Mohsen Rezaeian, Taeho Gregory Rhee, Muhammad Riaz, Jefferson Antonio Buendia Rodriguez, Leonardo Roever, Marina Romozzi, Moustaq Karim Khan Rony, Kevin T Root, Himanshu Sekhar Rout, Aly M A Saad, Cameron John Sabet, Basema Ahmad Saddik, Reihaneh Sadeghian, Mohammad Reza Saeb, Umar Saeed, Usman Saeed, Fatemeh Saheb Sharif-Askari, Narjes Saheb Sharif-Askari, Amirhossein Sahebkar, Zahra Saif, S Mohammad Sajadi, Afeez Abolarinwa Salami, Sohrab Salimi, Yoseph Leonardo Samodra, Abdallah M Samy, Gargi Sachin Sarode, Sachin C Sarode, Brijesh Sathian, Anudeep Sathyanarayan, Maheswar Satpathy, Monika Sawhney, Siddharthan Selvaraj, Mohammad H Semreen, Ashenafi Kibret Sendekie, Subramanian Senthilkumaran, Yashendra Sethi, Allen Seylani, Ataollah Shahbandi, Samiah Shahid, Masood Ali Shaikh, Summaiya Zareen Shaikh, Muhammad Aaqib Shamim, Mehran Shams-Beyranvand, Alfiya Shamsutdinova, Amin Sharifan, Javad Sharifi Rad, Anupam Sharma, Vishal Sharma, Maryam Shayan, Zubeda Begum Sheikh, Mahabalesh Shetty, Pavanchand H Shetty, Premalatha K Shetty, Aminu Shittu, Nathan A Shlobin, Seyed Afshin Shorofi, Sunil Shrestha, Emmanuel Edwar Siddig, Gagandeep Singh, Harmanjit Singh, Jasvinder A Singh, Paramdeep Singh, Puneetpal Singh, Surjit Singh, Shipra Solanki, Soroush Soraneh, Muhammad Haroon Stanikzai, Mark J M Sullman, Katharina S Sunnerhagen, Vinay Suresh, Chandan Kumar Swain, Lukasz Szarpak, Payam Tabaee Damavandi, Rafael Tabarés-Seisdedos, Celine Tabche, Jabeen Taiba, Manoj Tanwar, Minale Tareke, Mohamad-Hani Temsah, Reem Mohamad Hani Temsah, Masayuki Teramoto, Pugazhenthan Thangaraju, Sathish Thirunavukkarasu, Jansje Henny Vera Ticoalu, Tenaw Yimer Tiruye, Krishna Tiwari, Vikas Kumar Tiwari, Marcos Roberto Tovani-Palone, Thang Huu Tran, Nguyen Tran Minh Duc, Manjari Tripathi, Samuel Joseph Tromans, Daniel Hsiang-Te Tsai, Aristidis Tsatsakis, Evangelia Eirini Tsermpini, Munkhtuya Tumurkhuu, Aniefiok John Udoakang, Saeed Ullah, Muhammad Umair, Bhaskaran Unnikrishnan, Daniele Urso, Jibrin Sammani Usman, Asokan Govindaraj Vaithinathan, Alireza Vakilian, Ravi Prasad Varma, Narayanaswamy Venketasubramanian, Jorge Hugo Villafañe, Manish Vinayak, Andres Fernando Vinueza Veloz, Mandaras Tariku Walde, Shu Wang, Yanzhong Wang, Abdul Waris, Nuwan Darshana Wickramasinghe, Andrea Sylvia Winkler, Subah Abderehim Yesuf, Arzu Yiğit, Vahit Yiğit, Mekdes Tigistu Yilma, Yazachew Engida Yismaw, Dong Keon Yon, Naohiro Yonemoto, Chuanhua Yu, Milad Zandi, Aurora Zanghì, Mohammed G M Zeariya, Zhongyi Zhao, Claire Chenwen Zhong, Magdalena Zielińska, Osama A Zitoun, Sa'ed H Zyoud, Samer H Zyoud, Ilari Rautalin, Charles Richard James Newton, Samuel Wiebe, Christopher J L Murray

## Abstract

**Background:**

Epilepsy is one of the most common serious neurological disorders and affects individuals of all ages across the globe. The aim of this study is to provide estimates of the epilepsy burden on the global, regional, and national levels for 1990–2021.

**Methods:**

Using well established Global Burden of Diseases, Injuries, and Risk Factors Study (GBD) methodology, we quantified the prevalence of active idiopathic (epilepsy of genetic or unknown origin) and secondary epilepsy (epilepsy due to an underlying abnormality of the brain structure or chemistry), as well as incidence, death, and disability-adjusted life-years (DALYs) by age, sex, and location (globally, 21 GBD regions and seven super-regions, World Bank country income levels, Socio-demographic Index [SDI], and 204 countries) and their trends from 1990 to 2021. Vital registrations and verbal autopsies provided information about deaths, and data on the prevalence and severity of epilepsy, largely came from population representative surveys. All estimates were calculated with 95% uncertainty intervals (UIs).

**Findings:**

In 2021, there were 51·7 million (95% UI 44·9–58·9) people with epilepsy (idiopathic and secondary combined) globally, with an age-standardised prevalence of 658 per 100 000 (569–748). Idiopathic epilepsy had an age-standardised prevalence of 307 per 100 000 (235–389) globally, with 24·2 million (18·5–30·7) prevalent cases, and secondary epilepsy had a global age-standardised prevalence of 350 per 100 000 (322–380). In 2021, 0·7% of the population had active epilepsy (0·3% attributed to idiopathic epilepsy and 0·4% to secondary epilepsy), and the age-standardised global prevalence of epilepsy from idiopathic and secondary epilepsy combined increased from 1990 to 2021 by 10·8% (1·1–21·3), mainly due to corresponding changes in secondary epilepsy. However, age-standardised death and DALY rates of idiopathic epilepsy reduced from 1990 to 2021 (decline of 15·8% [8·8–22·8] and 14·5% [4·2–24·2], respectively). There were three-fold to four-fold geographical differences in the burden of active idiopathic epilepsy, with the bulk of the burden residing in low-income to middle-income countries: 82·1% (81·1–83·4) of incident, 80·4% prevalent (79·7–82·7), 84·7% (83·7–85·1) fatal epilepsy, and 87·9% (86·2–89·2) epilepsy DALYs.

**Interpretation:**

Although the global trends in idiopathic epilepsy deaths and DALY rates have improved in the preceding decades, in 2021 there were almost 52 million people with active epilepsy (24 million from idiopathic epilepsy and 28 million from secondary epilepsy), with the bulk of the burden (>80%) residing in low-income to middle-income countries. Better treatment and prevention of epilepsy are required, along with further research on risk factors of idiopathic epilepsy, good-quality long-term epilepsy surveillance studies, and exploration of the possible effect of stigma and cultural differences in seeking medical attention for epilepsy.

**Funding:**

Bill and Melinda Gates Foundation

## Introduction

Epilepsy is one of the most common serious brain conditions of increasing burden that affects individuals of all ages across the globe,^1^, ^2^ increases risk of premature death up to three times compared with the general population, and is characterised by recurrent, unprovoked seizures due to abnormal excessive or synchronous neuronal activity in the brain.^3^ The disease imposes a substantial economic, psychosocial, physical, and mental burden for health systems, societies, and affected individuals and their families.^4^, ^5^, ^6^

In 2022, epilepsy was identified by the 75th World Health Assembly and WHO as one of the top priorities in prevention and control of non-communicable diseases, and a special intersectoral global action plan on epilepsy and other neurological disorders for 2022–31 was adopted.^6^ To enable evidence-based actions and awareness campaigns, and to strengthen public and private efforts to improve quality of and access to care and reduce the effect of the disease, accurate and regularly updated data on epilepsy incidence, prevalence, death, and disability by age, sex, and location are of crucial importance.^2^ From public health perspectives, it is important to provide burden estimates for idiopathic (genetic) epilepsy separately from and combined with secondary epilepsy (epilepsy syndrome due to an underlying abnormality of the brain structure or chemistry)^7^ for identifying prognosis and opportunities for prevention efforts, which are clearly different between the two types of epileptic seizures. As in the Global Burden of Diseases, Injuries, and Risk Factors Study (GBD) report on epilepsy published in 2019,^8^ causes of secondary epilepsy included, among others, stroke, neurodegenerative disorders, infections and inflammatory disorders, brain tumours, traumatic brain injuries, and congenital abnormalities. In this GBD study, these conditions were not considered risks, but rather quantified as sequelae of the underlying causes of secondary epilepsy. Therefore, the causes of secondary epilepsy are more amendable to prevention, but their treatment usually is less successful because they do not address the often severe comorbid disabilities from motor or intellectual impairments. This information might also be used for projections of the burden of epilepsy, which is also important for health-care planning and resource allocation.^9^ As emphasised by WHO, an understanding of the development of epilepsy after a brain insult or parasitic infection is crucial to the development of secondary prevention strategies.^2^

**Research in context**

**Evidence before this study**

Global Burden of Diseases, Injuries, and Risk Factors Study (GBD) 2016 showed that despite a substantial decrease in age-standardised rates of idiopathic epilepsy mortality (24·5% [95% UI 10·8–31·8]) and disability-adjusted life-years (DALYs; 19·4% [9·0–27·7]) from 1990 to 2016, there was a small, although non-substantial, increase in the age-standardised prevalence (6% [–4·0 to 16·7]). The number of survivors with idiopathic epilepsy who remained disabled (as measured by DALYs) had increased: 15·3 million (11·5–19·6) and 11·3 million (8·6–14·1) in 1990 to 25·1 million (19·0–31·4) and 13·1 million (10·0–16·7) in 2016, respectively to prevalence and disability. We searched PubMed for papers from Jan 10, 2016, to Jan 28, 2022, without language restrictions, using the terms (2016/10/01(PDAT) : 3000(PDAT)) AND (“epilepsy”(MeSH Terms) OR “epilepsy, partial, motor”(MeSH Terms) OR “epilepsy, benign neonatal”(MeSH Terms) OR “epilepsy, reflex”(MeSH Terms) OR “myoclonic epilepsy, juvenile”(MeSH Terms) OR “epilepsy, frontal lobe”(MeSH Terms) OR “epilepsy, complex partial”(MeSH Terms) OR “epilepsy, post-traumatic”(MeSH Terms) OR “epilepsy, temporal lobe”(MeSH Terms) OR “epilepsy, absence”(MeSH Terms) OR “epilepsy, tonic-clonic”(MeSH Terms) OR “epilepsies, myoclonic”(MeSH Terms) OR “epilepsies, partial”(MeSH Terms) OR epilep*(Title/Abstract)) AND (inciden*(Title/Abstract) OR prevalen*(Title/Abstract)) NOT (animals(MeSH) NOT humans(MeSH)). Previous studies have often been hampered by incomplete data and lack of differentiation between idiopathic and secondary epilepsy. We aimed to overcome these limitations by integrating comprehensive data from population-representative surveys and vital registrations, and offer a more accurate and detailed picture of the global epilepsy burden.

**Added value of this study**

This systematic analysis of the GBD 1990–2021 data advances previous GBD estimates on the epilepsy burden and provides the most up-to-date prevalence estimates of not only active idiopathic epilepsy but also active secondary epilepsy on global, regional, and national (204 countries) levels by age and sex for the 1990–2021 period.

**Implications of all the available evidence**

These data are important for evidence-based implementation of the WHO Resolution WHA73·10 on integrated (multisectoral) response to epilepsy and other neurological disorders for global health policy and resource allocation. By identifying regions with the highest burden of epilepsy, this study provides crucial data for targeted interventions. Policy makers can use these insights to prioritise funding for epilepsy care, improve access to antiseizure medications, and implement training programmes for health-care providers in underserved regions. Additionally, the study underscores the need for ongoing surveillance and research to track progress and adapt strategies as needed. Urgent efforts must be made by all key stakeholders and decision makers to increase awareness and education about epilepsy, eliminate stigmatisation and discrimination associated with epilepsy, better control secondary causes of epilepsy (eg, stroke, CNS zoonotic diseases, and other infectious diseases), improve access to existing treatments in economically disadvantaged countries or populations, and foster workforce development, especially in low-income countries. Further research on risk factors of idiopathic epilepsy, good-quality long-term epilepsy surveillance studies, and exploration of the possible effect of stigma and cultural differences in seeking medical attention for epilepsy is required, in addition to developing new effective and affordable treatments.

Previous epilepsy burden reports of GBD^8^ and GBD-based papers^10^, ^11^, ^12^, ^13^ from the 1990–2016 period, and were largely limited to the burden of idiopathic epilepsy. This GBD 2021 study aims to quantify idiopathic and secondary epilepsy prevalence, as well as incidence, death, and disability-adjusted life-years (DALYs) by age, sex, and location (globally, 21 GBD regions and seven super-regions,^14^ World Bank country income levels,^15^ Socio-demographic Index [SDI],^16^ and 204 countries) and their trends from 1990 to 2021. This manuscript was produced as part of the GBD Collaborator Network and in accordance with the GBD Protocol.^17^

## Methods

### Mortality estimates

The GBD study systematically models 371 diseases and injuries at the global, regional, and national level (in select countries; also at the subnational level), with subnational analyses in selected countries.^18^, ^19^, ^20^

For assessment of mortality due to epilepsy, we used underlying cause-of-death data, making corrections for misclassifications and under-reporting.^18^ Data used to estimate epilepsy mortality included vital registration, verbal autopsy, and mortality surveillance data. The International Classification of Diseases (ICD) codes were used to reassign intermediary or unspecified causes to more specific categories. The codes for epilepsy for both ICD-9 (code 345) and ICD-10 (codes G40 and G41) were used. A Cause of Death Ensemble model^18^ was used, which combines multiple models to improve mortality estimation accuracy. This is a method produced specifically for cause of death analysis in the GBD study. Further details on the methodology have been published elsewhere^18^, ^21^ and are provided in appendix 1 (p 3–32).

### Non-fatal estimates

The guidelines for epidemiological studies on epilepsy, its classification, and definition from the International League Against Epilepsy^3^, ^22^ formed the basis for our reference definition. An epilepsy case was defined as someone with an active, recurring condition of epileptic seizures, at least two seizures, unprovoked by any immediate cause, and who has had at least one epileptic seizure in the past 5 years regardless of antiepileptic drug treatment.^3^ We used data from additional sources from Jan 10, 2016, to Jan 28, 2022. This latest systematic review included data from Jan 10, 2016, to Jan 28, 2022, because some relevant data for the 1990–2021 period might have been published after 2021. This review yielded 24 new sources on two measures (appendix 1 p 5). The studies included were population-based, representative surveys that reported prevalence, incidence, remission rate, excess mortality rate, relative risk of mortality, standardised mortality ratio, or with-condition mortality rate. Studies that had no clearly defined sample were excluded. Studies that recorded the lifetime recall of epilepsy were crosswalked (the process of adjusting data for known biases) to the reference definition for epilepsy. Using a GBD meta-regression–Bayesian, regularised, trimmed method^23^ on the log male:female ratio of prevalence, we split observations where sex was reported for males and females combined into observations for males and females separately. Data that covered an age period of more than 25 years were split into 5-year age bands using the age patterns discerned from DisMod-MR 2·1,^19^ a Bayesian meta-regression tool, built on a subset of the epilepsy data with age bands less than 25 years. DisMod-MR 2·1 was also used to model prevalence and incidence for idiopathic and secondary epilepsy combined.

### Idiopathic and secondary epilepsy

In GBD 2021, overall epilepsy was split into idiopathic epilepsy, in which the underlying cause is unknown or genetic in nature, and secondary epilepsy, in which the underlying cause is known (eg, epilepsy due to abnormality of the brain structure or chemistry). We make explicit estimates of secondary epilepsy due to neonatal, cerebral malaria, neonatal tetanus, meningitis, cystic echinococcosis, cysticercosis, and neonatal conditions. The majority of our epidemiological data sources use the International League Against Epilepsy 1985 proposal for classification of epilepsies and epileptic syndromes, definition for idiopathic (unknown cause but generally considered to be genetically determined) epilepsy,^24^, ^25^, ^26^, ^27^, ^28^ and therefore, for this review we also used this definition. Our systematic review covering Jan 10, 2016, to Jan 28, 2022, discovered no additional unique sources of the proportion of idiopathic epilepsy due to genetic or unknown causes beyond the 89 already identified, covering 18 of 21 world regions. Not all sources use MRI or CT scans as well as electroencephalograms to diagnose secondary epilepsy. Studies that did not use advanced diagnostic methods were readjusted to the study region where all possible diagnostic methods for secondary epilepsy were used using a binary covariate for study quality based on whether the study explicitly described use of neuroimaging diagnostics across all study participants. A mixed-effects model with random effects on super-region (the 21 world regions aggregated into seven groups defined in GBD) was built using these data. The prediction of the proportion of idiopathic epilepsy obtained from this model for each year and location combination was then used in conjunction with the incidence and prevalence results from the DisMod-MR 2·1 model to calculate incidence and prevalence for idiopathic and secondary epilepsy considered separately and combined. Consistent with previous GBD 2019 report on burden of epilepsy,^8^ secondary epilepsy was quantified as long-term consequences of meningitis, tetanus, malaria, cysticercosis, cystic echinococcosis, preterm birth complications, neonatal encephalopathy, neonatal sepsis, and neonatal haemolytic disease. Secondary epilepsy from other causes, such as brain cancer, traumatic brain injury, congenital anomalies, or stroke, was not quantified explicitly but assumed to be subsumed in the severity distributions and corresponding disability weights for those conditions.^29^

Disability-adjusted life-years (DALYs) are the sum of years of life lost and years lived with disability (YLDs). Uncertainty is propagated through each computation step by sampling 500 draws at each step. By ordering the draws, we were able to use the 2·5 and the 97·5 percentile values to form the uncertainty intervals. Differences between two draw sets are significant if the uncertainty level of the difference does not include zero. YLDs were calculated as prevalence multiplied by the category-specific disability weight. Further details on the methodology have been published elsewhere^19^ and are in appendix 1 (pp 3–32). This report adheres to the GATHER^30^ recommendations.

### Role of the funding source

The funder of the study had no role in study design, data collection, data analysis, data interpretation, or the writing of the report.

## Results

In 2021, there were 51·7 million (95% UI 44·9–58·9) people with epilepsy (idiopathic and secondary combined) globally (table 1), and the global age-standardised prevalence rate was 658 per 100 000 (569–748). Idiopathic epilepsy (appendix 1 pp 25–36) had an age-standardised prevalence rate of 307 per 100 000 (235–389) globally, with 24·2 million (18·5–30·7) prevalent cases (46·9% [41·1–52·1] of epilepsy from idiopathic and secondary epilepsy combined). Secondary epilepsy had a global age-standardised prevalence rate of 350 per 100 000 (322–380) and a combined number of 27·5 million (25·2–29·9) prevalent cases (53·1% [50·7–56·2] of epilepsy from idiopathic and secondary epilepsy combined).

**Table 1:** Prevalence of idiopathic epilepsy, secondary epilepsy, and idiopathic and secondary epilepsy combined, in 2021, and percentage change in age-standardised prevalence globally and by seven GBD super-regions, 21 GBD regions, and 204 individual countries or territories

		**Prevalence of idiopathic epilepsy**			**Prevalence of secondary epilepsy**			**Prevalence of idiopathic and secondary epilepsy combined**		
		2021 age-standardised prevalence per 100 000	2021 counts*	Percentage change in age-standardised rates, 1990–2021	2021 age-standardised prevalence per 100 000	2021 counts*	Percentage change in age-standardised rates, 1990–2021	2021 age-standardised prevalence per 100 000	2021 counts*	Percentage change in age-standardised rates, 1990–2021
**Global**		**307·4 (234·7 to 389·0)**	**24 221 000 (18 477 000 to 30 678 000)**	**6·9% (−9·7 to 25·5)**	**350·5 (322·3 to 380·5)**	**27 478 000 (25 245 000 to 29 861 000)**	**14·5% (5·3 to 25·5)**	**657·8 (569·0 to 748·4)**	**51 699 000 (44 924 000 to 58 903 000)**	**10·8% (1·1 to 21·3)**
**Central Europe, eastern Europe, and central Asia**		**326·3 (228·0 to 425·3)**	**1 385 000 (965 000 to 1 806 000)**	**–1·2% (−19·8 to 20·8)**	**340·7 (310·4 to 374·2)**	**1 421 000 (1 275 000 to 1 572 000)**	**–5·3% (−15·5 to 8·1)**	**667·0 (557·0 to 777·8)**	**2 806 000 (2 368 000 to 3 300 000)**	**–3·3% (−13·8 to 8·6)**
Central Asia		449·7 (262·4 to 619·7)	426 000 (248 000 to 589 000)	7·8% (−32·8 to 70·7)	323·7 (294·9 to 355·8)	314 000 (286 000 to 345 000)	16·1% (0·5 to 33·5)	773·4 (593·0 to 947·5)	740 000 (567 000 to 907 000)	11·1% (−15·4 to 47·5)
	Armenia	296·5 (95·5 to 496·2)	9 000 (3 000 to 15 000)	−3·1% (−72·1 to 282·9)	421·1 (367·8 to 475·1)	12 000 (11 000 to 14 000)	3·0% (−11·1 to 19·0)	717·5 (499·7 to 953·4)	21 000 (15 000 to 28 000)	0·4% (−34·9 to 58·5)
	Azerbaijan	449·6 (133·3 to 745·2)	46 000 (14 000 to 77 000)	7·0% (−71·8 to 315·6)	319·2 (288·1 to 354·0)	33 000 (30 000 to 37 000)	33·5% (10·2 to 59·4)	768·8 (450·7 to 1 066·5)	79 000 (47 000 to 110 000)	16·6% (−39·3 to 137·2)
	Georgia	357·5 (110·3 to 573·4)	13 000 (4 000 to 21 000)	−15·5% (−77·4 to 210·2)	392·0 (355·8 to 433·0)	15 000 (13 000 to 16 000)	8·2% (−4·5 to 23·3)	749·5 (494·7 to 973·4)	28 000 (18 000 to 36 000)	−4·6% (−44·4 to 69·5)
	Kazakhstan	475·8 (136·7 to 769·9)	90 000 (26 000 to 145 000)	9·0% (−72·4 to 304·0)	349·1 (312·5 to 396·4)	67 000 (59 000 to 76 000)	1·7% (−11·7 to 18·3)	824·9 (474·7 to 1 112·7)	156 000 (90 000 to 210 000)	5·8% (−43·7 to 98·9)
	Kyrgyzstan	416·9 (112·5 to 660·2)	28 000 (8 000 to 45 000)	−9·4% (−78·6 to 329·7)	320·3 (289·9 to 352·2)	23 000 (21 000 to 25 000)	28·6% (6·3 to 56·2)	737·2 (433·4 to 987·6)	51 000 (31 000 to 69 000)	4·0% (−48·0 to 129·9)
	Mongolia	403·0 (82·2 to 672·8)	13 000 (3 000 to 22 000)	23·9% (−71·9 to 472·3)	439·3 (388·8 to 493·5)	15 000 (13 000 to 16 000)	40·3% (24·7 to 57·4)	842·2 (528·6 to 1 113·5)	28 000 (18 000 to 37 000)	31·9% (−25·7 to 139·5)
	Tajikistan	417·8 (113·0 to 698·8)	41 000 (11 000 to 69 000)	−18·3% (−78·0 to 213·4)	327·6 (292·8 to 363·4)	36 000 (32 000 to 39 000)	36·5% (14·4 to 60·9)	745·4 (445·2 to 1 032·5)	77 000 (47 000 to 106 000)	−0·8% (−46·7 to 110·3)
	Turkmenistan	459·8 (115·9 to 748·7)	24 000 (6 000 to 39 000)	18·5% (−70·9 to 300·8)	266·8 (239·1 to 296·4)	14 000 (13 000 to 16 000)	46·8% (18·7 to 80·2)	726·6 (374·9 to 1 012·0)	38 000 (20 000 to 53 000)	27·5% (−38·8 to 165·9)
	Uzbekistan	479·2 (142·1 to 765·4)	162 000 (48 000 to 261 000)	19·8% (−65·0 to 421·7)	281·3 (253·6 to 311·4)	100 000 (90 000 to 110 000)	37·1% (11·8 to 66·1)	760·5 (423·6 to 1 045·6)	262 000 (148 000 to 359 000)	25·7% (−39·2 to 170·9)
Central Europe		387·9 (266·4 to 516·7)	469 000 (319 000 to 622 000)	1·0% (−24·5 to 36·9)	408·9 (372·0 to 451·6)	478 000 (431 000 to 534 000)	−4·6% (−14·8 to 8·4)	796·8 (655·6 to 937·0)	948 000 (787 000 to 1 126 000)	−1·9% (−14·6 to 14·1)
	Albania	437·1 (113·6 to 705·2)	12 000 (3 000 to 19 000)	2·5% (−74·1 to 259·8)	372·3 (324·8 to 424·3)	9 000 (8 000 to 10 000)	−0·1% (−17·5 to 21·1)	809·4 (490·0 to 1 087·1)	21 000 (12 000 to 28 000)	1·3% (−43·4 to 81·9)
	Bosnia and Herzegovina	379·1 (107·0 to 617·1)	13 000 (4 000 to 21 000)	16·2% (−70·4 to 325·8)	519·8 (458·2 to 580·7)	17 000 (15 000 to 19 000)	−5·3% (−11·9 to 2·3)	898·8 (614·2 to 1 157·1)	30 000 (20 000 to 38 000)	2·7% (−34·0 to 51·7)
	Bulgaria	429·1 (118·8 to 682·0)	30 000 (8 000 to 47 000)	6·3% (−69·9 to 254·0)	383·1 (335·6 to 434·4)	22 000 (19 000 to 25 000)	−0·6% (−18·1 to 27·1)	812·1 (499·4 to 1 073·1)	52 000 (30 000 to 69 000)	3·0% (−38·5 to 75·9)
	Croatia	443·8 (122·6 to 705·1)	20 000 (6 000 to 32 000)	−17·0% (−78·2 to 221·2)	456·7 (402·8 to 521·3)	20 000 (18 000 to 24 000)	−21·7% (−32·9 to −9·1)	900·5 (579·3 to 1 179·1)	41 000 (26 000 to 54 000)	−19·5% (−51·4 to 33·0)
	Czechia	450·3 (126·9 to 694·6)	51 000 (15 000 to 79 000)	1·4% (−71·1 to 231·9)	375·8 (332·9 to 422·1)	35 000 (31 000 to 40 000)	−2·3% (−18·7 to 20·9)	826·0 (507·9 to 1 087·9)	87 000 (51 000 to 115 000)	−0·3% (−42·8 to 67·7)
	Hungary	375·0 (118·2 to 588·8)	37 000 (12 000 to 58 000)	−12·6% (−73·7 to 229·6)	486·1 (431·8 to 544·4)	46 000 (41 000 to 53 000)	−12·4% (−23·9 to 3·3)	861·0 (591·0 to 1 105·3)	83 000 (57 000 to 108 000)	−12·5% (−44·3 to 35·9)
	Montenegro	333·3 (89·4 to 532·7)	2 000 (1 000 to 3 000)	−4·7% (−73·3 to 249·2)	437·6 (388·5 to 487·1)	3 000 (2 000 to 3 000)	−7·5% (−14·6 to 2·5)	770·8 (523·4 to 998·7)	5 000 (3 000 to 6 000)	−6·3% (−38·0 to 42·7)
	North Macedonia	341·9 (96·4 to 539·1)	8 000 (2 000 to 12 000)	−6·0% (−76·2 to 191·6)	464·4 (407·0 to 521·3)	10 000 (9 000 to 11 000)	−4·7% (−15·9 to 15·5)	806·3 (547·2 to 1 028·2)	18 000 (12 000 to 23 000)	−5·3% (−39·4 to 44·7)
	Poland	350·7 (236·8 to 458·2)	141 000 (95 000 to 185 000)	18·1% (−14·7 to 59·7)	304·7 (266·5 to 345·8)	129 000 (109 000 to 152 000)	4·0% (−5·3 to 13·4)	655·4 (534·7 to 779·2)	271 000 (220 000 to 323 000)	11·1% (−4·7 to 30·2)
	Romania	398·1 (121·7 to 617·6)	78 000 (24 000 to 122 000)	−3·1% (−69·7 to 238·3)	539·9 (482·8 to 609·4)	102 000 (90 000 to 116 000)	2·3% (−12·9 to 21·3)	938·0 (640·1 to 1 196·8)	179 000 (124 000 to 230 000)	−0·1% (−34·0 to 57·5)
	Serbia	371·2 (109·1 to 567·6)	36 000 (11 000 to 55 000)	−12·8% (−73·9 to 217·2)	434·4 (393·9 to 482·7)	41 000 (36 000 to 46 000)	−8·0% (−20·1 to 6·0)	805·6 (529·5 to 1 020·8)	77 000 (50 000 to 97 000)	−10·3% (−44·4 to 44·0)
	Slovakia	458·3 (136·9 to 727·4)	27 000 (8000 to 42 000)	6·2% (−69·1 to 235·9)	519·1 (464·3 to 580·3)	29 000 (26 000 to 33 000)	−14·7% (−23·4 to −5·5)	977·4 (631·9 to 1 257·2)	56 000 (35 000 to 73 000)	−6·0% (−40·5 to 46·7)
	Slovenia	376·7 (120·6 to 590·9)	8 000 (3000 to 13 000)	−18·5% (−77·4 to 168·8)	373·0 (319·1 to 430·2)	7 000 (6000 to 8000)	0·1% (−17·4 to 26·0)	749·7 (481·6 to 980·0)	15 000 (9 000 to 20 000)	−10·2% (−46·7 to 57·5)
Eastern Europe		226·5 (152·2 to 303·9)	489 000 (333 000 to 658 000)	−15·6% (−36·9 to 10·4)	302·3 (265·4 to 339·1)	629 000 (550 000 to 710 000)	−11·8% (−22·2 to 4·0)	528·8 (425·3 to 623·6)	1 118 000 (919 000 to 1 331 000)	−13·5% (−25·2 to −0·2)
	Belarus	234·2 (65·1 to 384·3)	23 000 (6000 to 37 000)	−25·5% (−78·7 to 161·6)	308·7 (275·5 to 347·3)	29 000 (26 000 to 33 000)	−18·7% (−31·6 to −2·7)	542·9 (361·0 to 700·1)	52 000 (35 000 to 67 000)	−21·8% (−51·5 to 23·7)
	Estonia	420·7 (126·8 to 632·4)	6 000 (2000 to 9 000)	17·4% (−65·4 to 340·7)	425·0 (377·0 to 478·7)	6 000 (5000 to 7000)	−6·3% (−14·2 to 2·7)	845·7 (550·0 to 1 086·5)	12 000 (8000 to 15 000)	4·2% (−34·1 to 64·0)
	Latvia	362·8 (117·0 to 571·2)	7 000 (2000 to 12 000)	16·5% (−65·0 to 377·4)	396·8 (348·4 to 446·7)	8 000 (7000 to 9000)	1·9% (−8·0 to 16·3)	759·5 (501·2 to 981·0)	15 000 (10 000 to 20 000)	8·4% (−31·0 to 72·4)
	Lithuania	389·2 (123·9 to 586·2)	12 000 (4000 to 18 000)	6·7% (−68·6 to 282·7)	391·4 (348·8 to 437·0)	11 000 (10 000 to 13 000)	−8·1% (−18·5 to 3·6)	780·6 (519·4 to 985·9)	23 000 (15 000 to 29 000)	−1·3% (−40·1 to 58·0)
	Moldova	247·5 (76·3 to 407·6)	9 000 (3000 to 15 000)	−22·5% (−79·0 to 183·2)	353·1 (314·2 to 391·6)	13 000 (11 000 to 14 000)	−13·8% (−25·7 to 0·1)	600·7 (418·8 to 776·8)	22 000 (15 000 to 28 000)	−17·6% (−48·7 to 27·5)
	Russia	211·2 (143·6 to 281·8)	321 000 (220 000 to 436 000)	−15·2% (−30·1 to −1·9)	287·3 (246·2 to 327·5)	418 000 (364 000 to 478 000)	−11·1% (−21·1 to 6·0)	498·5 (404·5 to 592·2)	739 000 (609 000 to 891 000)	−12·8% (−21·6 to −2·5)
	Ukraine	253·1 (77·6 to 403·7)	111 000 (34 000 to 176 000)	−16·0% (−73·4 to 176·9)	333·8 (297·2 to 369·9)	145 000 (127 000 to 162 000)	−11·1% (−22·6 to 4·1)	586·9 (393·9 to 748·9)	255 000 (175 000 to 328 000)	−13·3% (−43·9 to 31·6)
**High income**		**343·7 (233·4 to 454·9)**	**4 073 000 (2 740 000 to 5 373 000)**	**8·9% (−12·4 to 27·7)**	**295·2 (271·2 to 321·3)**	**3 116 000 (2 848 000 to 3 406 000)**	**4·4% (−6·4 to 15·4)**	**638·9 (524·1 to 757·3)**	**7 189 000 (5 826 000 to 8 590 000)**	**6·8% (−4·2 to 17·3)**
Australasia		316·1 (123·3 to 491·0)	102 000 (39 000 to 157 000)	−3·6% (−62·1 to 141·4)	248·1 (225·4 to 273·7)	71 000 (64 000 to 78 000)	−15·6% (−23·2 to −6·6)	564·3 (369·3 to 737·3)	172 000 (109 000 to 230 000)	−9·3% (−41·6 to 38·8)
	Australia	316·5 (100·8 to 512·0)	85 000 (27 000 to 137 000)	−1·2% (−70·3 to 227·2)	251·9 (227·2 to 278·8)	60 000 (54 000 to 66 000)	−17·2% (−24·9 to −8·0)	568·4 (344·8 to 763·5)	145 000 (86 000 to 198 000)	−9·0% (−47·2 to 53·6)
	New Zealand	312·9 (122·1 to 471·4)	16 000 (6000 to 24 000)	−14·2% (−64·2 to 119·6)	229·5 (206·5 to 254·0)	11 000 (10 000 to 12 000)	−5·5% (−26·8 to 14·0)	542·5 (344·3 to 698·5)	27 000 (17 000 to 35 000)	−10·7% (−42·8 to 47·3)
High-income Asia Pacific		276·7 (168·6 to 384·4)	563 000 (345 000 to 767 000)	5·4% (−28·5 to 49·2)	231·4 (212·5 to 251·5)	394 000 (358 000 to 430 000)	4·5% (−14·2 to 22·8)	508·1 (398·5 to 618·1)	957 000 (735 000 to 1 174 000)	5·0% (−15·6 to 28·9)
	Brunei	418·3 (132·5 to 644·5)	2 000 (1000 to 3000)	−21·4% (−71·6 to 194·9)	365·5 (333·8 to 399·7)	2 000 (2000 to 2000)	−5·9% (−16·6 to 5·8)	783·8 (496·0 to 1 023·9)	4 000 (2000 to 5000)	−14·8% (−47·5 to 54·9)
	Japan	261·6 (169·0 to 352·3)	377 000 (250 000 to 503 000)	11·3% (−7·7 to 31·9)	212·5 (192·0 to 233·9)	225 000 (203 000 to 248 000)	12·6% (−11·4 to 35·8)	474·1 (379·6 to 568·2)	602 000 (473 000 to 730 000)	11·9% (−4·5 to 27·1)
	Singapore	271·3 (78·8 to 431·8)	15 000 (4000 to 24 000)	14·6% (−70·7 to 332·6)	246·2 (225·4 to 268·6)	13 000 (12 000 to 15 000)	1·0% (−10·4 to 14·2)	517·5 (322·8 to 679·6)	29 000 (17 000 to 38 000)	7·7% (−39·0 to 81·0)
	South Korea	315·4 (84·6 to 484·7)	169 000 (45 000 to 262 000)	−5·2% (−73·8 to 207·0)	278·4 (252·0 to 306·2)	154 000 (136 000 to 174 000)	−14·7% (−25·0 to −4·4)	593·8 (357·9 to 768·0)	323 000 (197 000 to 424 000)	−9·9% (−49·4 to 47·5)
High-income North America		334·7 (218·9 to 460·7)	1 316 000 (865 000 to 1 780 000)	12·7% (−13·1 to 37·0)	354·6 (319·3 to 392·4)	1 350 000 (1 199 000 to 1 511 000)	3·0% (−4·1 to 10·2)	689·4 (555·4 to 821·8)	2 666 000 (2 152 000 to 3 202 000)	7·5% (−4·8 to 18·6)
	Canada	272·3 (76·4 to 430·2)	110 000 (32 000 to 171 000)	3·3% (−66·2 to 214·5)	263·3 (239·6 to 289·1)	89 000 (81 000 to 98 000)	2·3% (−8·4 to 14·0)	535·6 (340·2 to 693·1)	199 000 (121 000 to 261 000)	2·8% (−35·1 to 62·5)
	Greenland	554·6 (167·4 to 870·9)	330 (100 to 520)	−10·8% (−73·2 to 209·6)	290·9 (264·5 to 320·9)	150 (140 to 170)	2·4% (−12·2 to 19·4)	845·5 (455·8 to 1 167·6)	490 (260 to 680)	−6·7% (−52·6 to 101·9)
	USA	341·6 (217·9 to 466·9)	1 205 000 (771 000 to 1 614 000)	13·7% (−13·5 to 37·7)	365·3 (327·5 to 405·1)	1 261 000 (1 113 000 to 1 419 000)	3·4% (−4·3 to 10·6)	706·9 (573·1 to 837·0)	2 466 000 (2 001 000 to 2 953 000)	8·1% (−4·8 to 20·3)
Southern Latin America		339·7 (161·0 to 515·6)	233 000 (113 000 to 355 000)	7·3% (−44·9 to 120·5)	391·4 (356·4 to 425·4)	266 000 (241 000 to 291 000)	3·9% (−5·2 to 14·5)	731·1 (542·5 to 906·6)	499 000 (370 000 to 621 000)	5·4% (−22·6 to 41·4)
	Argentina	277·7 (71·5 to 454·7)	127 000 (33 000 to 207 000)	11·9% (−72·5 to 301·6)	366·6 (331·2 to 401·3)	166 000 (149 000 to 183 000)	2·2% (−5·8 to 11·4)	644·4 (435·3 to 830·4)	293 000 (197 000 to 380 000)	6·1% (−30·8 to 57·2)
	Chile	478·8 (148·1 to 773·6)	92 000 (29 000 to 149 000)	−0·1% (−74·0 to 254·5)	447·8 (404·6 to 496·1)	87 000 (78 000 to 97 000)	2·8% (−11·1 to 16·1)	926·5 (579·5 to 1 215·7)	179 000 (112 000 to 236 000)	1·3% (−41·2 to 77·8)
	Uruguay	394·4 (95·3 to 636·9)	14 000 (3 000 to 23 000)	13·7% (−73·9 to 305·3)	372·9 (334·6 to 414·1)	13 000 (12 000 to 15 000)	7·8% (−5·1 to 21·1)	767·4 (460·9 to 1 017·6)	27 000 (16 000 to 36 000)	10·8% (−38·3 to 90·5)
Western Europe		381·1 (238·1 to 502·5)	1 859 000 (1 139 000 to 2 480 000)	8·3% (−21·3 to 44·3)	258·5 (234·8 to 285·5)	1 035 000 (946 000 to 1 143 000)	1·7% (−10·9 to 15·5)	639·6 (495·8 to 764·1)	2 894 000 (2 197 000 to 3 534 000)	5·5% (−12·4 to 25·1)
	Andorra	348·7 (100·7 to 530·1)	310 (90 to 460)	−5·9% (−64·3 to 142·6)	214·0 (193·6 to 236·4)	170 (150 to 190)	−6·8% (−20·2 to 6·4)	562·7 (319·8 to 745·6)	470 (260 to 630)	−6·2% (−46·3 to 58·8)
	Austria	332·7 (99·5 to 511·7)	31 000 (10 000 to 48 000)	3·4% (−61·0 to 315·7)	401·4 (361·7 to 447·0)	36 000 (32 000 to 41 000)	24·5% (11·5 to 37·7)	734·1 (488·2 to 931·2)	68 000 (45 000 to 86 000)	14·0% (−23·4 to 90·4)
	Belgium	425·6 (125·9 to 656·7)	56 000 (16 000 to 85 000)	14·1% (−69·6 to 297·4)	176·9 (160·4 to 196·9)	18 000 (16 000 to 20 000)	12·6% (−1·2 to 28·5)	602·5 (302·3 to 832·2)	74 000 (34 000 to 104 000)	13·7% (−48·6 to 141·4)
	Cyprus	287·6 (81·0 to 450·5)	4 000 (1000 to 6000)	−5·5% (−74·5 to 267·4)	230·4 (203·7 to 261·8)	3 000 (3000 to 3000)	−13·4% (−27·3 to 5·2)	517·9 (305·6 to 690·0)	7 000 (4 000 to 9 000)	−9·2% (−48·2 to 56·4)
	Denmark	266·1 (74·9 to 413·4)	17 000 (5000 to 26 000)	−1·5% (−65·6 to 208·5)	245·0 (218·8 to 277·4)	13 000 (11 000 to 15 000)	−5·3% (−18·7 to 12·0)	511·0 (311·0 to 663·6)	30 000 (17 000 to 40 000)	−3·4% (−38·5 to 56·1)
	Finland	336·3 (91·5 to 529·4)	20 000 (6000 to 32 000)	9·3% (−68·2 to 217·8)	201·5 (183·1 to 222·0)	10 000 (9000 to 11 000)	−3·5% (−18·3 to 12·3)	537·7 (303·9 to 726·7)	30 000 (16 000 to 42 000)	4·1% (−45·2 to 86·2)
	France	426·9 (155·3 to 661·3)	323 000 (119 000 to 501 000)	9·7% (−60·4 to 274·1)	191·0 (172·0 to 213·9)	112 000 (101 000 to 126 000)	−4·3% (−18·5 to 11·4)	617·9 (340·6 to 864·1)	436 000 (230 000 to 617 000)	4·9% (−42·7 to 107·7)
	Germany	538·6 (163·0 to 815·6)	548 000 (171 000 to 827 000)	17·9% (−61·8 to 223·9)	349·2 (318·2 to 387·3)	256 000 (233 000 to 283 000)	−2·4% (−12·1 to 8·0)	887·8 (521·3 to 1 159·8)	804 000 (432 000 to 1 083 000)	9·0% (−36·6 to 88·5)
	Greece	267·3 (68·7 to 422·8)	28 000 (7000 to 44 000)	0·1% (−74·9 to 218·2)	321·5 (290·3 to 352·2)	32 000 (29 000 to 37 000)	30·3% (13·9 to 48·5)	588·8 (390·2 to 752·6)	61 000 (40 000 to 78 000)	14·6% (−30·4 to 85·5)
	Iceland	308·1 (88·0 to 484·8)	1 000 (0 to 2000)	11·0% (−66·5 to 250·3)	261·1 (237·5 to 285·6)	860 (780 to 940)	−3·1% (−15·1 to 9·8)	569·2 (346·5 to 749·5)	2 000 (1 000 to 3 000)	4·1% (−35·3 to 70·9)
	Ireland	382·9 (113·0 to 589·7)	20 000 (6000 to 30 000)	13·7% (−64·2 to 302·2)	222·3 (200·7 to 246·5)	10 000 (9000 to 11 000)	−3·8% (−17·4 to 12·2)	605·2 (344·7 to 814·6)	30 000 (16 000 to 41 000)	6·6% (−41·6 to 93·7)
	Israel	307·6 (105·5 to 466·1)	30 000 (10 000 to 46 000)	14·7% (−60·3 to 314·5)	291·5 (262·9 to 321·9)	28 000 (25 000 to 31 000)	−6·7% (−16·1 to 3·1)	599·1 (393·0 to 770·7)	59 000 (38 000 to 75 000)	3·2% (−34·0 to 60·4)
	Italy	253·4 (174·1 to 344·3)	172 000 (116 000 to 234 000)	−3·7% (−28·3 to 25·3)	211·6 (192·2 to 234·5)	109 000 (99 000 to 121 000)	−4·7% (−25·7 to 16·0)	465·0 (377·0 to 561·2)	281 000 (224 000 to 344 000)	−4·2% (−20·6 to 13·7)
	Luxembourg	439·9 (139·5 to 663·0)	3 000 (1 000 to 5 000)	7·6% (−66·2 to 252·4)	221·3 (193·0 to 253·6)	1 000 (1 000 to 2 000)	−12·8% (−31·2 to 9·0)	661·3 (368·2 to 884·0)	4 000 (2 000 to 6 000)	−0·2% (−47·6 to 88·0)
	Malta	290·5 (82·7 to 458·4)	1 000 (0 to 2 000)	16·6% (−65·0 to 314·8)	237·2 (217·9 to 261·2)	920 (840 to 1010)	2·6% (−8·6 to 12·8)	527·7 (326·1 to 696·9)	2 000 (1 000 to 3 000)	9·9% (−35·3 to 82·8)
	Monaco	356·4 (121·8 to 564·0)	150 (50 to 240)	6·4% (−67·8 to 269·3)	291·3 (261·3 to 325·2)	120 (100 to 140)	−4·0% (−14·3 to 6·4)	647·7 (406·4 to 860·7)	270 (170 to 370)	1·4% (−40·9 to 70·0)
	Netherlands	347·3 (99·4 to 537·7)	65 000 (18 000 to 99 000)	11·7% (−66·5 to 292·6)	223·0 (200·9 to 248·6)	34 000 (31 000 to 38 000)	−2·2% (−19·9 to 17·1)	570·3 (319·8 to 758·6)	99 000 (52 000 to 133 000)	5·8% (−41·7 to 88·3)
	Norway	413·3 (247·4 to 567·6)	24 000 (15 000 to 33 000)	−2·2% (−31·7 to 33·8)	312·7 (266·3 to 363·7)	16 000 (13 000 to 18 000)	−16·9% (−29·6 to 2·1)	726·0 (560·7 to 875·4)	40 000 (30 000 to 49 000)	−9·1% (−24·4 to 9·0)
	Portugal	269·2 (73·8 to 420·7)	33 000 (9 000 to 51 000)	17·4% (−67·9 to 364·7)	207·5 (186·9 to 232·0)	19 000 (17 000 to 21 000)	5·4% (−11·2 to 22·8)	476·7 (277·2 to 634·4)	52 000 (27 000 to 70 000)	11·8% (−38·3 to 101·3)
	San Marino	284·9 (78·7 to 448·8)	90 (30 to 150)	−1·7% (−70·8 to 228·4)	259·8 (235·1 to 285·0)	90 (80 to 100)	−6·1% (−16·0 to 3·4)	544·6 (328·5 to 706·4)	180 (110 to 240)	−3·8% (−41·9 to 60·5)
	Spain	269·3 (85·1 to 429·5)	132 000 (43 000 to 209 000)	5·7% (−65·9 to 318·7)	261·7 (236·9 to 292·1)	132 000 (117 000 to 151 000)	−5·1% (−16·1 to 6·6)	531·0 (347·7 to 695·8)	264 000 (172 000 to 348 000)	0·1% (−38·5 to 63·7)
	Sweden	245·2 (105·3 to 363·5)	27 000 (11 000 to 40 000)	−15·0% (−62·0 to 99·2)	282·4 (252·1 to 311·6)	26 000 (24 000 to 29 000)	6·2% (−9·6 to 24·2)	527·6 (378·3 to 657·8)	53 000 (37 000 to 67 000)	−4·8% (−31·6 to 38·6)
	Switzerland	314·1 (92·6 to 495·1)	31 000 (9 000 to 48 000)	−7·8% (−72·0 to 236·9)	285·5 (253·2 to 324·2)	23 000 (20 000 to 26 000)	−0·5% (−15·1 to 17·6)	599·6 (370·1 to 782·7)	54 000 (31 000 to 72 000)	−4·4% (−42·0 to 59·4)
	UK	413·6 (276·6 to 554·0)	289 000 (194 000 to 383 000)	2·4% (−12·3 to 15·4)	249·8 (214·6 to 287·0)	153 000 (131 000 to 176 000)	22·5% (2·4 to 45·1)	663·4 (523·3 to 809·0)	442 000 (347 000 to 537 000)	9·1% (−1·7 to 20·7)
**Latin America and Caribbean**		**470·6 (346·3 to 607·6)**	**2 811 000 (2 070 000 to 3 628 000)**	**–9·7% (−29·1 to 16·0)**	**482·7 (435·7 to 530·3)**	**2 899 000 (2 604 000 to 3 203 000)**	**10·3% (−2·1 to 22·2)**	**953·4 (813·1 to 1 103·3)**	**5 709 000 (4 853 000 to 6 613 000)**	**–0·5% (−12·5 to 13·8)**
Andean Latin America		561·9 (309·6 to 821·7)	372 000 (205 000 to 544 000)	−4·5% (−53·7 to 105·5)	478·8 (430·5 to 528·4)	314 000 (283 000 to 347 000)	−7·1% (−14·5 to 0·6)	1 040·7 (775·5 to 1 314·2)	686 000 (511 000 to 868 000)	−5·7% (−35·0 to 37·6)
	Bolivia	421·1 (105·8 to 704·0)	49 000 (12 000 to 82 000)	−20·0% (−80·4 to 236·5)	401·3 (357·8 to 451·7)	46 000 (41 000 to 52 000)	−3·1% (−12·0 to 6·5)	822·4 (505·8 to 1 112·2)	95 000 (58 000 to 129 000)	−12·6% (−54·3 to 62·0)
	Ecuador	710·8 (226·2 to 1 140·8)	129 000 (41 000 to 207 000)	4·4% (−70·0 to 359·3)	460·3 (412·6 to 511·5)	82 000 (74 000 to 91 000)	−6·5% (−15·3 to 3·3)	1 171·1 (691·3 to 1 600·2)	211 000 (124 000 to 288 000)	−0·2% (−48·5 to 96·9)
	Peru	534·4 (155·1 to 856·1)	194 000 (56 000 to 311 000)	−5·2% (−72·4 to 314·9)	511·9 (462·3 to 566·1)	186 000 (167 000 to 206 000)	−7·8% (−15·7 to 1·1)	1 046·3 (667·5 to 1 377·7)	380 000 (243 000 to 503 000)	−6·5% (−46·2 to 68·0)
Caribbean		371·1 (236·3 to 507·4)	177 000 (114 000 to 241 000)	−3·3% (−37·2 to 47·2)	467·9 (431·5 to 505·1)	222 000 (204 000 to 240 000)	−2·7% (−8·7 to 3·7)	839·0 (691·7 to 996·8)	398 000 (329 000 to 472 000)	−3·0% (−19·3 to 17·1)
	Antigua and Barbuda	662·3 (195·6 to 1 027·4)	610 (180 to 950)	−5·4% (−71·1 to 309·8)	379·7 (350·6 to 412·5)	330 (300 to 350)	0·8% (−13·8 to 16·3)	1 041·9 (583·7 to 1 406·1)	940 (510 to 1270)	−3·2% (−49·6 to 99·4)
	The Bahamas	493·1 (155·9 to 765·5)	2 000 (1 000 to 3 000)	−12·3% (−72·8 to 199·4)	509·1 (462·0 to 561·3)	2 000 (2 000 to 2 000)	−10·0% (−17·6 to −0·8)	1 002·1 (659·1 to 1 281·8)	4 000 (3 000 to 5 000)	−11·2% (−44·0 to 43·2)
	Barbados	463·0 (148·0 to 739·6)	1 000 (0 to 2 000)	−13·8% (−77·4 to 220·3)	515·1 (468·2 to 564·5)	2 000 (1 000 to 2 000)	−9·6% (−17·5 to −1·0)	978·1 (656·9 to 1 254·5)	3 000 (2 000 to 4 000)	−11·6% (−47·3 to 46·4)
	Belize	401·3 (116·3 to 686·1)	2 000 (1 000 to 3 000)	8·8% (−72·9 to 402·8)	548·1 (498·5 to 596·7)	2 000 (2 000 to 3 000)	7·1% (−3·4 to 18·8)	949·4 (657·5 to 1 237·4)	4 000 (3 000 to 5 000)	7·8% (−31·4 to 77·5)
	Bermuda	452·6 (139·4 to 709·8)	300 (90 to 470)	−18·1% (−74·0 to 201·7)	472·5 (428·7 to 516·7)	320 (280 to 360)	−16·8% (−23·7 to −9·2)	925·2 (605·3 to 1 195·9)	620 (410 to 810)	−17·4% (−48·9 to 36·7)
	Cuba	270·1 (78·5 to 445·2)	29 000 (9 000 to 48 000)	−10·0% (−76·4 to 253·7)	270·3 (251·8 to 291·8)	27 000 (25 000 to 30 000)	−16·1% (−26·1 to −4·2)	540·4 (346·2 to 721·0)	56 000 (36 000 to 76 000)	−13·2% (−49·6 to 48·0)
	Dominica	665·6 (224·1 to 1 029·3)	460 (150 to 710)	7·9% (−69·3 to 384·6)	595·9 (529·3 to 667·6)	410 (360 to 460)	−7·5% (−14·9 to 0·6)	1 261·5 (807·4 to 1 646·9)	860 (550 to 1130)	0·0% (−41·8 to 73·2)
	Dominican Republic	423·0 (129·2 to 696·3)	47 000 (14 000 to 77 000)	29·2% (−63·5 to 408·4)	576·8 (521·8 to 625·7)	64 000 (58 000 to 69 000)	22·0% (12·5 to 32·9)	999·7 (689·5 to 1 293·7)	110 000 (76 000 to 142 000)	24·9% (−20·0 to 98·4)
	Grenada	477·2 (131·8 to 790·3)	500 (140 to 820)	2·8% (−68·5 to 297·7)	557·1 (507·0 to 611·6)	580 (520 to 640)	−5·7% (−14·7 to 3·9)	1 034·3 (677·4 to 1 356·9)	1000 (1000 to 1000)	−1·9% (−37·9 to 62·6)
	Guyana	502·4 (138·6 to 804·2)	4 000 (1 000 to 6 000)	17·4% (−67·0 to 302·5)	602·9 (547·4 to 665·9)	5000 (4000 to 5000)	−1·9% (−9·0 to 5·0)	1 105·4 (733·7 to 1 427·2)	8000 (6000 to 11 000)	6·0% (−33·3 to 58·8)
	Haiti	324·3 (80·6 to 567·8)	42 000 (10 000 to 74 000)	−16·2% (−82·8 to 306·5)	465·3 (420·9 to 513·1)	59 000 (54 000 to 65 000)	−19·6% (−25·2 to −13·2)	789·6 (538·2 to 1 042·2)	101 000 (69 000 to 134 000)	−18·3% (−49·7 to 30·4)
	Jamaica	426·3 (124·7 to 693·1)	12 000 (4000 to 20 000)	−2·2% (−71·1 to 250·9)	598·1 (540·5 to 652·2)	17 000 (15 000 to 18 000)	−4·1% (−11·8 to 3·1)	1 024·4 (723·0 to 1 318·1)	29 000 (21 000 to 37 000)	−3·4% (−35·7 to 47·9)
	Puerto Rico	460·9 (129·5 to 727·0)	17 000 (5000 to 26 000)	−15·3% (−74·8 to 167·0)	502·4 (456·0 to 551·1)	17 000 (15 000 to 20 000)	−14·2% (−21·9 to −6·5)	963·3 (619·6 to 1 259·1)	34 000 (22 000 to 45 000)	−14·7% (−47·0 to 34·9)
	Saint Kitts and Nevis	564·3 (166·3 to 909·6)	340 (100 to 540)	−20·8% (−75·6 to 219·4)	528·2 (474·0 to 592·7)	320 (280 to 370)	−18·8% (−27·5 to −10·0)	1 092·4 (694·8 to 1 427·4)	660 (420 to 860)	−19·8% (−50·5 to 38·2)
	Saint Lucia	519·3 (139·4 to 852·3)	950 (260 to 1560)	−10·7% (−77·7 to 256·3)	740·9 (675·0 to 811·5)	1000 (1000 to 1000)	−14·3% (−20·0 to −7·3)	1 260·2 (878·6 to 1 609·9)	2000 (2000 to 3000)	−12·9% (−43·9 to 35·3)
	Saint Vincent and the Grenadines	494·2 (132·8 to 812·4)	580 (150 to 940)	7·1% (−71·0 to 404·8)	571·8 (515·1 to 629·0)	670 (590 to 740)	2·0% (−7·5 to 12·7)	1 066·0 (716·2 to 1 381·9)	1000 (1000 to 2000)	4·3% (−38·1 to 71·4)
	Suriname	542·9 (184·4 to 868·1)	3000 (1000 to 5000)	7·4% (−64·9 to 410·5)	588·8 (527·3 to 656·9)	3000 (3000 to 4000)	1·2% (−7·8 to 12·3)	1 131·7 (761·1 to 1 451·9)	7000 (4000 to 9000)	4·1% (−36·2 to 79·3)
	Trinidad and Tobago	593·4 (168·6 to 955·2)	9000 (2000 to 14 000)	1·8% (−69·1 to 280·7)	781·0 (706·1 to 858·5)	11 000 (10 000 to 12 000)	−1·4% (−7·5 to 5·7)	1 374·5 (944·8 to 1 773·3)	20 000 (14 000 to 25 000)	0·0% (−35·6 to 54·2)
	Virgin Islands	500·4 (152·1 to 777·4)	470 (150 to 720)	−2·6% (−70·5 to 285·6)	537·7 (490·8 to 592·2)	470 (420 to 540)	0·2% (−7·7 to 9·5)	1 038·1 (683·4 to 1 328·5)	940 (610 to 1230)	−1·2% (−38·4 to 60·0)
Central Latin America		537·4 (384·2 to 714·3)	1 367 000 (977 000 to 1 819 000)	−9·3% (−33·3 to 25·2)	535·9 (477·0 to 592·0)	1 366 000 (1 213 000 to 1 512 000)	5·3% (−8·7 to 18·3)	1 073·3 (905·6 to 1 258·9)	2 732 000 (2 297 000 to 3 214 000)	−2·5% (−16·5 to 16·3)
	Colombia	504·4 (135·2 to 858·9)	250 000 (67 000 to 428 000)	−8·2% (−77·3 to 298·9)	413·5 (380·3 to 447·2)	203 000 (187 000 to 220 000)	6·3% (−6·4 to 20·2)	917·9 (549·7 to 1 273·7)	453 000 (271 000 to 630 000)	−2·2% (−47·4 to 92·2)
	Costa Rica	495·7 (150·0 to 804·0)	24 000 (7000 to 38 000)	0·5% (−72·0 to 277·9)	431·9 (378·8 to 489·1)	21 000 (18 000 to 24 000)	−8·2% (−16·9 to 0·6)	927·6 (579·8 to 1 245·8)	45 000 (28 000 to 60 000)	−3·8% (−45·2 to 69·1)
	El Salvador	401·8 (98·6 to 658·9)	26 000 (6000 to 43 000)	10·1% (−72·6 to 339·0)	453·0 (408·9 to 501·9)	29 000 (26 000 to 32 000)	6·1% (−4·9 to 17·1)	854·7 (556·5 to 1 120·2)	55 000 (36 000 to 73 000)	7·9% (−38·3 to 78·6)
	Guatemala	505·2 (140·3 to 844·9)	81 000 (22 000 to 136 000)	11·5% (−69·6 to 424·1)	575·5 (507·1 to 638·7)	89 000 (78 000 to 98 000)	−3·6% (−11·3 to 5·9)	1 080·7 (702·2 to 1 432·0)	169 000 (109 000 to 226 000)	2·9% (−38·3 to 73·9)
	Honduras	437·9 (96·9 to 757·0)	43 000 (10 000 to 74 000)	−9·1% (−80·1 to 375·8)	576·5 (520·3 to 635·0)	56 000 (51 000 to 62 000)	−3·4% (−12·1 to 6·5)	1 014·4 (659·8 to 1 333·2)	99 000 (65 000 to 130 000)	−6·0% (−43·7 to 63·7)
	Mexico	582·8 (400·6 to 753·8)	755 000 (518 000 to 979 000)	−11·8% (−34·5 to 14·3)	603·3 (529·1 to 679·7)	788 000 (686 000 to 890 000)	9·0% (−9·1 to 26·9)	1 186·2 (989·1 to 1 389·0)	1 543 000 (1 285 000 to 1 816 000)	−2·3% (−16·4 to 14·4)
	Nicaragua	364·8 (85·9 to 645·8)	24 000 (6000 to 42 000)	−12·2% (−78·9 to 338·9)	476·4 (428·0 to 521·9)	31 000 (28 000 to 34 000)	−8·3% (−16·6 to 1·3)	841·1 (563·5 to 1 138·4)	55 000 (37 000 to 75 000)	−10·0% (−47·1 to 48·1)
	Panama	571·5 (170·7 to 897·7)	25 000 (7000 to 39 000)	29·6% (−62·8 to 588·5)	463·9 (407·1 to 526·0)	20 000 (18 000 to 23 000)	3·6% (−6·4 to 13·7)	1 035·4 (642·0 to 1 379·4)	45 000 (28 000 to 60 000)	16·5% (−33·7 to 104·4)
	Venezuela	520·8 (149·0 to 889·5)	139 000 (40 000 to 236 000)	−10·4% (−74·5 to 299·4)	469·5 (407·9 to 533·9)	128 000 (110 000 to 147 000)	−8·3% (−16·4 to 1·2)	990·3 (604·9 to 1 371·3)	267 000 (165 000 to 366 000)	−9·4% (−46·9 to 64·2)
Tropical Latin America		389·5 (262·4 to 529·0)	895 000 (602 000 to 1 213 000)	−15·9% (−39·8 to 26·5)	433·8 (393·0 to 477·4)	998 000 (901 000 to 1 104 000)	26·6% (11·3 to 45·0)	823·3 (688·2 to 964·6)	1 893 000 (1 582 000 to 2 240 000)	2·1% (−14·7 to 28·7)
	Brazil	388·9 (262·2 to 526·5)	866 000 (581 000 to 1 166 000)	−16·5% (−39·7 to 25·5)	433·7 (392·7 to 477·8)	967 000 (872 000 to 1 070 000)	27·9% (12·0 to 47·0)	822·6 (685·4 to 963·3)	1 833 000 (1 527 000 to 2 167 000)	2·2% (−14·8 to 29·6)
	Paraguay	399·9 (124·0 to 668·6)	29 000 (9000 to 48 000)	8·6% (−66·7 to 308·4)	435·4 (397·2 to 478·6)	31 000 (28 000 to 34 000)	−8·3% (−16·2 to 0·8)	835·3 (552·4 to 1 095·8)	60 000 (40 000 to 78 000)	−0·9% (−38·7 to 53·0)
**North Africa and Middle East**		**316·2 (217·5 to 430·7)**	**1 941 000 (1 323 000 to 2 650 000)**	**1·4% (−30·7 to 44·5)**	**313·0 (286·6 to 340·5)**	**2 017 000 (1 845 000 to 2 194 000)**	**11·8% (−3·0 to 28·1)**	**629·2 (519·2 to 744·5)**	**3 957 000 (3 268 000 to 4 685 000)**	**6·3% (−13·3 to 30·2)**
North Africa and Middle East										
	Afghanistan	317·9 (70·6 to 594·2)	102 000 (23 000 to 192 000)	−18·4% (−79·9 to 417·3)	288·6 (263·2 to 314·0)	108 000 (98 000 to 118 000)	−4·9% (−14·9 to 7·1)	606·6 (351·8 to 886·8)	210 000 (129 000 to 300 000)	−12·5% (−54·4 to 72·5)
	Algeria	284·4 (72·0 to 471·3)	124 000 (31 000 to 205 000)	−17·4% (−82·1 to 221·7)	246·0 (218·3 to 274·3)	111 000 (99 000 to 124 000)	9·7% (−5·3 to 27·5)	530·4 (318·5 to 716·3)	235 000 (143 000 to 316 000)	−6·7% (−50·7 to 81·1)
	Bahrain	417·2 (115·7 to 663·3)	6000 (2000 to 9000)	−7·3% (−74·8 to 219·0)	339·6 (307·6 to 372·7)	5 000 (5 000 to 6 000)	4·4% (−12·6 to 26·0)	756·8 (464·6 to 1 002·6)	11 000 (7000 to 15 000)	−2·4% (−46·2 to 79·3)
	Egypt	303·1 (81·0 to 484·3)	319 000 (83 000 to 515 000)	6·0% (−71·5 to 406·9)	379·2 (338·9 to 418·7)	431 000 (384 000 to 476 000)	7·2% (−6·2 to 22·9)	682·3 (454·8 to 881·5)	750 000 (505 000 to 964 000)	6·7% (−35·0 to 82·2)
	Iran	305·4 (211·4 to 392·6)	248 000 (171 000 to 319 000)	−1·5% (−30·7 to 40·7)	252·3 (225·6 to 282·3)	215 000 (192 000 to 241 000)	80·4% (32·3 to 141·7)	557·7 (458·2 to 645·3)	463 000 (382 000 to 537 000)	23·9% (−1·7 to 61·0)
	Iraq	278·9 (83·4 to 449·8)	116 000 (35 000 to 190 000)	6·2% (−70·7 to 336·9)	375·7 (324·9 to 417·2)	166 000 (143 000 to 185 000)	1·2% (−11·9 to 18·3)	654·7 (457·1 to 844·7)	283 000 (199 000 to 364 000)	3·3% (−33·6 to 62·6)
	Jordan	268·8 (74·0 to 436·0)	33 000 (9 000 to 55 000)	−1·0% (−70·7 to 272·3)	335·7 (302·1 to 371·3)	43 000 (39 000 to 48 000)	−1·7% (−14·6 to 15·8)	604·5 (409·2 to 788·2)	77 000 (52 000 to 100 000)	−1·4% (−35·9 to 52·5)
	Kuwait	358·1 (95·0 to 569·3)	15 000 (4 000 to 23 000)	−6·4% (−74·5 to 254·9)	343·8 (310·9 to 380·2)	16 000 (14 000 to 18 000)	31·7% (7·4 to 58·4)	701·9 (428·9 to 917·3)	31 000 (20 000 to 41 000)	9·0% (−39·3 to 100·1)
	Lebanon	289·8 (93·5 to 476·6)	16 000 (5000 to 25 000)	0·6% (−71·9 to 356·7)	283·6 (254·1 to 315·9)	15 000 (14 000 to 17 000)	−9·3% (−23·7 to 6·8)	573·3 (373·5 to 756·8)	31 000 (20 000 to 41 000)	−4·6% (−44·2 to 64·8)
	Libya	266·7 (80·2 to 428·6)	18 000 (5000 to 29 000)	−10·3% (−75·7 to 368·6)	247·7 (222·6 to 274·4)	17 000 (16 000 to 19 000)	0·7% (−16·7 to 20·6)	514·4 (321·5 to 673·1)	35 000 (22 000 to 46 000)	−5·3% (−47·9 to 83·5)
	Morocco	281·1 (69·5 to 485·0)	103 000 (25 000 to 177 000)	9·3% (−71·4 to 353·2)	311·2 (284·0 to 340·4)	116 000 (106 000 to 127 000)	6·2% (−6·7 to 20·6)	592·3 (377·3 to 797·2)	219 000 (140 000 to 294 000)	7·6% (−36·7 to 88·5)
	Oman	321·8 (89·0 to 497·8)	14 000 (4000 to 22 000)	27·0% (−62·3 to 500·2)	293·5 (267·1 to 323·6)	15 000 (13 000 to 16 000)	5·1% (−9·5 to 22·5)	615·3 (377·4 to 795·8)	29 000 (18 000 to 37 000)	15·5% (−31·6 to 100·1)
	Palestine	279·0 (87·4 to 468·9)	15 000 (5000 to 25 000)	3·1% (−74·8 to 565·3)	282·2 (256·6 to 314·3)	16 000 (14 000 to 17 000)	−1·4% (−17·0 to 17·6)	561·2 (367·2 to 754·5)	30 000 (20 000 to 41 000)	0·8% (−41·8 to 84·1)
	Qatar	381·3 (122·2 to 587·3)	10 000 (3000 to 15 000)	−20·1% (−77·8 to 172·5)	372·4 (335·2 to 414·1)	11 000 (10 000 to 13 000)	−8·1% (−23·1 to 7·6)	753·7 (494·9 to 964·5)	21 000 (14 000 to 28 000)	−14·5% (−49·9 to 49·0)
	Saudi Arabia	518·0 (145·1 to 805·9)	187 000 (52 000 to 291 000)	30·4% (−62·4 to 457·6)	282·7 (257·8 to 310·5)	112 000 (102 000 to 123 000)	14·6% (−4·5 to 39·2)	800·7 (437·9 to 1 091·1)	298 000 (168 000 to 405 000)	24·3% (−38·5 to 151·9)
	Sudan	242·3 (64·5 to 411·8)	111 000 (30 000 to 189 000)	6·0% (−71·9 to 426·6)	255·9 (230·8 to 284·1)	122 000 (110 000 to 135 000)	32·4% (14·0 to 53·8)	498·2 (318·1 to 673·2)	233 000 (151 000 to 314 000)	18·1% (−37·2 to 127·9)
	Syria	229·3 (61·2 to 381·0)	32 000 (9 000 to 53 000)	7·7% (−72·2 to 398·5)	295·0 (265·2 to 323·6)	42 000 (37 000 to 46 000)	−6·7% (−16·3 to 4·8)	524·3 (356·2 to 684·3)	74 000 (50 000 to 96 000)	−0·9% (−39·0 to 55·2)
	Tunisia	257·9 (75·8 to 429·9)	29 000 (9 000 to 49 000)	10·0% (−73·9 to 350·9)	271·5 (247·3 to 300·9)	31 000 (29 000 to 35 000)	1·6% (−13·2 to 20·9)	529·4 (347·0 to 710·3)	61 000 (40 000 to 81 000)	5·5% (−40·5 to 79·5)
	Türkiye	405·8 (127·1 to 642·6)	328 000 (101 000 to 516 000)	−2·4% (−71·3 to 276·7)	322·2 (290·0 to 358·6)	264 000 (237 000 to 293 000)	−5·3% (−19·8 to 8·6)	728·0 (435·7 to 969·4)	591 000 (355 000 to 784 000)	−3·7% (−47·7 to 75·0)
	United Arab Emirates	482·4 (148·1 to 748·3)	43 000 (14 000 to 68 000)	−15·0% (−72·7 to 171·8)	341·1 (309·6 to 378·4)	34 000 (31 000 to 38 000)	0·0% (−14·9 to 14·7)	823·5 (491·7 to 1 092·0)	77 000 (49 000 to 102 000)	−9·4% (−49·0 to 68·7)
	Yemen	197·4 (36·9 to 352·4)	71 000 (13 000 to 127 000)	−3·6% (−82·2 to 555·5)	322·0 (269·0 to 366·3)	123 000 (101 000 to 141 000)	−5·3% (−17·9 to 9·5)	519·4 (349·2 to 695·1)	194 000 (131 000 to 260 000)	−4·6% (−39·9 to 55·1)
**South Asia**		**259·7 (190·7 to 329·9)**	**4 708 000 (3 434 000 to 6 020 000)**	**4·9% (−25·0 to 57·7)**	**406·5 (350·2 to 464·2)**	**7 499 000 (6 386 000 to 8 614 000)**	**0·7% (−7·6 to 11·7)**	**666·2 (558·2 to 776·5)**	**12 207 000 (10 168 000 to 14 275 000)**	**2·3% (−11·2 to 18·9)**
South Asia										
	Bangladesh	199·3 (59·0 to 356·3)	325 000 (96 000 to 583 000)	−1·5% (−73·7 to 422·2)	321·3 (262·9 to 382·9)	538 000 (438 000 to 645 000)	−13·5% (−22·7 to −4·6)	520·7 (359·1 to 712·3)	864 000 (595 000 to 1 182 000)	−9·3% (−41·9 to 35·5)
	Bhutan	297·6 (78·9 to 525·4)	2000 (1000 to 4000)	10·5% (−73·2 to 491·2)	473·0 (423·5 to 526·6)	4 000 (3 000 to 4 000)	−8·0% (−15·0 to 0·1)	770·6 (532·4 to 1 012·6)	6000 (4000 to 8000)	−1·6% (−36·8 to 51·0)
	India	256·2 (185·6 to 325·7)	3 550 000 (2 570 000 to 4 559 000)	4·4% (−25·2 to 51·1)	416·3 (357·0 to 476·8)	5 849 000 (4 969 000 to 6 714 000)	−0·3% (−9·2 to 11·6)	672·5 (563·4 to 794·5)	9 399 000 (7 854 000 to 11 179 000)	1·5% (−11·7 to 18·4)
	Nepal	339·5 (68·8 to 609·1)	103 000 (21 000 to 185 000)	1·8% (−78·4 to 464·7)	556·6 (500·2 to 618·0)	171 000 (153 000 to 189 000)	−17·1% (−23·1 to −9·9)	896·1 (619·0 to 1 197·6)	274 000 (189 000 to 365 000)	−10·8% (−42·2 to 38·1)
	Pakistan	308·5 (157·1 to 447·3)	727 000 (365 000 to 1 075 000)	2·5% (−46·3 to 141·9)	365·6 (316·6 to 416·5)	938 000 (806 000 to 1 071 000)	26·6% (12·4 to 48·2)	674·1 (502·5 to 835·5)	1 665 000 (1 242 000 to 2 078 000)	14·3% (−15·7 to 65·2)
**Southeast Asia, east Asia, and Oceania**		**232·2 (168·0 to 298·2)**	**5 041 000 (3 658 000 to 6 516 000)**	**15·7% (−13·6 to 51·6)**	**272·9 (249·2 to 298·7)**	**5 864 000 (5 279 000 to 6 448 000)**	**19·0% (2·4 to 37·4)**	**505·0 (432·5 to 579·7)**	**10 904 000 (9 320 000 to 12 538 000)**	**17·4% (1·2 to 37·1)**
East Asia		216·1 (149·9 to 279·2)	3 220 000 (2 285 000 to 4 176 000)	12·3% (−18·8 to 56·7)	250·7 (226·9 to 275·4)	3 701 000 (3 333 000 to 4 139 000)	13·0% (−4·1 to 32·8)	466·7 (397·1 to 542·9)	6 921 000 (5 825 000 to 8 079 000)	12·7% (−4·3 to 33·8)
	China	214·7 (150·1 to 278·6)	3 086 000 (2 177 000 to 4 021 000)	13·4% (−18·7 to 59·1)	249·6 (225·7 to 274·8)	3 565 000 (3 207 000 to 3 993 000)	13·9% (−3·6 to 34·4)	464·4 (392·5 to 539·1)	6 651 000 (5 573 000 to 7 767 000)	13·7% (−3·6 to 36·2)
	North Korea	177·8 (47·7 to 304·9)	46 000 (12 000 to 79 000)	−31·0% (−82·5 to 203·9)	267·3 (241·6 to 292·4)	69 000 (62 000 to 76 000)	−11·6% (−26·2 to 6·4)	445·1 (312·4 to 583·0)	115 000 (81 000 to 151 000)	−20·5% (−49·9 to 29·9)
	Taiwan (province of China)	334·2 (84·6 to 509·7)	87 000 (22 000 to 132 000)	11·8% (−68·0 to 325·6)	291·0 (266·0 to 318·5)	67 000 (61 000 to 76 000)	−1·2% (−12·3 to 10·7)	625·3 (374·1 to 798·7)	155 000 (90 000 to 199 000)	5·3% (−38·5 to 84·6)
Oceania		248·8 (111·2 to 390·5)	34 000 (14 000 to 54 000)	−2·9% (−57·7 to 120·6)	309·2 (282·0 to 338·5)	44 000 (41 000 to 48 000)	−5·7% (−11·9 to 1·4)	558·0 (418·0 to 710·8)	78 000 (59 000 to 100 000)	−4·5% (−31·9 to 33·9)
	American Samoa	366·2 (92·7 to 594·3)	180 (50 to 290)	5·0% (−70·9 to 288·9)	382·6 (347·1 to 420·8)	190 (170 to 210)	−4·8% (−12·0 to 3·5)	748·8 (479·1 to 979·5)	370 (240 to 480)	−0·2% (−39·4 to 62·9)
	Cook Islands	364·6 (115·3 to 578·1)	70 (20 to 110)	2·6% (−70·9 to 287·0)	345·0 (314·1 to 381·9)	60 (50 to 60)	−4·9% (−13·3 to 4·5)	709·6 (456·5 to 936·7)	120 (80 to 170)	−1·2% (−41·8 to 70·3)
	Federated States of Micronesia	289·1 (62·6 to 488·8)	290 (60 to 500)	−5·5% (−78·5 to 274·7)	334·3 (300·3 to 366·8)	350 (320 to 390)	−7·8% (−15·0 to 0·9)	623·5 (392·6 to 830·7)	650 (410 to 860)	−6·7% (−44·4 to 53·9)
	Fiji	381·3 (111·3 to 598·0)	3000 (1000 to 5000)	7·6% (−66·6 to 379·1)	329·6 (299·3 to 362·0)	3 000 (3 000 to 3 000)	−1·2% (−11·5 to 9·6)	710·9 (434·9 to 939·1)	7000 (4000 to 9000)	3·3% (−38·4 to 81·1)
	Guam	351·4 (123·2 to 567·2)	570 (200 to 920)	4·6% (−65·1 to 280·9)	327·9 (298·1 to 362·2)	500 (450 to 550)	0·0% (−9·2 to 10·2)	679·3 (446·3 to 890·2)	1 000 (1000 to 1000)	2·3% (−36·9 to 69·2)
	Kiribati	317·8 (79·1 to 551·9)	380 (90 to 670)	−10·9% (−81·1 to 282·5)	330·5 (297·4 to 365·1)	420 (370 to 460)	−1·3% (−9·3 to 8·8)	648·3 (408·0 to 892·9)	800 (510 to 1100)	−6·3% (−47·3 to 70·7)
	Marshall Islands	278·6 (67·4 to 492·3)	160 (40 to 270)	2·1% (−79·8 to 393·8)	320·1 (288·0 to 351·6)	190 (170 to 210)	−8·2% (−14·8 to −0·9)	598·7 (376·7 to 818·4)	340 (220 to 470)	−3·7% (−44·3 to 67·7)
	Nauru	376·9 (99·3 to 611·3)	40 (10 to 70)	−13·9% (−77·9 to 309·9)	309·4 (279·6 to 341·3)	40 (30 to 40)	−9·4% (−17·5 to 0·5)	686·3 (411·0 to 928·5)	80 (50 to 100)	−11·9% (−52·2 to 68·2)
	Niue	347·9 (108·6 to 551·5)	6 (2 to 9)	3·6% (−64·7 to 313·9)	354·1 (323·5 to 391·7)	6 (5 to 6)	1·5% (−9·2 to 12·5)	701·9 (459·1 to 910·0)	10 (10 to 20)	2·5% (−34·4 to 70·2)
	Northern Mariana Islands	326·8 (80·8 to 516·1)	160 (40 to 250)	−13·6% (−78·4 to 265·0)	358·4 (323·5 to 395·4)	170 (160 to 190)	−5·4% (−13·0 to 4·5)	685·2 (434·7 to 881·7)	330 (210 to 420)	−9·5% (−46·6 to 52·2)
	Palau	403·1 (115·7 to 631·4)	70 (20 to 110)	−1·2% (−72·5 to 331·9)	325·1 (296·4 to 356·7)	60 (50 to 60)	3·2% (−7·9 to 16·4)	728·2 (430·6 to 955·9)	130 (70 to 170)	0·7% (−45·4 to 89·0)
	Papua New Guinea	227·3 (54·7 to 402·6)	24 000 (6 000 to 42 000)	0·5% (−74·5 to 376·3)	305·6 (278·0 to 336·8)	33 000 (30 000 to 36 000)	−5·1% (−11·4 to 2·5)	532·9 (355·3 to 717·1)	57 000 (38 000 to 76 000)	−2·8% (−38·4 to 61·0)
	Samoa	295·3 (90·6 to 476·0)	610 (190 to 990)	3·3% (−71·8 to 406·8)	328·1 (296·0 to 364·9)	700 (630 to 770)	−9·3% (−17·0 to −0·9)	623·3 (415·3 to 811·7)	1000 (1000 to 2000)	−3·7% (−41·9 to 56·0)
	Solomon Islands	256·7 (61·9 to 458·6)	2 000 (0 to 3 000)	2·8% (−75·7 to 385·8)	325·8 (293·8 to 361·3)	2000 (2000 to 3000)	−9·3% (−15·9 to −1·3)	582·5 (381·0 to 794·1)	4000 (3000 to 5000)	−4·3% (−41·7 to 55·0)
	Tokelau	268·2 (64·5 to 454·5)	4 (1 to 6)	1·8% (−78·3 to 359·3)	319·2 (287·6 to 350·1)	4 (4 to 5)	−3·8% (−13·2 to 6·8)	587·4 (382·0 to 777·7)	8 (5 to 11)	−1·3% (−41·0 to 66·1)
	Tonga	261·6 (79·9 to 422·3)	270 (80 to 450)	7·6% (−72·3 to 341·5)	253·8 (227·3 to 284·6)	270 (240 to 300)	−3·9% (−15·7 to 10·2)	515·4 (327·3 to 675·8)	540 (350 to 710)	1·6% (−41·4 to 68·8)
	Tuvalu	272·2 (74·7 to 438·5)	30 (10 to 50)	1·0% (−73·4 to 279·4)	263·8 (237·1 to 294·1)	30 (30 to 40)	20·4% (3·8 to 39·2)	536·0 (340·6 to 709·6)	70 (40 to 90)	9·7% (−39·6 to 98·4)
	Vanuatu	254·8 (56·8 to 433·2)	780 (170 to 1350)	2·5% (−75·6 to 384·7)	327·7 (296·6 to 357·2)	1000 (1000 to 1000)	−3·9% (−11·8 to 6·5)	582·5 (377·7 to 774·7)	2000 (1000 to 2000)	−1·2% (−39·2 to 68·1)
Southeast Asia		263·0 (186·3 to 349·7)	1 787 000 (1 253 000 to 2 367 000)	19·3% (−12·8 to 64·9)	304·7 (278·2 to 334·2)	2 118 000 (1 921 000 to 2 323 000)	27·3% (12·7 to 43·2)	567·6 (477·8 to 663·7)	3 905 000 (3 287 000 to 4 559 000)	23·5% (5·3 to 46·5)
	Cambodia	233·4 (73·5 to 429·0)	39 000 (12 000 to 72 000)	8·8% (−70·0 to 491·5)	333·7 (298·5 to 369·4)	58 000 (52 000 to 64 000)	−0·2% (−10·1 to 12·4)	567·1 (405·1 to 770·0)	97 000 (70 000 to 131 000)	3·3% (−33·6 to 68·0)
	Indonesia	219·0 (147·8 to 294·1)	584 000 (393 000 to 785 000)	17·6% (−19·2 to 76·1)	241·0 (214·7 to 268·9)	677 000 (603 000 to 756 000)	100·7% (46·8 to 164·5)	460·0 (380·9 to 549·3)	1 261 000 (1 044 000 to 1 503 000)	50·2% (18·8 to 94·5)
	Laos	252·3 (60·5 to 427·9)	18 000 (4 000 to 31 000)	12·6% (−75·3 to 677·6)	323·7 (290·5 to 360·9)	24 000 (22 000 to 27 000)	7·4% (−5·9 to 22·7)	576·0 (388·3 to 767·3)	42 000 (29 000 to 56 000)	9·6% (−36·4 to 88·4)
	Malaysia	353·4 (94·8 to 559·1)	110 000 (30 000 to 174 000)	13·5% (−67·4 to 279·2)	358·4 (323·9 to 390·0)	115 000 (104 000 to 126 000)	−4·5% (−15·5 to 8·2)	711·8 (447·9 to 925·2)	225 000 (143 000 to 291 000)	3·6% (−37·8 to 69·3)
	Maldives	376·5 (120·1 to 631·7)	2000 (1000 to 3000)	−6·2% (−71·1 to 382·9)	355·4 (323·7 to 393·1)	2000 (2000 to 2000)	−6·4% (−20·3 to 10·0)	731·9 (472·9 to 987·5)	4000 (2000 to 5000)	−6·3% (−44·2 to 69·9)
	Mauritius	630·3 (207·5 to 981·0)	8000 (3 000 to 13 000)	31·4% (−62·1 to 543·2)	547·5 (500·9 to 597·8)	7000 (6000 to 7000)	17·3% (2·8 to 34·8)	1 177·7 (735·6 to 1 536·9)	15 000 (9000 to 20 000)	24·5% (−30·3 to 117·6)
	Myanmar	292·2 (84·4 to 518·6)	164 000 (47 000 to 291 000)	15·9% (−70·5 to 482·6)	305·9 (274·7 to 340·8)	173 000 (155 000 to 193 000)	22·7% (9·6 to 41·2)	598·1 (386·9 to 829·2)	337 000 (219 000 to 466 000)	19·3% (−31·1 to 112·8)
	Philippines	243·5 (175·2 to 319·5)	271 000 (193 000 to 360 000)	3·8% (−17·0 to 30·6)	325·7 (289·8 to 362·7)	376 000 (331 000 to 419 000)	31·5% (10·9 to 56·1)	569·2 (478·9 to 663·0)	646 000 (540 000 to 756 000)	18·0% (2·1 to 35·2)
	Sri Lanka	476·5 (117·2 to 777·7)	108 000 (26 000 to 176 000)	2·4% (−77·5 to 320·0)	511·9 (470·2 to 557·8)	112 000 (103 000 to 122 000)	−13·8% (−20·8 to −6·2)	988·4 (618·0 to 1 303·7)	220 000 (136 000 to 291 000)	−6·7% (−44·1 to 52·6)
	Seychelles	331·8 (94·3 to 560·3)	340 (100 to 580)	11·7% (−65·9 to 292·1)	353·2 (321·5 to 388·7)	360 (330 to 400)	−2·8% (−12·6 to 7·4)	685·0 (447·6 to 919·4)	700 (460 to 950)	3·7% (−35·9 to 65·0)
	Thailand	344·2 (111·1 to 559·4)	236 000 (74 000 to 382 000)	49·3% (−53·9 to 565·2)	405·3 (368·8 to 438·1)	257 000 (235 000 to 279 000)	15·9% (2·7 to 30·3)	749·5 (509·3 to 973·5)	492 000 (329 000 to 644 000)	29·2% (−15·7 to 107·0)
	Timor-Leste	251·7 (69·0 to 428·9)	4000 (1000 to 6000)	14·7% (−69·2 to 561·7)	354·1 (311·4 to 399·6)	5000 (5000 to 6000)	−0·7% (−10·5 to 11·0)	605·8 (412·9 to 805·9)	9000 (6000 to 12 000)	5·2% (−33·9 to 65·6)
	Viet Nam	246·7 (56·2 to 409·7)	241 000 (55 000 to 398 000)	37·9% (−69·7 to 544·2)	311·3 (280·5 to 340·3)	310 000 (278 000 to 340 000)	1·9% (−9·0 to 13·0)	558·0 (371·2 to 733·8)	551 000 (367 000 to 724 000)	15·2% (−29·4 to 80·0)
**Sub-Saharan Africa**		**386·3 (286·7 to 491·3)**	**4 264 000 (3 099 000 to 5 546 000)**	**4·1% (−17·0 to 37·2)**	**417·5 (381·6 to 460·0)**	**4 662 000 (4 247 000 to 5 113 000)**	**34·0% (21·6 to 48·2)**	**803·8 (693·4 to 922·4)**	**8 926 000 (7 605 000 to 10 225 000)**	**17·8% (2·7 to 36·1)**
Central sub-Saharan Africa		394·3 (177·8 to 625·8)	525 000 (235 000 to 847 000)	−4·7% (−59·2 to 153·9)	479·0 (429·2 to 527·1)	596 000 (541 000 to 650 000)	11·6% (3·0 to 22·0)	873·3 (648·2 to 1 118·6)	1 121 000 (818 000 to 1 456 000)	3·6% (−27·5 to 53·7)
	Angola	542·0 (142·7 to 917·0)	169 000 (45 000 to 283 000)	11·2% (−75·6 to 480·1)	560·0 (483·1 to 634·9)	157 000 (140 000 to 174 000)	15·3% (7·0 to 25·9)	1 102·0 (689·5 to 1 484·9)	326 000 (201 000 to 442 000)	13·2% (−38·3 to 102·1)
	Central African Republic	325·7 (65·0 to 600·6)	18 000 (4000 to 33 000)	−10·7% (−80·2 to 419·3)	421·9 (373·1 to 466·0)	21 000 (18 000 to 23 000)	−5·7% (−12·0 to 2·0)	747·6 (482·7 to 1 032·3)	38 000 (24 000 to 54 000)	−7·9% (−47·9 to 64·3)
	Congo (Brazzaville)	515·4 (144·1 to 887·6)	26 000 (7000 to 45 000)	−1·9% (−74·7 to 389·7)	582·0 (512·7 to 649·8)	29 000 (26 000 to 32 000)	9·0% (−0·6 to 20·3)	1 097·4 (719·1 to 1 477·0)	56 000 (36 000 to 75 000)	3·6% (−41·5 to 82·3)
	Democratic Republic of the Congo	327·0 (73·1 to 584·1)	290 000 (64 000 to 529 000)	−14·4% (−81·5 to 382·3)	443·0 (400·1 to 489·4)	370 000 (338 000 to 404 000)	11·0% (0·3 to 23·4)	770·0 (500·9 to 1 042·3)	660 000 (428 000 to 903 000)	−1·4% (−41·7 to 77·3)
	Equatorial Guinea	706·0 (172·0 to 1 150·4)	10 000 (2000 to 17 000)	75·4% (−58·7 to 1 085·1)	577·8 (498·3 to 663·7)	8000 (7000 to 9000)	25·8% (14·5 to 39·6)	1 283·8 (750·4 to 1 754·2)	18 000 (10 000 to 25 000)	49·0% (−18·6 to 185·3)
	Gabon	688·2 (146·3 to 1 158·0)	12 000 (3 000 to 20 000)	1·9% (−73·6 to 397·7)	671·9 (590·2 to 760·2)	12 000 (10 000 to 13 000)	1·9% (−7·3 to 12·6)	1 360·0 (822·9 to 1 833·1)	23 000 (14 000 to 32 000)	1·9% (−43·7 to 89·6)
Eastern sub-Saharan Africa		355·2 (242·0 to 477·8)	1 507 000 (1 015 000 to 2 022 000)	2·7% (−25·3 to 57·7)	428·7 (390·3 to 474·7)	1 854 000 (1 669 000 to 2 040 000)	49·3% (32·8 to 67·6)	783·8 (658·0 to 921·0)	3 361 000 (2 792 000 to 3 932 000)	23·8% (3·1 to 53·5)
	Burundi	306·8 (57·7 to 602·2)	41 000 (8000 to 81 000)	−23·1% (−84·5 to 418·3)	423·7 (381·3 to 471·2)	55 000 (50 000 to 62 000)	26·5% (11·0 to 45·9)	730·5 (469·2 to 1 028·7)	96 000 (62 000 to 136 000)	−0·5% (−43·8 to 101·0)
	Comoros	378·8 (108·6 to 661·6)	3000 (1000 to 5000)	−4·8% (−74·5 to 471·1)	453·3 (409·2 to 500·7)	4000 (3000 to 4000)	70·3% (42·1 to 106·2)	832·1 (566·0 to 1 130·7)	6000 (4000 to 9000)	25·3% (−30·8 to 168·3)
	Djibouti	429·9 (113·8 to 738·0)	5000 (1000 to 9000)	4·4% (−74·4 to 459·3)	361·6 (315·4 to 409·0)	5 000 (4 000 to 6 000)	100·0% (61·2 to 144·1)	791·5 (465·3 to 1 116·7)	10 000 (6 000 to 14 000)	33·6% (−33·7 to 213·1)
	Eritrea	380·1 (98·2 to 676·6)	25 000 (6000 to 45 000)	8·6% (−74·9 to 601·0)	452·1 (407·3 to 507·9)	31 000 (28 000 to 34 000)	43·8% (28·4 to 65·1)	832·2 (539·2 to 1 131·5)	56 000 (37 000 to 76 000)	25·3% (−30·5 to 149·7)
	Ethiopia	257·5 (139·1 to 372·4)	277 000 (148 000 to 406 000)	0·2% (−47·4 to 151·5)	413·2 (368·7 to 459·3)	455 000 (405 000 to 509 000)	66·0% (41·2 to 97·8)	670·7 (538·4 to 804·8)	733 000 (575 000 to 886 000)	32·6% (−2·8 to 94·7)
	Kenya	411·9 (308·0 to 527·6)	202 000 (147 000 to 267 000)	3·0% (−22·4 to 45·3)	503·7 (452·4 to 563·1)	253 000 (225 000 to 281 000)	30·8% (12·3 to 51·9)	915·6 (786·0 to 1 060·7)	455 000 (385 000 to 533 000)	16·6% (−0·1 to 39·9)
	Madagascar	337·3 (88·4 to 620·0)	97 000 (25 000 to 180 000)	−12·2% (−80·0 to 278·1)	450·7 (408·2 to 499·3)	131 000 (118 000 to 145 000)	43·2% (25·7 to 65·2)	788·0 (531·1 to 1 078·8)	228 000 (154 000 to 313 000)	12·8% (−36·3 to 108·4)
	Malawi	338·2 (75·6 to 610·6)	68 000 (15 000 to 124 000)	−4·4% (−76·2 to 384·8)	426·7 (386·1 to 475·2)	84 000 (76 000 to 94 000)	42·4% (25·0 to 64·7)	764·9 (506·5 to 1 043·4)	152 000 (102 000 to 210 000)	17·1% (−31·6 to 127·5)
	Mozambique	409·6 (77·4 to 763·8)	127 000 (23 000 to 236 000)	28·0% (−73·4 to 656·0)	457·1 (404·9 to 520·1)	143 000 (128 000 to 160 000)	43·8% (30·5 to 60·3)	866·8 (530·2 to 1 227·2)	269 000 (167 000 to 378 000)	35·9% (−28·0 to 148·7)
	Rwanda	360·5 (77·4 to 634·1)	48 000 (10 000 to 85 000)	−11·1% (−81·4 to 351·0)	414·1 (368·2 to 466·5)	54 000 (48 000 to 61 000)	30·6% (14·2 to 52·2)	774·6 (486·1 to 1 043·4)	102 000 (63 000 to 137 000)	7·2% (−42·5 to 109·3)
	Somalia	211·5 (34·2 to 459·8)	46 000 (7000 to 102 000)	−11·1% (−84·3 to 560·1)	282·1 (243·8 to 319·4)	70 000 (61 000 to 79 000)	97·4% (68·1 to 131·5)	493·6 (306·4 to 744·8)	116 000 (75 000 to 176 000)	29·6% (−37·4 to 205·8)
	South Sudan	334·8 (76·4 to 623·4)	32 000 (8000 to 61 000)	−18·9% (−84·4 to 276·2)	274·7 (242·8 to 311·8)	29 000 (25 000 to 32 000)	54·1% (36·5 to 75·6)	609·5 (345·2 to 893·8)	61 000 (35 000 to 89 000)	3·1% (−53·9 to 127·8)
	Tanzania	425·8 (103·0 to 749·0)	251 000 (59 000 to 445 000)	12·2% (−68·0 to 523·8)	410·8 (367·5 to 461·4)	251 000 (223 000 to 279 000)	64·1% (42·4 to 86·0)	836·6 (525·0 to 1 163·3)	502 000 (318 000 to 693 000)	32·9% (−28·2 to 181·0)
	Uganda	404·7 (85·8 to 722·6)	178 000 (39 000 to 320 000)	10·7% (−75·8 to 578·0)	462·5 (416·4 to 520·9)	202 000 (180 000 to 225 000)	48·6% (30·8 to 69·3)	867·2 (552·5 to 1 191·2)	380 000 (242 000 to 527 000)	28·1% (−31·7 to 150·2)
	Zambia	547·3 (147·9 to 944·1)	106 000 (28 000 to 184 000)	6·3% (−69·9 to 376·4)	443·3 (390·9 to 501·9)	86 000 (77 000 to 96 000)	26·0% (11·9 to 42·4)	990·6 (574·7 to 1 401·3)	192 000 (111 000 to 271 000)	14·3% (−40·1 to 128·2)
Southern sub-Saharan Africa		403·7 (271·2 to 545·3)	316 000 (207 000 to 431 000)	−2·5% (−33·0 to 47·1)	535·3 (479·8 to 593·4)	422 000 (377 000 to 467 000)	18·6% (3·9 to 36·2)	939·0 (790·6 to 1 101·4)	738 000 (625 000 to 867 000)	8·5% (−8·2 to 29·0)
	Botswana	513·1 (142·2 to 844·8)	12 000 (3000 to 20 000)	39·5% (−58·8 to 434·0)	678·1 (607·3 to 757·2)	16 000 (14 000 to 18 000)	22·1% (12·1 to 36·0)	1 191·1 (799·4 to 1 549·3)	28 000 (19 000 to 36 000)	29·1% (−17·7 to 97·0)
	Eswatini	449·0 (107·6 to 751·3)	5000 (1000 to 8000)	26·0% (−74·1 to 591·4)	492·9 (422·3 to 561·4)	5 000 (4 000 to 6 000)	18·2% (5·5 to 34·0)	941·9 (604·9 to 1 276·3)	10 000 (7 000 to 14 000)	21·8% (−31·3 to 118·3)
	Lesotho	360·1 (79·7 to 602·7)	7000 (1000 to 11 000)	45·4% (−70·6 to 537·8)	437·4 (378·7 to 494·7)	8 000 (7 000 to 9 000)	24·8% (8·6 to 40·7)	797·5 (519·1 to 1 055·8)	14 000 (9 000 to 19 000)	33·4% (−20·9 to 119·3)
	Namibia	405·5 (123·2 to 688·0)	9000 (3000 to 16 000)	15·4% (−68·7 to 384·6)	581·7 (525·0 to 645·0)	14 000 (12 000 to 15 000)	16·1% (4·6 to 30·4)	987·1 (684·5 to 1 291·7)	23 000 (16 000 to 30 000)	15·8% (−26·7 to 80·2)
	South Africa	407·3 (266·3 to 544·6)	229 000 (148 000 to 306 000)	−6·8% (−39·1 to 45·2)	532·4 (476·0 to 589·6)	300 000 (267 000 to 334 000)	21·9% (5·1 to 44·0)	939·6 (771·1 to 1 116·4)	528 000 (433 000 to 629 000)	7·6% (−12·4 to 33·2)
	Zimbabwe	380·2 (89·8 to 677·6)	55 000 (13 000 to 98 000)	1·2% (−76·0 to 409·1)	556·4 (495·1 to 616·3)	79 000 (71 000 to 88 000)	9·4% (−1·9 to 21·8)	936·6 (648·9 to 1 240·2)	134 000 (94 000 to 178 000)	5·9% (−32·1 to 73·5)
Western sub-Saharan Africa		404·5 (293·9 to 526·1)	1 916 000 (1 369 000 to 2 514 000)	9·6% (−15·1 to 52·1)	364·1 (331·7 to 402·8)	1 790 000 (1 623 000 to 1 964 000)	38·4% (27·4 to 50·8)	768·6 (655·9 to 889·8)	3 706 000 (3 135 000 to 4 294 000)	21·6% (3·8 to 47·6)
	Benin	391·2 (96·6 to 719·4)	52 000 (13 000 to 96 000)	2·2% (−78·0 to 556·0)	494·3 (441·4 to 549·5)	60 000 (54 000 to 67 000)	14·6% (5·5 to 25·0)	885·6 (581·5 to 1 226·2)	112 000 (71 000 to 159 000)	8·8% (−38·3 to 95·1)
	Burkina Faso	362·1 (66·6 to 692·2)	82 000 (15 000 to 157 000)	9·2% (−78·8 to 445·4)	264·2 (238·0 to 298·2)	61 000 (55 000 to 69 000)	11·9% (1·7 to 24·1)	626·4 (335·3 to 961·6)	143 000 (77 000 to 219 000)	10·3% (−48·2 to 140·5)
	Cameroon	402·8 (99·8 to 694·6)	124 000 (31 000 to 214 000)	4·8% (−74·0 to 387·9)	399·5 (357·6 to 444·4)	118 000 (107 000 to 130 000)	9·8% (1·2 to 20·6)	802·3 (506·7 to 1 091·7)	242 000 (151 000 to 332 000)	7·2% (−41·8 to 96·5)
	Cabo Verde	500·8 (125·8 to 858·8)	3000 (1000 to 5000)	29·1% (−69·1 to 558·0)	677·2 (610·4 to 746·0)	4000 (3000 to 4000)	12·8% (1·9 to 25·2)	1 178·0 (797·2 to 1 537·5)	6000 (4000 to 9000)	19·2% (−25·8 to 100·7)
	Chad	342·1 (60·3 to 649·1)	61 000 (11 000 to 116 000)	21·2% (−71·8 to 514·6)	246·7 (220·8 to 278·8)	45 000 (41 000 to 51 000)	30·5% (18·4 to 43·8)	588·7 (306·3 to 894·5)	106 000 (56 000 to 160 000)	24·9% (−40·8 to 172·7)
	Côte d'Ivoire	446·6 (109·5 to 797·9)	119 000 (29 000 to 214 000)	9·8% (−74·9 to 454·0)	516·5 (453·4 to 579·3)	133 000 (118 000 to 147 000)	20·0% (10·0 to 32·5)	963·1 (609·7 to 1 324·0)	252 000 (160 000 to 351 000)	15·1% (−35·5 to 100·2)
	The Gambia	334·6 (77·4 to 578·0)	8000 (2000 to 14 000)	15·6% (−76·0 to 505·4)	467·6 (415·1 to 519·3)	12 000 (11 000 to 14 000)	47·7% (23·1 to 76·8)	802·2 (536·2 to 1 071·6)	20 000 (14 000 to 27 000)	32·4% (−22·7 to 141·5)
	Ghana	387·6 (104·4 to 634·9)	129 000 (35 000 to 214 000)	27·5% (−67·7 to 520·9)	465·1 (416·1 to 516·1)	152 000 (137 000 to 168 000)	25·8% (14·9 to 37·9)	852·7 (575·6 to 1 122·1)	281 000 (187 000 to 371 000)	26·6% (−26·4 to 116·8)
	Guinea	374·1 (81·7 to 702·2)	50 000 (11 000 to 95 000)	8·8% (−74·8 to 515·4)	268·1 (240·3 to 298·3)	40 000 (36 000 to 44 000)	55·0% (39·7 to 72·4)	642·2 (346·4 to 965·0)	90 000 (50 000 to 135 000)	24·3% (−40·7 to 206·5)
	Guinea-Bissau	385·9 (71·8 to 712·7)	8000 (1000 to 14 000)	−4·3% (−82·1 to 496·2)	385·3 (347·4 to 429·6)	8000 (8000 to 9000)	29·2% (16·0 to 46·6)	771·2 (461·9 to 1 104·1)	16 000 (10 000 to 23 000)	9·9% (−46·4 to 125·7)
	Liberia	365·3 (72·6 to 657·7)	19 000 (4 000 to 35 000)	−12·3% (−83·9 to 398·1)	533·0 (481·5 to 579·2)	27 000 (25 000 to 30 000)	22·7% (10·3 to 38·9)	898·3 (618·5 to 1 209·1)	47 000 (32 000 to 63 000)	5·6% (−37·5 to 90·5)
	Mali	288·3 (64·0 to 547·2)	68 000 (15 000 to 132 000)	36·7% (−67·2 to 687·9)	255·4 (228·0 to 285·9)	70 000 (62 000 to 79 000)	62·2% (44·8 to 81·8)	543·6 (316·0 to 804·3)	138 000 (85 000 to 200 000)	47·6% (−29·9 to 212·6)
	Mauritania	384·2 (106·1 to 649·1)	16 000 (5 000 to 28 000)	0·4% (−74·2 to 367·6)	536·4 (456·7 to 605·4)	26 000 (21 000 to 29 000)	24·6% (4·6 to 50·6)	920·6 (623·5 to 1 218·3)	42 000 (29 000 to 56 000)	13·2% (−29·2 to 89·9)
	Niger	264·6 (48·4 to 540·1)	67 000 (12 000 to 136 000)	−6·6% (−81·7 to 451·7)	254·9 (228·0 to 284·6)	74 000 (66 000 to 82 000)	66·4% (48·1 to 85·8)	519·5 (305·0 to 784·8)	142 000 (87 000 to 210 000)	19·0% (−43·2 to 185·4)
	Nigeria	443·4 (326·7 to 580·9)	984 000 (707 000 to 1 293 000)	10·7% (−15·8 to 57·3)	346·5 (313·5 to 386·3)	821 000 (738 000 to 908 000)	52·2% (36·6 to 69·0)	789·9 (673·0 to 928·3)	1 805 000 (1 518 000 to 2 101 000)	25·8% (4·6 to 55·4)
	São Tomé and Príncipe	439·0 (133·7 to 723·0)	930 (280 to 1550)	28·3% (−68·3 to 547·7)	614·5 (557·4 to 672·7)	1000 (1000 to 1000)	36·0% (24·9 to 50·8)	1 053·6 (735·6 to 1 365·5)	2000 (2000 to 3000)	32·7% (−17·4 to 120·1)
	Senegal	400·8 (89·7 to 717·9)	62 000 (14 000 to 113 000)	13·4% (−77·5 to 701·7)	411·2 (366·4 to 458·0)	72 000 (64 000 to 80 000)	92·7% (66·4 to 121·2)	812·0 (494·0 to 1 122·0)	134 000 (84 000 to 183 000)	43·2% (−29·6 to 218·4)
	Sierra Leone	346·4 (73·4 to 636·3)	31 000 (6 000 to 57 000)	2·6% (−79·0 to 462·9)	249·7 (222·8 to 279·8)	24 000 (21 000 to 27 000)	49·2% (33·3 to 67·0)	596·1 (322·7 to 892·3)	55 000 (31 000 to 81 000)	18·0% (−43·8 to 173·2)
	Togo	399·6 (86·9 to 733·2)	33 000 (7 000 to 61 000)	−21·9% (−82·4 to 282·0)	525·9 (469·4 to 580·7)	41 000 (37 000 to 45 000)	−1·1% (−10·0 to 10·9)	925·4 (611·3 to 1 263·1)	74 000 (48 000 to 101 000)	−11·3% (−48·4 to 57·4)

Age-standardised rates in the main text of this Article were rounded to the nearest integer. GBD=Global Burden of Diseases, Injuries, and Risk Factors Study.

* Counts in millions and hundreds of thousands were rounded to the nearest thousand; counts less than one thousand were rounded to the nearest ten; counts less than one hundred were presented in exact numbers.

In 2021, the age-standardised prevalence of epilepsy from idiopathic and secondary epilepsy combined ranged from 445 per 100 000 (95% UI 312–583) in North Korea to 1374 (945–1773) in Trinidad and Tobago (table 1). By World Bank country income level, the highest age-standardised prevalence of epilepsy from idiopathic and secondary epilepsy combined (appendix 1 p 50) was observed in low-income countries (LICs; 672 per 100 000 [563–789]), followed by lower-middle-income countries (LMICs; 665 per 100 000 [570–765]); high-income countries (HICs; 662 per 100 000 [544–779]); and upper-middle-income countries (UMICs; 626 per 100 000 [539–718]). There were no significant differences in the age-standardised prevalence of idiopathic and secondary epilepsy between GBD super-regions, except for South Asia, where the prevalence of secondary epilepsy of 407 per 100 000 (350–464) was over 1·5 times greater than the prevalence of idiopathic epilepsy (260 [191–330]).

Over the 1990–2021 period (table 1), there was a substantial increase in age-standardised prevalence of secondary and combined epilepsy (14·5% [95% UI 5·3 to 25·5] and 10·8% [1·1 to 21·3]), but a small increase in the age-standardised prevalence of idiopathic epilepsy (6·9% [–9·7 to 25·5]). Over the past three decades (table 1), the largest increase in combined epilepsy prevalence was observed in Indonesia (50·2% [18·8 to 94·5]), where the prevalence of combined epilepsy remains one of the lowest in the world since 1990, while the largest decrease was observed in Russia (12·8% [2·5 to 21·6]). From 1990 to 2021, no country showed substantial increase in the age-standardised prevalence of idiopathic epilepsy, and only Russia had a substantial decrease in the age-standardised prevalence of idiopathic epilepsy (15·2% [1·9 to 30·1]).

**Figure 1:**
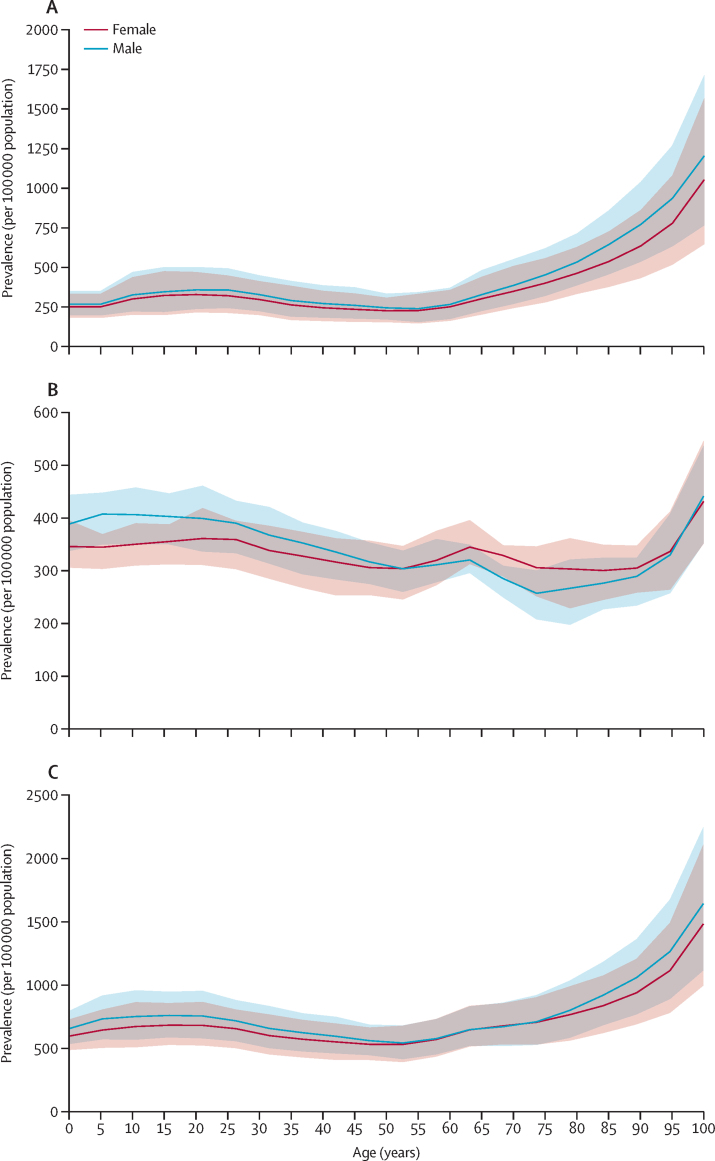
Global age-specific prevalence of idiopathic epilepsy, secondary epilepsy, and combined epilepsy from idiopathic and secondary epilepsy, by age and sex, 2021 (A) Global prevalence of idiopathic epilepsy. (B) Global prevalence of secondary epilepsy. (C) Global prevalence of combined epilepsy. Shadowed areas represent 95% UIs.

There was no substantial sex difference in the prevalence of epilepsy from idiopathic and secondary epilepsy combined (males: 685 per 100 000 [95% UI 593–778]; females: 631 [548–721]) and idiopathic epilepsy (males: 322 [247–405]; females 293 [223–373]; figure 1). The prevalence of idiopathic epilepsy increased from birth (174 per 100 000 [109–276]; age 0–6 days) to age 19 years (343 [227–487]; age 15–19 years), then decreased to age 50 (233 [150–339]; age 50–54 years), and then increased with age steeply after age 55 years, with no substantial sex differences in the age pattern. Although age and sex prevalence patterns were largely similar for idiopathic and secondary epilepsy, secondary epilepsy prevalence showed consistently greater values than idiopathic epilepsy up to age 65 years, after which the age-specific prevalence of idiopathic epilepsy was substantially greater than that for secondary epilepsy (figure 1; table 1).

**Figure 2:**
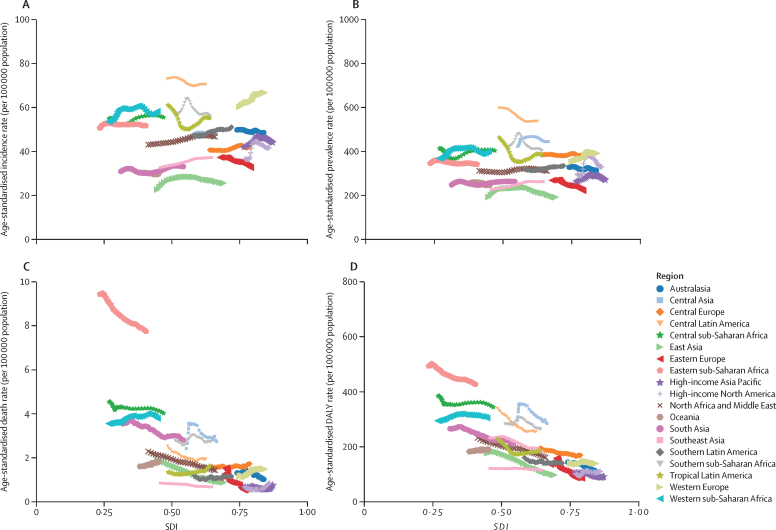
Age-standardised incidence, prevalence, death, and DALY rates for idiopathic epilepsy per 100 000 people in 21 GBD regions by SDI, both sexes, 1990–2021 (A) Age-standardised incidence rates for epilepsy. (B) Age-standardised prevalence rates for epilepsy. (C) Age-standardised death rates for epilepsy. (D) Age-standardised DALY rates for epilepsy. Age-standardised DALY rates are plotted for 21 GBD regions between 1990 and 2021 against their SDI values. Points from left to right represent the values from 1990 to 2021. DALYs=disability-adjusted life-years. GBD=Global Burden of Diseases, Injuries, and Risk Factors Study. SDI=Socio-demographic Index.

Globally, in 2021, there were approximately 3·3 million (95% UI 2·4–4·1) new cases of active idiopathic epilepsy (age-standardised incidence rate 42·8 per 100 000 [31·2–53·7]), 140 000 deaths (117 000–153 000; age-standardised death rate 1·7 [1·5–1·9]), and 13·9 million DALYs (10·7–17·6; age-standardised DALYs rate 177·8 [137·7–225·9]) due to active idiopathic epilepsy (table 2; appendix 1 pp 25–50). Among global deaths and DALYs from idiopathic and secondary epilepsy combined, idiopathic epilepsy accounted for 0·21% (0·17–0·22) of deaths and 0·48% (0·38–0·59) of DALYs, ranking it as the 72nd most common cause of death and 44th most common cause of death and disability combined in the world. Over the past three decades, there was a substantial reduction in the age-standardised death and DALY rates in both males and females (table 2; figure 2; appendix 1 pp 50,52). There were no substantial sex differences in the incidence and DALY rates of idiopathic epilepsy (appendix 1 pp 50,52) in 2021, but death rates in males (2·1 [1·8–2·4]) were substantially greater than in females (1·4 [1·0–1·5]). The global age-standardised prevalence in 2021 was not substantially different between males and females: 322 per 100 000 (247–405) for males and 293 per 100 000 (223–373) for females. The prevalence of idiopathic epilepsy has not changed substantially for either males (302 per 100 000 [227–379]) or females (274 per 100 000 [205–347]) since 1990. The highest prevalence of idiopathic epilepsy in 2021 was in Ecuador, with an age-standardised prevalence of 711 per 100 000 (226–1141). North Korea had the lowest age-standardised prevalence of 178 per 100 000 (48–305).

**Table 2:** Incidence, prevalence, deaths, and DALYs for idiopathic epilepsy in 2021, and percentage change in age-standardised rates by location, and by World Bank country income level and SDI level

		**Incidence**		**Prevalence**		**Deaths**		**DALYs**	
		Counts	Percentage change in age-standardised rates, 1990–2021	Counts	Percentage change in age-standardised rates, 1990–2021	Counts	Percentage change in age-standardised rates, 1990–2021	Counts	Percentage change in age-standardised rates, 1990–2021
Global		3 273 000 (2 404 000 to 4 125 000)	12·3% (−4·8 to 32·6)	24 221 000 (18 477 000 to 30 678 000)	6·9% (−9·7 to 25·5)	140 000 (117 000 to 153 000)	−15·8% (−22·8 to −8·8)	13 878 000 (10 733 000 to 17 620 000)	−14·5% (−24·2 to −4·2)
Countries categorised by the World Bank income level									
	High-income countries	585 000 (398 000 to 778 000)	10·3% (−10·5 to 29·0)	4 750 000 (3 203 000 to 6 234 000)	9·5% (−10·0 to 27·1)	21 000 (19 000 to 23 000)	13·9% (3·4 to 20·3)	1 675 000 (1 155 000 to 2 427 000)	−4·7% (−18·5 to 9·8)
	Upper-middle-income countries	936 000 (669 000 to 1 200 000)	16·6% (−5·6 to 43·6)	7 540 000 (5 502 000 to 9 564 000)	7·9% (−13·4 to 33·0)	29 000 (25 000 to 32 000)	−41·1% (−47·9 to −34·2)	3 485 000 (2 480 000 to 4 648 000)	−30·3% (−42·1 to −15·8)
	Lower-middle-income countries	1 383 000 (995 000 to 1 762 000)	13·2% (−10·8 to 50·0)	9 768 000 (7 446 000 to 12 218 000)	8·5% (−14·9 to 44·0)	65 000 (50 000 to 73 000)	−21·5% (−31·5 to −10·7)	6 473 000 (5 066 000 to 8 074 000)	−17·3% (−28·7 to −2·2)
	Low-income countries	365 000 (241 000 to 507 000)	5·2% (−22·8 to 49·0)	2 141 000 (1 434 000 to 2 900 000)	−2·4% (−28·1 to 40·9)	25 000 (20 000 to 30 000)	−15·6% (−26·7 to −1·4)	2 231 000 (1 736 000 to 2 840 000)	−12·7% (−26·3 to 3·4)
Countries categorised by SDI									
	Low SDI regions	566 000 (371 000 to 776 000)	6·5% (−16·7 to 49·5)	3 443 000 (2 343 000 to 4 625 000)	−0·2% (−22·5 to 40·4)	37 000 (30 000 to 44 000)	−18·4% (−28·1 to −7·5)	3 320 000 (2 620 000 to 4 187 000)	−16·0% (−27·6 to −2·0)
	Low-middle SDI regions	799 000 (581 000 to 1 033 000)	13·3% (−15·5 to 57·4)	5 744 000 (4 245 000 to 7 424 000)	8·6% (−18·6 to 50·7)	43 000 (33 000 to 48 000)	−18·5% (−29·7 to −6·1)	4 116 000 (3 208 000 to 5 234 000)	−16·0% (−29·5 to 3·4)
	Middle SDI regions	965 000 (694 000 to 1 234 000)	14·8% (−8·0 to 45·1)	7 331 000 (5 384 000 to 9 313 000)	8·6% (−13·3 to 37·8)	29 000 (25 000 to 32 000)	−37·1% (−42·4 to −31·4)	3 519 000 (2 606 000 to 4 601 000)	−26·7% (−39·0 to −12·6)
	High-middle SDI regions	426 000 (289 000 to 568 000)	10·9% (−12·4 to 36·6)	3 488 000 (2 452 000 to 4 628 000)	1·6% (−20·3 to 24·3)	14 000 (12 000 to 15 000)	−37·7% (−44·7 to −31·0)	1 444 000 (1 022 000 to 2 022 000)	−33·1% (−45·4 to −17·5)
	High SDI regions	514 000 (342 000 to 685 000)	10·8% (−11·6 to 32·1)	4 193 000 (2 842 000 to 5 536 000)	10·5% (−11·6 to 31·3)	17 000 (16 000 to 19 000)	7·5% (−1·4 to 13·3)	1 466 000 (992 000 to 2 150 000)	−5·5% (−20·2 to 10·2)

DALYs=disability-adjusted life-years. SDI=Socio-demographic Index.

**Figure 3:**
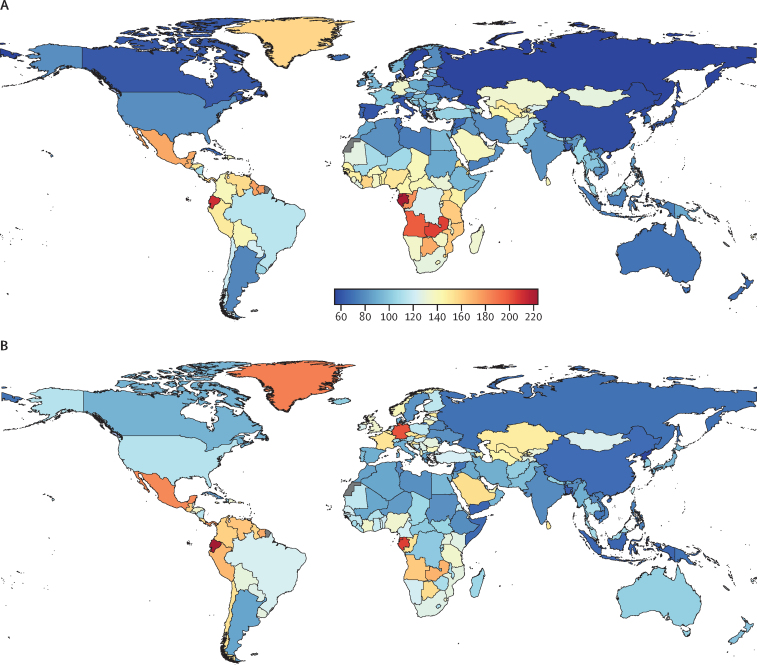
Age-standardised years lived with disability and prevalence of idiopathic epilepsy per 100 000 people, both sexes, 2021 (A) Age-standardised years lived with disability per 100 000 people. (B) Prevalence of idiopathic epilepsy per 100 000 people.

Geographical variations in the age-standardised YLD rates were four-fold, with the highest estimates (figure 3B) in sub-Saharan Africa (particularly in Gabon [470 per 100 000; 220–815]) and central and Latin America (particularly in Guyana [400 per 100 000; 195–654]), and lowest in western Europe (particularly in Italy [105 per 100 000; 54–187]). Lower age-standardised YLD rates were observed in the regions of high-income Asia Pacific, east Asia, Australasia, and eastern Europe. Similar to the geographical differences in age-standardised prevalence and YLDs of epilepsy from idiopathic and secondary epilepsy combined, the age-standardised prevalence of idiopathic epilepsy showed four-fold geographical variations (figure 3A; appendix 1 pp 25–36), with the highest rates in some sub-Saharan African countries (Gabon: 688 per 100 000 [146–1158]; Angola: 542 per 100 000 [143–917]; and Zambia: 547 per 100 000 [148–944]), Latin America countries (Ecuador: 711 per 100 000 [226–1141]), central Latin America (Mexico: 583 per 100 000 [401–754]), some western European countries (Germany: 539 per 100 000 [163–816]), and central Asia (Kazakhstan, Uzbekistan, and Turkmenistan, with a range of 460–479 per 100 000 [116–770]). The lowest rates were in North Korea (178 per 100 000 [48–305]), Yemen (197 per 100 000 [37–352]), Bangladesh (199 per 100 000 [59–356]), Russia (211 per 100 000 [144–282]), Somalia (212 per 100 000 [34–460]), and China (215 per 100 000 [150–279]). The lowest age-standardised prevalence of epilepsy from idiopathic and secondary epilepsy combined was observed in east Asia (especially North Korea: 445 per 100 000 [312–583]; Indonesia: 460 per 100 000 [381–549]; and China: 464 per 100 000 [393–539]), and eastern Europe (Russia 498 per 100 000 [404–592]), whereas the highest age-standardised prevalence was in some countries of the Latin America and Caribbean region (Trinidad and Tobago: 1374 per 100 000 [945–1773]; and Dominica: 1262 per 100 000 [807–1647]) and some sub-Saharan African countries (Gabon: 1360 per 100 000 [823–1833]; and Equatorial Guinea: 1284 per 100 000 [750–1754]; table 1).

The age-standardised prevalence of idiopathic epilepsy showed four-fold geographical variations (figure 1A; appendix 1 pp 25–36), with the highest rates in some sub-Saharan African countries (Gabon: 688 per 100 000 [146–1158]; Angola: 542 [143–917]; and Zambia: 547 [148–944]), Latin America countries (Ecuador: 711 [226–1141]), central Latin America (Mexico: 583 [401–754]), some western European countries (Germany: 539 [163–816]), and central Asia (Kazakhstan, Uzbekistan, and Turkmenistan, with a range of 460–479 [116–770]). The lowest rates were in North Korea (178 [48–305]), Yemen (197 [37–352]), Bangladesh (199 [59–356]), Russia (211 [144–282]), Somalia (212 [34–460]), and China (215 [150–279]). The lowest age-standardised prevalence of epilepsy from idiopathic and secondary epilepsy combined was observed in east Asia (especially in North Korea: 445 per 100 000 [312–583]; Indonesia: 460 [381–549]; and China: 464 [393–539]), and eastern Europe (Russia: 498 [404–592]), whereas the highest age-standardised prevalence was in some countries of the Latin America and Caribbean region (Trinidad and Tobago: 1374 [945–1773]; and Dominica: 1262 [807–1647]) and some sub-Saharan African countries (Gabon: 1360 [823–1833]; and Equatorial Guinea: 1284 [750–1754]; table 1).

Age-standardised idiopathic epilepsy incidence, prevalence, death, and DALY rates per 100 000 people in GBD regions by SDI quintiles are presented in figure 2. Globally, across all SDI quintiles in almost all GBD regions, there was a trend towards reduction of age-standardised idiopathic epilepsy death and DALY rates, but an increase in the age-standardised idiopathic epilepsy incidence rate and some increase in age-standardised death rates in the high SDI quintile, although not substantially (appendix 1 pp 25–36, 53). The bulk of the idiopathic epilepsy incidence (82·1%), prevalence (80·4%), deaths (84·7%), and DALYs (87·9%) occurred in LMICs. From 1990 to 2021 (appendix 1 pp 51, 53), age-standardised death rates substantially increased in HICs (13·9% [3·4–20·3]) but substantially reduced in UMICs (41·1% decline [34·2–47·9]), LMICs (21·5% decline [10·7–31·5]), and LICs (15·6% decline [1·4–26·7]). Over the same period, UMICs and LMICs also had a substantial reduction in age-standardised DALY rates (30·3% [15·8–42·1] and 17·3% [2·2–28·7] decrease, respectively), and similar trend patterns were observed in age-standardised DALY rates by SDI quintiles—some reduction in the rates in high and lower SDI regions (appendix 1 p 54). However, no substantial changes from 1990 to 2021 were observed in age-standardised incidence rates and prevalence in any of the World Bank country income levels (appendix 1 p 53).

Although the global age-standardised incidence rates and prevalence of idiopathic epilepsy did not change substantially from 1990 to 2021 (12·3% [95% UI –4·8 to 32·6] and 6·9% [–9·7 to 25·5], respectively), the age-standardised death and DALY rates over that time reduced substantially (15·8% [8·8–22·8] and 14·5% [4·2–24·2] decline, respectively; appendix 1 pp 25–36). Age-standardised death rates in LICs (4·6 per 100 000 [3·9–5·6]) and LMICs (2·2 per 100 000 [1·7–2·5]), and age-standardised rates of DALYs in LICs (313·6 per 100 000 [248·0–392·3]) were greater than those in HICs and UMICs (LICs: 1·2 per 100 000 [1·1–1·2]; HICs and UMICs: 1·0 per 100 000 [0·9–1·1]; LICs: 129·2 per 100 000 [87·7–189·7]; and HICs and UMICs: 136·1 per 100 000 [97·8–182·2], respectively), especially in males (appendix 1 p 52). In 2021 (appendix 1 pp 25–36), there were large between-country variations in the age-standardised death rate of idiopathic epilepsy: the lowest rates were in Viet Nam (0·1 per 100 000 [0·0–0·3]) and San Marino (0·1 per 100 000 [0·1–0·2]) and the highest rates in Zambia (12·9 per 100 000 [9·5–17·1]) and Somalia (10·3 per 100 000 [6·7–16·9]). Similarly, there were large variations in the age-standardised DALY rates: the lowest rates were in San Marino (67·0 per 100 000 [20·3–146·5]), Russia (72·8 per 100 000 [48·2–110·6]), and Viet Nam (79·1 per 100 000 [18·1–169·2]), and the highest rates were in Zambia (746·5 per 100 000 [505·8–1031·4]) and Somalia (505·5 per 100 000 [305·5–770·1]).

In 2021, the lowest age-standardised incidence rate of idiopathic epilepsy (appendix 1 pp 25–36) was observed in North Korea (21·7 per 100 000 [95% UI 5·9–38·7]), Bangladesh (25·7 per 100 000 [7·4–45·5]), Papua New Guinea (27·9 per 100 000 [6·6–50·8]), and China (28·2 per 100 000 [19·0–37·9]), with the highest incidence rates in Ecuador (94·9 per 100 000 [29·9–160·5]), Germany (91·8 per 100 000 [27·5–140·5]), Equatorial Guinea (84·9 per 100 000 [20·6–140·9]), and Gabon (82·8 per 100 000 [17·8–140·9]).

## Discussion

Globally in 2021, across all ages, 0·7% of the population had active epilepsy. The overall global prevalence of active epilepsy from idiopathic and secondary epilepsy combined in our study (658 per 100 000 [95% UI 569–748]) is similar to the results of a recent meta-analysis of 197 epilepsy prevalence studies (638 per 100 000 [557–730]).^31^ Our estimates of the combined number of prevalent active epilepsy cases (51·7 million people), with the majority of the cases in LMICs (83·7%), were similar to those estimated by WHO in 2024.^32^ Similar to previous studies,^31^ we found no substantial difference in the age-standardised prevalence of active epilepsy between LMICs and HICs. However, from 1990 to 2021, there was a substantial increase in the age-standardised prevalence of secondary epilepsy in southeast Asia and sub-Saharan Africa, and a substantial decrease in age-standardised death and DALY rates from idiopathic epilepsy. We also found that the age-standardised prevalence of secondary epilepsy as well as age-standardised death and DALY rates of idiopathic epilepsy are higher in LICs than in HICs, but the age-standardised prevalence of idiopathic epilepsy or all-cause epilepsy does not have a substantial difference by income level. The observed reduction in death and DALY rates from idiopathic epilepsy might be related to improved access and treatment of idiopathic epilepsy.^5^

The substantial increase in the prevalence of secondary epilepsy in non-high-income regions was likely related to the greater exposure of the population of these countries to perinatal risk factors^33^ and higher rates of CNS zoonotic and other infections.^34^, ^35^ In addition, poorer treatment (limited availability of antiseizure medications and access to specialist antiepileptic services) might also contribute to the greater age-standardised prevalence and DALYs in LICs and some LMICs compared with HICs and countries with higher SDI. It is also possible that case verification of epilepsy in high-income regions is better than in non-high-income regions, which might contribute to the observed differences. The true gap between the burden of epilepsy in HICs or high SDI countries and LICs or low SDI countries is probably even greater because of possible under-reporting of cases of epilepsy in LICs or low SDI countries, often due to stigmatisation of the disease in many countries.^36^

We also found three-fold to four-fold geographical variations in the prevalence of idiopathic and secondary epilepsy combined, secondary epilepsy, and idiopathic epilepsy, with slightly greater age-standardised prevalence in males compared with females. Substantial geographical variations in the prevalence of active epilepsy, with the predominance in LMICs, and slightly higher rates in males were also shown in other studies,^31^, ^37^, ^38^ whereas some studies found significant greater burden of epilepsy in males compared with females.^10^ Congruent with previous studies, we found that the prevalence of idiopathic epilepsy was relatively low early in life, increased during adolescence, and decreased after age 30 years,^31^ but unlike previous studies that showed fairly constant prevalence after age 30 years,^31^, ^39^ we found a substantial increase in the prevalence of idiopathic epilepsy after age 55 years, especially noticeable for secondary epilepsy. The large increase in the prevalence of secondary epilepsy in the elderly (after age 90 years) is likely to be related to the increase in the prevalence of stroke, brain injuries, and neurodegenerative disorders in this age group.^20^ Another likely reason is the reluctance to investigate underlying causes of epilepsy in the elderly, particularly if they have dementia, stroke, or other degenerative diseases. Many older people only have a CT scan that can only detect gross structural lesions, and cannot reliably detect temporal lobe lesions, whereas MRI is the investigation of choice in epilepsy. This investigation is often not done in older people because the need to keep still for longer and can sometimes require a general anaesthetic.

In 2021, active idiopathic epilepsy led to almost 14 million DALYs, or 0·5% of DALYs from idiopathic and secondary epilepsy combined; the age-standardised incidence, death, and DALY rates of active idiopathic epilepsy were 42·8 per 100 000 (95% UI 31·2–53·7), 1·7 per 100 000 (1·5–1·9), and 177·8 per 100 000 (137·7–225·9), respectively. Although the age-standardised death and DALY rates of idiopathic epilepsy substantially decreased over time (appendix 1 p 52), the age-standardised incidence rate showed a non-significant trend towards increasing. Greater incidence of epilepsy (mainly epilepsy from idiopathic and secondary epilepsy combined) in LMICs was also found in a recent meta-analysis.^31^ The very low age-standardised incidence and death rates in some LMICs, such as Viet Nam and San Marino, is difficult to explain, but at least part of it might be related to the possible effect of stigma and cultural or organisational differences in epilepsy reporting.^31^ Data sources for epilepsy are also very limited in some countries. For example, there were no non-fatal epilepsy data source in San Marino, and the last cause-of-death source was from 2005. There were two non-fatal data sources in Viet Nam, both from rural areas, and only two cause-of-death sources (no vital registration system). Similarly, the causes for decreasing death and DALY rates (particularly in LMICs) are not clear but might be related to the emerging better treatment, improved identification of less severe events, with lower death rates of epilepsy and CNS infections in these countries.^20^, ^40^ The increasing incidence of idiopathic epilepsy (particularly in middle-income countries) might reflect better identification of cases of idiopathic epilepsy in these countries over that time period.

Globally, between 1990 and 2021, there was a substantial increase not only in prevalent cases of all types of epilepsy, but also an increase in the age-standardised prevalence of secondary and combined epilepsy, whereas the age-standardised prevalence of idiopathic epilepsy did not change substantially. As prevalence of many neurological conditions (eg, cerebral malaria, neonatal encephalopathy, neonatal sepsis, and nervous system cancer) have increased over the past three decades,^20^ it is probably not surprising that the prevalence of secondary epilepsy, often related to them, also increased from 1990 to 2021.

The effect of the COVID-19 pandemic on patients with epilepsy has been substantial, including increased poor COVID-19 outcomes, mental health challenges, and difficulties in the self-management of epilepsy.^41^ For example, a survey in Brazil reported worsening of seizure control due to cancellation of appointments and challenges in access to medications.^42^ COVID-19 was also associated with decreased confidence of health care in remote management of epilepsy,^43^ worsening or aggravating of pre-existing epilepsy,^44^, ^45^, ^46^ and the risk of de novo seizures,^47^ which exceeds the risk of seizure or epilepsy after influenza.^48^ These observations highlight the necessity of further research on the long-term consequences of decreased epilepsy diagnosis and care, and its subsequent increase in epilepsy-associated mortality.^46^ Addressing these issues can help the scientific community and health-care policy makers at both global and national levels recognise gaps and insufficiencies. This awareness can lead to better preparedness for managing similar global health challenges in the future, thereby reducing the additional burden on epilepsy patients.

The major strength of this study is that it provides the most up-to-date prevalence estimates of active idiopathic epilepsy and active secondary epilepsy, as well as the prevalence of active epilepsy from idiopathic and secondary epilepsy combined on the global, regional, and national (204 countries) levels by age and sex for the 1990–2021 period. These data are of crucial importance for health-care planning, prevention, resource allocation, and workforce development. However, there are some general and epilepsy-specific limitations of the study detailed in our previous 1990–2016 GBD epilepsy burden paper.^8^ The most important limitation of the study is the scarcity of reliable population-based epidemiological studies on various types of epilepsy in most countries of the world. In addition, the GBD study cannot provide analysis of all causes of secondary epilepsy (eg, stroke, degenerative diseases, and zoonotic diseases) and various phenotypes of idiopathic epilepsy (eg, juvenile myoclonic epilepsy, childhood absence epilepsy, juvenile absence epilepsy, and genetic generalised epilepsy) due to the scarcity of such reliable estimates in most countries. Although from previous research we know that the most common structural causes of secondary epilepsy include various neurological conditions, including traumatic brain injury, stroke, CNS zoonotic disorders, neuroinfectious diseases, neurodegenerative diseases, brain tumour, and various neural development lesions,^49^ accounting for remaining aetiologies explicitly would be desirable in future GBD rounds. We also acknowledge that many studies on epilepsy included in the GBD analysis are not nationally representative but instead focus on smaller populations within a geographic location and we had limited ability to adjust for quality of studies. Another important limitation was the scarcity of reliable data on risk factors of idiopathic epilepsy sufficient for the GBD modelling.

In conclusion, our estimates of incidence, death, prevalence, and DALYs show diverging trends in the burden of epilepsy in the world, with the bulk of the burden residing in LMICs. Urgent efforts must be made by all key stakeholders and decision makers to increase awareness and education about epilepsy, eliminate stigmatisation and discrimination associated with epilepsy, better control secondary causes of epilepsy (stroke, CNS zoonotic diseases, and other infectious diseases), improve access to existing treatments in economically disadvantaged countries or populations, and foster workforce development, especially in LMICs. Such initiatives are important for the implementation of the WHO intersectoral global action plan on epilepsy and other neurological disorders 2022–2031 and Universal Health Coverage,^6^, ^50^ and particularly for LMICs, in which three-quarters of people with epilepsy do not get the treatment they need,^32^ and access to specialised neurological care is very limited.^20^ Further research on risk factors of idiopathic epilepsy, good-quality long-term epilepsy surveillance studies, and examination of the possible effects of stigma and cultural differences on seeking medical attention for epilepsy, as well as developing new effective and affordable treatments, need to be explored.

### GBD 2021 Epilepsy Collaborators

### Affiliations

### Contributors

### Data sharing

To download GBD data used in these analyses, please visit the GBD 2021 Sources Tool website. To download forecasted estimates used in these analyses, please visit the GBD visualisation tools at https://collab2021.healthdata.org/gbd-compare/.

Editorial note: The Lancet Group takes a neutral position with respect to territorial claims in published maps and institutional affiliations.

## Declaration of interests

S Afzal reports support for the present manuscript from King Edward Medical University for study material, research articles, valid data sources and authentic real time information for this manuscript. S Afzal also reports payment or honoraria for lectures, presentations, speakers bureaus, manuscript writing or educational events from King Edward Medical University and collaborative partners including University of Johns Hopkins, University of California, University of Massachusetts, KEMCAANA, KEMCA_UK; support for attending meetings and/or travel from King Edward Medical University; participation on a Data Safety Monitoring Board or Advisory Board with National Bioethics Committee Pakistan, King Edward Medical University Ethical Review Board, Ethical Review Board Fatima Jinnah Medical University and Sir Ganga Ram Hospital as a Member of the Technical Working Group on Infectious Diseases; leadership or fiduciary roles in board, society, committee or advocacy groups, paid or unpaid with the Pakistan Association of Medical Editors, the Faculty of Public Health Royal Colleges UK (FFPH) as Fellow, the Society of Prevention, Advocacy And Research, King Edward Medical University. (SPARK), and with the Pakistan Society of Infectious Diseases as a member; other financial or non-financial interest serving as Dean of Public Health and Preventive Medicine King Edward Medical University, as Chief Editor Annals of King Edward Medical University since 2014, as Director of Quality Enhancement Cell King Edward Medical University, as Principal School of Artificial Intelligence at International Level, as Fellow of Faculty of Public Health United Kingdom, as Advisory Board Member and Chair Scientific Session KEMCA-United Kingdom, as Chairperson International Scientific Conference KEMCAANA United States, as Member of the Research and Publications Higher Education Commission HEC Pakistan, as Member of the Research and Journals Committee Pakistan Medical and Dental Council, as Member of the National Bioethics Committee Pakistan, as Member of the Corona Experts Advisory Group (Punjab), as Member Dengue Experts Advisory Group (Punjab), and as Chair of the Punjab Residency Program Research Committee; all outside the submitted work. R Ancuceanu reports consulting fees from Abbvie and Merck Romania; payment or honoraria for lectures, presentations, speakers bureaus, manuscript writing or educational events from Abbvie, Laropharm, Reckitt, and Merck Romania; support for attending meetings and/or travel from Merck Romania; all outside the submitted work. S Bhaskar reports grants or contracts from Japan Society for the Promotion of Science (JSPS), Japanese Ministry of Education, Culture, Sports, Science and Technology (MEXT) for a Grant-in-Aid for Scientific Research (KAKENHI) (Grant ID: 23KF0126) and from JSPS and the Australian Academy of Science for a JSPS International Fellowship (Grant ID: P23712); leadership or fiduciary roles in board, society, committee or advocacy groups, paid or unpaid with Rotary District 9675, Sydney, Australia as District (Chair, Diversity, Equity & Inclusion), Global Health & Migration Hub Community, Global Health Hub Germany, Berlin, Germany (Chair, Founding Member and Manager), PLOS One, BMC Neurology, Frontiers in Neurology, Frontiers in Stroke, Frontiers in Public Health, Journal of Aging Research, Neurology International, Diagnostics, & BMC Medical Research Methodology (Editorial Board Member), College of Reviewers, Canadian Institutes of Health Research (CIHR), Government of Canada (Member), World Headache Society, Bengaluru, India (Director of Research), Cariplo Foundation, Milan, Italy (Expert Adviser/Reviewer), National Cerebral and Cardiovascular Center, Department of Neurology, Suita, Osaka, Japan (Visiting Director), Cardiff University Biobank, Cardiff, UK (Member, Scientific Review Committee), Rotary Reconciliation Action Plan (Chair); all outside the submitted work. S Cortese reports grants or contracts from NIHR and the European Research Agency; payment or honoraria for lectures, presentations, speakers bureaus, manuscript writing or educational events from the British Association of Psychopharmacology (BAP), Canadian ADHD Resource Alliance (CADDRA), Medice, and the Association for Child and Adolescent Mental Health (ACAMH); all outside the submitted work. A Hassan reports consulting fees from Novartis, Sanofi Genzyme, Biologix, Merck, Hikma Pharma, Janssen, Inspire Pharma, Future Pharma, and Elixir Pharma; payment or honoraria for lectures, presentations, speakers bureaus, manuscript writing or educational events from Novartis, Allergan, Merck, Biologix, Janssen, Roche, Sanofi Genzyme, Bayer, Hikma Pharma, Al Andalus, Chemipharm, Lundbeck, Inspire Pharma, Future Pharma and Habib Scientific Office, and Everpharma; support for attending meetings and/or travel from Novartis, Allergan, Merck, Biologix, Roche, Sanofi Genzyme, Bayer, Hikma Pharma, Chemipharm, Al Andalus, and Clavita Pharm; leadership or fiduciary role in other board, society, committee or advocacy group, paid or unpaid, as vice president of MENA Headache Society, board member of the Headache Chapter of the Egyptian Society of Neurology, board member of Multiple Sclerosis Chapter of the Egyptian Society of Neurology, member of the Committee of Education of the International Headache Society (IHS), membership committee of IHS, and regional committee of IHS; all outside the submitted work. I Ilic reports supports from the present manuscript from the Ministry of Science, Technological Development and Innovation of the Republic of Serbia (project no. 175042, 2011-2023). M Ilic reports support for the present manuscript from the Ministry of Science, Technological Development and Innovation of the Republic of Serbia (project no. 451-03-47/2023-01/200111). K Krishan reports non-financial support from the UGC Centre of Advanced Study, CAS II, awarded to the Department of Anthropology, Panjab University, Chandigarh, India, outside the submitted work. H R Marateb reports grants or contracts paid to their institution from Beatriu de Pinós post-doctoral program from the Office of the Secretary of Universities and Research from the Ministry of Business and Knowledge of the Government of Catalonia program: 2020 BP 00261, outside the submitted work. L Monasta reports support for the present manuscript from the Italian Ministry of Health (Ricerca Corrente 34/2017), payments made to the Institute for Maternal and Child Health IRCCS Burlo Garofolo. S K Panda reports support for the present manuscript via salary from Siksha ‘O’ Anusandhan (Deemed to be University). S K Panda also reports grants or contracts from DST-GOVT. OF ODISHA (Letter No. 3444/ST) outside the submitted work. R Passera reports participation on a Data Safety Monitoring Board or Advisory Board as a member of the Data Safety Monitoring Board of the clinical trial “Consolidation with ADCT-402 (loncastuximab tesirine) after immunochemotherapy: a phase II study in BTKi-treated/ineligible Relapse/Refractory Mantle Cell Lymphoma (MCL) patients” - FIL, Fondazione Italiana Linfomi, Alessandria; leadership or fiduciary roles in board, society, committee or advocacy groups as Member of the EBMT Statistical Committee, European Society for Blood and Marrow Transplantation, Paris (F) (unpaid) and as a past member of 2020-2023 (biostatistician) of the IRB/IEC Comitato Etico AO SS. Antonio e Biagio Alessandria-ASL AL-VC (paid reimbursement of expenses); all outside the submitted work. I Rautalin reports support for the present manuscript from Sigrid Juselius Foundation, Finnish Medical Foundation, Sakari Alhopuro Foundation, Finnish Foundation for Cardiovascular Research, Maud Kuistila Foundation for personal research grants with no role in the design and conduct of the study; in the collection, management, analysis, and interpretation of the data; or in the manuscript's preparation, review, or approval. U Saeed reports support for the present manuscript from the Ontario Graduate Scholarship awarded at the University of Toronto, Canada. Y L Samodra reports leadership or fiduciary roles in board, society, committee or advocacy groups, paid or unpaid with Benang Merah Research Center (bmrc.id) as Co-founder, outside the submitted work. V Sharma reports other financial or non-financial support from DFSS (MHA)‘s research project (DFSS28(1)2019/EMR/6) at Institute of Forensic Science & Criminology, Panjab University, Chandigarh, India, outside the submitted work. J A Singh reports consulting fees from ROMTech, Atheneum, Clearview Healthcare Partners, American College of Rheumatology, Yale, Hulio, Horizon Pharmaceuticals, DINORA, ANI/Exeltis, USA Inc., Frictionless Solutions, Schipher, Crealta/Horizon, Medisys, Fidia, PK Med, Two labs Inc., Adept Field Solutions, Clinical Care Options, Putnam Associates, Focus Forward, Navigant Consulting, Spherix, MedIQ, Jupiter Life Science, UBM LLC, Trio Health, Medscape, WebMD, and Practice Point Communications, and the National Institutes of Health; payment or honoraria for lectures, presentations, speakers bureaus, manuscript writing or educational events from Simply Speaking; support for attending meetings and/or travel from OMERACT as past steering committee member; participation on a Data Safety Monitoring Board or Advisory Board, unpaid, with the FDA Arthritis Advisory Committee; leadership or fiduciary roles in board, society, committee or advocacy groups, paid or unpaid, with OMERACT as past steering committee member, the Veterans Affairs Rheumatology Field Advisory Committee as Chair, and the UAB Cochrane Musculoskeletal Group Satellite Center on Network Meta-analysis as Editor and Director; stock or stock options in Atai Life Sciences, Kintara Therapeutics, Intelligent Biosolutions, Acumen Pharmaceutical, TPT Global Tech, Vaxart pharmaceuticals, Atyu Biopharma, Adaptimmune Therapeutics, GeoVax Labs, Pieris Pharmaceuticals, Enzolytics Inc., Seres Therapeutics, Tonix Pharmaceuticals Holding Corp., Aebona Pharmaceuticals, and Charlotte's Web Holdings, Inc. and previously-owned stock options in Amarin, Viking and Moderna Pharmaceuticals; all outside the submitted work. R Tabarés-Seisdedos reports grants or contracts from the Valencian Regional Government's Ministry of Education (PROMETEO/CIPROM/2022/58) and the Spanish Ministry of Science, Innovation and Universities (PID2021-129099OB-I00) outside the submitted work. J H V Ticoalu reports leadership or fiduciary roles in board, society, committee or advocacy groups, paid or unpaid with Benang Merah Research Center (bmrc.id) as Co-founder, outside the submitted work. S J Tromans reports grants or contracts paid to their institution, University of Leicester, from NHS Digital, via the Department of Health and Social Care as part of the 2023 Adult Psychiatric Morbidity Survey team collecting epidemiological data on community-based adults living in England; leadership or fiduciary roles in other board, society, committee or advocacy groups, paid or unpaid, with the Neurodevelopmental Psychiatry Special Interest Group and Psychiatry of Intellectual Disability Faculty at the Royal College of Psychiatrist as Academic Secretary, with BMC Psychiatry, Advances in Autism, Advances in Mental Health and Intellectual Disability, and Progress in Neurology and Psychiatry as Editorial Board Member, and with Psychiatry of Intellectual Disability Across Cultures (Oxford University Press) as Editor; all outside the submitted work. S Wiebe reports grants or contracts from Alberta Strategy for patient-oriented research and Epilepsy Canada; consulting fees from UCB Pharm, Eisai, Sunovion, and Liva Nova for educational grants paid to their institution; payment or honoraria for lectures, presentations, speakers bureaus, manuscript writing or educational events from Torrent Pharma and Biopas; participation on Advisory Boards with Jazz Pharmaceuticals and Paladyn Labs; leadership or fiduciary roles in board, society, committee or advocacy groups, paid or unpaid with the International League Against Epilepsy as Executive Committee Member; all outside the submitted work. M Zielińska reports other financial or non-financial interests as an AstraZeneca employee outside the submitted work.
