# Supplementary material for: Global, regional, and national burden of epilepsy, 1990–2021: a systematic analysis for the Global Burden of Disease Study 2021
Source: Lancet Public Health. 2025 Feb 24;10(3):e203–27. doi: 10.1016/S2468-2667(24)00302-5 (PMC11876103; doi:10.1016/S2468-2667(24)00302-5)
Supplement: Supplementary appendix 1 [file mmc1.pdf]

THE LANCET
Public Health

Supplementary appendix 1

This appendix formed part of the original submission and has been peer reviewed. We post it as supplied by the authors.

Supplement to: GBD Epilepsy Collaborators. Global, regional, and national burden of epilepsy, 1990–2021: a systematic analysis for the Global Burden of Disease Study 2021. *Lancet Public Health* 2025; published online Feb 24. https://doi.org/10.1016/S2468-2667(24)00302-5.

The Lancet Public Health

Appendix 1: Authorship appendix to "Global, regional, and national burden of epilepsy, 1990–2021: a systematic analysis for the Global Burden of Disease Study 2021"

This appendix provides further authorship detail for "Global, regional, and national burden of epilepsy, 1990–2021: a systematic analysis for the Global Burden of Disease Study 2021"

Table of Contents

GBD 2021 Epilepsy Collaborators **2**

Affiliations **4**

Authors' Contributions **17**

Managing the overall research enterprise 17

Writing the first draft of the manuscript 17

Primary responsibility for applying analytical methods to produce estimates 17

Primary responsibility for seeking, cataloguing, extracting, or cleaning data; designing or coding figures and tables 18

Providing data or critical feedback on data sources 18

Developing methods or computational machinery 19

Providing critical feedback on methods or results 19

Drafting the work or revising it critically for important intellectual content 21

Managing the estimation or publications process 23

GBD 2021 Epilepsy Collaborators

Valery L Feigin,* Theo Vos,* Balakrishnan Sukumaran Nair, Simon I Hay, Yohannes Habtegiorgis Abate, Abdallah H A Abd Al Magied, Samar Abd ElHafeez, Atef Abdelkader, Mohammad-Amin Abdollahifar, Auwal Abdullahi, Richard Gyan Aboagye, Lucas Guimarães Abreu, Samir Abu Rumeileh, Hasan Abualruz, Salahdein Aburuz, Ahmed Abu-Zaid, Isaac Yeboah Addo, Rufus Adesoji Adedoyin, Abiola Victor Adepoju, Muhammad Sohail Afzal, Saira Afzal, Aqeel Ahmad, Sajjad Ahmad, Tauseef Ahmad, Ali Ahmadi, Amir Mahmoud Ahmadzade, Ayman Ahmed, Haroon Ahmed, Mehrunnisha Sharif Ahmed, Muktar Beshir Ahmed, Salah Al Awaidy, Omar Al Omari, Yazan Al-Ajlouni, Mohammed Albashtawy, Bassam Al-Fatly, Abdelazeem M Algammal, Abid Ali, Mohammed Usman Ali, Syed Shujait Ali, Waad Ali, Sheikh Mohammad Alif, Joseph Uy Almazan, Najim Z Alshahrani, Awais Altaf, Mohammad Al-Wardat, Yaser Mohammed Al-Worafi, Hany Aly, Karem H Alzoubi, Sohrab Amiri, Robert Ancuceanu, Dhanalakshmi Angappan, Mohammed Tahir Ansari, Saeid Anvari, Anayochukwu Edward Anyasodor, Jalal Arabloo, Mosab Arafat, Aleksandr Y Aravkin, Brhane Berhe Aregawi, Abdulfatai Aremu, Maha Moh'd Wahbi Atout, Alok Atreya, Avinash Aujayeb, Setognal Birara Aychiluhm, Shahkaar Aziz, Ahmed Y Azzam, Ashish D Badiye, Ruhai Bai, Atif Amin Baig, Shankar M Bakkannavar, Soham Bandyopadhyay, Indrajit Banerjee, Mainak Bardhan, Suzanne Lyn Barker-Collo, Amadou Barrow, Zarrin Basharat, Azadeh Bashiri, Afisu Basiru, Mohammad-Mahdi Bastan, Sai Batchu, Babak Behnam, Diana Fernanda Bejarano Ramirez, Maryam Bemanalizadeh, Kebede A Beyene, Devidas S Bhagat, Akshaya Srikanth Bhagavathula, Sonu Bhaskar, Ajay Nagesh Bhat, Gurjit Kaur Bhatti, Jasvinder Singh Bhatti, Mohiuddin Ahmed Bhuiyan, Soumitra S Bhuyan, Cem Bilgin, Francesca Bisulli, Archith Boloor, Sri Harsha Boppana, Souad Bouaoud, Yasser Bustanji, Mehtap Çakmak Barsbay, Felix Carvalho, Joao Mauricio Castaldelli-Maia, Rama Mohan Chandika, Vijay Kumar Chattu, Anis Ahmad Chaudhary, Patrick R Ching, Hitesh Chopra, Dinh-Toi Chu, Hongyuan Chu, Samuele Cortese, Paolo Angelo Cortesi, Natalia Cruz-Martins, Omid Dadras, Xiaochen Dai, Emanuele D'Amico, Lalit Dandona, Rakhi Dandona, Samuel Demissie Darcho, Amira Hamed Darwish, Amol S Dhane, Vishal R Dhulipala, Michael J Diaz, Thanh Chi Do, Sushil Dohare, Ojas Prakashbhai Doshi, Haneil Larson Dsouza, Arkadiusz Marian Dziedzic, Alireza Ebrahimi, Negin Eissazade, Michael Ekholuenetale, Rabie Adel El Arab, Ibrahim Farahat El Bayoumy, Omar Abdelsadek Abdou El Meligy, Hala Rashad Elhabashy, Muhammed Elhadi, Chadi Eltaha, Adeniyi Francis Fagbamigbe, Ayesha Fahim, Jawad Fares, Mohsen Farjoud Kouhanjani, Abidemi Omolara Fasanmi, Ali Fatehizadeh, Patrick Fazeli, Timur Fazylov, Ginenus Fekadu, Seyed-Mohammad Fereshtehnejad, Pietro Ferrara, Nuno Ferreira, Getahun Fetensa, Florian Fischer, Matteo Foschi, Muktar A Gadanya, Yaseen Galali, Balasankar Ganesan, Xiang Gao, Ravindra Kumar Garg, Miglas Welay Gebregergis, Fataneh Ghadirian, Seyyed-Hadi Ghamari, Jaleed Ahmed Gilani, Alem Abera Girmay, Giorgia Giussani, Elena V Gnedovskaya, Mahaveer Golechha, Mahdi Gouravani, Ayman Grada, Shi-Yang Guan, Sapna Gupta, Mohammad Haghani Dogahe, Arvin Haj-Mirzaian, Nadia M Hamdy, Netanja I Harlianto, Ahmed I Hasaballah, Hamidreza Hasani, Amr Hassan, Ikrama Ibrahim Hassan, Mahgol Sadat Hassan Zadeh Tabatabaei, Omar E Hegazi, Golnaz Heidari, Mehdi Hemmati, Kamal Hezam, Nguyen Quoc Hoan, Ramesh Holla, Mehdi Hosseinzadeh, Junjie Huang, Hong-Han Huynh, Bing-Fang Hwang, Segun Emmanuel Ibitoye, Adalia Ikiroma, Olayinka Stephen Ilesanmi, Irena M Ilic, Milena D Ilic, Mohammad Tarique Imam, Mustapha Immurana, Arit Inok, Md Rabiul Islam, Chidozie Declan Iwu, Louis Jacob, Abdollah Jafarzadeh, Haitham Jahrami, Ammar Abdulrahman Jairoun, Mihajlo Jakovljevic, Reza Jalilzadeh Yengejeh, Roland Dominic G Jamora, Talha Jawaid, Sathish Kumar Jayapal, Zixiang Ji, Jost B Jonas, Nitin Joseph, Charity Ehimwenma Joshua, Zubair Kabir, Rizwan Kalani, Arun Kamireddy, Kehinde Kazeem Kanmodi, Neeti Kapoor, Faizan Zaffar Kashoo, Harkiran Kaur, Foad Kazemi, Himanshu Khajuria, Alireza Khalilian, Maseer Khan, Haitham Khatatbeh, Hamid Reza Khayat

Kashani, Khalid A Kheirallah, Feriha Fatima Khidri, Moein Khormali, Atulya Aman Khosla, Jagdish Khubchandani, Yun Jin Kim, Yun Seo Kim, Ruth W Kimokoti, Hyun Yong Koh, Ali-Asghar Kolahi, Karel Kostev, Kewal Krishan, Vijay Krishnamoorthy, Jera Kruja, Mohammed Kuddus, Mukhtar Kulimbet, G Anil Kumar, Manasi Kumar, Satyajit Kundu, Ville Kytö, Chandrakant Lahariya, Dharmesh Kumar Lal, Judit Lám, Iván Landires, Francesco Lanfranchi, Nhi Huu Hanh Le, Seung Won Lee, Virendra S Ligade, Stephen S Lim, Christine Linehan, Xiaofeng Liu, Xuefeng Liu, José Francisco López-Gil, Giancarlo Lucchetti, Azeem Majeed, Kashish Malhotra, Ahmad Azam Malik, Vahid Mansouri, Hamid Reza Marateb, Miquel Martorell, Roy Rillera Marzo, Yasith Mathangasinghe, Rishi P Mediratta, Man Mohan Mehndiratta, Hadush Negash Meles, Endalkachew Belayneh Melese, George A Mensah, Atte Meretoja, Tomislav Mestrovic, Sachith Mettananda, Giuseppe Minervini, Reza Mirfakhraie, Moonis Mirza, Awoke Misganaw, Arup Kumar Misra, Abdalla Z Mohamed, Nouh Saad Mohamed, Abdollah Mohammadian-Hafshejani, Ibrahim Mohammadzadeh, Syam Mohan, Ali H Mokdad, Lorenzo Monasta, AmirAli Moodi Ghalibaf, Maryam Moradi, Rohith Motappa, Lorenzo Muccioli, Francesk Mulita, Yanjinlkham Munkhsaikhan, Efren Murillo-Zamora, Sathish Muthu, Amin Nabavi, Ganesh R Naik, Shumaila Nargus, Abdulqadir J Nashwan, Zuhair S Natto, Javaid Nauman, Muhammad Naveed, Biswa Prakash Nayak, Athare Nazri-Panjaki, Gaurav Nepal, Henok Biresaw Netsere, Hau Thi Hien Nguyen, Robina Khan Niazi, Ali Nikoobar, Majid Nozari, Chisom Adaobi Nri-Ezedi, Vincent Ebuka Nwatah, Ogochukwu Janet Nzoputam, Bogdan Oancea, Andrew T Olagunju, Oladotun Victor Olalusi, Ahmed Omar Bali, Michal Ordak, Verner N Orish, Esteban Ortiz-Prado, Nikita Otstavnov, Amel Ouyahia, Mayowa O Owolabi, Alicia Padron-Monedero, Jagadish Rao Padubidri, Sujogya Kumar Panda, Songhomitra Panda-Jonas, Deepshikha Pande Katare, Anamika Pandey, Leonidas D Panos, Ioannis Pantazopoulos, Paraskevi Papadopoulou, Utsav Parekh, Romil R Parikh, Nicholas Parsons, Roberto Passera, Shankargouda Patil, Shrikant Pawar, Hamidreza Pazoki Toroudi, Umberto Pensato, Prince Peprah, Mario F P Peres, Simone Perna, Hoang Nhat Pham, Zahra Zahid Piracha, Michael A Piradov, Dimitri Poddighe, Ramesh Poluru, Ahmad Pour-Rashidi, Jalandhar Pradhan, Manya Prasad, Dimas Ria Angga Pribadi, Jagadeesh Puvvula, Nameer Hashim Qasim, Venkatraman Radhakrishnan, Pankaja Raghav, Fakher Rahim, Mosiur Rahman, Amir Masoud Rahmani, Mohammad Rahmanian, Adarsh Raja, Ali Rajabpour Sanati, Pushp Lata Rajpoot, Mahmoud Mohammed Ramadan, Shakthi Kumaran Ramasamy, Nemanja Rancic, Sowmya J Rao, Mohammad-Mahdi Rashidi, Devarajan Rathish, Salman Rawaf, Murali Mohan Rama Krishna Reddy, Elrashdy M Moustafa Mohamed Redwan, Mohsen Rezaeian, Taeho Gregory Rhee, Muhammad Riaz, Jefferson Antonio Buendia Rodriguez, Leonardo Roever, Marina Romozzi, Moustaq Karim Khan Rony, Kevin T Root, Himanshu Sekhar Rout, Aly M A Saad, Cameron John Sabet, Basema Ahmad Saddik, Reihaneh Sadeghian, Mohammad Reza Saeb, Umar Saeed, Usman Saeed, Fatemeh Saheb Sharif-Askari, Narjes Saheb Sharif-Askari, Amirhossein Sahebkar, Zahra Saif, S Mohammad Sajadi, Afeez Abolarinwa Salami, Sohrab Salimi, Yoseph Leonardo Samodra, Abdallah M Samy, Gargi Sachin Sarode, Sachin C Sarode, Brijesh Sathian, Anudeep Sathyanarayan, Maheswar Satpathy, Monika Sawhney, Siddharthan Selvaraj, Mohammad H Semreen, Ashenafi Kibret Sendekie, Subramanian Senthilkumaran, Yashendra Sethi, Allen Seylani, Ataollah Shahbandi, Samiah Shahid, Masood Ali Shaikh, Summaiya Zareen Shaikh, Muhammad Aaqib Shamim, Mehran Shams-Beyranvand, Alfiya Shamsutdinova, Amin Sharifan, Javad Sharifi Rad, Anupam Sharma, Vishal Sharma, Maryam Shayan, Zubeda Begum Sheikh, Mahabalesh Shetty, Pavanchand H Shetty, Premalatha K Shetty, Aminu Shittu, Nathan A Shlobin, Seyed Afshin Shorofi, Sunil Shrestha, Emmanuel Edwar Siddig, Gagandeep Singh, Harmanjit Singh, Jasvinder A Singh, Paramdeep Singh, Puneetpal Singh, Surjit Singh, Shipra Solanki, Soroush Soraneh, Muhammad Haroon Stanikzai, Mark J M Sullman, Katharina S Sunnerhagen, Vinay Suresh, Chandan Kumar Swain, Lukasz Szarpak, Payam Tabaee

Damavandi, Rafael Tabarés-Seisdedos, Celine Tabche, Jabeen Taiba, Manoj Tanwar, Minale Tareke, Mohamad-Hani Temsah, Reem Mohamad Hani Temsah, Masayuki Teramoto, Pugazhenthan Thangaraju, Sathish Thirunavukkarasu, Jansje Henny Vera Ticoalu, Tenaw Yimer Tiruye, Krishna Tiwari, Vikas Kumar Tiwari, Marcos Roberto Tovani-Palone, Thang Huu Tran, Nguyen Tran Minh Duc, Manjari Tripathi, Samuel Joseph Tromans, Daniel Hsiang-Te Tsai, Aristidis Tsatsakis, Evangelia Eirini Tsermpini, Munkhtuya Tumurkhuu, Aniefiok John Udoakang, Saeed Ullah, Muhammad Umair, Bhaskaran Unnikrishnan, Daniele Urso, Jibrin Sammani Usman, Asokan Govindaraj Vaithinathan, Alireza Vakilian, Ravi Prasad Varma, Narayanaswamy Venketasubramanian, Jorge Hugo Villafañe, Manish Vinayak, Andres Fernando Vinueza Veloz, Mandaras Tariku Walde, Shu Wang, Yanzhong Wang, Abdul Waris, Nuwan Darshana Wickramasinghe, Andrea Sylvia Winkler, Subah Abderehim Yesuf, Arzu Yiğit, Vahit Yiğit, Mekdes Tigistu Yilma, Yazachew Engida Yismaw, Dong Keon Yon, Naohiro Yonemoto, Chuanhua Yu, Milad Zandi, Aurora Zanghì, Mohammed G M Zeariya, Zhongyi Zhao, Claire Chenwen Zhong, Magdalena Zielińska, Osama A Zitoun, Sa'ed H Zyoud, Samer H Zyoud, Ilari Rautalin,[§] Charles Richard James Newton,[§] Samuel Wiebe,[§] and Christopher J L Murray.[§]

*Co-first authors

§Senior authors

Affiliations

National Institute for Stroke and Applied Neurosciences (Prof V L Feigin PhD), National Institute of Stroke and Applied Neurosciences (B S Nair MPH), The National Institute for Stroke and Applied Neurosciences (I Rautalin PhD), Auckland University of Technology, Auckland, New Zealand; Institute for Health Metrics and Evaluation (Prof V L Feigin PhD, Prof T Vos PhD, Prof S I Hay FMedSci, A Y Aravkin PhD, X Dai PhD, Prof L Dandona MD, Prof R Dandona PhD, Prof S S Lim PhD, T Mestrovic PhD, Prof A H Mokdad PhD, Prof C J L Murray DPhil), Department of Health Metrics Sciences, School of Medicine (Prof T Vos PhD, Prof S I Hay FMedSci, A Y Aravkin PhD, X Dai PhD, Prof R Dandona PhD, Prof S S Lim PhD, A Misganaw PhD, Prof A H Mokdad PhD, Prof C J L Murray DPhil), Department of Applied Mathematics (A Y Aravkin PhD), School of Health Systems and Public Health (C Iwu MPH), Department of Neurology (R Kalani MD), Department of Anesthesiology & Pain Medicine (V Krishnamoorthy MD), University of Washington, Seattle, WA, USA; Third Department of Neurology (E V Gnedovskaya PhD), Research Center of Neurology, Moscow, Russia (Prof V L Feigin PhD, Prof M A Piradov DSc); Department of Clinical Governance and Quality Improvement (Y H Abate MSc), Aleta Wondo General Hospital, Aleta Wondo, Ethiopia; College of Pharmacy (A H A Abd Al Magied MSc), Department of Mathematics and Sciences (A Abdelkader PhD), Department of Clinical Sciences (O E Hegazi BPharm), Center for Medical and Bio-Allied Health Sciences Research (S H Zyoud PhD), Ajman University, Ajman, United Arab Emirates; Department of Epidemiology (S Abd ElHafeez DrPH), Pediatric Dentistry and Dental Public Health Department (Prof O A A El Meligy PhD), Alexandria University, Alexandria, Egypt; Department of Small Animal Clinical Sciences (M Abdollahifar PhD), University of Saskatchewan, Saskatoon, SK, Canada; Department of Physiotherapy (A Abdullahi PhD, J S Usman PhD), Department of Community Medicine (Prof M A Gadanya MD), Bayero University Kano, Kano, Nigeria; Department of Physiotherapy (A Abdullahi PhD), Federal University Wukari, Wukari, Nigeria; Department of Family and Community Health (R G Aboagye MPH), Institute of Health Research (M Immurana PhD), Department of Microbiology and Immunology (Prof V N Orish PhD), University of Health and Allied Sciences, Ho, Ghana;

Department of Pediatric Dentistry (Prof L Abreu PhD), Federal University of Minas Gerais, Belo Horizonte, Brazil; Department of Neurology (S Abu Rumeileh MD), Martin Luther University Halle-Wittenberg, Halle (Saale), Germany; Department of Nursing (H Abualruz PhD), Al Zaytoonah University of Jordan, Amman, Jordan; Department of Pharmacology and Therapeutics (Prof S Aburuz PhD), College of Medicine and Health Sciences (J Nauman PhD), United Arab Emirates University, Al Ain, United Arab Emirates; College of Pharmacy (Prof S Aburuz PhD), University of Jordan, Amman, Jordan; Department of Biochemistry and Molecular Medicine (A Abu-Zaid PhD), College of Pharmacy (R M H Temsah PharmD), Alfaisal University, Riyadh, Saudi Arabia; College of Graduate Health Sciences (A Abu-Zaid PhD), Department of Ophthalmology (A Nabavi MD), University of Tennessee, Memphis, TN, USA; School of Medicine (I Y Addo PhD), University of Sydney, Sydney, NSW, Australia; Centre for Social Research in Health (I Y Addo PhD), School of Population Health (Prof B A Saddik PhD), University of New South Wales, Sydney, NSW, Australia; Department of Medical Rehabilitation (Prof R A Adedoyin PhD), Obafemi Awolowo University, Ile-Ife, Nigeria; Department of HIV and Infectious Diseases (A V Adepoju MD), Jhpiego, Abuja, Nigeria; Department of Adolescent Research and Care (A V Adepoju MD), Adolescent Friendly Research Initiative and Care, Ado Ekiti, Nigeria; Department of Life Sciences (M S Afzal PhD, Prof M Umair PhD), University of Management and Technology, Lahore, Pakistan; Department of Community Medicine (Prof S Afzal PhD), King Edward Memorial Hospital, Lahore, Pakistan; Department of Public Health (Prof S Afzal PhD), Public Health Institute, Lahore, Pakistan; College of Medicine (A Ahmad PhD), Shaqra University, Shaqra, Saudi Arabia; Department of Health and Biological Sciences (S Ahmad PhD), Abasyn University, Peshawar, Pakistan; Department of Natural Sciences (S Ahmad PhD), Gilbert and Rose-Marie Chagoury School of Medicine (Prof L Roever PhD), Lebanese American University, Beirut, Lebanon; School of Public Health (T Ahmad PhD), Zhejiang University, Hangzhou, China; Department of Epidemiology and Biostatistics (A Ahmadi PhD), Modeling in Health Research Center (A Mohammadian-Hafshejani PhD), Medical Plants Research Center (R Sadeghian PhD), Shahrekord University of Medical Sciences, Shahrekord, Iran; Department of Epidemiology (A Ahmadi PhD), Psychiatric Nursing and Management Department (F Ghadirian PhD), Social Determinants of Health Research Center (S Ghamari MD, A Kolahi MD, A Nikoobar BSc, M Rashidi MD), Obesity Research Center (A Haj-Mirzaian MD), Department of Neurosurgery (H Khayat Kashani MD), Department of Genetics (R Mirfakhraie PhD), Skull Base Research Center (I Mohammadzadeh MD), Student Research Committee (M Rahmanian MD), Department of Anesthesiology (S Salimi MD), Ophthalmic Research Center (ORC) (M Shayan MD), Shahid Beheshti University of Medical Sciences, Tehran, Iran; Department of Neuroscience (A Ahmadzade MD), Biotechnology Research Center (Prof A Sahebkar PhD), Mashhad University of Medical Sciences, Mashhad, Iran; Institute of Endemic Diseases (A Ahmed MSc), Unit of Basic Medical Sciences (E E Siddig MD), University of Khartoum, Khartoum, Sudan; Swiss Tropical and Public Health Institute (A Ahmed MSc), University of Basel, Basel, Switzerland; Department of Biosciences (H Ahmed PhD), COMSATS Institute of Information Technology, Islamabad, Pakistan; College of Nursing (M S Ahmed MSc), Majmaah University, Al Majmaah, Saudi Arabia; Department of Epidemiology (M B Ahmed PhD), Jimma University, Jimma, Ethiopia; College of Medicine and Public Health. (M B Ahmed PhD), College of Medicine and Public Health (G R Naik PhD), Department of Nursing and Health Sciences (S Shorofi PhD), Flinders University, Adelaide, SA, Australia; Department of Communicable Diseases (S Al Awaidy MSc), Centre of Studies and Research (S Jayapal PhD), Ministry of Health, Muscat, Oman; Middle East, Eurasia, and Africa Influenza Stakeholders Network, Muscat, Oman (S Al Awaidy MSc); Fundamentals and Administration Department (Prof O Al Omari PhD), Department of Geography (W Ali PhD), Sultan Qaboos University, Muscat, Oman; School of Medicine (Y Al-Ajlouni MD), New York Medical College,

Valhalla, NY, USA; Department of Epidemiology (Y Al-Ajlouni MD), Columbia University, New York, NY, USA; Department of Community and Mental Health (Prof M Albashtawy PhD), Al al-Bayt University, Mafraq, Jordan; Department of Neurology (B Al-Fatly MSc), Institute of Public Health (F Fischer PhD), Charité Universitätsmedizin Berlin (Charité Medical University Berlin), Berlin, Germany; Department of Bacteriology, Immunology, and Mycology (Prof A M Algammal PhD), Suez Canal University, Ismailia, Egypt; Department of Zoology (A Ali PhD), Abdul Wali Khan University Mardan, Mardan, Pakistan; Department of Medical Rehabilitation (Physiotherapy) (M U Ali PhD), University of Maiduguri, Maiduguri, Nigeria; Department of Rehabilitation Sciences (M U Ali PhD, J S Usman PhD), Hong Kong Polytechnic University, Hong Kong, China; Center for Biotechnology and Microbiology (S S Ali PhD), University of Swat, Swat, Pakistan; Institute of Health and Wellbeing (S M Alif PhD), Federation University Australia, Melbourne, VIC, Australia; School of Public Health and Preventive Medicine (S M Alif PhD), School of Psychological Sciences (N Parsons PhD), Monash University, Melbourne, VIC, Australia; Department of Medicine (J U Almazan PhD), Nazarbayev University, Astana, Kazakhstan; Department of Family and Community Medicine (N Z Alshahrani MD), University of Jeddah, Jeddah, Saudi Arabia; Institute of Molecular Biology and Biotechnology (A Altaf PhD, S Shahid PhD), University Institute of Public Health (S Nargus PhD), Research Centre for Health Sciences (RCHS) (S Shahid PhD), The University of Lahore, Lahore, Pakistan; Department of Rehabilitation Sciences (M Al-Wardat PhD), Department of Clinical Pharmacy (Prof K H Alzoubi PhD), Department of Public Health (Prof K A Kheirallah PhD), Jordan University of Science and Technology, Irbid, Jordan; Department of Medical Sciences (Prof Y M Al-Worafi PhD), Azal University for Human Development, Sana'a, Yemen; Department of Clinical Sciences (Prof Y M Al-Worafi PhD), University of Science and Technology of Fujairah, Fujairah, United Arab Emirates; Department of Pediatrics (Prof H Aly MD), Lerner Research Institute (X Liu PhD), Cleveland Clinic, Cleveland, OH, USA; Department of Pharmacy Practice and Pharmacotherapeutics (Prof K H Alzoubi PhD), Department of Basic Biomedical Sciences (Prof Y Bustanji PhD), Department of Clinical Sciences (Prof M M Ramadan PhD), College of Medicine (Prof B A Saddik PhD), Sharjah Institute of Medical Sciences (F Saheb Sharif-Askari PhD), Clinical Sciences Department (N Saheb Sharif-Askari PhD), College of Pharmacy (Prof M H Semreen PhD), Research Institute of Medical & Health Sciences (Prof M H Semreen PhD), University of Sharjah, Sharjah, United Arab Emirates; Spiritual Health Research Center (S Amiri PhD), Baqiyatallah University of Medical Sciences, Tehran, Iran; Faculty of Pharmacy (Prof R Ancuceanu PhD), Carol Davila University of Medicine and Pharmacy, Bucharest, Romania; Department of Child Neurology (D Angappan MD), Oregon Health and Science University, Portland, OR, USA; School of Pharmacy (M T Ansari PhD), University of Nottingham Malaysia, Semenyih, Malaysia; Regenerative Medicine, Organ Procurement and Transplantation Multi-disciplinary Center (S Anvari MD), Brachial Plexus and Peripheral Nerve Injury Center, Rasht, Iran (M Haghani Dogahe MD), Guilan University of Medical Sciences, Rasht, Iran; Rural Health Research Institute (A E Anyasodor PhD, S B Aychiluhm MPH), Charles Sturt University, Orange, NSW, Australia; Health Management and Economics Research Center (J Arabloo PhD), School of Medicine (M Bastan MD, N Eissazade MD), Department of Ophthalmology (H Hasani MD), Physiology Research Center (H Pazoki Toroudi PhD), Department of Physiology (H Pazoki Toroudi PhD), Iran University of Medical Sciences, Tehran, Iran (M Moradi MD); College of Pharmacy (M Arafat PhD), Al Ain University, Abu Dhabi, United Arab Emirates; College of Medicine and Health Sciences (B B Aregawi PhD), Department of Midwifery (M W Gebregergis MSc), Department of Medical Laboratory Sciences (H N Meles MSc), Adigrat University, Adigrat, Ethiopia; Department of Veterinary Pharmacology and Toxicology (A Aremu PhD), Department of Veterinary Physiology and Biochemistry (A Basiru PhD), University of Ilorin, Ilorin, Nigeria; Faculty of Nursing (M M W Atout PhD), Philadelphia

University, Amman, Jordan; Department of Forensic Medicine (A Atreya MD), Lumbini Medical College, Palpa, Nepal; Northumbria HealthCare NHS Foundation Trust, Newcastle upon Tyne, UK (A Aujayeb MBBS); Institute of Public Health (S B Aychiluhm MPH), Department of Internal Medicine (E Melese MD), School of Nursing (H B Netsere MSc), Department of Clinical Pharmacy (A K Sendekie MSc), University of Gondar, Gondar, Ethiopia; Institute of Biotechnology and Genetic Engineering (S Aziz MS), The University of Agriculture, Peshawar, Pakistan; ASIDE Healthcare, Lewes, DE, USA (A Azzam MD); Faculty of Medicine (A Azzam MD), October 6 University, 6th of October City, Egypt; Department of Forensic Science (A D Badiye PhD, N Kapoor PhD), Government Institute of Forensic Science Nagpur, Nagpur, India; Rashtrasant Tukadoji Maharaj Nagpur University, Nagpur, India (A D Badiye PhD); School of Public Affairs (R Bai MD), Nanjing University of Information Science and Technology, Nanjing, China; International Medical School (A A Baig PhD), Management and Science University, Alam, Malaysia; Department of Forensic Medicine and Toxicology (S M Bakkannavar MD), Kasturba Medical College, Mangalore (R Holla MD, Prof B Unnikrishnan MD), Department of Pharmaceutical Regulatory Affairs and Management (V S Ligade PhD), Manipal Academy of Higher Education, Manipal, India (H L Dsouza MD); Nuffield Department of Surgical Sciences (S Bandyopadhyay MPH), Department of Psychiatry (Prof C R J Newton MD), University of Oxford, Oxford, UK; Department of Neurosurgery (S Bandyopadhyay MPH), School of Psychology (Prof S Cortese PhD), University of Southampton, Southampton, UK; Department of Pharmacology (Prof I Banerjee MD), Sir Seewoosagur Ramgoolam Medical College, Belle Rive, Mauritius; Miller School of Medicine (M Bardhan MD), University of Miami, Miami, FL, USA; School of Psychology (Prof S L Barker-Collo PhD), School of Pharmacy (K A Beyene PhD), University of Auckland, Auckland, New Zealand; Department of Public and Environmental Health (A Barrow MPH), University of The Gambia, Banjul, The Gambia; Department of Epidemiology (A Barrow MPH), College of Medicine (M J Diaz BS, K T Root BS), University of Florida, Gainesville, FL, USA; Alpha Genomics Private Limited, Islamabad, Pakistan (Z Basharat PhD); Health Information Management (A Bashiri PhD), School of Medicine (M Farjoud Kouhanjani MD), Epilepsy Research Center (M Farjoud Kouhanjani MD), Shiraz University of Medical Sciences, Shiraz, Iran; Non-communicable Diseases Research Center (M Bastan MD, S Ghamari MD, M Rashidi MD), Department of Pediatric Neurology (M Bemanalizadeh MD), Iranian Research Center for HIV/AIDS (IRCHA) (O Dadras PhD), School of Medicine (M Gouravani MD, A Shahbandi MD), Sina Trauma and Surgery Research Center (M Hassan Zadeh Tabatabaei MD, M Khormali MD), Digestive Diseases Research Institute (V Mansouri MD), Department of Neurosurgery (A Pour-Rashidi MD), Sina Hospital (A Sharifan PharmD), Tehran University of Medical Sciences, Tehran, Iran; Cooper University Hospital (S Batchu MD), Cooper University Hospital, Camden, NJ, USA; Avicenna Biotech Research, Germantown, MD, USA (B Behnam MD); Department of Regulatory Affairs (B Behnam MD), Amarex Clinical Research, Germantown, MD, USA; Transplant and Hepatobiliary Surgery Service (D F Bejarano Ramirez MSc), Hospital Universitorio Fundación Santa Fe de Bogotá, Bogota, Colombia; Subdirectorate of Clinical Studies and Clinical Epidemiology (D F Bejarano Ramirez MSc), Hospital Universitorio Fundación Santa Fe de Bogotá, Bogotá, Colombia; Department of Pediatrics (M Bemanalizadeh MD), Isfahan University of Medical Sciences, Isfahan, Iran; Department of Pharmaceutical and Administrative Sciences (K A Beyene PhD), University of Health Sciences and Pharmacy in St. Louis, St Louis, MO, USA; Department of Forensic Chemistry (D S Bhagat PhD), Government Institute of Forensic Science, Aurangabad, Aurangabad, India; Department of Public Health (A S Bhagavathula PhD), North Dakota State University, Fargo, ND, USA; Division of Gastroenterology and Hepatology (A S Bhagavathula PhD), Mayo Clinic, Jacksonville, FL, USA; Global Health Neurology Lab (S Bhaskar MD), NSW Brain Clot Bank, Sydney, NSW, Australia; Division of Cerebrovascular Medicine and

Neurology (S Bhaskar MD), National Cerebral and Cardiovascular Center, Suita, Japan; Department of General Medicine (A N Bhat MD), Department of Internal Medicine (A Boloor MD, M M R Reddy MD), Department of Community Medicine (N Joseph MD, R Motappa MD), Department of Forensic Medicine and Toxicology (Prof J Padubidri MD, P H Shetty MD), Manipal College of Dental Sciences, Mangalore (Prof P K Shetty MDS), Manipal Academy of Higher Education, Mangalore, India; Department of Medical Lab Technology (Prof G K Bhatti PhD), Chandigarh University, Mohali, India; Department of Human Genetics and Molecular Medicine (Prof J S Bhatti PhD), Central University of Punjab, Bathinda, India; Department of Pharmacy (Prof M A Bhuiyan PhD), University of Asia Pacific, Dhaka, Bangladesh; Department of Health Administration (S S Bhuyan PhD), Rutgers University, New Brunswick, NJ, USA; Department of Radiology (C Bilgin MD), Neurovascular Research Laboratory (C Bilgin MD), Mayo Clinic College of Medicine, Rochester, MN, USA; Department of Biomedical and NeuroMotor Sciences (Prof F Bisulli PhD), Department of Biomedical and Neuromotor Sciences (L Muccioli MD), University of Bologna, Bologna, Italy; UOC Clinica Neurologica (Prof F Bisulli PhD), IRCCS Istituto delle Scienze Neurologiche di Bologna (Institute of Neurological Sciences of Bologna), Bologna, Italy; Department of Anesthesia and Critical Care Medicine (S Boppana MD), Russell H. Morgan Department of Radiology and Radiological Science (A Kamireddy MD), Department of Neurosurgery (F Kazemi MD), Johns Hopkins University, Baltimore, MD, USA (E Melese MD); Department of Medicine (Prof S Bouaoud DrPH), Faculty of Medicine (Prof A Ouyahia PhD), University Ferhat Abbas of Setif, Setif, Algeria; Department of Epidemiology and Preventive Medicine (Prof S Bouaoud DrPH), University Hospital Saadna Abdenour, Setif, Algeria; School of Pharmacy (Prof Y Bustanji PhD), The University of Jordan, Amman, Jordan; Faculty of Health Sciences (M Çakmak Barsbay PhD), Ankara University, Ankara, Turkiye; Research Unit on Applied Molecular Biosciences (UCIBIO), Faculty of Pharmacy (Prof F Carvalho PhD), Institute for Research and Innovation in Health (i3S) (Prof N Cruz-Martins PhD), University of Porto, Porto, Portugal; Department of Psychiatry (Prof J Castaldelli-Maia PhD, Prof M F P Peres MD), University of São Paulo, São Paulo, Brazil; Department of Clinical Nutrition (R M Chandika PhD), Department of Public Health, College of Nursing and Health Sciences (S Dohare MD), Epidemiology Program, Department of Public Health, CNHS (M Khan MD), Substance Abuse and Toxicology Research Center (S Mohan PhD), Department of Public Health (P Rajpoot PhD), Jazan University, Jazan, Saudi Arabia; Temerty Faculty of Medicine (V Chattu MD), Division of Neurology (S Fereshtehnejad PhD), Institute of Medical Science (U Saeed MSc), University of Toronto, Toronto, ON, Canada; Department of Community Medicine (V Chattu MD), Datta Meghe Institute of Medical Sciences, Sawangi, India; Department of Biology (A A Chaudhary PhD), Al-Imam Mohammad Ibn Saud Islamic University, Riyadh, Saudi Arabia; Division of Infectious Diseases (P R Ching MD), Virginia Commonwealth University, Richmond, VA, USA; Centre for Research Impact & Outcome (H Chopra PhD), Chitkara University, Rajpura, India; The Interdisciplinary Research Group on Biomedicine and Health (D Chu PhD), Faculty of Applied Sciences (D Chu PhD), VNU International School (VNUIS), Hanoi, Vietnam; Department of Pediatrics (H Chu PhD), Peking University, Beijing, China; Department of Child and Adolescent Psychiatry (Prof S Cortese PhD), Institute for Excellence in Health Equity (M Kumar PhD), New York University, New York, NY, USA; Research Center on Public Health (CESP), School of Medicine and Surgery (P Cortesi PhD), Center for Public Health Research (P Ferrara PhD), University of Milan Bicocca, Monza, Italy; Department of Diagnostic and Therapeutic Technologies (Prof N Cruz-Martins PhD), Cooperativa de Ensino Superior Politécnico e Universitário (Polytechnic and University Higher Education Cooperative), Vila Nova de Famalicão, Portugal; Research Center for Child Psychiatry (O Dadras PhD), Heart Center (V Kytö MD), University of Turku, Turku, Finland; Department of Medical and Surgical Sciences and Advanced Technologies "GF

Ingrassia" (Prof E D'Amico MD), University of Catania, Catania, Italy; Public Health Foundation of India, Gurugram, India (Prof L Dandona MD, Prof R Dandona PhD, G Kumar PhD, A Pandey PhD); Department of Public Health (S D Darcho MPH), Department of Psychiatry (M T Walde MSc), Haramaya University, Harar, Ethiopia; Department of Pediatrics (A H Darwish MD), Tanta University, Tanta, Egypt; Research and Development Cell (A S Dhane MBA), Department of Oral Pathology and Microbiology (Prof G S Sarode PhD, Prof S C Sarode PhD), Dr. D. Y. Patil Vidyapeeth, Pune (Deemed to be University), Pune, India; University of South Carolina, Columbia, SC, USA (V R Dhulipala MD); Department of Medicine (T C Do MD), Pham Ngoc Thach University of Medicine, Ho Chi Minh City, Vietnam; Independent Consultant, South Plainfield, NJ, USA (O P Doshi MS); Department of Forensic Medicine and Toxicology (H L Dsouza MD), Kasturba Medical College, Mangalore, Mangalore, India; Department of Conservative Dentistry with Endodontics (A M Dziedzic DSc), Medical University of Silesia, Katowice, Poland; Department of Orthopaedic Surgery (A Ebrahimi MD), Department of Radiology (A Haj-Mirzaian MD, X Liu PhD), Massachusetts General Hospital, Boston, MA, USA; Faculty of Science and Health (M Ekholuenetale PhD), University of Portsmouth, Hampshire, UK; Almoosa College of Health Sciences, Al Ahsa, Saudi Arabia (R A El Arab PhD); Department of Public Health and Community Medicine (Prof I F El Bayoumy DrPH), Tanta University, Tanta city, Egypt; School of Public Health (Prof I F El Bayoumy DrPH), Texila American University, Guyana, Guyana; Department of Pediatric Dentistry (Prof O A A El Meligy PhD), Rabigh Faculty of Medicine (Prof A Malik PhD), Department of Dental Public Health (Z S Natto DrPH), King Abdulaziz University, Jeddah, Saudi Arabia; Department of Neurophysiology (Prof H R Elhabashy MD), Department of Neurology (Prof A Hassan MD), Cairo University, Cairo, Egypt; Faculty of Medicine (M Elhadi MD), University of Tripoli, Tripoli, Libya; Houston Methodist Hospital, Houston, TX, USA (M Elhadi MD); Department of Pediatrics (C Eltaha MD), University of Texas, Dallas, TX, USA; Department of Epidemiology and Medical Statistics (A F Fagbamigbe PhD), Department of Health Promotion and Education (S Ibitoye PhD), Department of Medicine (O V Olalusi MD, Prof M O Owolabi DrM), University of Ibadan, Ibadan, Nigeria; Research Centre for Healthcare and Community (A F Fagbamigbe PhD), Coventry University, Coventry, UK; Department of Oral Biology (A Fahim PhD), Riphah International University, Islamabad, Pakistan (Z Z Piracha PhD); Department of Neurological Surgery (J Fares MD), Neurological Surgery (N A Shlobin BA), Department of Preventive Medicine (M Teramoto MD), Northwestern University, Chicago, IL, USA; Satcher Health Leadership Institute (A O Fasanmi PhD), Morehouse School of Medicine, Atlanta, GA, USA; School of Medicine (A O Fasanmi PhD), Department of Family and Preventive Medicine (S Thirunavukkarasu PhD), Emory University, Atlanta, GA, USA; School of Engineering (A Fatehizadeh PhD), Edith Cowan University, Joondalup, WA, Australia; Department of Biology and Medicine (P Fazeli MSc), Brown University, Providence, RI, USA; Laboratory of Experimental Medicine (T Fazylov MD), Research and Publication Activity Division (M Kulimbet MSc), Science Department (A Shamsutdinova MD), Kazakh National Medical University, Almaty, Kazakhstan; Department of Infectious Diseases and Public Health (G Fekadu PhD), Department of Biomedical Sciences (A Waris MS), City University of Hong Kong, Hong Kong, China; Department of Pharmacy (G Fekadu PhD), Department of Nursing (G Fetensa MSc), Department of Public Health (M T Yilma MPH), Wollega University, Nekemte, Ethiopia; Department of Neurobiology, Care Sciences, and Society (S Fereshtehnejad PhD), Karolinska Institute, Stockholm, Sweden; Laboratory of Public Health (P Ferrara PhD), IRCCS Istituto Auxologico Italiano, Milan, Italy; Department of Social Sciences (Prof N Ferreira PhD, Prof M J M Sullman PhD), Department of Life and Health Sciences (Prof M J M Sullman PhD), University of Nicosia, Nicosia, Cyprus; Department of Neuroscience (M Foschi MD), Multiple Sclerosis Research Center, Ravenna, Italy; Department of Biotechnological and Applied Clincial Sciences (M Foschi MD),

University of L'Aquila, L'Aquila, Italy; Department of Community Medicine (Prof M A Gadanya MD), Aminu Kano Teaching Hospital, Kano, Nigeria; Department of Food Technology (Y Galali ResM), Salahaddin University-Erbil, Erbil, Iraq; Department of Nutrition and Dietetics (Y Galali ResM), Cihan University-Erbil, Erbil, Iraq; Institute of Health and Wellbeing (B Ganesan PhD), Federation University Australia, Churchill, VIC, Australia; Department of Biostatistics (Prof X Gao PhD), Key Lab of Environment and Health (Prof X Gao PhD), Xuzhou Medical University, Xuzhou, China; Department of Neurology (Prof R Garg MD, V Suresh MBBS), King George's Medical University, Lucknow, India; Department of Medicine (J A Gilani MD), Aga Khan University, Karachi, Pakistan; Department of Nursing (A A Girmay MSc), Aksum University, Aksum, Ethiopia; Laboratory of Neurological Disorders (G Giussani PhD), Mario Negri Institute for Pharmacological Research, Milan, Italy; Department of Health Systems and Policy Research (Prof M Golechha PhD), Indian Institute of Public Health, Gandhinagar, India; Department of Dermatology (A Grada MD), Department of Quantitative Health Science (X Liu PhD), Case Western Reserve University, Cleveland, OH, USA; Department of Epidemiology and Biostatistics (S Guan MD), Anhui Medical University, Hefei, China; Department of Toxicology (S Gupta MSc), Shriram Institute for Industrial Research, Delhi, India; Department of Biochemistry (Prof N M Hamdy PhD), Department of Entomology (A M Samy PhD), Medical Ain Shams Research Institute (MASRI) (A M Samy PhD), Ain Shams University, Cairo, Egypt; Faculty of Medicine (N I Harlianto MD), Utrecht University, Utrecht, Netherlands; Department of Radiology (N I Harlianto MD), University Medical Center Utrecht, Utrecht, Netherlands; Department of Zoology and Entomology (A I Hasaballah PhD, M G M Zeariya PhD), Al-Azhar University, Cairo, Egypt; Public Health Department (I I Hassan PhD), Dalhatu Araf Specialist Hospital, Lafia, Nigeria; Department of Public Health (I I Hassan PhD), Federal University of Lafia, Lafia, Nigeria; Independent Consultant, Santa Clara, CA, USA (G Heidari MD); Department of Medicine (M Hemmati MD), MedStar Health, Washington, DC, USA; Department of Medicine (M Hemmati MD, C J Sabet MA), Georgetown University, Washington, DC, USA; Department of Microbiology (K Hezam PhD), Taiz University, Taiz, Yemen; School of Medicine (K Hezam PhD), Nankai University, Tianjin, China; School of Dentistry (N Hoan DDS), Hanoi Medical University, Hanoi, Vietnam; School of Computer Science (Prof M Hosseinzadeh PhD), Faculty of Medicine (H T H Nguyen MD), Institute for Research and Training in Medicine, Biology and Pharmacy (H T H Nguyen MD), Duy Tan University, Da Nang, Vietnam; Jadara University Research Center (Prof M Hosseinzadeh PhD), Jadara University, Irbid, Jordan; Faculty of Medicine (J Huang MD), Jockey Club School of Public Health and Primary Care (C Zhong PhD), The Chinese University of Hong Kong, Hong Kong, China; International Master Program for Translational Science (H Huynh BS), Taipei Medical University, Taipei, Taiwan; Department of Occupational Safety and Health (Prof B Hwang PhD), China Medical University, Taiwan, Taichung, Taiwan; Department of Occupational Therapy (Prof B Hwang PhD), Asia University, Taiwan, Taichung, Taiwan; Collaborative Alliance Research and Education (CARE) Programme (A Ikiroma PhD), Episcope Research Service, Aberdeen, Scotland; West Africa RCC (O S Ilesanmi PhD), Africa Centre for Disease Control and Prevention, Abuja, Nigeria; Department of Community Medicine (O S Ilesanmi PhD), Department of Neurology (O V Olalusi MD), Department of Medicine (Prof M O Owolabi DrM), Department of Oral and Maxillofacial Surgery (A A Salami BDS), University College Hospital, Ibadan, Ibadan, Nigeria; Faculty of Medicine (I M Ilic PhD), University of Belgrade, Belgrade, Serbia; Faculty of Medical Sciences (Prof M D Ilic PhD), University of Kragujevac, Kragujevac, Serbia; Department of Clinical Pharmacy (M Imam PhD), Prince Sattam bin Abdulaziz University, Al Kharj, Saudi Arabia; Faculty of Health and Life Sciences (A Inok PhD), University of Exeter, Exeter, UK; School of Pharmacy (M Islam PhD), BRAC University, Dhaka, Bangladesh; Department of Physical and Medicine (L Jacob MD), Université Paris Cité, Paris, France;

Research and Development Unit (L Jacob MD), Biomedical Research Networking Center for Mental Health Network (CiberSAM), Barcelona, Spain; Department of Immunology (Prof A Jafarzadeh PhD), Kerman University of Medical Sciences, Kerman, Iran; Department of Immunology (Prof A Jafarzadeh PhD), Department of Epidemiology and Biostatistics (Prof M Rezaeian PhD), Department of Neurology (A Vakilian MD), Non-communicable Diseases Research Center (A Vakilian MD), Rafsanjan University of Medical Sciences, Rafsanjan, Iran; College of Medicine and Medical Sciences (H Jahrami PhD), Arabian Gulf University, Manama, Bahrain; Department of Psychiatry (Z Saif MBA), Ministry of Health, Manama, Bahrain (H Jahrami PhD); Department of Health and Safety (A A Jairoun PhD), Dubai Municipality, Dubai, United Arab Emirates; The World Academy of Sciences UNESCO, Trieste, Italy (Prof M Jakovljevic PhD); Shaanxi University of Technology, Hanzhong, China (Prof M Jakovljevic PhD); Department of Environmental Engineering (Prof R Jalilzadeh Yengejeh PhD), Islamic Azad University, Ahvaz, Iran; Department of Neurosciences (Prof R G Jamora PhD), University of the Philippines Manila, Manila, Philippines; Institute for Neurosciences (Prof R G Jamora PhD), St. Luke's Medical Center, Bonifacio Global City, Philippines; Department of Pharmacology (T Jawaid PhD), Imam Mohammad Ibn Saud Islamic University, Riyadh, Saudi Arabia; Department of Public Health (Z Ji MMed), Tongji University, Shanghai, China; Rothschild Foundation Hospital (Prof J B Jonas MD), Institut Français de Myopie, Paris, France; Singapore Eye Research Institute (Prof J B Jonas MD), Singapore Eye Research Institute, Singapore, Singapore; Department of Economics (C E Joshua BSc), National Open University, Benin City, Nigeria; School of Public Health (Z Kabir PhD), University College Cork, Cork, Ireland; Faculty of Dentistry (K K Kanmodi MPH, A A Salami BDS), University of Puthisastra, Phnom Penh, Cambodia; Office of the Executive Director (K K Kanmodi MPH), Cephas Health Research Initiative Inc, Ibadan, Nigeria; Department of Physical Therapy and Health Rehabilitation (F Z Kashoo MSc), Majmaah University, Majmaah, Saudi Arabia; Public Health Foundation of India, New Delhi, India (H Kaur MPH); Amity Institute of Forensic Sciences (H Khajuria PhD, B P Nayak PhD), Amity University, Noida, India; Department of Biostatistics (Prof A Khalilian PhD), Department of Medical-Surgical Nursing (S Shorofi PhD), Mazandaran University of Medical Sciences, Sari, Iran; Faculty of Nursing (H Khatatbeh PhD), Yarmouk University, Irbid, Jordan; Department of Biochemistry (F Khidri PhD), Liaquat University Of Medical and Health Sciences, Jamshoro, Pakistan; Department of Internal Medicine (A A Khosla MD), Corewell Health East William Beaumont University Hospital, Royal Oak, MI, USA; Department of Medical Oncology (A A Khosla MD), Miami Cancer Institute, Miami, FL, USA; Department of Public Health (J Khubchandani PhD), New Mexico State University, Las Cruces, NM, USA; School of Traditional Chinese Medicine (Y Kim PhD), Xiamen University Malaysia, Sepang, Malaysia; Department of Medicine (Y Kim BS), Yonsei University, Seoul, South Korea; Millennium Prevention, Inc., Westwood, MA, USA (R W Kimokoti MD); Department of Neurology (H Koh PhD), School of Medicine (Prof J A Singh MD), Baylor College of Medicine, Houston, TX, USA; Department of Epidemiology (Prof K Kostev PhD), IQVIA, Frankfurt am Main, Germany; University Hospital Marburg, Marburg, Germany (Prof K Kostev PhD); Department of Anthropology (Prof K Krishan PhD), Institute of Forensic Science & Criminology (V Sharma PhD), Panjab University, Chandigarh, India; Department of Anesthesiology (V Krishnamoorthy MD), Duke University, Durham, NC, USA; Department of Neuroscience (Prof J Kruja PhD), University of Medicine, Tirana, Albania; Department of Neuroscience (Prof J Kruja PhD), Medical Sciences University Hospital, Tirana, Albania; Department of Biochemistry (Prof M Kuddus PhD), Department of Public Health (M G M Zeariya PhD), University of Hail, Hail, Saudi Arabia; Center of Medicine and Public Health (M Kulimbet MSc), Director of Central Asia Research Collaboration Group (Prof F Rahim PhD), Asfendiyarov Kazakh National Medical University, Almaty, Kazakhstan; Department of Psychiatry (M Kumar PhD), University

of Nairobi, Nairobi, Kenya; Public Health, School of Medicine and Dentistry (S Kundu MPH), Griffith University, Gold Coast, QLD, Australia; Clinical Research Center (V Kytö MD), Turku University Hospital, Turku, Finland; Integrated Department of Epidemiology, Health Policy, Preventive Medicine and Pediatrics (Prof C Lahariya MD), Foundation for People-centric Health Systems, New Delhi, India; Centre for Health: The Specialty Practice, New Delhi, India (Prof C Lahariya MD); Indian Council of Medical Research, New Delhi, India (D K Lal MD); Health Services Management Training Centre (J Lám PhD), Semmelweis University, Budapest, Hungary; NEVES Society for Patient Safety, Budapest, Hungary (J Lám PhD); Unidad de Genética y Salud Pública (Prof I Landires MD), Instituto de Ciencias Médicas, Las Tablas, Panama; Ministry of Health (Prof I Landires MD), Hospital Joaquín Pablo Franco Sayas, Las Tablas, Panama; Department of Health Sciences (DISSAL) (F Lanfranchi MD), University of Genoa, Genoa, Italy; Faculty of Medicine (N Le MD), Department of Internal Medicine (T H Tran MD), University of Medicine and Pharmacy at Ho Chi Minh City, Ho Chi Minh City, Vietnam; Department of Cardiovascular Research (N Le MD), Methodist Hospital, Merrillville, IN, USA; Department of Precision Medicine (Prof S Lee MD), Sungkyunkwan University, Suwon-si, South Korea; UCD Centre for Disability Studies (C Linehan PhD), University College Dublin, Dublin, Ireland; Department of Radiology and Biomedical Imaging (X Liu PhD), Department of Genetics (S Pawar PhD), Department of Psychiatry (T Rhee PhD), Yale University, New Haven, CT, USA; One Health Research Group (J López-Gil PhD), One Health Global Research Group (Prof E Ortiz-Prado PhD), Universidad de las Americas (University of the Americas), Quito, Ecuador; School of Medicine (Prof G Lucchetti PhD), Federal University of Juiz de Fora, Juiz de Fora, Brazil; Department of Primary Care and Public Health (Prof A Majeed MD, Prof S Rawaf MD, C Tabche MSc), Imperial College London, London, UK; Rama Medical College Hospital and Research Centre, Uttar Pradesh, India (K Malhotra MBBS); Institute of Applied Health Research (K Malhotra MBBS), University of Birmingham, Birmingham, UK; Department of Biomedical Engineering (H Marateb PhD), University of Isfahan, Isfahan, Iran; Biomedical Engineering Research Center (CREB) (H Marateb PhD), Universitat Politècnica de Catalunya (Barcelona Tech - UPC), Barcelona, Spain; Department of Nutrition and Dietetics (M Martorell PhD), Centre for Healthy Living (M Martorell PhD), University of Concepción, Concepción, Chile; Faculty of Humanities and Health Sciences (Prof R R Marzo MD), Curtin University, Sarawak, Malaysia; Jeffrey Cheah School of Medicine and Health Sciences (Prof R R Marzo MD), Monash University, Subang Jaya, Malaysia; Department of Anatomy and Developmental Biology (Y Mathangasinghe PhD), Monash University, Clayton, VIC, Australia; Department of Anatomy, Genetics and Biomedical Informatics (Y Mathangasinghe PhD), University of Colombo, Colombo, Sri Lanka; Division of Pediatric Hospital Medicine (R P Mediratta MD), Stanford University, Palo Alto, CA, USA; Neurology Department (Prof M Mehndiratta MD), Janakpuri Super Specialty Hospital Society, New Delhi, India; Department of Neurology (Prof M Mehndiratta MD), Govind Ballabh Institute of Medical Education and Research, New Delhi, India; Center for Translation Research and Implementation Science (G A Mensah MD), National Institutes of Health, Bethesda, MD, USA; Department of Medicine (G A Mensah MD), University of Cape Town, Cape Town, South Africa; General Administration Department (A Meretoja MD), Department of Neurosurgery (I Rautalin PhD), Helsinki University Hospital, Helsinki, Finland; School of Health Sciences (A Meretoja MD), University of Melbourne, Melbourne, VIC, Australia; University Centre Varazdin (T Mestrovic PhD), University North, Varazdin, Croatia; Department of Paediatrics (Prof S Mettananda DPhil), University of Kelaniya, Ragama, Sri Lanka; University Paediatrics Unit (Prof S Mettananda DPhil), Colombo North Teaching Hospital, Ragama, Sri Lanka; Multidisciplinary Department of Medical-Surgical and Dental Specialties (G Minervini PhD), University of Campania Luigi Vanvitelli, Naples, Italy; Saveetha Dental College and Hospitals (G Minervini PhD, S Selvaraj PhD, M Tovani-Palone PhD), Centre of

Molecular Medicine and Diagnostics (COMManD) (Prof S Patil PhD), Center for Global Health Research (Prof A Sahebkar PhD), Saveetha University, Chennai, India; Department of Hospital Administration (M Mirza MD), Department of Radiodiagnosis (P Singh MD), All India Institute of Medical Sciences, Bathinda, India; National Data Management Center for Health (A Misganaw PhD), Ethiopian Public Health Institute, Addis Ababa, Ethiopia; Department of Pharmacology (A K Misra MD), All India Institute of Medical Sciences, Mangalagiri, India; Center for Brain and Health (A Z Mohamed PhD), New York University Abu Dhabi, Abu Dhabi, United Arab Emirates; Molecular Biology Unit (N S Mohamed MSc), Bio-Statistical and Molecular Biology Department (N S Mohamed MSc), Sirius Training and Research Centre, Khartoum, Sudan; School of Health Sciences (S Mohan PhD), University of Petroleum and Energy Studies, Dehradun, India; Clinical Epidemiology and Public Health Research Unit (L Monasta DSc), Burlo Garofolo Institute for Maternal and Child Health, Trieste, Italy; Faculty of Medicine (A Moodi Ghalibaf MD, A Rajabpour Sanati MD), Birjand University of Medical Sciences, Birjand, Iran; Department of Surgery (F Mulita PhD), General University Hospital of Patras, Patras, Greece; Faculty of Medicine (F Mulita PhD), Department of Emergency Medicine (Prof I Pantazopoulos PhD), University of Thessaly, Larissa, Greece; Department of Community and Global Health (Y Munkhsaikhan MD), The University of Tokyo, Tokyo, Japan; Clinical Epidemiology Research Unit (E Murillo-Zamora PhD), Mexican Institute of Social Security, Villa de Alvarez, Mexico; Postgraduate in Medical Sciences (E Murillo-Zamora PhD), Universidad de Colima, Colima, Mexico; Department of Research Methods (S Muthu PhD), Orthopaedic Research Group, Coimbatore, India; Department of Biotechnology (S Muthu PhD), Karpagam Academy of Higher Education (Deemed to be University), Coimbatore, India; Department of Engineering (G R Naik PhD), Western Sydney University, Sydney, NSW, Australia; Nursing & Midwifery Research Department (NMRD) (A J Nashwan PhD), Department of Geriatric and Long Term Care (B Sathian PhD), Hamad Medical Corporation, Doha, Qatar; Department of Health Policy and Oral Epidemiology (Z S Natto DrPH), Department of Ophthalmology (M Shayan MD), Harvard University, Boston, MA, USA; Department of Circulation and Medical Imaging (J Nauman PhD), Norwegian University of Science and Technology, Trondheim, Norway; Department of Biotechnology (M Naveed PhD), University of Central Punjab, Lahore, Pakistan; Department of Health Promotion (A Nazri-Panjaki MSc), Zahedan University of Medical Sciences, Zahedan, Iran; Department of General Medicine (G Nepal MD), Rani Primary Healthcare Centre, Biratnagar, Nepal; College of Medicine and Health Sciences (H B Netsere MSc), Department of Psychiatry (M Tareke MSc), Department of Pharmacology (Y Yismaw MSc), Bahir Dar University, Bahir Dar, Ethiopia; International Islamic University Islamabad, Islamabad, Pakistan (R K Niazi PhD); School of Health (M Nozari PhD), Bam University of Medical Sciences, Bam, Iran; Department of Paediatrics (C A Nri-Ezedi PhD), Nnamdi Azikiwe University, Awka, Nigeria; Department of Pediatrics (V E Nwatah MD), National Hospital Abuja, Abuja, Nigeria; Department of International Public Health (V E Nwatah MD), University of Liverpool, Liverpool, UK; Department of Physiology (O J Nzoputam PhD), University of Benin, Edo, Nigeria; Department of Physiology (O J Nzoputam PhD), Benson Idahosa University, Benin City, Nigeria; Department of Applied Economics and Quantitative Analysis (Prof B Oancea PhD), University of Bucharest, Bucharest, Romania; Bioinformatics Department (Prof B Oancea PhD), National Institute of Research and Development for Biological Sciences, Bucharest, Romania; Department of Psychiatry and Behavioural Neurosciences (Prof A T Olagunju PhD), McMaster University, Hamilton, ON, Canada; Department of Psychiatry (Prof A T Olagunju PhD), University of Lagos, Lagos, Nigeria; Diplomacy and Public Relations Department (A Omar Bali PhD), University of Human Development, Sulaymaniyah, Iraq; Department of Pharmacotherapy and Pharmaceutical Care (M Ordak PhD), Department of Biochemistry and Pharmacogenomics (M Zielińska MPharm), Medical University of

Warsaw, Warsaw, Poland; Sickle Cell Unit (Prof V N Orish PhD), Ho Teaching Hospital, Ho, Ghana; Laboratory of Public Health Indicators Analysis and Health Digitalization (N Otstavnov BA), Moscow Institute of Physics and Technology, Dolgoprudny, Russia; Division of Infectious Diseases (Prof A Ouyahia PhD), University Hospital of Setif, Setif, Algeria; National School of Public Health (A Padron-Monedero PhD), Institute of Health Carlos III, Madrid, Spain; Centre for Biotechnology (S K Panda PhD), Siksha 'O' Anusandhan (Deemed to be University), Bhubaneswar, India; Department of Ophthalmology (S Panda-Jonas MD), Heidelberg University, Heidelberg, Germany; Amity Institute of Biotechnology (Prof D Pande Katare PhD), Amity University Uttar Pradesh, Noida, India; Department of Neurology (L D Panos MD), Department of Emergency Medicine (Prof I Pantazopoulos PhD), University of Bern, Bern, Switzerland; Department of Neurology (L D Panos MD), University of Cyprus, Nicosia, Cyprus; Department of Science and Mathematics (Prof P Papadopoulou PhD), Deree-The American College of Greece, Athens, Greece; Department of Biophysics (Prof P Papadopoulou PhD), University of Athens, Athens, Greece; Department of Forensic Medicine and Toxicology (U Parekh MD), All India Institute of Medical Sciences, Rajkot, India; Department of Epidemiology and Community Health (R R Parikh MD), University of Minnesota, Minneapolis, MN, USA; Department of Medical Sciences (R Passera PhD), University of Torino, Torino, Italy; Department of Imaging (R Passera PhD), AOU Città della Salute e della Scienza di Torino, Torino, Italy; College of Dental Medicine (Prof S Patil PhD), Roseman University of Health Sciences, South Jordan, UT, USA; Department of Biomedical Sciences (U Pensato MD), Humanitas University, Pieve Emanuele (MI), Italy; Australian Institute of Health Innovation (P Peprah MSc), Macquarie University, Sydney, NSW, Australia; International Institute for Educational Planning (IIEP) (Prof M F P Peres MD), Albert Einstein Hospital, São Paulo, Brazil; Department of Food, Environmental and Nutritional Sciences (Prof S Perna PhD), University of Milan, Milano, Italy; Department of Internal Medicine (H Pham MD), University of Arizona, Tucson, AZ, USA; Department of Cardiovascular Medicine (H Pham MD), Mayo Clinic, Rochester, MN, USA; International Center of Medical Sciences Research (Z Z Piracha PhD), International Center of Medical Sciences Research, Islamabad, Pakistan; College of Health Sciences (CHS) (Prof D Poddighe PhD), VinUniversity, Hanoi, Vietnam; Clinical Academic Department of Pediatrics (Prof D Poddighe PhD), University Medical Center (UMC), Astana, Kazakhstan; Department of Data Management and Analysis (R Poluru PhD), The International Clinical Epidemiology Network (INCLEN) Trust International, New Delhi, India; Department of Humanities and Social Sciences (Prof J Pradhan PhD), National Institute of Technology Rourkela, Rourkela, India; Department of Clinical Research and Epidemiology (M Prasad MD), Institute of Liver and Biliary Sciences, New Delhi, India; Health Sciences Department (D R A Pribadi MSc), Muhammadiyah University of Surakarta, Sukoharjo, Indonesia; Department of Biostatistics, Epidemiology, and Informatics (J Puvvula PhD), University of Pennsylvania, Philadelphia, PA, USA; Cihan University-Sulaimaniya Research Center (N H Qasim DSc), Cihan University-Sulaimaniya, Sulaymaniyah, Iraq; Department of Medical Oncology (Prof V Radhakrishnan MD), Cancer Institute (W.I.A), Chennai, India; Department of Community Medicine and Family Medicine (Prof P Raghav MD), Department of Pharmacology (M Shamim MBBS, S Singh MD, K Tiwari MBBS), All India Institute of Medical Sciences, Jodhpur, India; Osh State University, Osh, Kyrgyzstan (Prof F Rahim PhD); Department of Population Science and Human Resource Development (Prof M Rahman DrPH), University of Rajshahi, Rajshahi, Bangladesh; Future Technology Research Center (A Rahmani PhD), National Yunlin University of Science and Technology, Yunlin, Taiwan; Department of Cardiology (A Raja MD), Dow University of Health Sciences, Karachi, Pakistan; Department of Cardiology (Prof M M Ramadan PhD), Mansoura University, Mansoura, Egypt; Department of Radiology (S Ramasamy MD), Stanford University, Stanford, CA, USA; Centre for Clinical

Pharmacology (N Rancic PhD), University of Defence in Belgrade, Belgrade, Serbia; Centre for Clinical Pharmacology (N Rancic PhD), Medical College of Georgia at Augusta University, Belgrade, Serbia; Department of Oral Pathology, Microbiology and Forensic Odontology (S Rao MDS), Sharavathi Dental College and Hospital, Shimogga, India; Department of Family Medicine (Prof D Rathish PhD), Department of Community Medicine (N D Wickramasinghe MD), Rajarata University of Sri Lanka, Anuradhapura, Sri Lanka; Academic Public Health England (Prof S Rawaf MD), Public Health England, London, UK; Department of Biological Sciences (Prof E M M Redwan PhD), King Abdulaziz University, Jeddah, Egypt; Department of Protein Research (Prof E M M Redwan PhD), Research and Academic Institution, Alexandria, Egypt; Department of Public Health Sciences (T Rhee PhD), University of Connecticut, Farmington, CT, USA; Department of Pharmacy (M Riaz PhD), Shaheed Benazir Bhutto University Sheringal Pakistan, Dir Upper, Pakistan; Department of Pharmacology and Toxicology (Prof J A B Rodriguez PhD), University of Antioquia, Medellin, Colombia; Warwick Medical School (Prof J A B Rodriguez PhD), University of Warwick, Coventry, UK; Department of Clinical Research (Prof L Roever PhD), University of Sao Paulo, Ribeirão Preto, Brazil; Fondazione Policlinico Universitario A. Gemelli (M Romozzi MD), Cuore Università Cattolica del Sacro Cuore (Catholic University of Sacred Heart), Rome, Italy; Department of Public Health (M Rony MPH), Bangladesh Open University, Gazipur, Bangladesh; Department of Analytical and Applied Economics (Prof H Rout PhD, C Swain MPhil), RUSA Centre of Excellence in Public Policy and Governance (Prof H Rout PhD), UGC Centre of Advanced Study in Psychology (Prof M Satpathy PhD), Utkal University, Bhubaneswar, India; Cardiovascular Department (Prof A M A Saad MD), Zagazig University, Zagazig, Egypt; Department of Pharmaceutical Chemistry (Prof M Saeb PhD), International Medical University, Gdańsk, Poland; Operational Research Center in Healthcare (Prof U Saeed PhD), Near East University (NEU), Nicosia Cyprus, Turkiye; International Center of Medical Sciences Research (ICMSR), Islamabad, Pakistan (Prof U Saeed PhD); Hurvitz Brain Sciences Research Program (U Saeed MSc), Sunnybrook Research Institute, Toronto, ON, Canada; Department of Nutrition and Dietetics (Prof S Sajadi PhD), Cihan University, Erbil, Erbil, Iraq; Institute of Epidemiology and Preventive Medicine (Y L Samodra PhD), National Taiwan University, Taipei, Taiwan; Benang Merah Research Center (BMRC), Minahasa Utara, Indonesia (Y L Samodra PhD); Faculty of Health & Social Sciences (B Sathian PhD), Bournemouth University, Bournemouth, UK; Department of Medicine (A Sathyanarayan MD), Bangalore Medical College and Research Institute, Bangalore, India; Udyam-Global Association for Sustainable Development, Bhubaneswar, India (Prof M Satpathy PhD); Department of Public Health Sciences (M Sawhney PhD), University of North Carolina at Charlotte, Charlotte, NC, USA; Emergency Department (S Senthilkumaran PhD), Manian Medical Centre, Erode, India; Department of Medicine and Surgery (Y Sethi MBBS), Government Doon Medical College, Dehradun, India; National Heart, Lung, and Blood Institute (A Seylani BS), National Institutes of Health, Rockville, MD, USA; Independent Consultant, Karachi, Pakistan (M A Shaikh MD); Department of Neuro-Physiotherapy (S Z Shaikh PhD), Independent Consultant, Thane, India; School of Medicine (M Shams-Beyranvand MSc), Alborz University of Medical Sciences, Karaj, Iran; Department for Evidence-based Medicine and Evaluation (A Sharifan PharmD), University for Continuing Education Krems, Krems, Austria; Department of Medicine (J Sharifi Rad PhD), Korea University, Seoul, South Korea; Department of Hemato-oncology (A Sharma MD), Fortis Hospital, Noida, India; Department of Neurology (Z B Sheikh MD), West Virginia University, Morgantown, WV, USA; K S Hegde Medical Academy (Prof M Shetty MD), Nitte University, Mangalore, India; Department of Veterinary Public Health and Preventive Medicine (A Shittu MSc), Usmanu Danfodiyo University, Sokoto, Sokoto, Nigeria; Department of Research and Academics (S Shrestha PhD), Kathmandu Cancer Center, Bhaktapur, Nepal; Department of Medical Microbiology and

Infectious Diseases (E E Siddig MD), Erasmus University, Rotterdam, Netherlands; Department of Neurology (Prof G Singh MD), Dayanand Medical College and Hospital, Ludhiana, India; Institute of Neurology (Prof G Singh MD), University College London, London, UK; Department of Pharmacology (H Singh DM), Government Medical College and Hospital, Chandigarh, India; Department of Medicine Service (Prof J A Singh MD), US Department of Veterans Affairs (VA), Houston, TX, USA; Department of Human Genetics (P Singh PhD), Punjabi University, Patiala, India; Department of Biochemistry (S Solanki MD), American University of Integrative Sciences, Bridgetown, Barbados; Student Research Committee (S Soraneh MD), Urmia University of Medical Sciences, Urmia, Iran; School of Medicine (S Soraneh MD), Babol University of Medical Sciences, Babol, Iran; Department of Public Health (M Stanikzai MPH), Kandahar University, Kandahar, Afghanistan; Institute of Neuroscience and Physiology (Prof K S Sunnerhagen PhD), University of Gothenburg, Gothenburg, Sweden; Department of Neurocare (Prof K S Sunnerhagen PhD), Sabzevar University of Medical Sciences, Gothenburg, Sweden; Department of Clinical Research and Development (Prof L Szarpak PhD), LUXMED Group, Warsaw, Poland; Collegium Medicul (Prof L Szarpak PhD), John Paul II Catholic University of Lublin, Lublin, Poland; Department of Neurology (P Tabaee Damavandi MD), Neurocenter of Southern Switzerland (NSI), Lugano, Switzerland; Department of Medicine (Prof R Tabarés-Seisdedos PhD), University of Valencia, Valencia, Spain; Carlos III Health Institute (Prof R Tabarés-Seisdedos PhD), Biomedical Research Networking Center for Mental Health Network (CiberSAM), Madrid, Spain; Department of Environmental, Agricultural and Occupational Health (J Taiba PhD), University of Nebraska Medical Center, Omaha, NE, USA; Sri Ramachandra Medical College and Research Institute, Chennai, India (J Taiba PhD); Department of Radiology (M Tanwar MD), University of Alabama at Birmingham, Birmingham, AL, USA; Pediatric Intensive Care Unit (Prof M Temsah MD), King Saud University, Riyadh, Saudi Arabia; Department of Pharmacology (P Thangaraju MD), All India Institute of Medical Sciences, Raipur, India; Faculty of Public Health (J H V Ticoalu MPH), Universitas Sam Ratulangi (Sam Ratulangi University), Manado, Indonesia; Department of Allied Health and Human Performance (T Y Tiruye PhD), University of South Australia, Adelaide, SA, Australia; Public Health Department (T Y Tiruye PhD), Debre Markos University, Debre Markos, Ethiopia; Department of Physiology (V K Tiwari MD), All India Institute of Medical Sciences, New Delhi, India; Systems Neuroscience (V K Tiwari MD), Tohoku University, Sendai, Japan; Department of Business Analytics (T H Tran MD), University of Massachusetts Dartmouth, Dartmouth, MA, USA; Molecular Neuroscience Research Center (N Tran Minh Duc MD), Shiga University of Medical Science, Shiga, Japan; Department of Neurology (Prof M Tripathi MD), All India Institute of Medical Sciences, Delhi, India; Department of Health Sciences (S J Tromans PhD), University of Leicester, Leicester, UK; Adult Learning Disability Service (S J Tromans PhD), Leicestershire Partnership National Health Service Trust, Leicester, UK; School of Pharmacy (D Tsai MSc), National Cheng Kung University, Tainan, Taiwan; Centre for Neonatal and Paediatric Infection (D Tsai MSc), St George's University of London, London, UK; Department of Medicine (Prof A Tsatsakis DSc), University of Crete, Heraklion, Greece; Department of Psychiatry (E Tsermpini PhD), Dalhousie University, Halifax, NS, Canada; Department of Internal Medicine (M Tumurkhuu PhD), Wake Forest University, Winston-Salem, NC, USA; Department of Biosciences and Biotechnology (A J Udoakang PhD), University of Medical Sciences, Ondo, Ondo, Nigeria; International Center for Chemical and Biological Sciences (S Ullah MSc), University of Karachi, Karachi, Pakistan; Medical Genomics Research Department (Prof M Umair PhD), King Abdullah International Medical Research Center, Riyadh, Saudi Arabia; Center for Neurodegenerative Diseases and the Aging Brain (D Urso MD), University of Bari, Tricase, Italy; Institute of Psychiatry, Psychology & Neuroscience (D Urso MD), School of Life Course and Population Sciences (Prof Y Wang PhD), King's College London,

London, UK; College of Health and Sport Sciences (A G Vaithinathan MSc), University of Bahrain, Zallaq, Bahrain; Achutha Menon Centre for Health Science Studies (R P Varma MD), Sree Chitra Tirunal Institute for Medical Sciences and Technology, Trivandrum, India; Raffles Neuroscience Centre (Prof N Venketasubramanian MSc), Raffles Hospital, Singapore, Singapore; Yong Loo Lin School of Medicine (Prof N Venketasubramanian MSc), National University of Singapore, Singapore, Singapore; Department of Physiotherapy (J H Villafañe PhD), Universidad Europea de Madrid (European University of Madrid), Villaviciosa de Odón, Spain; Department of Cardiology (M Vinayak MD), Icahn School of Medicine at Mount Sinai, New York, NY, USA; Programa de doctorado IPK (A Vinueza Veloz MSc), Institute of Tropical Medicine, La Habana, Cuba; Department of Neurosurgery (S Wang MD), Capital Medical University, Beijing, China; Department of Neurosurgery (S Wang MD), Beijing Tiantan Hospital, Beijing, China; Institute of Health and Society (Prof A S Winkler PhD), University of Oslo, Oslo, Norway; Department of Neurology (Prof A S Winkler PhD), Technical University of Munich, Munich, Germany; Department of Family Medicine (S A Yesuf MSc), St. Paul's Hospital Millennium Medical College, Addis Ababa, Ethiopia; Independent Consultant, Addis Ababa, Ethiopia (S A Yesuf MSc); Department of Health Management (A Yiğit PhD, V Yiğit PhD), Süleyman Demirel Üniversitesi (Süleyman Demirel University), Isparta, Turkiye; Pharmacy Department (Y Yismaw MSc), Alkan Health Science, Business and Technology College, Bahir Dar, Ethiopia; Department of Pediatrics (Prof D Yon MD), Kyung Hee University, Seoul, South Korea; Department of Biostatistics (Prof N Yonemoto PhD), University of Toyama, Toyama, Japan; Department of Public Health (Prof N Yonemoto PhD), Juntendo University, Tokyo, Japan; Department of Epidemiology and Biostatistics (Prof C Yu PhD), Wuhan University, Wuhan, China; Hepatitis Research Center (M Zandi PhD), Lorestan University of Medical Sciences, Khorramabad, Iran; Sant'Elia Hospital (A Zanghì MD), University of Catania, Caltanissetta, Italy; Department of Health Management (Z Zhao PhD), Shengjing Hospital of China Medical University, Shenyang, China; School of Public Health Sciences (O A Zitoun MD), University of Waterloo, Waterloo, ON, Canada; College of Medicine (O A Zitoun MD), Sulaiman Alrajhi University, Al Bukairiyah, Saudi Arabia; Department of Clinical and Community Pharmacy (Prof S H Zyoud PhD), An-Najah National University, Nablus, Palestine; Clinical Research Centre, An-Najah National University Hospital (Prof S H Zyoud PhD), An-Najah National University Hospital, Nablus, Palestine; Department of Neurosciences (Prof C R J Newton MD), Kenya Medical Research Institute/Wellcome Trust Research Programme, Kilifi, Kenya; Department of Clinical Neurosciences (Prof S Wiebe MD), Community Health Sciences (Prof S Wiebe MD), University of Calgary, Calgary, AB, Canada

Authors’ Contributions

Managing the overall research enterprise

Valery L Feigin, Simon I Hay, Christopher J L Murray, Balakrishnan Sukumaran Nair, Charles Richard James Newton, Ilari Rautalin, Theo Vos, and Samuel Wiebe.

Writing the first draft of the manuscript

Valery L Feigin

Primary responsibility for applying analytical methods to produce estimates

Christopher J L Murray

Primary responsibility for seeking, cataloguing, extracting, or cleaning data; designing or coding figures and tables

Balakrishnan Sukumaran Nair and Ilari Rautalin.

Providing data or critical feedback on data sources

Yohannes Habtegiorgis Abate, Abdallah H A Abd Al Magied, Samar Abd ElHafeez, Mohammad-Amin Abdollahifar, Auwal Abdullahi, Richard Gyan Aboagye, Lucas Guimarães Abreu, Samir Abu Rumeileh, Ahmed Abu-Zaid, Abiola Victor Victor Adepoju, Muhammad Sohail Afzal, Saira Afzal, Sajjad Ahmad, Tauseef Ahmad, Ali Ahmadi, Ayman Ahmed, Haroon Ahmed, Mehrunnisha Sharif Ahmed, Muktar Beshir Ahmed, Salah Al Awaidy, Yazan Al-Ajlouni, Mohammed Albashtawy, Abdelazeem M Algammal, Abid Ali, Syed Shujait Ali, Sheikh Mohammad Alif, Joseph Uy Almazan, Awais Altaf, Mohammad Al-Wardat, Dhanalakshmi Angappan, Saeid Anvari, Jalal Arabloo, Alok Atreya, Ahmed Y Azzam, Atif Amin Baig, Indrajit Banerjee, Mainak Bardhan, Amadou Barrow, Mohammad-Mahdi Bastan, Babak Behnam, Akshaya Srikanth Bhagavathula, Sonu Bhaskar, Ajay Nagesh Bhat, Gurjit Kaur Bhatti, Jasvinder Singh Singh Bhatti, Mohiuddin Ahmed Bhuiyan, Cem Bilgin, Francesca Bisulli, Archith Boloor, Souad Bouaoud, Mehtap Çakmak Barsbay, Joao Mauricio Castaldelli-Maia, Vijay Kumar Chattu, Hitesh Chopra, Dinh-Toi Chu, Samuele Cortese, Paolo Angelo Cortesi, Natalia Cruz-Martins, Xiaochen Dai, Lalit Dandona, Rakhi Dandona, Samuel Demissie Darcho, Michael J Diaz, Thanh Chi Do, Ojas Prakashbhai Doshi, Haneil Larson Dsouza, Michael Ekholuenetale, Rabie Adel El Arab, Ibrahim Farahat El Bayoumy, Chadi Eltaha, Adeniyi Francis Fagbamigbe, Jawad Fares, Abidemi Omolara Fasanmi, Ali Fatehizadeh, Timur Fazylov, Valery L Feigin, Ginenus Fekadu, Seyed-Mohammad Fereshtehnejad, Muktar A Gadanya, Yaseen Galali, Ravindra Kumar Garg, Seyyed-Hadi Ghamari, Jaleed Ahmed Gilani, Alem Abera Girmay, Mahaveer Golechha, Ayman Grada, Shi-Yang Guan, Sapna Gupta, Arvin Haj-Mirzaian, Nadia M Hamdy, Mahgol Sadat Hassan Zadeh Tabatabaei, Simon I Hay, Golnaz Heidari, Mehdi Hemmati, Nguyen Quoc Hoan, Mehdi Hosseinzadeh, Hong-Han Huynh, Segun Emmanuel Ibitoye, Olayinka Stephen Ilesanmi, Haitham Jahrami, Mihajlo Jakovljevic, Talha Jawaid, Sathish Kumar Jayapal, Jost B Jonas, Charity Ehimwenma Joshua, Zubair Kabir, Neeti Kapoor, Faizan Zaffar Kashoo, Harkiran Kaur, Himanshu Khajuria, Maseer Khan, Feriha Fatima Khidri, Atulya Aman Khosla, Yun Jin Kim, Kewal Krishan, Vijay Krishnamoorthy, Jera Kruja, G Anil Kumar, Manasi Kumar, Ville Kytö, Chandrakant Lahariya, Dharmesh Kumar Lal, Nhi Huu Hanh Le, Seung Won Lee, Stephen S Lim, Xuefeng Liu, José Francisco López-Gil, Kashish Malhotra, Roy Rillera Marzo, Man Mohan Mehndiratta, Endalkachew Belayneh Melese, Atte Meretoja, Sachith Mettananda, Awoke Misganaw, Arup Kumar Misra, Nouh Saad Mohamed, Abdollah Mohammadian-Hafshejani, Syam Mohan, Ali H Mokdad, Lorenzo Monasta, Maryam Moradi, Francesk Mulita, Efren Murillo-Zamora, Christopher J L Murray, Ganesh R Naik, Balakrishnan Sukumaran Nair, Shumaila Nargus, Zuhair S Natto, Muhammad Naveed, Biswa Prakash Nayak, Henok Biresaw Netsere, Charles Richard James Newton, Hau Thi Hien Nguyen, Robina Khan Niazi, Ogochukwu Janet Nzoputam, Bogdan Oancea, Andrew T Olagunju, Oladotun Victor Olalusi, Ahmed Omar Bali, Amel Ouyahia, Mayowa O Owolabi, Jagadish Rao Padubidri, Sujogya Kumar Panda, Songhomitra Panda-Jonas, Anamika Pandey, Romil R Parikh, Shankargouda Patil, Shrikant Pawar, Prince Peprah, Simone Perna, Hoang Nhat Pham, Zahra Zahid Piracha, Ramesh Poluru, Jalandhar Pradhan, Jagadeesh Puvvula, Pankaja Raghav, Fakher Rahim, Amir Masoud Rahmani, Adarsh Raja, Pushp Lata Rajpoot, Mahmoud Mohammed Ramadan, Shakthi Kumaran Ramasamy, Nemanja Rancic, Sowmya J Rao, Ilari Rautalin, Salman Rawaf, Jefferson Antonio Buendia Rodriguez, Leonardo Roever, Marina Romozzi, Moustaq Karim Khan Rony, Aly M A Saad, Cameron John Sabet, Basema Ahmad Saddik, Umar Saeed, Narjes Saheb Sharif-Askari, Zahra Saif, S Mohammad Sajadi, Afeez

Abolarinwa Salami, Sohrab Salimi, Abdallah M Samy, Brijesh Sathian, Anudeep Sathyanarayan, Maheswar Satpathy, Monika Sawhney, Siddharthan Selvaraj, Mohammad H Semreen, Subramanian Senthilkumaran, Yashendra Sethi, Allen Seylani, Samiah Shahid, Masood Ali Shaikh, Summaiya Zareen Shaikh, Muhammad Aaqib Shamim, Mehran Shams-Beyranvand, Alfiya Shamsutdinova, Amin Sharifan, Javad Sharifi Rad, Vishal Sharma, Aminu Shittu, Nathan A Shlobin, Sunil Shrestha, Jasvinder A Singh, Paramdeep Singh, Muhammad Haroon Stanikzai, Mark J M Sullman, Chandan Kumar Swain, Lukasz Szarpak, Rafael Tabarés-Seisdedos, Celine Tabche, Manoj Tanwar, Minale Tareke, Pugazhenthan Thangaraju, Krishna Tiwari, Vikas Kumar Tiwari, Marcos Roberto Tovani-Palone, Munkhtuya Tumurkhuu, Muhammad Umair, Bhaskaran Unnikrishnan, Jibrin Sammani Usman, Narayanaswamy Venketasubramanian, Theo Vos, Mandaras Tariku Walde, Shu Wang, Abdul Waris, Samuel Wiebe, Mekdes Tigistu Yilma, Dong Keon Yon, Naohiro Yonemoto, Chuanhua Yu, Magdalena Zielińska, and Sa'ed H Zyoud.

Developing methods or computational machinery

Aleksandr Y Aravkin, Xiaochen Dai, Simon I Hay, Ali H Mokdad, Christopher J L Murray, and Theo Vos.

Providing critical feedback on methods or results

Yohannes Habtegiorgis Abate, Abdallah H A Abd Al Magied, Samar Abd ElHafeez, Atef Abdelkader, Auwal Abdullahi, Richard Gyan Aboagye, Samir Abu Rumeileh, Salahdein Aburuz, Ahmed Abu-Zaid, Isaac Yeboah Addo, Rufus Adesoji Adedoyin, Abiola Victor Victor Adepoju, Muhammad Sohail Afzal, Saira Afzal, Aqeel Ahmad, Sajjad Ahmad, Tauseef Ahmad, Ali Ahmadi, Amir Mahmoud Ahmadzade, Ayman Ahmed, Haroon Ahmed, Mehrunnisha Sharif Ahmed, Muktar Beshir Ahmed, Salah Al Awaidy, Omar Al Omari, Mohammed Albashtawy, Bassam Al-Fatly, Abdelazeem M Algammal, Abid Ali, Mohammed Usman Ali, Syed Shujait Ali, Joseph Uy Almazan, Najim Z Alshahrani, Awais Altaf, Mohammad Al-Wardat, Yaser Mohammed Al-Worafi, Hany Aly, Karem H Alzoubi, Sohrab Amiri, Robert Ancuceanu, Mohammed Tahir Tahir Ansari, Saeid Anvari, Anayochukwu Edward Anyasodor, Jalal Arabloo, Mosab Arafat, Maha Moh'd Wahbi Atout, Alok Atreya, Avinash Aujayeb, Setognal Birara Aychiluhm, Shahkaar Aziz, Ahmed Y Azzam, Ashish D Badiye, Ruhai Bai, Atif Amin Baig, Shankar M Bakkannavar, Indrajit Banerjee, Mainak Bardhan, Amadou Barrow, Zarrin Basharat, Mohammad-Mahdi Bastan, Sai Batchu, Babak Behnam, Diana Fernanda Bejarano Ramirez, Maryam Bemanalizadeh, Devidas S Bhagat, Akshaya Srikanth Bhagavathula, Sonu Bhaskar, Ajay Nagesh Bhat, Gurjit Kaur Bhatti, Jasvinder Singh Singh Bhatti, Mohiuddin Ahmed Bhuiyan, Soumitra S Bhuyan, Cem Bilgin, Francesca Bisulli, Archith Boloor, Sri Harsha Boppana, Souad Bouaoud, Yasser Bustanji, Mehtap Çakmak Barsbay, Joao Mauricio Castaldelli-Maia, Rama Mohan Chandika, Vijay Kumar Chattu, Hitesh Chopra, Dinh-Toi Chu, Hongyuan Chu, Samuele Cortese, Natalia Cruz-Martins, Omid Dadras, Xiaochen Dai, Emanuele D'Amico, Samuel Demissie Darcho, Amira Hamed Darwish, Amol S Dhane, Vishal R Dhulipala, Michael J Diaz, Thanh Chi Do, Ojas Prakashbhai Doshi, Haneil Larson Dsouza, Arkadiusz Marian Dziedzic, Alireza Ebrahimi, Michael Ekholuenetale, Rabie Adel El Arab, Ibrahim Farahat El Bayoumy, Omar Abdelsadek Abdou El Meligy, Hala Rashad Elhabashy, Muhammed Elhadi, Chadi Eltaha, Adeniyi Francis Fagbamigbe, Ayesha Fahim, Jawad Fares, Abidemi Omolara Fasanmi, Ali Fatehizadeh, Patrick Fazeli, Valery L Feigin, Ginenus Fekadu, Seyed-Mohammad Fereshtehnejad, Pietro Ferrara, Getahun Fetensa, Florian Fischer, Matteo Foschi, Muktar A Gadanya, Yaseen Galali, Balasankar Ganesan, Xiang Gao, Ravindra Kumar Garg, Miglas Welay Gebregergis, Seyyed-Hadi Ghamari, Jaleed Ahmed Gilani, Alem Abera Girmay, Giorgia Giussani, Mahaveer Golechha, Ayman Grada, Shi-Yang Guan, Sapna Gupta, Arvin Haj-Mirzaian, Nadia M Hamdy, Netanja I Harlianto, Ahmed I Hasaballah, Hamidreza Hasani, Ikrama Ibrahim Hassan, Mahgol Sadat Hassan Zadeh Tabatabaei, Simon I

Hay, Golnaz Heidari, Mehdi Hemmati, Kamal Hezam, Nguyen Quoc Hoan, Ramesh Holla, Mehdi Hosseinzadeh, Hong-Han Huynh, Bing-Fang Hwang, Segun Emmanuel Ibitoye, Olayinka Stephen Ilesanmi, Irena M Ilic, Milena D Ilic, Mohammad Tarique Imam, Mustapha Immurana, Arit Inok, Md Rabiul Islam, Chidozie Declan Iwu, Louis Jacob, Haitham Jahrami, Ammar Abdulrahman Jairoun, Mihajlo Jakovljevic, Reza Jalilzadeh Yengejeh, Roland Dominic G Jamora, Sathish Kumar Jayapal, Zixiang Ji, Jost B Jonas, Nitin Joseph, Charity Ehimwenma Joshua, Zubair Kabir, Rizwan Kalani, Kehinde Kazeem Kanmodi, Neeti Kapoor, Faizan Zaffar Kashoo, Foad Kazemi, Alireza Khalilian, Maseer Khan, Haitham Khatatbeh, Hamid Reza Khayat Kashani, Khalid A Kheirallah, Feriha Fatima Khidri, Moein Khormali, Atulya Aman Khosla, Jagdish Khubchandani, Yun Jin Kim, Ruth W Kimokoti, Hyun Yong Koh, Ali-Asghar Kolahi, Kewal Krishan, Vijay Krishnamoorthy, Mohammed Kuddus, Satyajit Kundu, Ville Kytö, Chandrakant Lahariya, Dharmesh Kumar Lal, Judit Lám, Iván Landires, Francesco Lanfranchi, Nhi Huu Hanh Le, Seung Won Lee, Virendra S Ligade, Stephen S Lim, Xiaofeng Liu, Xuefeng Liu, José Francisco López-Gil, Giancarlo Lucchetti, Azeem Majeed, Kashish Malhotra, Ahmad Azam Malik, Vahid Mansouri, Hamid Reza Marateb, Miquel Martorell, Roy Rillera Marzo, Yasith Mathangasinghe, Rishi P Mediratta, Man Mohan Mehndiratta, Hadush Negash Meles, Endalkachew Belayneh Melese, Atte Meretoja, Tomislav Mestrovic, Reza Mirfakhraie, Moonis Mirza, Awoke Misganaw, Abdalla Z Mohamed, Nouh Saad Mohamed, Abdollah Mohammadian-Hafshejani, Ibrahim Mohammadzadeh, Syam Mohan, Ali H Mokdad, AmirAli Moodi Ghalibaf, Maryam Moradi, Rohith Motappa, Lorenzo Muccioli, Francesk Mulita, Yanjinlkham Munkhsaikhan, Efren Murillo-Zamora, Christopher J L Murray, Sathish Muthu, Ganesh R Naik, Shumaila Nargus, Abdulqadir J Nashwan, Zuhair S Natto, Javaid Nauman, Muhammad Naveed, Biswa Prakash Nayak, Athare Nazri-Panjaki, Henok Biresaw Netsere, Charles Richard James Newton, Robina Khan Niazi, Ali Nikoobar, Majid Nozari, Chisom Adaobi Nri-Ezedi, Vincent Ebuka Nwatah, Ogochukwu Janet Nzoputam, Bogdan Oancea, Andrew T Olagunju, Oladotun Victor Olalusi, Ahmed Omar Bali, Michal Ordak, Nikita Otstavnov, Amel Ouyahia, Mayowa O Owolabi, Jagadish Rao Padubidri, Sujogya Kumar Panda, Songhomitra Panda-Jonas, Deepshikha Pande Katare, Leonidas D Panos, Ioannis Pantazopoulos, Paraskevi Papadopoulou, Utsav Parekh, Romil R Parikh, Nicholas Parsons, Shankargouda Patil, Shrikant Pawar, Prince Peprah, Mario F P Peres, Simone Perna, Hoang Nhat Pham, Zahra Zahid Piracha, Michael A Piradov, Ramesh Poluru, Ahmad Pour-Rashidi, Jalandhar Pradhan, Manya Prasad, Dimas Ria Angga Pribadi, Jagadeesh Puvvula, Nameer Hashim Qasim, Venkatraman Radhakrishnan, Pankaja Raghav, Fakher Rahim, Mosiur Rahman, Amir Masoud Rahmani, Mohammad Rahmanian, Adarsh Raja, Ali Rajabpour Sanati, Pushp Lata Rajpoot, Mahmoud Mohammed Ramadan, Shakthi Kumaran Ramasamy, Nemanja Rancic, Sowmya J Rao, Mohammad-Mahdi Rashidi, Devarajan Rathish, Ilari Rautalin, Salman Rawaf, Murali Mohan Rama Krishna Reddy, Elrashdy M Moustafa Mohamed Redwan, Mohsen Rezaeian, Taeho Gregory Rhee, Jefferson Antonio Buendia Rodriguez, Leonardo Roever, Marina Romozzi, Moustaq Karim Khan Rony, Kevin T Root, Himanshu Sekhar Rout, Aly M A Saad, Cameron John Sabet, Basema Ahmad Saddik, Reihaneh Sadeghian, Mohammad Reza Saeb, Umar Saeed, Usman Saeed, Fatemeh Saheb Sharif-Askari, Narjes Saheb Sharif-Askari, Zahra Saif, S Mohammad Sajadi, Afeez Abolarinwa Salami, Yoseph Leonardo Samodra, Abdallah M Samy, Gargi Sachin Sarode, Sachin C Sarode, Brijesh Sathian, Maheswar Satpathy, Monika Sawhney, Siddharthan Selvaraj, Mohammad H Semreen, Yashendra Sethi, Ataollah Shahbandi, Samiah Shahid, Masood Ali Shaikh, Summaiya Zareen Shaikh, Muhammad Aaqib Shamim, Mehran Shams-Beyranvand, Amin Sharifan, Javad Sharifi Rad, Anupam Sharma, Vishal Sharma, Maryam Shayan, Zubeda Begum Sheikh, Aminu Shittu, Nathan A Shlobin, Seyed Afshin Shorofi, Sunil Shrestha, Emmanuel Edwar Siddig, Gagandeep Singh, Harmanjit Singh, Jasvinder A Singh, Paramdeep Singh, Puneetpal Singh, Soroush Soraneh, Muhammad Haroon Stanikzai, Mark J M Sullman, Katharina S

Sunnerhagen, Vinay Suresh, Chandan Kumar Swain, Lukasz Szarpak, Payam Tabaee Damavandi, Rafael Tabarés-Seisdedos, Celine Tabche, Jabeen Taiba, Manoj Tanwar, Minale Tareke, Mohamad-Hani Temsah, Masayuki Teramoto, Pugazhenthan Thangaraju, Sathish Thirunavukkarasu, Jansje Henny Vera Ticoalu, Tenaw Yimer Tiruye, Krishna Tiwari, Vikas Kumar Tiwari, Marcos Roberto Tovani-Palone, Nguyen Tran Minh Duc, Manjari Tripathi, Samuel Joseph Tromans, Daniel Hsiang-Te Tsai, Munkhtuya Tumurkhuu, Aniefiok John Udoakang, Saeed Ullah, Muhammad Umair, Bhaskaran Unnikrishnan, Jibrin Sammani Usman, Alireza Vakilian, Narayanaswamy Venketasubramanian, Jorge Hugo Villafañe, Manish Vinayak, Andres Fernando Vinueza Veloz, Theo Vos, Mandaras Tariku Walde, Shu Wang, Yanzhong Wang, Abdul Waris, Nuwan Darshana Wickramasinghe, Samuel Wiebe, Andrea Sylvia Winkler, Subah Abderehim Yesuf, Arzu Yiğit, Vahit Yiğit, Mekdes Tigistu Yilma, Yazachew Engida Engida Engida Yismaw, Dong Keon Yon, Naohiro Yonemoto, Chuanhua Yu, Milad Zandi, Aurora Zanghì, Mohammed G M Zeariya, Zhongyi Zhao, Claire Chenwen Zhong, Magdalena Zielińska, Osama A Zitoun, Sa'ed H Zyoud,

Drafting the work or revising it critically for important intellectual content

Yohannes Habtegiorgis Abate, Abdallah H A Abd Al Magied, Samar Abd ElHafeez, Atef Abdelkader, Auwal Abdullahi, Lucas Guimarães Abreu, Samir Abu Rumeileh, Hasan Abualruz, Salahdein Aburuz, Ahmed Abu-Zaid, Isaac Yeboah Addo, Rufus Adesoji Adedoyin, Abiola Victor Victor Adepoju, Muhammad Sohail Afzal, Saira Afzal, Ali Ahmadi, Ayman Ahmed, Mehrunnisha Sharif Ahmed, Muktar Beshir Ahmed, Omar Al Omari, Mohammed Albashtawy, Bassam Al-Fatly, Abdelazeem M Algammal, Abid Ali, Mohammed Usman Ali, Syed Shujait Ali, Waad Ali, Najim Z Alshahrani, Mohammad Al-Wardat, Yaser Mohammed Al-Worafi, Hany Aly, Sohrab Amiri, Robert Ancuceanu, Dhanalakshmi Angappan, Saeid Anvari, Anayochukwu Edward Anyasodor, Jalal Arabloo, Brhane Berhe Aregawi, Abdulfatai Aremu, Maha Moh'd Wahbi Atout, Alok Atreya, Avinash Aujayeb, Shahkaar Aziz, Ahmed Y Azzam, Ashish D Badiye, Atif Amin Baig, Soham Bandyopadhyay, Indrajit Banerjee, Mainak Bardhan, Suzanne Lyn Barker-Collo, Amadou Barrow, Azadeh Bashiri, Afisu Basiru, Mohammad-Mahdi Bastan, Sai Batchu, Babak Behnam, Kebede A Beyene, Akshaya Srikanth Bhagavathula, Sonu Bhaskar, Ajay Nagesh Bhat, Gurjit Kaur Bhatti, Jasvinder Singh Singh Bhatti, Sri Harsha Boppana, Souad Bouaoud, Yasser Bustanji, Mehtap Çakmak Barsbay, Felix Carvalho, Joao Mauricio Castaldelli-Maia, Rama Mohan Chandika, Vijay Kumar Chattu, Anis Ahmad Chaudhary, Patrick R Ching, Dinh-Toi Chu, Hongyuan Chu, Samuele Cortese, Paolo Angelo Cortesi, Natalia Cruz-Martins, Emanuele D'Amico, Samuel Demissie Darcho, Amira Hamed Darwish, Amol S Dhane, Vishal R Dhulipala, Michael J Diaz, Thanh Chi Do, Sushil Dohare, Ojas Prakashbhai Doshi, Haneil Larson Dsouza, Arkadiusz Marian Dziedzic, Negin Eissazade, Michael Ekholuenetale, Rabie Adel El Arab, Ibrahim Farahat El Bayoumy, Omar Abdelsadek Abdou El Meligy, Muhammed Elhadi, Chadi Eltaha, Adeniyi Francis Fagbamigbe, Ayesha Fahim, Jawad Fares, Mohsen Farjoud Kouhanjani, Ali Fatehizadeh, Valery L Feigin, Seyed-Mohammad Fereshtehnejad, Pietro Ferrara, Nuno Ferreira, Getahun Fetensa, Florian Fischer, Matteo Foschi, Muktar A Gadanya, Balasankar Ganesan, Ravindra Kumar Garg, Miglas Welay Gebregergis, Fataneh Ghadirian, Seyyed-Hadi Ghamari, Jaleed Ahmed Gilani, Alem Abera Girmay, Elena V Gnedovskaya, Mahdi Gouravani, Ayman Grada, Shi-Yang Guan, Sapna Gupta, Mohammad Haghani Dogahe, Arvin Haj-Mirzaian, Nadia M Hamdy, Netanja I Harlianto, Ahmed I Hasaballah, Hamidreza Hasani, Amr Hassan, Mahgol Sadat Hassan Zadeh Tabatabaei, Simon I Hay, Omar E Hegazi, Golnaz Heidari, Mehdi Hemmati, Kamal Hezam, Nguyen Quoc Hoan, Ramesh Holla, Mehdi Hosseinzadeh, Junjie Huang, Hong-Han Huynh, Segun Emmanuel Ibitoye, Adalia Ikiroma, Olayinka Stephen Ilesanmi, Irena M Ilic, Milena D Ilic, Mustapha Immurana, Arit Inok, Md Rabiul Islam, Chidozie Declan Iwu, Louis Jacob, Abdollah Jafarzadeh, Haitham Jahrami, Mihajlo Jakovljevic, Roland Dominic G Jamora, Sathish Kumar Jayapal, Jost B Jonas, Nitin Joseph, Charity Ehimwenma Joshua, Rizwan Kalani,

Arun Kamireddy, Kehinde Kazeem Kanmodi, Neeti Kapoor, Faizan Zaffar Kashoo, Foad Kazemi, Himanshu Khajuria, Maseer Khan, Haitham Khatatbeh, Khalid A Kheirallah, Feriha Fatima Khidri, Atulya Aman Khosla, Jagdish Khubchandani, Yun Seo Kim, Hyun Yong Koh, Karel Kostev, Kewal Krishan, Mohammed Kuddus, Mukhtar Kulimbet, Satyajit Kundu, Chandrakant Lahariya, Judit Lám, Iván Landires, Francesco Lanfranchi, Nhi Huu Hanh Le, Christine Linehan, José Francisco López-Gil, Giancarlo Lucchetti, Kashish Malhotra, Ahmad Azam Malik, Hamid Reza Marateb, Miquel Martorell, Roy Rillera Marzo, Yasith Mathangasinghe, Rishi P Mediratta, Hadush Negash Meles, Endalkachew Belayneh Melese, George A Mensah, Atte Meretoja, Tomislav Mestrovic, Sachith Mettananda, Giuseppe Minervini, Moonis Mirza, Abdalla Z Mohamed, Nouh Saad Mohamed, Ali H Mokdad, Lorenzo Monasta, AmirAli Moodi Ghalibaf, Maryam Moradi, Rohith Motappa, Francesk Mulita, Yanjinlkham Munkhsaikhan, Efren Murillo-Zamora, Christopher J L Murray, Sathish Muthu, Amin Nabavi, Balakrishnan Sukumaran Nair, Shumaila Nargus, Abdulqadir J Nashwan, Zuhair S Natto, Javaid Nauman, Biswa Prakash Nayak, Gaurav Nepal, Hau Thi Hien Nguyen, Robina Khan Niazi, Chisom Adaobi Nri-Ezedi, Vincent Ebuka Nwatah, Ogochukwu Janet Nzoputam, Bogdan Oancea, Andrew T Olagunju, Oladotun Victor Olalusi, Verner N Orish, Esteban Ortiz-Prado, Nikita Otstavnov, Mayowa O Owolabi, Alicia Padron-Monedero, Jagadish Rao Padubidri, Sujogya Kumar Panda, Songhomitra Panda-Jonas, Deepshikha Pande Katare, Leonidas D Panos, Ioannis Pantazopoulos, Paraskevi Papadopoulou, Romil R Parikh, Nicholas Parsons, Roberto Passera, Shankargouda Patil, Shrikant Pawar, Hamidreza Pazoki Toroudi, Umberto Pensato, Mario F P Peres, Simone Perna, Hoang Nhat Pham, Zahra Zahid Piracha, Michael A Piradov, Dimitri Poddighe, Ramesh Poluru, Jalandhar Pradhan, Manya Prasad, Jagadeesh Puvvula, Pankaja Raghav, Fakher Rahim, Amir Masoud Rahmani, Mohammad Rahmanian, Adarsh Raja, Ali Rajabpour Sanati, Mahmoud Mohammed Ramadan, Shakthi Kumaran Ramasamy, Nemanja Rancic, Sowmya J Rao, Devarajan Rathish, Ilari Rautalin, Salman Rawaf, Elrashdy M Moustafa Mohamed Redwan, Muhammad Riaz, Jefferson Antonio Buendia Rodriguez, Leonardo Roever, Marina Romozzi, Moustaq Karim Khan Rony, Kevin T Root, Aly M A Saad, Cameron John Sabet, Basema Ahmad Saddik, Reihaneh Sadeghian, Umar Saeed, Usman Saeed, Fatemeh Saheb Sharif-Askari, Amirhossein Sahebkar, Zahra Saif, Afeez Abolarinwa Salami, Abdallah M Samy, Gargi Sachin Sarode, Sachin C Sarode, Anudeep Sathyanarayan, Maheswar Satpathy, Siddharthan Selvaraj, Ashenafi Kibret Sendekie, Yashendra Sethi, Allen Seylani, Samiah Shahid, Summaiya Zareen Shaikh, Muhammad Aaqib Shamim, Mehran Shams-Beyranvand, Alfiya Shamsutdinova, Javad Sharifi Rad, Anupam Sharma, Vishal Sharma, Zubeda Begum Sheikh, Mahabalesh Shetty, Pavanchand H Shetty, Premalatha K Shetty, Aminu Shittu, Nathan A Shlobin, Seyed Afshin Shorofi, Sunil Shrestha, Emmanuel Edwar Siddig, Gagandeep Singh, Harmanjit Singh, Jasvinder A Singh, Paramdeep Singh, Puneetpal Singh, Surjit Singh, Shipra Solanki, Soroush Soraneh, Muhammad Haroon Stanikzai, Mark J M Sullman, Katharina S Sunnerhagen, Lukasz Szarpak, Payam Tabaee Damavandi, Manoj Tanwar, Minale Tareke, Mohamad-Hani Temsah, Reem Mohamad Hani Temsah, Masayuki Teramoto, Pugazhenthan Thangaraju, Sathish Thirunavukkarasu, Tenaw Yimer Tiruye, Krishna Tiwari, Vikas Kumar Tiwari, Marcos Roberto Tovani-Palone, Thang Huu Tran, Nguyen Tran Minh Duc, Manjari Tripathi, Samuel Joseph Tromans, Daniel Hsiang-Te Tsai, Aristidis Tsatsakis, Evangelia Eirini Tsermpini, Aniefiok John Udoakang, Muhammad Umair, Bhaskaran Unnikrishnan, Daniele Urso, Jibrin Sammani Usman, Asokan Govindaraj Vaithinathan, Alireza Vakilian, Ravi Prasad Varma, Narayanaswamy Venketasubramanian, Jorge Hugo Villafañe, Manish Vinayak, Theo Vos, Mandaras Tariku Walde, Shu Wang, Yanzhong Wang, Nuwan Darshana Wickramasinghe, Samuel Wiebe, Andrea Sylvia Winkler, Arzu Yiğit, Vahit Yiğit, Mekdes Tigistu Yilma, Dong Keon Yon, Naohiro Yonemoto, Aurora Zanghì, Mohammed G M Zeariya, Zhongyi Zhao, Claire Chenwen Zhong, Magdalena Zielińska, Osama A Zitoun, Sa'ed H Zyoud, and Samer H Zyoud.

Managing the estimation or publications process

Simon I Hay, Ali H Mokdad, and Christopher J L Murray.
