# Supplementary material for: Global, regional, and national burden of epilepsy, 1990–2021: a systematic analysis for the Global Burden of Disease Study 2021
Source: Lancet Public Health. 2025 Feb 24;10(3):e203–27. doi: 10.1016/S2468-2667(24)00302-5 (PMC11876103; doi:10.1016/S2468-2667(24)00302-5)
Supplement: Supplementary appendix 2 [file mmc2.pdf]

THE LANCET
Public Health

## Supplementary appendix 2

This appendix formed part of the original submission and has been peer reviewed. We post it as supplied by the authors.

Supplement to: GBD Epilepsy Collaborators. Global, regional, and national burden of epilepsy, 1990–2021: a systematic analysis for the Global Burden of Disease Study 2021. *Lancet Public Health* 2025; published online Feb 24. https://doi.org/10.1016/S2468-2667(24)00302-5.

// The Lancet Public Health

# The Lancet Public Health

## Supplementary appendix

This appendix formed part of the original submission and has been peer reviewed. We post it as supplied by the authors.

Supplement to: GBD 2021 Epilepsy Collaborators. Global, regional, and national burden of epilepsy, 1990–2021: a systematic analysis for the Global Burden of Disease Study 2021.

Most of the supplementary materials on the study methodology were adapted from the previous GBD publications:


1. Global, regional, and national burden of epilepsy, 1990-2016: a systematic analysis for the Global Burden of Disease Study 2016. *Lancet Neurol*. 2019;18:357-375
2. Naghavi M, Ong KL, Aali A, Ababneh HS, Abate YH, Abbafati C, . . . Murray CJL. Global burden of 288 causes of death and life expectancy decomposition in 204 countries and territories and 811 subnational locations, 1990-2021: a systematic analysis for the Global Burden of Disease Study 2021. *The Lancet*. 2024;403:2100-2132
3. Ferrari AJ, Santomauro DF, Aali A, Abate YH, Abbafati C, Abbastabar H, . . . Murray CJL. Global incidence, prevalence, years lived with disability (YLDs), disability-adjusted life-years (DALYs), and healthy life expectancy (HALE) for 371 diseases and injuries in 204 countries and territories and 811 subnational locations, 1990-2021: a systematic analysis for the Global Burden of Disease Study 2021. *The Lancet*. 2024;403:2133-2161.
4. Steinmetz JD, Seeher KM, Schiess N, Nichols E, Cao B, Servili C, . . . Dua T. Global, regional, and national burden of disorders affecting the nervous system, 1990–2021: a systematic analysis for the Global Burden of Disease Study 2021. *The Lancet Neurology*. 2024;23:344-381
5. Global burden and strength of evidence for 88 risk factors in 204 countries and 811 subnational locations, 1990-2021: a systematic analysis for the Global Burden of Disease Study 2021. *The Lancet*. 2024;403:2162-2203.
6. Schumacher AE, Kyu HH, Aali A, Abbafati C, Abbas J, Abbasgholizadeh R, . . . Murray CJL. Global age-sex-specific mortality, life expectancy, and population estimates in 204 countries and territories and 811 subnational locations, 1950-2021, and the impact of the COVID-19 pandemic: a comprehensive demographic analysis for the Global Burden of Disease Study 2021. *The Lancet*. 2024;403:1989-2056

# Table of contents


**SUMMARY OF GENERAL GLOBAL BURDEN OF DISEASE STUDY METHODS** .......... 3

**DETAILS ON DATA SOURCES** .......... 5

**SOURCES USED FOR EPILEPSY REGRESSIONS** .......... 5

IDIOPATHIC REGRESSION .......... 5
SEVERE REGRESSION .......... 10
TREATED WITHOUT FITS REGRESSION .......... 12
TREATMENT GAP REGRESSION .......... 12

**NON-FATAL ESTIMATES** .......... 16

**CAUSE OF DEATH ENSEMBLE MODEL** .......... 17

**GATHER COMPLIANCE TABLE** .......... 17

**EPILEPSY MORTALITY** .......... 20

INPUT DATA .......... 20
MODELLING STRATEGY .......... 20

**EPILEPSY IMPAIRMENT** .......... 21

CASE DEFINITION .......... 21
INPUT DATA .......... 22
*Disability weights* .......... *22*
*Modelling strategy* .......... *22*

**DEFINITION OF GBD SUPER-REGIONS AND REGIONS** .......... 24

**TABLES OF THE EPILEPSY BURDEN ESTIMATES** .......... 25

TABLE 1. ABSOLUTE NUMBER, WITH 95% UNCERTAINTY INTERVALS (UI), OF DEATHS, DALYS, AND INCIDENCE ASSOCIATED WITH IDIOPATHIC EPILEPSY IN 2021 AND PERCENTAGE CHANGE IN THE AGE-STANDARDISED METRICS FOR 1990–2021 BY SEVEN GBD SUPER-REGIONS, 21 GBD REGIONS, AND 204 COUNTRIES/TERRITORIES .......... 25
TABLE 2. AGE-STANDARDISED INCIDENCE, PREVALENCE, DEATH, AND DALY RATES OF IDIOPATHIC EPILEPSY PER 100,000 PEOPLE BY SEVEN GBD SUPER-REGIONS, 21 GBD REGIONS, AND 204 COUNTRIES/TERRITORIES, BOTH SEXES, 2021 .36
TABLE 3. AGE-STANDARDISED INCIDENCE, DEATHS, AND DALYS OF IDIOPATHIC EPILEPSY PER 100,000 PEOPLE (WITH 95% UI) IN 2021 BY SEX AND WORLD BANK COUNTRY INCOME LEVEL .......... 50

**FIGURES** .......... 51

APPENDIX FIGURE 1. AGE-STANDARDISED PREVALENCE OF EPILEPSY FROM IDIOPATHIC AND SECONDARY EPILEPSY COMBINED PER 100,000 PEOPLE (WITH 95% UI) BY WORLD BANK COUNTRY INCOME LEVEL AND SOCIO-DEMOGRAPHIC INDEX (SDI) QUINTILES, BOTH SEXES, 1990–2021 .......... 51
FIGURE 2. AGE-STANDARDISED INCIDENCE, PREVALENCE, DEATH, AND DALY RATES OF IDIOPATHIC EPILEPSY PER 100,000 PEOPLE IN THE WORLD BY SEX, 1990–2021 .......... 52
FIGURE 3. AGE-STANDARDISED IDIOPATHIC EPILEPSY INCIDENCE, DEATH, AND DALY RATES PER 100,000 PEOPLE (WITH 95% UI) BY WORLD BANK COUNTRY INCOME LEVEL FROM 1990 TO 2021, BOTH SEXES .......... 53
FIGURE 4. AGE-STANDARDISED IDIOPATHIC EPILEPSY INCIDENCE, DEATH, AND DALY RATES PER 100,000 PEOPLE (WITH 95% UI) BY SOCIO-DEMOGRAPHIC INDEX QUINTILE FROM 1990 TO 2021, BOTH SEXES .......... 54

**REFERENCES** .......... 55

## Summary of general Global Burden of Disease study methods

A Cause of Death Ensemble model can find optimal coverage of input data by specific combinations of predictive covariates through the use of out-of-sample predictive validity testing. The input data for the Cause of Death Ensemble model included both vital registration (3460 site-years) and verbal autopsy data (54 site-years). A site-year is a unique combination of calendar year, location, and data source. The Cause of Death Ensemble model for epilepsy also utilised predictive covariates for pigs (per capita), proxy for neurocysticercosis infection, SEV scalar: epilepsy, mean systolic blood pressure (mmHg), Healthcare Access and Quality Index, mean body-mass index, mean serum total cholesterol (mmol/L), cumulative cigarettes (10 years), cumulative cigarettes (5 years), education (years per capita), log LDI (per capita), and Socio-demographic Index. More information on calculations can be found in the appendix and in the GBD 2021 risk factor overview paper.[1]

The Institute for Health Metrics and Evaluation, with a growing collaboration of scientists, produces annual updates of the Global Burden of Disease study. Estimates span the period from 1990 to the most recent completed year. Annual updates allow incorporation of new data and method improvements to ensure that the most up-to-date information is available to policy makers in a timely fashion to help make resource allocation decisions.

In the methods section, we present a summary of the general methods of the Global Burden of Disease (GBD). The guiding principle of GBD is to assess health loss due to mortality and disability comprehensively, where we define disability as any departure from full health. In GBD 2021, estimates were made for 204 countries and territories and 579 subnational locations, for 21 years starting from 1990, for 23 age groups and both sexes. Deaths were estimated for 264 diseases and injuries, while prevalence and incidence were estimated for 328 diseases and injuries. In order to allow meaningful comparisons between deaths and non-fatal disease outcomes as well as between diseases, the data on deaths and prevalence are summarised in a single indicator, the disability-adjusted life-year (DALY). DALYs are the sum of years of life lost (YLLs) and years lived with disability (YLDs). YLLs are estimated as the multiplication of counts of death and a standard, "ideal", remaining life expectancy at the age of death. The standard life expectancy is derived from the lowest observed mortality rates in any population in the world greater than 5 million.[2] YLDs are estimated as the product of prevalence of individual consequences of disease (or "sequelae") times a disability weight that quantifies the relative severity of a sequela as a number between zero (representing "full health") and 1 (representing death). Disability weights have been estimated in nine population surveys and an open-access internet survey in which respondents are asked to choose the "healthier"[2] between random pairs of health states that are presented with a short description of the main features.

All-cause mortality rates are estimated from vital registration data in countries with complete coverage. For other countries, the probabilities of death before age 5 and between ages 15 and 60 are estimated from censuses and surveys asking mothers to provide a history of children ever born and those still alive, and surveys asking adults about siblings who are alive or have passed away. Using model life tables, these probabilities of death are transformed into age-specific death rates by location, year, and sex. GBD has collated a large database of cause of death data from vital registrations and verbal autopsy surveys in which relatives are asked a standard set of questions to ascertain the likely cause of death, supplemented with police and mortuary data for injury deaths in countries with no other data. For countries with vital registration data, the completeness is assessed with demographic methods based on comparing recorded deaths with population counts between two successive censuses. The cause of death information is provided in a large number of different classification systems based on versions of the International Classification of Diseases or bespoke classifications in some countries. All data are mapped into the disease and injury categories of GBD. All classification systems contain codes that are less informative because they lack a specific diagnosis (e.g., unspecified cancer) or refer to codes that cannot be underlying cause of death (e.g., low back pain or senility) or are intermediate causes (e.g., heart failure or sepsis). Such deaths are redistributed to more precise

underlying causes of death.[4] After these redistributions and corrections for under-registration, the data are analysed in CODEm (Cause of Death Ensemble model), a highly systematised tool that runs many different models on the same data and chooses an ensemble of models that best reflects all the available input data. Models are chosen with variations in the statistical approach ("mixed effects" of spatiotemporal Gaussian process regression), in the unit of analysis (rates or cause fractions), and the choice of predictive covariates. The statistical performance of all models is tested by holding out 30% of the data and checking how well a model covers the data that were held out. To enforce consistency from CODEm, the sum of all cause-specific mortality rates is scaled to that of the all-cause mortality rates in each age, sex, location, and year category.

Our Global Health Data Exchange (GHDx, http://ghdx.healthdata.org/) is the largest repository of health data globally. We first set a reference case definition and/or study method that best quantifies each disease or injury or consequence thereof. If there is evidence of a systematic bias in data that used different case definitions or methods compared to reference data, we adjust those datapoints to reflect what its value would have been if measured as the reference. This is a necessary step if one wants to use all data pertaining to a particular quantity of interest rather than choosing a small subset of data of the highest quality only. DisMod-MR 2.1, a Bayesian meta-regression tool, is our main method of analysing non-fatal data. It is designed as a geographical cascade where a first model is run on all the world's data, which produces an initial global fit and estimates coefficients for predictor variables and the adjustments for alternative study characteristics. The global fit adjusted by the values of random effects for each of seven GBD super-regions, the coefficients on sex and country predictors, are passed down as data to a model for each super-region together with the input data for that geography. The same steps are repeated going from super-region to 21 region fits and then to 204 fits by country and, where applicable, a further level down to subnational units. Below the global fit, all models are run separately by sex and for six time periods: 1990, 1995, 2000, 2005, 2010, and 2019 and 2021. During each fit, all data on prevalence, incidence, remission (i.e., cure rate) and mortality are forced to be internally consistent. For most diseases, the bulk of data on prevalence or incidence is at the disease level, with fewer studies providing data on the proportions of cases of disease in each of the sequelae defined for the disease. The proportions in each sequela are pooled using DisMod-MR 2.1 or meta-analysis or derived from analyses of patient-level datasets. The multiplication of prevalent cases for each disease sequela and the appropriate disability weight produces YLD estimates that do not yet take into account comorbidity. To correct for comorbidity, these data are used in a simulation to create hypothetical individuals in each age, sex, location, and year combination who experience no, one, or multiple sequelae simultaneously. We assume that disability weights are multiplicative rather than additive as this avoids assigning a combined disability weight value in any individual to exceed 1, i.e., be worse than a "year lost due to death". This comorbidity adjustment leads to an average scaling down of disease-specific YLDs ranging from about 2% in young children up to 17% in oldest ages.

All our estimates of causes of death are categorical: each death is assigned to a single underlying cause. This has the attractive property that all estimates add to 100%. For risks, we use a different, "counterfactual" approach, i.e., answering the question: "what would the burden have been if the population had been exposed to a theoretical minimum level of exposure to a risk?" Thus, we need to define what level of exposure to a risk factor leads to the lowest amount of disease. We then analyse data on the prevalence of exposure to a risk and derive relative risks for any risk-outcome pair for which we find sufficient evidence of a causal relationship. Prevalence of exposure is estimated in DisMod-MR 2.1, using spatiotemporal Gaussian process regression, or from satellite imagery in the case of ambient air pollution. Relative risk data are pooled using meta-analysis of cohort, case-control, and/or intervention studies. For each risk and outcome pair, we evaluate the evidence and judge if the evidence falls into the categories of "convincing" or "probable" as defined by the World Cancer Research Fund.[5] From the prevalence and relative risk results, population attributable fractions are estimated relative to the theoretical minimum risk exposure level (TMREL). When we aggregate estimates for clusters of risks, e.g., metabolic or behavioural risks, we use a multiplicative function rather than simple addition and take into account how much of each risk is mediated through another

risk. For instance, some of the risk of high body-mass index is directly onto stroke as an outcome, but much of its impact is mediated through high blood pressure, high cholesterol, or high fasting plasma glucose, and we would not want to double count the mediated effects when we estimate aggregates across risk factors.[3]

Uncertainty is propagated through each computation step by sampling 500 draws at each prevalence, death, YLL, YLD, or DALY estimate and performing aggregations across causes and locations at the level of each of the 1,000 values for all intermediate steps in the calculation. The lower and upper bounds of the 95% uncertainty interval are the 25$^{th}$ and 975$^{th}$ values of the ordered 1000 values. For all age-standardised rates, GBD uses a standard population calculated as the non-weighted average across all countries of the percentage of the population in each five-year age group for the years 2010 to 2035 from the United Nations Population Division's World Population Prospects (2022 revision).[4,5]

GBD uses a composite indicator of sociodemographic development, SDI, which reflects the geometric mean of normalised values of a location's income per capita, the average years of schooling in the population 15 and over, and the total fertility rate. Countries and territories are grouped into five quintiles of high, high-middle, middle, low-middle, and low SDI based on their 2016 values.[6]

## Details on data sources

Overall, we had 343 unique sources of prevalence data for 20 of 21 world regions, 86 unique incidence regions for 15 of 21 world regions, and three unique sources on remission for three of 21 world regions. There are several locations where we are lacking data, primarily eastern Europe and Russia.

## Sources used for epilepsy regressions

### Idiopathic regression


| |
|---|
| Huang DH, Zheng JO, Chen J, Yu L. Treatment gaps of epilepsy and retention rates of sodium valproate in rural Guangxi, China. *Genet Mol Res*. 2014; 13(3): 6202-12. |
| Kong ST, Ho CS, Ho PC, Lim SH. Prevalence of drug resistant epilepsy in adults with epilepsy attending a neurology clinic of a tertiary referral hospital in Singapore. *Epilepsy Res*. 2014; 108(7): 1253-62. |
| Choudhary A, Gulati S, Sagar R, Kabra M, Sapra S. Behavioral comorbidity in children and adolescents with epilepsy. *J Clin Neurosci*. 2014; 21(8): 1337-40. |
| Stefan H, May TW, Pfafflin M, Brandt C, Furatsch N, Schmitz B, Wandschneider B, Kretz R, Runge U, Geithner J, Karakizlis C, Rosenow F, Kerling F. Epilepsy in the elderly: comparing clinical characteristics with younger patients. *Acta Neurol Scand*. 2014; 129(5): 283-93. |
| Kim DW, Lee SY, Chung SE, Cheong HK, Jung KY. Clinical characteristics of patients with treated epilepsy in Korea: a nationwide epidemiologic study. *Epilepsia*. 2014; 55(1): 67-75. |
| Pandey S, Singhi P, Bharti B. Prevalence and treatment gap in childhood epilepsy in a north Indian city: a community-based study. *J Trop Pediatr*. 2014; 60(2): 118-23. |
| Joseph N, Kumar GS, Nelliyanil M. Pattern of seizure cases in tertiary care hospitals in Karnataka state of India. *Ann Indian Acad Neurol*. 2013; 16(3): 347-51. |
| Torres-Ferrus M, Toledo M, Gonzalez-Cuevas M, Sero-Ballesteros L, Santamarina E, Raspall-Chaure M, Sueiras-Gil M, Cambrodi-Masip R, Sarria S, Alvarez-Sabin J, Salas-Puig J. [Aetiology and treatment of epilepsy in a series of 1,557 patients]. *Rev Neurol*. 2013; 57(7): 306-12. |
| Tanaka A, Akamatsu N, Shouzaki T, Toyota T, Yamano M, Nakagawa M, Tsuji S. Clinical characteristics and treatment responses in new-onset epilepsy in the elderly. *Seizure*. 2013; 22(9): 772-5. |

Chong J, Hesdorffer DC, Thurman DJ, Lopez D, Harris RB, Hauser WA, Labiner ET, Velarde A, Labiner DM. The prevalence of epilepsy along the Arizona-Mexico border. *Epilepsy Res*. 2013; 105(2-Jan): 206-15.

Nickels KC, Grossardt BR, Wirrell EC. Epilepsy-related mortality is low in children: a 30-year population-based study in Olmsted County, MN. *Epilepsia*. 2012; 53(12): 2164-71.

Hunter E, Rogathi J, Chigudu S, Jusabani A, Jackson M, McNally R, Gray W, Whittaker RG, Iqbal A, Birchall D, Aris E, Walker R. Prevalence of active epilepsy in rural Tanzania: a large community-based survey in an adult population. *Seizure*. 2012; 21(9): 691-8.

Garcia-Martin G, Perez-Errazquin F, Chamorro-Munoz MI, Romero-Acebal M, Martin-Reyes G, Dawid-Milner MS. Prevalence and clinical characteristics of epilepsy in the South of Spain. *Epilepsy Res*. 2012; 102(1-2): 100-8.

Zhao Y-H, Zhang Q, Long N, Yang C, Hong J, Mu L, Zhou D. Prevalence of epilepsy and alcohol-related risk in Zayul County, Tibet Autonomous Region in China: an initial survey. *Epilepsy Behav*. 2010; 19(4): 635-8.

Suastegui R, Gutierrez J, Ramos R, Bouchan S, Navarrete H, Ruiz J, Plascencia N, Jauri S, Leon C, Castillo V, Ojeda EA. [Clinical characteristics of the late-onset epilepsy in Mexico to the beginning of the new millennium: 455 cases]. *Rev Invest Clin*. 2009; 61(5): 354-63.

Tse E, Hamiwka L, Sherman EM, Wirrell E. Social skills problems in children with epilepsy: prevalence, nature and predictors. *Epilepsy Behav*. 2007; 11(4): 499-505.

Dura Trave T, Yoldi Petri ME. [Epilepsy and epileptic syndromes among primary school children (6-12 years)]. *An Pediatr* (Barc). 2007; 66(1): 11-6.

Oun A, Haldre S, Magi M. Use of antiepileptic drugs in Estonia: an epidemiologic study of adult epilepsy. *Eur J Neurol*. 2006; 13(5): 465-70.

Arruda WO. Etiology of epilepsy. A prospective study of 210 cases. *Arq Neuropsiquiatr*. 1991;49(3): 251-4.

Valença MM, Valença LP. [Etiology of the epileptic seizures in Recife city, Brazil: study of 249 patients]. *Arq Neuropsiquiatr*. 2000; 58(4): 1064-72.

Kwong KL, Chak WK, Wong SN, So KT. Epidemiology of childhood epilepsy in a cohort of 309 Chinese children. *Pediatr Neurol*. 2001; 24(4): 276-82.

Ng KK, Ng PW, Tsang KL, Hong Kong Epilepsy Study Group. Clinical characteristics of adult epilepsy patients in the 1997 Hong Kong epilepsy registry. *Chin Med J* (Engl). 2001; 114(1): 84-7.

Hui AC, Wong A, Wong HC, Man BL, Au-Yeung KM, Wong KS. Refractory epilepsy in a Chinese population. *Clin Neurol Neurosurg*. 2007; 109(8): 672-5.

Fong GCY, Mak W, Cheng TS, Chan KH, Fong JKY, Ho SL. A prevalence study of epilepsy in Hong Kong. *Hong Kong Med J*. 2003; 9(4): 252-7.

Pi X, Cui L, Liu A, Zhang J, Ma Y, Liu B, Cai C, Zhu C, Zhou T, Chen J, Zhou Z, Wang C, Li L, Li S, Wu J, Xiao B. Investigation of prevalence, clinical characteristics and management of epilepsy in Yueyang city of China by a door-to-door survey. *Epilepsy Res*. 2012; 101(1-2): 129-34.

Dechef G. Notions sur l'épidémiologie de l'épilepsie au Congo (Kinshasa). *Afr J Med Sci*. 1970;1(3): 309-14.

Chaves-Sell F, Dubuisson-Schonemberg V. [Profile of epilepsy in a neurology clinic in Costa Rica]. *Rev Neurol*. 2001; 33(5): 411-13.

Pascual López MA, Pascual Gispert J, Rodr¡guez Rivera L, Rojas Ochoa F, Tejeiros A. [Epilepsy: epidemiological study in a child population]. *Bol Med Hosp Infant Mex*. 1980; 37(4): 811-21.

Arteaga-Rodríguez C, Ramírez-Chávez J, Rodríguez-Rivera L, Moréra-Mendez F, Hernández-Fustes OJ. Aetiological factors of the epilepsies. *Rev Neurol*. 1998; 27: 427-30.

Nieto Barrera M. [Neuroepidemiology of epilepsies]. *An Esp Pediatr*. 1988; 29(Supp 33): 59-63.

Dur-Trav, T, Yoldi-Petri ME, Gallinas-Victoriano F. Incidence of epilepsies and epileptic syndromes among children in Navarre, Spain: 2002 through 2005. *J Child Neurol*. 2008; 23(8): 878-82.

Rakitin A, Liik M, Oun A, Haldre S. Mortality risk in adults with newly diagnosed and chronic epilepsy: a population-based study. *Eur J Neurol*. 2011; 18(3): 465-70.

Beilmann A, Napa A, Hämarik M, Sööt A, Talvik I, Talvik T. Incidence of childhood epilepsy in Estonia. *Brain Dev*. 1999; 21(3): 166-74.

Tekle-Haimanot R, Forsgren L, Ekstedt J. Incidence of epilepsy in rural central Ethiopia. *Epilepsia*. 1997; 38(5): 541-6.

Rantala H, Ingalsuo H. Occurrence and outcome of epilepsy in children younger than 2 years. *J Pediatr*. 1999; 135(6): 761-4.

Lomidze G, Kasradze S, Kvernadze D, Okujava N, Toidze O, de Boer HM, Dua T, Sander JW. The prevalence and treatment gap of epilepsy in Tbilisi, Georgia. *Epilepsy Res*. 2012; 98(2-3): 123-9.

Medina MT, Durón RM, Martínez L, Osorio JR, Estrada AL, Zúniga C, Cartagena D, Collins JS, Holden KR. Prevalence, incidence, and etiology of epilepsies in rural Honduras: the Salama Study. *Epilepsia*. 2005; 46(1): 124-31.

Goel D, Dhanai JS, Agarwal A, Mehlotra V, Saxena V. Neurocysticercosis and its impact on crude prevalence rate of epilepsy in an Indian community. *Neurol India*. 2011; 59(1): 37-40.

Olafsson E, Hauser WA. Prevalence of epilepsy in rural Iceland: a population-based study. *Epilepsia*. 1999; 40(11): 1529-34.

Olafsson E, Ludvigsson P, Gudmundsson G, Hesdorffer D, Kjartansson O, Hauser WA. Incidence of unprovoked seizures and epilepsy in Iceland and assessment of the epilepsy syndrome classification: a prospective study. *Lancet Neurol*. 2005; 4(10): 627-34.

Battaglia D, Randò T, Deodato F, Bruccini G, Baglio G, Frisone MF, Pantò T, Tortorella G, Guzzetta F. Epileptic disorders with onset in the first year of life: neurological and cognitive outcome. *Eur J Paediatr Neurol*. 1999; 3(3): 95-103.

Gallitto G, Serra S, La Spina P, Postorino P, Lagan… A, Tripodi F, Gangemi S, Calabrò S, Savica R, Di Perri R, Beghi E, Musolino R. Prevalence and characteristics of epilepsy in the Aeolian islands. *Epilepsia*. 2005; 46(11): 1828-35.

Mwinzi SMG, Ruberti FR, Stewart JD. Epilepsy in the Kenyan Africa. *Med Afr Noire*. 1976; 23: 331-334.

Feksi AT, Kaamugisha J, Sander JW, Gatiti S, Shorvon SD. Comprehensive primary health care antiepileptic drug treatment programme in rural and semi-urban Kenya. ICBERG (International Community-based Epilepsy Research Group). *Lancet*. 1991; 337(8738): 406-9.

Sridharan R, Radhakrishnan K, Ashok PP, Mousa ME. Epidemiological and clinical study of epilepsy in Benghazi, Libya. *Epilepsia*. 1986; 27(1): 60-5.

Andriantseheno LM, Andrianasy TF. The features of epilepsy in the Malagasy: a hospital study on 213 cases from the North Western part of Madagascar. *Afr J Neurol Sci*. 1997; 16: 28-33.

Medina MT, Rosas E, Rubio-Donnadieu F, Sotelo J. Neurocysticercosis as the main cause of late-onset epilepsy in Mexico. *Arch Intern Med*. 1990; 150(2): 325-7.

| |
|---|
| Uuriintuya M, Ulziibayar D, Bayarmaa D. Epilepsy in Mongolia. *Neurology Asia*. 2007; 12: 61-63. |
| Dada TO. Epilepsy in Lagos, Nigeria. *Afr J Med Sci*. 1970; 1: 161-184. |
| Danesi MA, Oni K. Features of partial epilepsy in Nigerians: a 3 year clinical and electroencephalographic study of 282 cases seen at the Lagos University Teaching Hospital. *Afr J Neurol Sci*. 1983; 2: 1-6. |
| Danesi MA. Acquired aetiological factors in Nigerian epileptics (an investigation of 378 patients). *Trop Geogr Med*. 1983; 35(3): 293-297. |
| Ojuawo A, Joiner KT. Childhood epilepsy in Ilorin, Nigeria. *East Afr Med J*. 1997; 74(2): 72-75. |
| Breteler M, de la Court A, Meinardi H, Hauser WA, Grobbee D, Hofman A. Prevalence of epilepsy in the elderly: the Rotterdam Study. *Epilepsia*. 1996; 37(2): 141-7. |
| Gaffo AL, Guillén-Pinto D, Campos-Olazábal P, Burneo JG. [Cysticercosis as the main cause of partial seizures in children in Peru]. *Rev Neurol*. 2004; 39(10): 924-6. |
| Collomb H, Girard PL, Konate S. L'épilepsie en milieu hospitalier à Dakar. *Med Afr Noire*. 1976; 23: 299-304. |
| Lee WL, Low PS, Murugasu B, Rajan U. Epidemiology of epilespy in Singapore children. *Neurol J Southeast Asia*. 1997; 2: 31-35. |
| Larsson K, Eeg-Olofsson O. A population based study of epilepsy in children from a Swedish county. *Eur J Paediatr Neurol*. 2006; 10(3): 107-13. |
| Adelöw C, Andell E, Amark P, Andersson T, Hellebro E, Ahlbom A, Tomson T. Newly diagnosed single unprovoked seizures and epilepsy in Stockholm, Sweden: First report from the Stockholm Incidence Registry of Epilepsy (SIRE). *Epilepsia*. 2009; 50(5): 1094-101. |
| Velioglu SK, Bakirdemir M, Can G, Topbas M. Prevalence of epilepsy in northeast Turkey. *Epileptic Disord*. 2010; 12(1): 22-37. |
| Matuja WB. Aetological factors in Tanzanian epileptics. *East Afr Med J*. 1989; 66(5): 343-348. |
| Dent W, Helbok R, Matuja WBP, Scheunemann S, Schmutzhard E. Prevalence of active epilepsy in a rural area in South Tanzania: a door-to-door survey. *Epilepsia*. 2005; 46(12): 1963-9. |
| Tuan NA, Cuong LQ, Allebeck P, Chuc NTK, Persson HE, Tomson T. The prevalence of epilepsy in a rural district of Vietnam: a population-based study from the EPIBAVI project. *Epilepsia*. 2008; 49(9):1634-7. |
| Chuke PO, Muras J. Experience in epilepsy in Lusaka. *Med J Zambia*. 1977; 11: 65-70. |
| Levy LF. Epilepsy in Rhodesia, Zambia, and Malawi. *Afr J Med Sci*. 1970; 1: 291-203. |
| Khedr EM, Shawky OA, Ahmed MA, Elfetoh NA, Al Attar G, Ali AM, Kandil MR, Farweez H. A community based epidemiological study of epilepsy in Assiut Governorate/Egypt. *Epilepsy Res*. 2013; 103(3-Feb): 294-302. |
| Al Rajeh S, Awada A, Bademosi O, Ogunniyi A. The prevalence of epilepsy and other seizure disorders in an Arab population: a community-based study. *Seizure*. 2001; 10(6): 410-4. |
| Ablah E, Hesdorffer DC, Liu Y, Paschal AM, Hawley S, Thurman D, Hauser WA. Prevalence of epilepsy in rural Kansas. *Epilepsy Res*. 2014; 108(4): 792-801. |
| El-Tallawy HN, Farghaly WM, Shehata GA, Abdel-Hakeem NM, Rageh TA, Abo-Elftoh NA, Hegazy A, Badry R. Epidemiology of epilepsy in New Valley Governorate, Al Kharga District, Egypt. *Epilepsy Res*. 2013; 104(2-Jan): 167-74. |

| |
|---|
| Saha, SP, Bhattachrya, S, Roy, BK, Basu, A, Roy, T, Maity, B, Das, SK. (2008). A prospective incidence study of epilepsy in a rural community of West-Bengal, India. *Neurology Asia*, 13, 41-48. |
| Josipovic-Jelic Z, Sonicki Z, Soljan I, Demarin V, Collaborative Group for Study of Epilepsy Epidemiology in Sibenik-Knin County, Croatia. Prevalence and socioeconomic aspects of epilepsy in the Croatian county of Sibenik-Knin: community-based survey. *Epilepsy Behav*. 2011; 20(4): 686-90. |
| Gaily E, Lommi M, Lapatto R, Lehesjoki AE. Incidence and outcome of epilepsy syndromes with onset in the first year of life: A retrospective population-based study. *Epilepsia*. 2016; nan. |
| Nilsson G, Fernell E, Arvidsson T, Neville B, Olsson I, Gillberg C. Prevalence of Febrile Seizures, Epilepsy, and Other Paroxysmal Attacks in a Swedish Cohort of 4-Year-Old Children. *Neuropediatrics*. 2016; nan. |
| El-Tallawy HN, Farghaly WM, Rageh TA, Shehata GA, Metwally NA, Badry R, Sayed MA, Abdelwarith AM, Kandil MR, Hamed MA, Mohamed KO, Tohamy AM. Spectrum of epilepsy - prevalence, impact, and treatment gap: an epidemiological study from Al-Quseir, Egypt. *Neuropsychiatr Dis Treat*.2016; 12: 1111-8. |
| Serrano-Castro PJ, Mauri-Llerda JA, Hernandez-Ramos FJ, Sanchez-Alvarez JC, Parejo-Carbonell B, Quiroga-Subirana P, Vazquez-Gutierrez F, Santos-Lasaosa S, Mendez-Lucena C, Redondo-Verge L, Tejero-Juste C, Morandeira-Rivas C, Sancho-Rieger J, Matias-Guiu J. Adult Prevalence of Epilepsy in Spain: EPIBERIA, a Population-Based Study. *Scientific World Journal*. 2015; 2015: 602710. |
| Hara HS, Gupta A, Singh M, Raj R, Singh H, Pawar G, Hara PK, Singh J. Epilepsy in Punjab (India): A Population-Based Epidemiologic Study. *Neuroepidemiology*. 2015; 45(4): 273-81. |
| Hashem S, Al-Kattan M, Ibrahim SY, Shalaby NM, Shamloul RM, Farrag M. Epilepsy prevalence in Al-Manial Island, Egypt. A door-to-door survey. *Epilepsy Res*. 2015; 117: 133-7. |
| Fawi G, Khedr EM, El-Fetoh NA, Thabit MN, Abbass MA, Zaki AF. Community-based epidemiological study of epilepsy in the Qena governorate in Upper Egypt, a door-to-door survey. *Epilepsy Res*. 2015; 113: 68-75. |
| San-Juan D, Alvarado-Leon S, Barraza-Diaz J, Davila-Avila NM, Ruiz AH, Anschel DJ. Prevalence of epilepsy, beliefs and attitudes in a rural community in Mexico: A door-to-door survey. *Epilepsy Behav*. 2015; 46: 140-3. |
| Syvertsen M, Nakken KO, Edland A, Hansen G, Hellum MK, Koht J. Prevalence and etiology of epilepsy in a Norwegian county-A population based study. *Epilepsia*. 2015; 56(5): 699-706. |
| Bourrous M, Elibrahimi I, Draiss G, Safini F, Amine M, Bouskraoui M. [Characteristics of the children with epilepsy followed in the Marrakech University Hospital]. *Rev Neurol* (Paris). 2010;166(11): 921-6. |
| Wright J, Pickard N, Whitfield A, Hakin N. A population-based study of the prevalence, clinical characteristics and effect of ethnicity in epilepsy. *Seizure*. 2000; 9(5): 309-13. |
| Muir TM, Bradley A, Wood SF, Murray GD, Brodie MJ. An audit of treated epilepsy in Glasgow. West of Scotland Epilepsy Research Group. *Seizure*. 1996; 5(1): 41-6. |
| Aziz H, Güvener A, Akhtar SW, Hasan KZ. Comparative epidemiology of epilepsy in Pakistan and Turkey: population-based studies using identical protocols. *Epilepsia*. 1997; 38(6): 716-22. |
| Tran D-S, Odermatt P, Le T-O, Huc P, Druet-Cabanac M, Barennes H, Strobel M, Preux P-M. Prevalence of epilepsy in a rural district of central Lao PDR. *Neuroepidemiology*. 2006; 26(4): 199-206. |
| Okuma T, Kumashiro H. Natural history and prognosis of epilepsy: report of a multi-institutional study in Japan. The group for the study of prognosis of epilepsy in Japan. *Epilepsia*. 1981; 22(1): 35-53. |

| |
|---|
| Manonmani V, Tan CT. A study of newly diagnosed epilepsy in Malaysia. *Singapore Med J*. 1999; 40(1): 32-5. |
| Andriantseheno LM, Ralaizandriny D. Prévalence communautaire de l'épilepsie chez les Malgaches. *Epilepsies*. 2004; 16(2): 83-6. |
| Farnarier G, Diop S, Coulibaly B, Arborio S, Dabo A, Diakite M, Traore S, Banou A, Nimaga K, Vaz T, Doumbo O. [Onchocerciasis and epilepsy. Epidemiological survey in Mali]. *Med Trop* (Mars).2000; 60(2): 151-5. |
| Kun LN, Ling LW, Wah YW, Lian TT. Epidemiologic study of epilepsy in young Singaporean men. *Epilepsia*. 1999; 40(10): 1384-7. |


### Severe regression


| |
|---|
| Choudhary A, Gulati S, Sagar R, Kabra M, Sapra S. Behavioral comorbidity in children and adolescents with epilepsy. *J Clin Neurosci*. 2014; 21(8): 1337-40. |
| Garcia-Martin G, Perez-Errazquin F, Chamorro-Munoz MI, Romero-Acebal M, Martin-Reyes G, Dawid-Milner MS. Prevalence and clinical characteristics of epilepsy in the South of Spain. *Epilepsy Res*. 2012; 102(1-2): 100-8. |
| Zhao Y-H, Zhang Q, Long N, Yang C, Hong J, Mu L, Zhou D. Prevalence of epilepsy and alcohol-related risk in Zayul County, Tibet Autonomous Region in China: an initial survey. *Epilepsy Behav*. 2010; 19(4): 635-8. |
| Tse E, Hamiwka L, Sherman EM, Wirrell E. Social skills problems in children with epilepsy: prevalence, nature and predictors. *Epilepsy Behav*. 2007; 11(4): 499-505. |
| Debouverie M, Kabore J, Dumas M, Weber M, Duboz P, Vaugelade J. Epidemiology of Epilepsy in Burkina Faso. In: Dumas M, Giordano C, Gentilini M, Chieze F, editors. *Neurologie Tropicale*. Paris, France: John Libbey Eurotext, 1993. 57-61. |
| Kwong KL, Chak WK, Wong SN, So KT. Epidemiology of childhood epilepsy in a cohort of 309 Chinese children. *Pediatr Neurol*. 2001; 24(4): 276-82. |
| Zhao Y, Zhang Q, Tsering T, Sangwan, Hu X, Liu L, Shang H, Chen Q, Liu Y, Yang X, Wang W, Li S, Wu J, Sander JW, Zhou D. Prevalence of convulsive epilepsy and health-related quality of life of the population with convulsive epilepsy in rural areas of Tibet Autonomous Region in China: an initial survey. *Epilepsy Behav*. 2008; 12(3): 373-81. |
| Pascual López MA, Pascual Gispert J, Rodríguez Rivera L, Rojas Ochoa F, Tejeiros A. [Epilepsy: epidemiological study in a child population]. *Bol Med Hosp Infant Mex*. 1980; 37(4): 811-21. |
| Del Brutto OH, Santibáñez R, Idrovo L, Rodrìguez S, Díaz-Calderón E, Navas C, Gilman RH, Cuesta F, Mosquera A, Gonzalez AE, Tsang VCW, García HH. Epilepsy and neurocysticercosis in Atahualpa: a door-to-door survey in rural coastal Ecuador. *Epilepsia*. 2005; 46(4): 583-7. |
| Cruz-Campos GA, Baquero-Toledo M. [Epilepsies in an outpatient setting: a study of 150 cases]. *Rev Neurol*. 2000; 30(12): 1108-12. |
| Josipovic-Jelic Z, Sonicki Z, Soljan I, Demarin V, Collaborative Group for Study of Epilepsy Epidemiology in Sibenik-Knin County, Croatia. Prevalence and socioeconomic aspects of epilepsy in the Croatian county of Sibenik-Knin: community-based survey. *Epilepsy Behav*. 2011; 20(4): 686-90. |
| Singh A, Kaur A. Epilepsy in rural Haryana – prevalence and treatment seeking behaviour. *J Indian Med Assoc*. 1997; 95(2): 37-47. |

| |
|---|
| Cornaggia CM, Canevini MP, Christe W, Giuccioli D, Facheris MA, Sabbadini M, Canger R. Epidemiologic survey of epilepsy among Army draftees in Lombardy, Italy. *Epilepsia*. 1990; 31(1): 27-32. |
| Okuma T, Kumashiro H. Natural history and prognosis of epilepsy: report of a multi-institutional study in Japan. The group for the study of prognosis of epilepsy in Japan. *Epilepsia*. 1981; 22(1): 35-53. |
| Feksi AT, Kaamugisha J, Gatiti S, Sander JW, Shorvon SD. A comprehensive community epilepsy programme: the Nakuru project. *Epilepsy Res*. 1991; 8(3): 252-9. |
| Manonmani V, Tan CT. A study of newly diagnosed epilepsy in Malaysia. *Singapore Med J*. 1999; 40(1): 32-5. |
| Waaler PE, Blom BH, Skeidsvoll H, Mykletun A. Prevalence, classification, and severity of epilepsy in children in western Norway. *Epilepsia*. 2000; 41(7): 802-10. |
| Simms V, Atijosan O, Kuper H, Nuhu A, Rischewski D, Lavy C. Prevalence of epilepsy in Rwanda: a national cross-sectional survey. *Trop Med Int Health*. 2008; 13(8): 1047-53. |
| Mrabet H, Mrabet A, Zouari B, Ghachem R. Health-related quality of life of people with epilepsy compared with a general reference population: a Tunisian study. *Epilepsia*. 2004; 45(7): 838-43. |
| Sahin A, Bolayir E, Sumer H, Tas A, Mollaoglu M, Dener S. Epidemiologic evaluation of epileptic and nonepileptic seizures in Sivas region of middle Anatolia. *Neurol Psych Brain Res*. 2004; 11(2):97-102. |
| Calisir N, Bora I, Irgil E, Boz M. Prevalence of epilepsy in Bursa city center, an urban area of Turkey. *Epilepsia*. 2006; 47(10): 1691-9. |
| Chen R-C, Chang Y-C, Chen TH-H, Wu H-M, Liou H-H. Mortality in adult patients with epilepsy in Taiwan. *Epileptic Disord*. 2005; 7(3): 213-9. |
| Haerer AF, Anderson DW, Schoenberg BS. Prevalence and clinical features of epilepsy in a biracial United States population. *Epilepsia*. 1986; 27(1): 66-75. |
| Nwani PO, Nwosu MC, Asomugha LA, Enwereji KO, Arinzechi EO, Ogunniyi AO. Epidemiology of active epilepsy in a suburban community in Southeast Nigeria: A door-to-door survey. *Niger J Clin Pract*. 2015; 18(4): 527-33. |
| Hart YM, Shorvon SD. The nature of epilepsy in the general population. I. Characteristics of patients receiving medication for epilepsy. *Epilepsy Res*. 1995; 21(1): 43-9. |
| Tidman L, Saravanan K, Gibbs J. Epilepsy in mainstream and special educational primary school settings. *Seizure*. 2003; 12(1): 47-51. |
| Moran NF, Poole K, Bell G, Solomon J, Kendall S, McCarthy M, McCormick D, Nashef L, Sander J, Shorvon SD. Epilepsy in the United Kingdom: seizure frequency and severity, anti-epileptic drug utilization and impact on life in 1652 people with epilepsy. *Seizure*. 2004; 13(6): 425-33. |
| Winkler AS, Kerschbaumsteiner K, Stelzhammer B, Meindl M, Kaaya J, Schmutzhard E. Prevalence, incidence, and clinical characteristics of epilepsy--a community-based door-to-door study in northern Tanzania. *Epilepsia*. 2009; 50(10): 2310-3. |
| Koul R, Razdan S, Motta A. Prevalence and pattern of epilepsy (Lath/Mirgi/Laran) in rural Kashmir, India. *Epilepsia*. 1988; 29(2): 116-22. |

## Treated without fits regression


| |
|---|
| Kong ST, Ho CS, Ho PC, Lim SH. Prevalence of drug resistant epilepsy in adults with epilepsy attending a neurology clinic of a tertiary referral hospital in Singapore. *Epilepsy Res*. 2014; 108(7): 1253-62. |
| Fruchter E, Kapara O, Reichenberg A, Yoffe R, Fono-Yativ O, Kreiss Y, Davidson M, Weiser M. Longitudinal association between epilepsy and schizophrenia: a population-based study. *Epilepsy Behav*. 2014; 31: 291-4. |
| Tanaka A, Akamatsu N, Shouzaki T, Toyota T, Yamano M, Nakagawa M, Tsuji S. Clinical characteristics and treatment responses in new-onset epilepsy in the elderly. *Seizure*. 2013; 22(9): 772-5. |
| Nickels KC, Grossardt BR, Wirrell EC. Epilepsy-related mortality is low in children: a 30-year population-based study in Olmsted County, MN. *Epilepsia*. 2012; 53(12): 2164-71. |
| Picot M-C, Baldy-Moulinier M, Daurès J-P, Dujols P, Crespel A. The prevalence of epilepsy and pharmacoresistant epilepsy in adults: a population-based study in a Western European country. *Epilepsia*. 2008; 49(7): 1230-8. |
| Tse E, Hamiwka L, Sherman EM, Wirrell E. Social skills problems in children with epilepsy: prevalence, nature and predictors. *Epilepsy Behav*. 2007; 11(4): 499-505. |
| Ding D, Hong Z, Chen GS, Dai XY, Wu JZ, Wang WZ, De Boer HM, Sander JW, Prilipko L, Chisholm D. Primary care treatment of epilepsy with phenobarbital in rural China: Cost-outcome analysis from the WHO/ILAE/IBE global campaign against epilepsy demonstration project. *Epilepsia*. 2008; 49(3):535-9. |
| Houinato D, Yemadje L-P, Glitho G, Adjien C, Avode G, Druet-Cabanac M, Preux P-M. Epidemiology of epilepsy in rural Benin: prevalence, incidence, mortality, and follow-up. *Epilepsia*. 2013; 54(4): 757-63. |
| Hunter E, Rogathi J, Chigudu S, Jusabani A, Jackson M, Whittaker RG, Gray W, McNally RJ, Aris E, Mushi D, Walker R. The epilepsy treatment gap in rural Tanzania: A community-based study in adults. *Seizure*. 2016; 36: 49-56. |
| Kun LN, Ling LW, Wah YW, Lian TT. Epidemiologic study of epilepsy in young Singaporean men. *Epilepsia*. 1999; 40(10): 1384-7. |


## Treatment gap regression


| |
|---|
| Huang DH, Zheng JO, Chen J, Yu L. Treatment gaps of epilepsy and retention rates of sodium valproate in rural Guangxi, China. *Genet Mol Res*. 2014; 13(3): 6202-12. |
| Pandey S, Singhi P, Bharti B. Prevalence and treatment gap in childhood epilepsy in a north Indian city: a community-based study. *J Trop Pediatr*. 2014; 60(2): 118-23. |
| Farghaly WM, El-Tallawy HN, Rageh TA, Mohamed EM, Metwally NA, Shehata GA, Badry R, Abd-Elhamed MA. Epidemiology of uncontrolled epilepsy in the Al-Kharga District, New Valley, Egypt. *Seizure*. 2013; 22(8): 611-6. |
| Hunter E, Rogathi J, Chigudu S, Jusabani A, Jackson M, McNally R, Gray W, Whittaker RG, Iqbal A, Birchall D, Aris E, Walker R. Prevalence of active epilepsy in rural Tanzania: a large community-based survey in an adult population. *Seizure*. 2012; 21(9): 691-8. |
| Mbuba CK, Ngugi AK, Fegan G, Ibinda F, Muchohi SN, Nyundo C, Odhiambo R, Edwards T, Odermatt P, Carter JA, Newton CR. Risk factors associated with the epilepsy treatment gap in Kilifi, Kenya: a cross-sectional study. *Lancet Neurol*. 2012; 11(8): 688-96. |
| Malik MA, Akram RM, Tarar MA, Sultan A. Childhood epilepsy. *J Coll Physicians Surg Pak*. 2011;21(2): 74-8. |

| |
|---|
| Zhao Y-H, Zhang Q, Long N, Yang C, Hong J, Mu L, Zhou D. Prevalence of epilepsy and alcohol-related risk in Zayul County, Tibet Autonomous Region in China: an initial survey. *Epilepsy Behav*. 2010;19(4): 635-8. |
| Nicoletti A, Sofia V, Vitale G, Bonelli SI, Bejarano V, Bartalesi F, Tran DS, Preux PM, Zappia M, Bartoloni A. Natural history and mortality of chronic epilepsy in an untreated population of rural Bolivia: a follow-up after 10 years. *Epilepsia*. 2009; 50(10): 2199-206. |
| Kobau R, Zahran H, Grant D, Thurman DJ, Price PH, Zack MM. Prevalence of active epilepsy and health-related quality of life among adults with self-reported epilepsy in California: California Health Interview Survey, 2003. *Epilepsia*. 2007; 48(10): 1904-13. |
| Noronha ALA, Borges MA, Marques LHN, Zanetta DMT, Fernandes PT, de Boer H, Espíndola J, Miranda CT, Prilipko L, Bell GS, Sander JW, Li LM. Prevalence and pattern of epilepsy treatment in different socioeconomic classes in Brazil. *Epilepsia*. 2007; 48(5): 880-5. |
| Oun A, Haldre S, Magi M. Use of antiepileptic drugs in Estonia: an epidemiologic study of adult epilepsy. *Eur J Neurol*. 2006; 13(5): 465-70. |
| Kochen S, Melcon MO. Prognosis of epilepsy in a community-based study: 8 years of follow-up in an Argentine community. *Acta Neurol Scand*. 2005; 112(6): 370-4. |
| Somoza MJ, Forlenza RH, Brussino M, Licciardi L. Epidemiological survey of epilepsy in the primary school population in Buenos Aires. *Neuroepidemiology*. 2005; 25(2): 62-8. |
| Gomes Md M da M, Zeitoune RG, Kropf LAL, van Beeck Ed E da S. A house-to-house survey of epileptic seizures in an urban community of Rio de Janeiro, Brazil. *Arq Neuropsiquiatr*. 2002; 60(3-B): 708-11. |
| Wang W, Wu J, Wang D, Chen G, Wang T, Yuan C, Yang B, Zhao D. [Epidemiological survey on epilepsy among rural populations in five provinces in China]. *Nat Med J Chin*. 2002; 82(7): 449-52. |
| Zhao Y, Zhang Q, Tsering T, Sangwan, Hu X, Liu L, Shang H, Chen Q, Liu Y, Yang X, Wang W, Li S, Wu J, Sander JW, Zhou D. Prevalence of convulsive epilepsy and health-related quality of life of the population with convulsive epilepsy in rural areas of Tibet Autonomous Region in China: an initial survey. *Epilepsy Behav*. 2008; 12(3): 373-81. |
| Placencia M, Shorvon SD, Paredes V, Bimos C, Sander JW, Suarez J, Cascante SM. Epileptic seizures in an Andean region of Ecuador. Incidence and prevalence and regional variation. *Brain*. 1992;115 (Pt 3): 771-82. |
| Del Brutto OH, Santibáñez R, Idrovo L, Rodrìguez S, Díaz-Calderón E, Navas C, Gilman RH, Cuesta F, Mosquera A, Gonzalez AE, Tsang VCW, García HH. Epilepsy and neurocysticercosis in Atahualpa: a door-to-door survey in rural coastal Ecuador. *Epilepsia*. 2005; 46(4): 583-7. |
| Benavente I, Rubio E, Morales C, Tajada N, Tamargo P. Prevalence of epilepsy amongst adolescents in Huesca, Spain: a community-based study. *Eur J Neurol*. 2009; 16(10): 1138-43. |
| Tekle-Haimanot R, Forsgren L, Ekstedt J. Incidence of epilepsy in rural central Ethiopia. *Epilepsia*. 1997; 38(5): 541-6. |
| Almu S, Tadesse Z, Cooper P, Hackett R. The prevalence of epilepsy in the Zay Society, Ethiopia – an area of high prevalence. *Seizure*. 2006; 15(3): 211-3. |
| Löfgren E, Pouta A, von Wendt L, Tapanainen J, Isojärvi JIT, Järvelin M-R. Epilepsy in the northern Finland birth cohort 1966 with special reference to fertility. *Epilepsy Behav*. 2009; 14(1): 102-7. |
| Jallon P. [Evaluation of the prevalence of epilepsy in a military selection centre]. *Rev Neurol* (Paris). 1991; 147(4): 319-22. |

| |
|---|
| Ross EM, Peckham CS, West PB, Butler NR. Epilepsy in childhood: findings from the National Child Development Study. *BMJ*. 1980; 280(6209): 207-10. |
| Coleman R, Loppy L, Walraven G. The treatment gap and primary health care for people with epilepsy in rural Gambia. *Bull World Health Organ*. 2002; 80(5): 378-83. |
| Medina MT, Durón RM, Martínez L, Osorio JR, Estrada AL, Zúniga C, Cartagena D, Collins JS, Holden KR. Prevalence, incidence, and etiology of epilepsies in rural Honduras: the Salama Study. *Epilepsia*. 2005; 46(1): 124-31. |
| Koul R, Razdan S, Motta A. Prevalence and pattern of epilepsy (Lath/Mirgi/Laran) in rural Kashmir, India. *Epilepsia*. 1988; 29(2): 116-22. |
| Mani KS, Rangan G, Srinivas HV, Kalyanasundaram S, Narendran S, Reddy AK. The Yelandur study: a community-based approach to epilepsy in rural South India – epidemiological aspects. *Seizure*. 1998; 7(4): 281-8. |
| Saha SP, Bhattacharya S, Das SK, Maity B, Roy T, Raut DK. Epidemiological study of neurological disorders in a rural population of Eastern India. *J Indian Med Assoc*. 2003; 101(5): 299-304. |
| Hackett RJ, Hackett L, Bhakta P. The prevalence and associated factors of epilepsy in children in Calicut District, Kerala, India. *Acta Paediatr*. 1997; 86(11): 1257-60. |
| Banerjee TK, Ray BK, Das SK, Hazra A, Ghosal MK, Chaudhuri A, Roy T, Raut DK. A longitudinal study of epilepsy in Kolkata, India. *Epilepsia*. 2010; 51(12): 2384-91. |
| Sureka RK, Sureka R. Prevalence of epilepsy in rural Rajasthan--a door-to-door survey. *J Assoc Physicians India*. 2007; 55: 741-2. |
| Edwards T, Scott A, Munyoki G, Odera V, Chengo E, Bauni E, Kwasa T, Sander L, Neville B, Newton C. Active convulsive epilepsy in a rural district of Kenya: a study of prevalence and possible risk factors. *Lancet Neurol*. 2008; 7(1): 50-6. |
| Tran D-S, Odermatt P, Le T-O, Huc P, Druet-Cabanac M, Barennes H, Strobel M, Preux P-M. Prevalence of epilepsy in a rural district of central Lao PDR. *Neuroepidemiology*. 2006; 26(4): 199-206. |
| Traore M, Tahny R, Sacko M. Prévalence de l'épilepsie chez les enfants de 3 à 15 ans dans 2 communes du district de Bamako. *Rev Neurol* (Paris). 2000; 156(Suppl 1): S18. |
| Brodtkorb E, Sjaastad O. Epilepsy prevalence by individual interview in a Norwegian community. *Seizure*. 2008; 17(7): 646-50. |
| Svendsen T, Lossius M, Nakken KO. Age-specific prevalence of epilepsy in Oppland County, Norway. *Acta Neurol Scand*. 2007; 116(5): 307-11. |
| Aziz H, Güvener A, Akhtar SW, Hasan KZ. Comparative epidemiology of epilepsy in Pakistan and Turkey: population-based studies using identical protocols. *Epilepsia*. 1997; 38(6): 716-22. |
| Ndoye NF, Sow AD, Diop AG, Sessouma B, Séne-Diouf F, Boissy L, Wone I, Touré K, Ndiaye M, Ndiaye P, de Boer H, Engel J, Mandlhate C, Meinardi H, Prilipko L, Sander JWAS. Prevalence of epilepsy its treatment gap and knowledge, attitude and practice of its population in sub-urban Senegal an ILAE/IBE/WHO study. *Seizure*. 2005; 14(2): 106-11. |
| Karaagaç N, Yeni SN, Senocak M, Bozluolçay M, Savrun FK, Ozdemir H, Cagatay P. Prevalence of epilepsy in Silivri, a rural area of Turkey. *Epilepsia*. 1999; 40(5): 637-42 |
| Topalkara K, Akyuz A, Sumer H, Bekar D, Topaktas S, Dener S. An epilepsy prevalence study performed using a stratified sampling method among urban residents of Sivas. *Epilepsi*. 1999; 5(1): 24-9. |

Sahin A, Bolayir E, Sumer H, Tas A, Mollaoglu M, Dener S. Epidemiologic evaluation of epileptic and nonepileptic seizures in Sivas region of middle Anatolia. *Neurol Psych Brain Res*. 2004; 11(2): 97-102.

Calisir N, Bora I, Irgil E, Boz M. Prevalence of epilepsy in Bursa city center, an urban area of Turkey. *Epilepsia*. 2006; 47(10): 1691-9.

Su C, Chang S, Chen Z, Lee C, Chen R. Neuroepidemiological survey in Ilan, Taiwan (NESIT): (4) Prevalence of epilepsy. *Acta Neurol Taiwan*. 1998; 7(2): 75-84.

Dent W, Helbok R, Matuja WBP, Scheunemann S, Schmutzhard E. Prevalence of active epilepsy in a rural area in South Tanzania: a door-to-door survey. *Epilepsia*. 2005; 46(12): 1963-9.

Winkler AS, Kerschbaumsteiner K, Stelzhammer B, Meindl M, Kaaya J, Schmutzhard E. Prevalence, incidence, and clinical characteristics of epilepsy--a community-based door-to-door study in northern Tanzania. *Epilepsia*. 2009; 50(10): 2310-3.

Chiang KL, Cheng CY. Prevalence and neuro-psychiatric comorbidities of pediatric epilepsy in Taiwan: a national population-based study. *Epilepsy Res*. 2014; 108(8): 1451-60.

Khedr EM, Shawky OA, Ahmed MA, Elfetoh NA, Al Attar G, Ali AM, Kandil MR, Farweez H. A community based epidemiological study of epilepsy in Assiut Governorate/Egypt. *Epilepsy Res*. 2013; 103(3-Feb): 294-302.

Hu J, Si Y, Zhou D, Mu J, Li J, Liu L, Zhu CR, Deng Y, He J, Zhang NM, Chen XF. Prevalence and treatment gap of active convulsive epilepsy: a large community-based survey in rural West China. *Seizure*. 2014; 23(5): 333-7.

Nwani PO, Nwosu MC, Enwereji KO, Asomugha AL, Arinzechi EO, Ogunniyi AO. Epilepsy treatment gap: prevalence and associated factors in Southeast Nigeria. *Acta Neurol Scand*. 2013; 128(2): 83-90.

El-Tallawy HN, Farghaly WM, Shehata GA, Abdel-Hakeem NM, Rageh TA, Abo-Elftoh NA, Hegazy A, Badry R. Epidemiology of epilepsy in New Valley Governorate, Al Kharga District, Egypt. *Epilepsy Res*. 2013; 104(2-Jan): 167-74.

Pi X, Zhou L, Cui L, Liu A, Zhang J, Ma Y, Liu B, Cai C, Zhu C, Zhou T, Chen J, Zhou Z, Wang C, Li L, Li S, Wu J, Xiao B. Prevalence and clinical characteristics of active epilepsy in southern Han Chinese. *Seizure*. 2014; 23(8): 636-40.

Lomidze G, Kasradze S, Kvernadze D, Okujava N, Toidze O, de Boer HM, Dua T, Sander JW. The prevalence and treatment gap of epilepsy in Tbilisi, Georgia. *Epilepsy Res*. 2012; 98(2-3): 123-9.

Goel D, Dhanai JS, Agarwal A, Mehlotra V, Saxena V. Neurocysticercosis and its impact on crude prevalence rate of epilepsy in an Indian community. *Neurol India*. 2011; 59(1): 37-40.

Velioglu SK, Bakirdemir M, Can G, Topbas M. Prevalence of epilepsy in northeast Turkey. *Epileptic Disord*. 2010; 12(1): 22-37.

El-Tallawy HN, Farghaly WM, Rageh TA, Shehata GA, Metwally NA, Badry R, Sayed MA, Abdelwarith AM, Kandil MR, Hamed MA, Mohamed KO, Tohamy AM. Spectrum of epilepsy - prevalence, impact, and treatment gap: an epidemiological study from Al-Quseir, Egypt. *Neuropsychiatr Dis Treat*. 2016; 12: 1111-8.

Hunter E, Rogathi J, Chigudu S, Jusabani A, Jackson M, Whittaker RG, Gray W, McNally RJ, Aris E, Mushi D, Walker R. The epilepsy treatment gap in rural Tanzania: A community-based study in adults. *Seizure*. 2016; 36: 49-56.

| |
|---|
| Serrano-Castro PJ, Mauri-Llerda JA, Hernandez-Ramos FJ, Sanchez-Alvarez JC, Parejo-Carbonell B, Quiroga-Subirana P, Vazquez-Gutierrez F, Santos-Lasaosa S, Mendez-Lucena C, Redondo-Verge L, Tejero-Juste C, Morandeira-Rivas C, Sancho-Rieger J, Matias-Guiu J. Adult Prevalence of Epilepsy in Spain: EPIBERIA, a Population-Based Study. *Scientific World Journal*. 2015; 2015: 602710. |
| Hashem S, Al-Kattan M, Ibrahim SY, Shalaby NM, Shamloul RM, Farrag M. Epilepsy prevalence in Al-Manial Island, Egypt. A door-to-door survey. *Epilepsy Res*. 2015; 117: 133-7. |
| Sebera F, Munyandamutsa N, Teuwen DE, Ndiaye IP, Diop AG, Tofighy A, Boon P, Dedeken P. Addressing the treatment gap and societal impact of epilepsy in Rwanda--Results of a survey conducted in 2005 and subsequent actions. *Epilepsy Behav*. 2015; 46: 126-32. |
| Banerjee TK, Dutta S, Ray BK, Ghosal M, Hazra A, Chaudhuri A, Das SK. Epidemiology of epilepsy and its burden in Kolkata, India. *Acta Neurol Scand*. 2015; 132(3): 203-11. |
| Wang WZ, Wu JZ, Wang DS, Dai XY, Yang B, Wang TP, Yuan CL, Scott RA, Prilipko LL, de Boer HM, Sander JW. The prevalence and treatment gap in epilepsy in China: an ILAE/IBE/WHO study. *Neurology*. 2003; 60(9): 1544–5. |
| Nizamie SH, Akthar S, Banerjee I, Goyal N. Health care delivery model in epilepsy to reduce treatment gap: World Health Organization study from a rural tribal population of India. *Epilepsy Res*. 2009; 84(2–3): 146–52. |
| Banerjee TK, Hazra A, Biswas A, Ray J, Roy T, Raut DK, Chaudhuri A, Das SK. Neurological disorders in children and adolescents. *Indian J Pediatr*. 2009; 76(2): 139-46. |
| Goel D, Agarwal A, Dhanai JS, Semval VD, Mehrotra V, Saxena V, Maithili B. Comprehensive rural epilepsy surveillance programme in Uttarakhand state of India. *Neurol India*. 2009; 57(3): 355–6. |
| Pal DK, Das T, Sengupta S. Comparison of key informant and survey methods for ascertainment of childhood epilepsy in West Bengal, India. *Int J Epidemiol*. 1998; 27(4): 672–6. |
| Levy LF. Epilepsy in Rhodesia, Zambia, and Malawi. *Afr J Med Sci*. 1970; 1: 291-203. |
| Kun LN, Ling LW, Wah YW, Lian TT. Epidemiologic study of epilepsy in young Singaporean men. *Epilepsia*. 1999; 40(10): 1384-7. |

## Non-fatal estimates

The guidelines for epidemiological studies on epilepsy, its classification and definition from the International League Against Epilepsy (ILAE)[7,8] formed the basis for our reference definition. An epilepsy case was defined as someone with an active, recurring condition of epileptic seizures, at least two, unprovoked by any immediate cause, and who has had at least one epileptic seizure in the past five years regardless of antiepileptic drug treatment.[7] We utilised data from additional sources from 2016 to January 2022. This latest systematic review covered January 10, 2016, to January 28, 2022. This review yielded 24 new sources on two measures (see appendix p. 6 for details). The studies that were included were population-based, representative surveys that reported prevalence, incidence, remission rate, excess mortality rate, relative risk of mortality, standardised mortality ratio, or with-condition mortality rate. Studies that had no clearly defined sample were excluded. Studies that recorded the lifetime recall of epilepsy were crosswalked (the process of adjusting data for known biases) to the reference definition for epilepsy. We used clinical claims data from Poland in 2018 and Taiwan (province of China) from 2016. These data were defined in ICD-10 terms. When a study reported both age- and sex-specific data separately, the male:female proportion was taken from the sex-specific data and was applied to the individual age-specific data to get age- and sex-specific estimates. Using a GBD meta-regression—Bayesian, regularised, trimmed (MR-BRT) method[9] on the

log male:female ratio of prevalence, we split observations where sex was reported for males and females combined into observations for males and females separately. Data which covered an age period of more than 25 years were split into five-year age bands using the age patterns discerned from DisMod-MR 2.1,[10] a Bayesian meta-regression tool, built on a subset of the epilepsy data with age bands less than 25 years. DisMod-MR 2.1 was also used to model prevalence and incidence for overall epilepsy. The log-transformed lag-distributed income per capita (LDI)[11] was used as a covariate for the excess mortality rate to account for the expected lower mortality rate of epilepsy in countries with higher Socio-demographic Index (SDI, a composite measure of lag-distributed income per capita, average years of education for those aged 15 years or older, and fertility rates among females younger than 25 years). Similarly, the log-transformed age-standardised GBD health indicator summary exposure value (SEV, a single, interpretable measure which captures risk-weighted exposure for a population, or risk-weighted prevalence of an exposure), was a covariate for prevalence and summarised the epilepsy exposure risk level for each country.

## Cause of Death Ensemble model

For assessment of mortality due to epilepsy, we used underlying cause of death (CoD), with corrections made to CoD data.[12] Data used to estimate epilepsy mortality included vital registration (VR), verbal autopsy, and China mortality surveillance data The International Classification of Diseases (ICD) was used to re-assign intermediary or unspecified causes. The codes for epilepsy for both ICD-9 (Code 345) and ICD-10 (Codes G40 and G41) were used. A Cause of Death Ensemble model[12] was used to model mortality. This is a method produced specifically for cause of death analysis in the GBD study. A site-year is a unique combination of calendar year, location, and data source. The Cause of Death Ensemble model for epilepsy also utilised predictive covariates for pigs (per capita), proxy for neurocysticercosis infection, SEV scalar: epilepsy, mean systolic blood pressure (mmHg), Healthcare Access and Quality Index, mean body-mass index, mean serum total cholesterol (mmol/L), cumulative cigarettes (10 years), cumulative cigarettes (5 years), education (years per capita), log LDI (per capita), and Socio-demographic Index. More information on calculations can be found in in the GBD 2021 risk factor overview paper.[13]

## GATHER compliance table

GATHER checklist of information that should be included in reports of global health estimates, with description of compliance and location of information for GBD 2021 (Table 1).

Table 1. GATHER checklist

| # | GATHER checklist item | Description of compliance | Reference |
|---|---|---|---|
| **Objectives and funding** | | | |
| 1 | Define the indicators, populations, and time periods for which estimates were made. | Narrative provided in paper and appendix describing indicators, definitions, and populations | Main text (Methods) and appendix |
| 2 | List the funding sources for the work. | Funding sources listed in paper | Summary (Funding) |
| **Data Inputs** | | | |
| *For all data inputs from multiple sources that are synthesised as part of the study:* | | | |

| | | | |
|---|---|---|---|
| 3 | Describe how the data were identified and how the data were accessed. | Narrative description of data seeking methods provided | Main text (Methods) and appendix |
| 4 | Specify the inclusion and exclusion criteria. Identify all ad-hoc exclusions. | Narrative about inclusion and exclusion criteria by data type provided; ad hoc exclusions in cause-specific write-ups | Main text (Methods) and appendix |
| 5 | Provide information on all included data sources and their main characteristics. For each data source used, report reference information or contact name/institution, population represented, data collection method, year(s) of data collection, sex and age range, diagnostic criteria or measurement method, and sample size, as relevant. | An interactive, online data source tool that provides metadata for data sources by component, geography, cause, risk, or impairment has been developed | Online data citation tools: http://ghdx.healthdata.org/gbd-2016 |
| 6 | Identify and describe any categories of input data that have potentially important biases (e.g., based on characteristics listed in item 5). | Summary of known biases by cause included in appendix | Appendix |
| *For data inputs that contribute to the analysis but were not synthesised as part of the study:* | | | |
| 7 | Describe and give sources for any other data inputs. | Included in online data source tool | http://ghdx.healthdata.o<br>rg/gbd-2016 |
| *For all data inputs:* | | | |
| 8 | Provide all data inputs in a file format from which data can be efficiently extracted (e.g., a spreadsheet as opposed to a PDF), including all relevant meta-data listed in item 5. For any data inputs that cannot be shared due to ethical or legal reasons, such as third-party ownership, provide a contact name or the name of the institution that retains the right to the data. | Downloads of input data available through online tools, including data visualisation tools and data query tools; input data not available in tools will be made available upon request | Online data visualisation tools, data query tools, and the Global Health Data Exchange |
| **Data analysis** | | | |
| 9 | Provide a conceptual overview of the data analysis method. A diagram may be helpful. | Flow diagrams of the overall methodological processes, as well as cause-specific modelling processes, have been provided | Main text (Methods) and appendix |
| 10 | Provide a detailed description of all steps of the analysis, including mathematical formulae. This description should cover, as relevant, data cleaning, data pre-processing, data adjustments and weighting of data sources, and mathematical or statistical model(s). | Flow diagrams and corresponding methodological write- ups for each cause, as well as the databases and modelling processes, have been provided | Main text (Methods) and<br>appendix |
| 11 | Describe how candidate models were evaluated and how the final model(s) were selected. | Provided in the methodological write- ups | Appendix |
| 12 | Provide the results of an evaluation of model performance, if done, as well as the results of any relevant sensitivity analysis. | Provided in the methodological write- ups | Appendix |

| | | | |
|---|---|---|---|
| 13 | Describe methods for calculating uncertainty of the estimates. State which sources of uncertainty were, and were not, accounted for in the uncertainty analysis. | Appendix | Appendix |
| 14 | State how analytic or statistical source code used to generate estimates can be accessed. | Appendix | http://ghdx.healthdata.o rg/gbd-2016-code |
| **Results and Discussion** | | | |
| 15 | Provide published estimates in a file format from which data can be efficiently extracted. | GBD 2016 results are available through online data visualisation tools, the Global Health Data Exchange, and the online data query tool | Main text, and online data tools (data visualisation tools, data query tools, and the Global Health Data Exchange) |
| 16 | Report a quantitative measure of the uncertainty of the estimates (e.g. uncertainty intervals). | Uncertainty intervals are provided with all results | Main text, appendix, and online data tools (data visualisation tools, data query tools, and the Global Health Data Exchange) |
| 17 | Interpret results in light of existing evidence. If updating a previous set of estimates, describe the reasons for changes in estimates. | Discussion of methodological changes between GBD rounds provided in the narrative of the manuscript and appendix | Main text (Methods and Discussion) and appendix |
| 18 | Discuss limitations of the estimates. Include a discussion of any modelling assumptions or data limitations that affect interpretation of the estimates. | Discussion of limitations provided in the narrative of the main paper, as well as in the methodological write-ups in the appendix | Main text (Limitations) and appendix |

## Epilepsy mortality

Flowchart

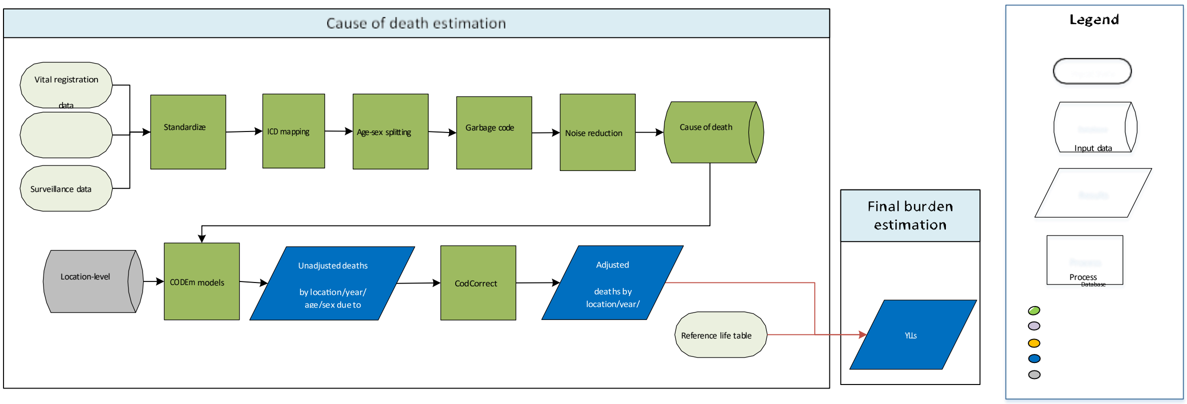


### Input data

Data used to estimate epilepsy mortality included vital registration (VR), verbal autopsy, and China mortality surveillance data from the cause of death (CoD) database. Our outlier criteria were to exclude datapoints that (1) were implausibly high or low relative to global or regional patterns, (2) substantially conflicted with established age or temporal patterns, or (3) significantly conflicted with other data sources from the same locations or locations with similar characteristics (i.e., Socio-demographic Index).

Based on these criteria, we excluded ICD-9 BTL data for Sri Lanka, Fiji, and Kiribati, as the estimates varied from year to year between zero and high values. We also excluded the Survey of Causes of Death Data and Medical Certification of Cause of Death Data for India, as these data types were not consistent with the Sample Registration System Data and would have led to discontinuities in our estimates over time.

### Modelling strategy

The standard CODEm modelling approach was applied to estimate deaths due to epilepsy. Separate models were conducted for male and female mortality, and the age range for both models was 28 days – 95+ years. For GBD 2021, the health systems access covariate was replaced with the Healthcare Access and Quality Index covariate. There were no other substantial changes for GBD 2021. The covariates used are displayed below (Table 2).

Table 2. List of covariates

| Level | Covariate | Direction |
|---|---|---|
| 1 | pig meat consumption (kcal per capita) | + |
| | pigs (per capita) | + |
| | SEV scalar: epilepsy | + |
| | mean systolic blood pressure (mmHg) | + |
| 2 | Healthcare Access and Quality Index | - |
| | mean body-mass index | + |
| | mean serum total cholesterol (mmol/L) | + |
| 3 | cumulative cigarettes (10 years) | + |
| | cumulative cigarettes (5 years) | + |

| | | |
|---|---|---|
| | education (years per capita) | - |
| | log LDI (per capita) | - |
| | Socio-demographic Index | - |
| | mean systolic blood pressure (mmHg) | + |
| 2 | Healthcare Access and Quality Index | - |
| | mean body-mass index | + |
| | mean serum total cholesterol (mmol/L) | + |
| 3 | cumulative cigarettes (10 years) | + |
| | cumulative cigarettes (5 years) | + |
| | education (years per capita) | - |
| | log LDI (per capita) | - |
| | Socio-demographic Index | - |

## Epilepsy impairment

Flowchart

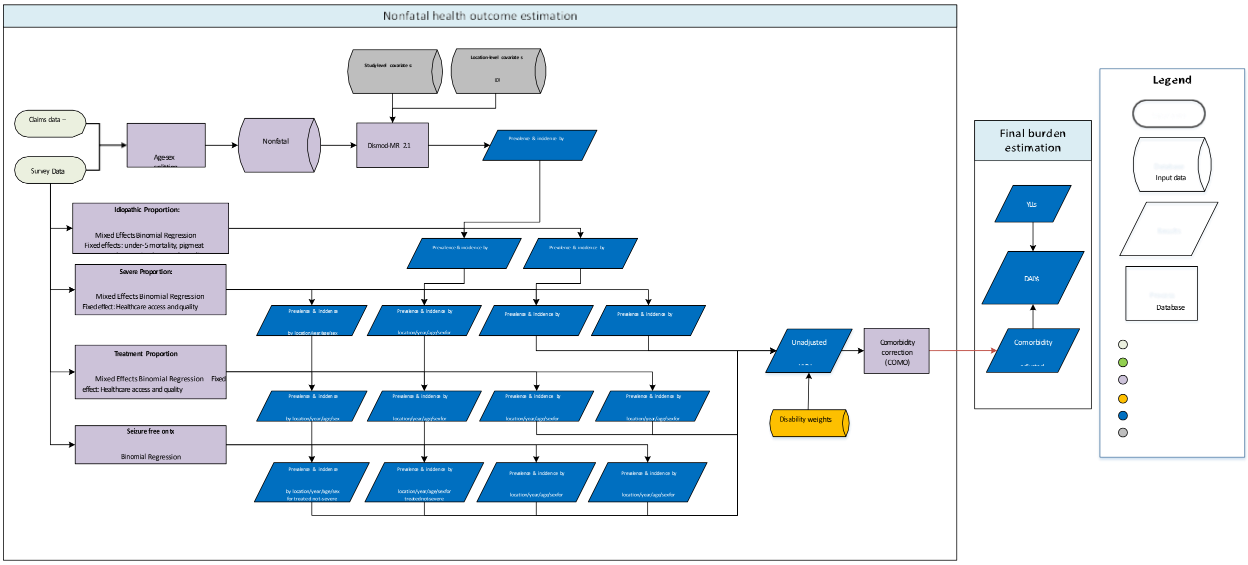


### Case definition

Since GBD 2013, we have used the following definitions from the “Guidelines for Epidemiologic Studies on Epilepsy”: 1) Epilepsy: a condition characterised by recurrent (two or more) epileptic seizures, unprovoked by any immediate identified cause, and 2) “Active” epilepsy: a prevalent case of active epilepsy is defined as a person with epilepsy who has had at least one epileptic seizure in the previous five years, regardless of antiepileptic drug (AED) treatment. We also use the following ICD-10 codes for epilepsy: G40 (Neuro, epilepsy, total) and G41 (Neuro, epilepsy, status epilepticus). We defined severe epilepsy as having seizures one or more times per month.

### Input data

The inputs for the regressions used to split the epilepsy impairment envelope were also updated for GBD 2021. These regressions are used to determine the proportion of epilepsy that is primary or idiopathic.

### *Disability weights*

To adjust for differences in methodological quality, all prevalence studies included in GBD were scored according to a modified version (dichotomised variables) of published methodological quality criteria for epilepsy epidemiological studies, taking into account the representativeness of the population of interest (representative of country or community versus selected population), quality of sampling (random sample of the population of interest versus not random sample), recall period (one-year prevalence versus other recall period), participation rate (≥70% versus <70%), survey method (face to face with epilepsy expert or trained interviewer versus other), validation of diagnostic instrument (sensitivity or specificity ≥70% versus <70% or no validation). In DisMod-MR, these methodological variables were evaluated for a systematic difference and corrected accordingly.

### *Modelling strategy*

We modelled the prevalence of epilepsy in two steps: first, we created an epilepsy impairment envelope. Second, we split the envelope into primary (or idiopathic) and secondary epilepsies. Each of these were subdivided into "severe" (on average one or more fits per month) and "non-severe." Non-severe cases were subdivided into "treated" and "untreated." Finally, "treated" cases were divided into "treated cases with fits" (between one and 11 fits on average in the preceding year) and "treated cases without fits" (no fits reported in the preceding year).

In the first step, we used the DisMod-MR tool for the epilepsy impairment envelope to model a consistent fit between incidence, prevalence, remission, and standardised mortality ratio data while using meta-regression to correct datapoints with non-reference study quality characteristics. We found no systematic bias for the covariate "non-standard case definition", indicating studies that did not define "active epilepsy"; additionally, the covariate was not significant as a "z-cov", which acts as a multiplier applied to the standard error and thus results in these datapoints being given less weight in the analysis than the "reference" datapoints. Therefore, we excluded this covariate from the model. We also included data on lifetime prevalence and therefore added a covariate on lifetime prevalence datapoints. We also included country-level covariates on prevalence for the SEV epilepsy scalar, which summarises the epilepsy risk exposure level for each country, and pig meat consumption per capita, which is used as a proxy for the level of neurocysticercosis, a common cause of secondary epilepsy. We included cause-specific mortality rate (CSMR) results from the epilepsy mortality model as input data to the DisMod model. Where age-specific prevalence data were available, we calculated excess mortality rate (EMR) from prevalence and CSMR. We included the log of the lag-distributed income (LDI) as a covariate on EMR to account for lower mortality in developed countries. We included Bayesian priors on remission to account for the scarcity of remission data. We set bounds on remission from 0 to 0.25 from age 0 to 60 and 0 to 0.05 from age 61 to 100.

In the second step, we used a mixed-effects generalised linear model (binomial family) to predict the proportion of idiopathic epilepsy, the proportion of severe epilepsy, the proportion of treated epilepsy, and the proportion of epilepsy that is treated without fits.

Because not all of the data on the proportion of idiopathic epilepsy use optimal case-finding methods (using CT scans or MRIs in addition to EEGs in order to diagnose secondary epilepsy), for GBD 2021 we decided to add a covariate to crosswalk studies with non-optimal case-finding methods to those with adequate methods. The regression for the proportion of epilepsy that is idiopathic therefore has fixed effects on this study quality covariate as well as the under-5 mortality rate, the log of pig meat consumption (per capita), and the proportion of a country with access to proper sanitation, as well as a random effect on super-region.

We used similar models to predict the proportion of severe epilepsy and treatment gap based on the reported proportions extracted from the systematic review. To predict the proportion of severe epilepsy and the treatment gap, we used mixed-effects models with a fixed effect on the log of HAQ Index and a random effect on super-region.

For GBD 2015, a meta-analysis was used to generate two different pooled estimates for proportion of treated epilepsy that is seizure-free in developing and developed countries, as there were not enough data to run a regression. However, for GBD 2021, the expanded dataset allowed for the implementation of a generalised linear model (binomial family) to generate predictions for the proportion of treated epilepsy that is seizure-free. We used a fixed effect on the log of HAQ Index.

Studies which did not use advanced diagnostic methods were readjusted to those that used in a similar region all possible diagnostic methods for secondary epilepsy using a binary covariate for study quality based on whether the study explicitely described use of neuroimaging diagnostics across all study participants. We tagged estimates in both HICs and LMICs as having done 'proper'/full work-up or not and adjusted them accordingly. In other words, we used a binary covariate for study quality based on whether the study explicitly described use of imaging diagnostics across all study participants. "High quality" tags were not unique to "high-income" countries. We've copied below an example of breakdown of how studies were tagged by country. Therefore, the adjustments in LMICs and HICs were made based upon similar to economic development areas that did use advanced methods as shown in the screenshot.

| Country | Imaging used for all participants | Imaging not used for all participants |
|---|---|---|
| Brazil | 2 | 0 |
| Canada | 0 | 1 |
| China | 4 | 3 |
| Costa Rica | 1 | 0 |
| Croatia | 1 | 0 |
| Cuba | 1 | 1 |
| DRC | 1 | 0 |
| Egypt | 4 | 1 |
| Estonia | 2 | 1 |
| Ethiopia | 0 | 1 |
| Finland | 0 | 2 |
| France | 0 | 1 |
| Georgia | 1 | 0 |
| Germany | 1 | 0 |
| Honduras | 1 | 0 |
| Iceland | 2 | 0 |
| India | 2 | 4 |
| Italy | 1 | 1 |
| Japan | 1 | 1 |
| Kenya | 0 | 2 |
| Lao | 0 | 1 |
| Libya | 0 | 1 |
| Madagascar | 0 | 2 |
| Malaysia | 0 | 1 |
| Mali | 1 | 0 |
| Mexico | 1 | 2 |
| Mongolia | 1 | 0 |
| Netherlands | 0 | 1 |
| Nigeria | 1 | 3 |
| Norway | 1 | 0 |
| Pakistan | 1 | 0 |
| Peru | 0 | 1 |
| Saudi Arabia | 1 | 0 |
| Senegal | 1 | 0 |
| Singapore | 1 | 2 |
| South Korea | 1 | 0 |
| Spain | 3 | 3 |
| Sweden | 1 | 2 |
| Tanzania | 0 | 3 |
| Turkey | 2 | 0 |
| UK | 2 | 0 |
| USA | 3 | 1 |
| Vietnam | 0 | 1 |
| Zambia | 0 | 1 |
| Zimbabwe | 0 | 1 |

We tested a fixed effect on Socio-demographic Index (SDI) and random effects on region and country in different models, but they did not improve the model. We generated 500 draws of country-specific estimates for each year between 1980 and 2021 for each of the models.

## Definition of GBD super-regions and regions

Table 7. GBD super-regions and regions

| Super-region | Region_name |
|---|---|
| East Asia, southeast Asia and Oceania | East Asia |
| | Southeast Asia |
| | Oceania |
| Central Asia, central Europe, eastern Europe | Central Asia |
| | Central Europe |
| | Eastern Europe |
| High income | High-income Asia Pacific |
| | Australasia |
| | Western Europe |
| | High-income North America<br>Southern Latin America |
| Latin America & Caribbean | Caribbean |
| | Andean Latin America |
| Central Latin America | |
| | Tropical Latin America |
| North Africa and Middle East | North Africa and Middle East |
| South Asia | South Asia |
| Sub-Saharan Africa | Central sub-Saharan Africa |
| | Eastern sub-Saharan Africa |
| | Southern sub-Saharan Africa |
| | Western sub-Saharan Africa |

## Tables of the epilepsy burden estimates

### Table 1. Absolute number, with 95% uncertainty intervals (UI), of deaths, DALYs, and incidence associated with idiopathic epilepsy in 2021 and percentage change in the age-standardised metrics for 1990–2021 by seven GBD super-regions, 21 GBD regions, and 204 countries/territories

| Region, country | Incidence | | Deaths | | DALYs | |
|---|---|---|---|---|---|---|
| | Counts | % change in age-standardised rates, 1990–2021 | Counts | % change in age-standardised rates, 1990–2021 | Counts | % change in age-standardised rates, 1990–2021 |
| **Global** | 3,272,734 (2,403,802 to 4,125,119) | 12.3% (-4.8 to 32.6) | 139,851 (116,953 to 153,370) | -15.8% (-22.8 to -8.8) | 13,877,827 (10,732,569 to 17,619,993) | -14.5% (-24.2 to -4.2) |
| **High income region** | 521,937 (347,791 to 693,196) | 9.1% (-13.2 to 28.7) | 18,152 (16,351 to 19,399) | 18.1% (9.3 to 23.8) | 1,402,064 (941,460 to 2,073,856) | -3.2% (-18.4 to 13.3) |
| **High income Asia Pacific** | 71,299 (44,656 to 98,720) | 7.7% (-26.0 to 49.7) | 2,303 (2,011 to 2,517) | 13.4% (-6.2 to 25.1) | 181,526 (116,478 to 288,640) | -12.0% (-38.5 to 23.8) |
| **High income North America** | 143,834 (90,670 to 199,908) | 15.5% (-10.7 to 38.8) | 3,822 (3,582 to 3,979) | 21.4% (17.3 to 24.8) | 423,104 (271,625 to 643,343) | 6.7% (-13.8 to 31.9) |
| **Southern Latin America** | 32,871 (15,909 to 49,491) | 10.0% (-45.5 to 124.7) | 761 (714 to 811) | -12.9% (-19.2 to -5.6) | 93,165 (55,654 to 147,779) | -14.9% (-49.0 to 46.0) |
| **Western Europe** | 260,298 (164,172 to 344,965) | 8.5% (-21.2 to 44.6) | 10,905 (9,548 to 11,837) | 25.9% (13.1 to 34.6) | 668,962 (460,088 to 990,189) | -3.3% (-25.6 to 21.9) |
| **Australasia** | 13,634 (5,442 to 21,149) | -1.7% (-60.2 to 145.7) | 361 (334 to 386) | -26.5% (-31.7 to -20.9) | 35,307 (19,319 to 62,512) | -22.4% (-58.9 to 48.0) |
| **Latin America and Caribbean** | 366,402 (264,770 to 476,603) | -4.1% (-23.8 to 23.0) | 10,545 (9,612 to 11,506) | -14.5% (-21.8 to -6.8) | 1,318,898 (987,773 to 1,773,671) | -23.6% (-37.8 to -8.2) |
| **Central Latin America** | 173,731 (121,135 to 229,075) | -3.7% (-28.7 to 31.0) | 4,826 (4,221 to 5,499) | -22.3% (-32.1 to -11.7) | 636,587 (455,770 to 857,865) | -25.4% (-41.4 to -7.0) |
| **Tropical Latin America** | 121,093 (81,539 to 161,143) | -9.6% (-35.0 to 34.5) | 3,795 (3,622 to 3,937) | 18.3% (11.8 to 24.2) | 422,868 (301,544 to 584,924) | -20.4% (-39.7 to 7.1) |
| **Andean Latin America** | 47,503 (25,464 to 68,780) | 2.0% (-49.9 to 120.6) | 912 (755 to 1,093) | -45.1% (-55.1 to -34.0) | 154,769 (96,445 to 230,916) | -34.4% (-62.9 to 15.4) |
| **Caribbean** | 24,075 (15,128 to 33,912) | -0.1% (-35.6 to 54.2) | 1,012 (840 to 1,221) | -19.4% (-32.0 to -6.0) | 104,675 (75,798 to 143,105) | -14.6% (-35.9 to 9.9) |
| **Sub-Saharan Africa** | 713,285 (484,928 to 952,443) | 7.5% (-14.6 to 39.6) | 38,666 (31,461 to 45,776) | -11.5% (-23.9 to 2.4) | 3,672,786 (2,873,814 to 4,586,892) | -8.5% (-20.8 to 5.2) |
| **Southern sub-Saharan Africa** | 46,002 (29,386 to 63,986) | 1.5% (-30.7 to 51.1) | 1,920 (1,668 to 2,371) | -5.3% (-22.1 to 16.1) | 206,134 (155,806 to 263,097) | -7.8% (-25.9 to 18.3) |
| **Western sub-Saharan Africa** | 323,994 (220,101 to 431,752) | 10.9% (-13.9 to 51.5) | 11,950 (7,788 to 14,810) | 3.3% (-31.7 to 43.2) | 1,327,545 (945,495 to 1,756,096) | 1.5% (-22.3 to 28.1) |
| **Central sub-Saharan Africa** | 85,155 (34,158 to 138,874) | 1.3% (-58.5 to 185.8) | 4,153 (3,086 to 5,731) | -13.4% (-32.9 to 10.5) | 441,289 (287,627 to 628,281) | -13.2% (-42.3 to 31.2) |

| | | | | | | |
|---|---|---|---|---|---|---|
| **Eastern sub-Saharan Africa** | 258,133<br>(167,483 to 358,826) | 6.9%<br>(-21.7 to 63.8) | 20,643<br>(17,587 to 24,914) | -20.4%<br>(-31.1 to -7.5) | 1,697,817<br>(1,361,673 to 2,111,472) | -14.3%<br>(-27.2 to 1.9) |
| **North Africa and Middle East** | 298,247<br>(202,461 to 422,106) | 10.2%<br>(-23.8 to 56.7) | 7,374<br>(5,915 to 8,547) | -41.2%<br>(-48.6 to -33.0) | 982,282<br>(700,420 to 1,361,899) | -31.9%<br>(-47.1 to -11.5) |
| **Southeast Asia, east Asia, and Oceania** | 625,602<br>(437,540 to 811,288) | 23.5%<br>(-6.7 to 61.0) | 16,320<br>(13,632 to 19,470) | -53.2%<br>(-60.2 to -44.9) | 2,217,008<br>(1,574,008 to 2,995,777) | -34.3%<br>(-48.5 to -17.2) |
| **Southeast Asia** | 248,332<br>(170,940 to 336,053) | 16.2%<br>(-14.7 to 58.2) | 3,647<br>(2,597 to 4,322) | -26.2%<br>(-35.5 to -15.4) | 755,399<br>(512,525 to 1,060,685) | -5.1%<br>(-28.6 to 24.2) |
| **Oceania** | 4,420<br>(1,714 to 7,277) | -1.4%<br>(-57.6 to 132.4) | 229<br>(165 to 329) | 10.4%<br>(-13.0 to 41.9) | 26,577<br>(16,444 to 39,292) | 3.0%<br>(-35.8 to 63.9) |
| **East Asia** | 372,849<br>(259,684 to 491,425) | 24.9%<br>(-9.6 to 72.5) | 12,444<br>(10,330 to 15,388) | -55.6%<br>(-62.9 to -46.0) | 1,435,032<br>(1,023,185 to 1,955,288) | -42.6%<br>(-56.1 to -25.3) |
| **Central Europe, eastern Europe, and central Asia** | 145,212<br>(100,228 to 193,226) | -0.7%<br>(-19.7 to 21.1) | 6,935<br>(6,084 to 7,586) | 3.5%<br>(-9.1 to 14.3) | 672,317<br>(510,497 to 873,611) | -9.0%<br>(-23.3 to 5.9) |
| **Central Asia** | 45,659<br>(26,279 to 63,772) | 6.9%<br>(-33.9 to 69.9) | 2,503<br>(2,203 to 2,820) | 11.6%<br>(-2.3 to 26.9) | 270,937<br>(207,394 to 353,474) | 1.7%<br>(-22.9 to 32.9) |
| **Eastern Europe** | 59,543<br>(39,815 to 81,362) | -10.9%<br>(-32.0 to 15.4) | 1,570<br>(1,385 to 1,743) | -40.1%<br>(-46.7 to -33.7) | 185,111<br>(125,736 to 264,284) | -35.4%<br>(-49.8 to -18.9) |
| **Central Europe** | 40,010<br>(26,684 to 53,769) | 3.4%<br>(-23.7 to 37.3) | 2,862<br>(2,346 to 3,210) | 15.7%<br>(-7.1 to 31.6) | 216,270<br>(159,252 to 295,130) | -10.6%<br>(-30.4 to 12.3) |
| **South Asia** | 602,050<br>(428,444 to 784,538) | 7.5%<br>(-23.3 to 57.8) | 41,859<br>(31,308 to 47,631) | -27.7%<br>(-38.9 to -12.2) | 3,612,472<br>(2,845,810 to 4,472,974) | -25.2%<br>(-38.5 to -5.4) |
| | | | | | | |
| **Countries** | | | | | | |
| Afghanistan | 16,320<br>(3,945 to 31,363) | -8.5%<br>(-76.2 to 458.5) | 877<br>(641 to 1,128) | -32.8%<br>(-47.8 to -13.9) | 98,313<br>(61,376 to 152,314) | -33.4%<br>(-59.7 to 14.1) |
| Albania | 991<br>(255 to 1,595) | 10.7%<br>(-72.8 to 286.6) | 60<br>(43 to 94) | -29.8%<br>(-48.2 to -5.5) | 5,560<br>(3,171 to 9,431) | -26.3%<br>(-60.2 to 48.1) |
| Algeria | 19,584<br>(5,214 to 33,093) | -6.0%<br>(-78.7 to 255.2) | 466<br>(377 to 583) | -41.6%<br>(-53.7 to -28.0) | 61,469<br>(32,146 to 101,649) | -38.8%<br>(-71.7 to 26.0) |
| American Samoa | 20<br>(5 to 32) | 4.5%<br>(-72.2 to 287.8) | 1<br>(0 to 1) | 49.7%<br>(-25.6 to 155.4) | 95<br>(50 to 153) | 14.2%<br>(-50.1 to 173.1) |
| Andorra | 43<br>(12 to 65) | -3.5%<br>(-64.3 to 153.4) | 1<br>(1 to 1) | -36.5%<br>(-58.3 to -5.0) | 92<br>(42 to 180) | -24.5%<br>(-66.0 to 75.6) |
| Angola | 25,615<br>(6,738 to 45,346) | 11.5%<br>(-76.1 to 479.3) | 952<br>(670 to 1,293) | -24.0%<br>(-45.7 to 6.0) | 121,740<br>(64,097 to 195,038) | -13.6%<br>(-56.2 to 63.7) |
| Antigua and Barbuda | 67<br>(18 to 106) | 1.4%<br>(-69.0 to 324.2) | 3<br>(3 to 3) | -34.3%<br>(-42.2 to -26.5) | 300<br>(173 to 481) | -26.8%<br>(-62.9 to 43.8) |
| Argentina | 19,540<br>(4,859 to 32,977) | 13.0%<br>(-71.6 to 311.0) | 372<br>(345 to 401) | -12.7%<br>(-19.9 to -4.8) | 51,394<br>(23,949 to 93,016) | -9.7%<br>(-58.8 to 96.3) |
| Armenia | 995<br>(331 to 1,644) | 1.5%<br>(-70.7 to 290.8) | 28<br>(22 to 34) | -16.0%<br>(-35.4 to 4.8) | 3,558<br>(1,720 to 6,136) | -20.2%<br>(-66.1 to 87.5) |
| Australia | 11,257<br>(3,191 to 18,357) | 0.2%<br>(-70.7 to 239.4) | 298<br>(275 to 320) | -24.6%<br>(-30.3 to -18.2) | 29,095<br>(14,924 to 56,000) | -20.2%<br>(-63.6 to 70.0) |

| | | | | | | |
|---|---|---|---|---|---|---|
| Austria | 4,492 (1,349 to 7,044) | 3.5% (-60.5 to 309.1) | 158 (142 to 172) | 27.1% (16.4 to 38.4) | 10,936 (5,535 to 19,587) | -4.2% (-55.5 to 116.8) |
| Azerbaijan | 4,594 (1,369 to 7,563) | 10.1% (-71.0 to 331.5) | 261 (187 to 376) | -11.2% (-34.4 to 20.2) | 27,588 (15,667 to 43,858) | -13.0% (-54.0 to 62.7) |
| Bahamas | 242 (77 to 393) | -3.6% (-71.0 to 232.5) | 6 (5 to 8) | -30.3% (-47.2 to -8.8) | 876 (415 to 1,458) | -24.8% (-69.4 to 68.1) |
| Bahrain | 810 (241 to 1,347) | 2.7% (-72.4 to 251.6) | 20 (16 to 27) | -40.7% (-51.0 to -27.0) | 2,587 (1,380 to 4,429) | -34.4% (-68.9 to 32.2) |
| Bangladesh | 41,793 (11,936 to 73,953) | 2.5% (-72.2 to 463.5) | 1,544 (1,137 to 2,102) | -43.5% (-57.1 to -23.3) | 176,492 (93,809 to 303,266) | -36.1% (-69.1 to 30.3) |
| Barbados | 167 (52 to 265) | -6.2% (-75.7 to 240.4) | 6 (5 to 8) | -36.3% (-50.5 to -18.8) | 624 (334 to 1,062) | -31.0% (-66.3 to 47.4) |
| Belarus | 2,510 (679 to 4,138) | -16.2% (-76.2 to 193.3) | 103 (84 to 124) | -47.3% (-58.2 to -33.7) | 9,621 (5,216 to 16,858) | -45.2% (-72.8 to 10.9) |
| Belgium | 7,213 (2,104 to 11,074) | 11.6% (-70.3 to 287.3) | 365 (321 to 402) | 54.0% (38.3 to 68.4) | 21,537 (12,266 to 37,721) | 11.4% (-49.6 to 132.7) |
| Belize | 248 (70 to 438) | 10.4% (-72.9 to 393.2) | 7 (6 to 8) | -19.9% (-32.1 to -5.1) | 916 (468 to 1,526) | -13.4% (-56.1 to 66.1) |
| Benin | 8,420 (1,944 to 15,562) | 6.1% (-77.6 to 563.1) | 354 (226 to 480) | 3.6% (-34.8 to 51.8) | 38,576 (20,894 to 63,322) | -1.4% (-52.1 to 91.7) |
| Bermuda | 34 (10 to 55) | -9.9% (-71.9 to 238.6) | 1 (1 to 1) | -49.0% (-59.1 to -35.9) | 96 (42 to 187) | -39.3% (-73.2 to 57.5) |
| Bhutan | 231 (64 to 419) | 9.8% (-73.7 to 497.7) | 19 (13 to 27) | -20.8% (-44.9 to 24.2) | 1,660 (953 to 2,604) | -13.7% (-56.1 to 65.6) |
| Bolivia | 5,800 (1,483 to 9,715) | -9.7% (-78.3 to 276.9) | 227 (160 to 312) | -42.7% (-58.7 to -19.1) | 28,633 (15,075 to 46,287) | -39.3% (-69.8 to 23.4) |
| Bosnia and Herzegovina | 1,111 (300 to 1,799) | 13.3% (-70.7 to 325.0) | 78 (49 to 104) | -12.5% (-45.0 to 22.2) | 6,085 (3,339 to 9,948) | -12.0% (-58.0 to 74.7) |
| Botswana | 1,526 (433 to 2,598) | 31.3% (-61.5 to 400.4) | 65 (47 to 84) | -21.1% (-49.0 to 19.0) | 7,654 (4,330 to 11,471) | -1.0% (-47.1 to 85.9) |
| Brazil | 117,285 (78,931 to 156,517) | -10.0% (-34.8 to 34.6) | 3,693 (3,526 to 3,826) | 18.8% (13.1 to 24.7) | 409,015 (290,945 to 565,775) | -20.8% (-39.8 to 8.3) |
| Brunei | 243 (75 to 386) | -11.5% (-67.5 to 225.4) | 5 (4 to 6) | -29.0% (-45.1 to -8.9) | 780 (387 to 1,314) | -30.7% (-69.1 to 62.0) |
| Bulgaria | 2,346 (643 to 3,762) | 4.2% (-70.8 to 247.2) | 180 (135 to 228) | 34.0% (0.5 to 77.9) | 14,512 (8,075 to 23,745) | 4.6% (-48.9 to 128.5) |
| Burkina Faso | 13,358 (2,354 to 26,262) | 8.3% (-78.6 to 450.2) | 633 (393 to 889) | 4.5% (-32.5 to 58.5) | 63,812 (32,779 to 101,254) | 2.1% (-48.7 to 91.4) |
| Burundi | 7,238 (1,429 to 14,567) | -13.3% (-81.9 to 477.5) | 642 (478 to 843) | -3.6% (-27.6 to 29.8) | 50,753 (32,859 to 73,842) | -17.0% (-47.9 to 29.5) |
| Côte d’Ivoire | 18,421 (4,398 to 33,460) | 12.4% (-74.1 to 444.9) | 803 (485 to 1,111) | 9.8% (-32.0 to 59.9) | 85,743 (46,260 to 138,935) | 6.3% (-46.2 to 99.9) |
| Cabo Verde | 369 (100 to 637) | 32.0% (-70.5 to 559.4) | 18 (13 to 23) | -28.6% (-47.4 to -4.0) | 1,619 (871 to 2,641) | -14.6% (-56.0 to 57.0) |

| | | | | | | |
|---|---|---|---|---|---|---|
| Cambodia | 5,545<br>(1,707 to 10,145) | 10.0%<br>(-69.3 to 487.7) | 99<br>(61 to 190) | -26.7%<br>(-47.3 to -3.3) | 19,343<br>(8,402 to 33,204) | -13.0%<br>(-66.7 to 116.2) |
| Cameroon | 20,248<br>(5,267 to 36,088) | 8.2%<br>(-73.2 to 402.0) | 855<br>(510 to 1,198) | 0.4%<br>(-38.4 to 53.8) | 90,245<br>(49,112 to 138,832) | -1.3%<br>(-52.0 to 88.5) |
| Canada | 12,188<br>(3,302 to 19,377) | 5.8%<br>(-65.2 to 228.0) | 395<br>(363 to 423) | -22.9%<br>(-28.1 to -17.4) | 36,611<br>(18,411 to 67,325) | -12.7%<br>(-58.8 to 80.8) |
| Central African Republic | 2,958<br>(609 to 5,533) | -4.7%<br>(-78.8 to 441.6) | 249<br>(175 to 354) | 3.6%<br>(-23.3 to 39.5) | 22,428<br>(14,325 to 33,082) | -0.8%<br>(-40.4 to 69.5) |
| Chad | 10,491<br>(1,871 to 20,868) | 18.9%<br>(-72.0 to 498.3) | 523<br>(342 to 728) | 35.8%<br>(-11.8 to 110.9) | 52,600<br>(28,779 to 83,422) | 27.0%<br>(-31.7 to 143.8) |
| Chile | 11,551<br>(3,444 to 18,452) | 5.1%<br>(-72.0 to 275.2) | 316<br>(294 to 339) | -21.3%<br>(-27.5 to -14.1) | 35,349<br>(17,967 to 63,167) | -24.2%<br>(-67.8 to 77.8) |
| China | 359,966<br>(248,027 to 475,661) | 26.1%<br>(-10.3 to 77.4) | 11,886<br>(9,815 to 14,767) | -56.6%<br>(-64.0 to -47.0) | 1,374,719<br>(969,523 to 1,888,680) | -43.2%<br>(-56.9 to -25.5) |
| Colombia | 30,190<br>(7,624 to 51,156) | -2.2%<br>(-75.2 to 327.2) | 739<br>(612 to 876) | -24.2%<br>(-37.5 to -10.6) | 102,285<br>(49,616 to 183,088) | -28.8%<br>(-71.7 to 67.5) |
| Comoros | 439<br>(132 to 782) | 4.5%<br>(-72.4 to 514.5) | 42<br>(31 to 59) | -13.8%<br>(-39.6 to 29.2) | 3,029<br>(1,963 to 4,322) | -14.3%<br>(-49.2 to 48.6) |
| Congo | 3,801<br>(1,063 to 6,520) | 4.7%<br>(-73.0 to 454.6) | 163<br>(115 to 221) | -18.5%<br>(-39.4 to 11.9) | 18,730<br>(10,262 to 28,721) | -15.7%<br>(-57.8 to 61.5) |
| Cook Islands | 7<br>(2 to 11) | 3.6%<br>(-71.3 to 300.2) | 0<br>(0 to 0) | -33.4%<br>(-52.4 to -2.7) | 24<br>(11 to 44) | -19.6%<br>(-69.3 to 94.0) |
| Costa Rica | 2,860<br>(838 to 4,631) | 1.5%<br>(-71.8 to 273.5) | 86<br>(76 to 97) | -0.5%<br>(-13.9 to 13.2) | 9,906<br>(5,298 to 16,667) | -9.4%<br>(-59.9 to 103.5) |
| Croatia | 1,579<br>(444 to 2,489) | -8.1%<br>(-76.6 to 280.1) | 98<br>(70 to 122) | 9.6%<br>(-22.3 to 38.9) | 7,892<br>(3,927 to 14,288) | -19.1%<br>(-62.1 to 86.6) |
| Cuba | 4,105<br>(1,142 to 6,896) | -5.5%<br>(-75.2 to 276.7) | 120<br>(103 to 138) | -29.2%<br>(-39.5 to -18.2) | 11,668<br>(5,783 to 20,845) | -27.4%<br>(-68.6 to 63.4) |
| Cyprus | 616<br>(170 to 987) | -1.4%<br>(-73.1 to 286.5) | 12<br>(10 to 14) | -51.9%<br>(-60.8 to -41.3) | 1,226<br>(587 to 2,275) | -34.3%<br>(-75.3 to 52.6) |
| Czechia | 4,163<br>(1,255 to 6,575) | 2.5%<br>(-70.6 to 234.4) | 257<br>(195 to 317) | 15.1%<br>(-14.7 to 46.0) | 20,234<br>(10,842 to 35,457) | -10.6%<br>(-56.4 to 101.5) |
| North Korea | 5,162<br>(1,382 to 9,075) | -13.0%<br>(-77.3 to 283.8) | 259<br>(177 to 417) | -24.7%<br>(-46.4 to 8.1) | 28,174<br>(15,412 to 46,236) | -34.1%<br>(-69.2 to 38.2) |
| Democratic Republic of the Congo | 49,843<br>(10,597 to 92,421) | -5.2%<br>(-78.8 to 449.8) | 2,705<br>(1,904 to 3,949) | -9.8%<br>(-33.0 to 19.4) | 266,322<br>(157,817 to 418,849) | -14.7%<br>(-51.1 to 46.9) |
| Denmark | 2,267<br>(606 to 3,531) | -3.0%<br>(-66.7 to 200.5) | 135<br>(119 to 151) | 37.7%<br>(16.4 to 60.6) | 7,279<br>(4,319 to 12,653) | -5.6%<br>(-52.1 to 86.7) |
| Djibouti | 800<br>(212 to 1,429) | 9.8%<br>(-72.2 to 463.7) | 59<br>(41 to 83) | -4.7%<br>(-33.1 to 34.4) | 4,900<br>(3,085 to 7,082) | -3.7%<br>(-43.5 to 64.0) |
| Dominica | 49<br>(17 to 78) | 8.0%<br>(-68.9 to 372.8) | 3<br>(2 to 3) | -14.2%<br>(-34.1 to 9.7) | 263<br>(153 to 408) | -4.1%<br>(-50.5 to 94.6) |
| Dominican Republic | 6,379<br>(1,815 to 10,939) | 22.4%<br>(-65.0 to 364.0) | 155<br>(124 to 204) | -27.6%<br>(-42.9 to -8.0) | 22,957<br>(11,030 to 39,726) | -7.7%<br>(-57.6 to 93.9) |

| | | | | | | |
|---|---|---|---|---|---|---|
| Ecuador | 17,237 (5,412 to 29,200) | 7.4% (-67.4 to 375.3) | 391 (260 to 520) | -32.6% (-54.3 to -10.6) | 57,008 (28,159 to 95,303) | -25.5% (-69.7 to 65.8) |
| Egypt | 52,019 (13,581 to 88,282) | 13.9% (-68.9 to 450.6) | 707 (495 to 884) | -16.1% (-45.8 to 12.0) | 141,500 (63,436 to 258,099) | -17.9% (-65.5 to 109.1) |
| El Salvador | 3,575 (850 to 6,058) | 11.2% (-72.3 to 345.4) | 66 (49 to 83) | -31.6% (-47.0 to -12.0) | 10,804 (4,827 to 19,130) | -22.7% (-67.5 to 71.4) |
| Equatorial Guinea | 1,372 (342 to 2,319) | 58.1% (-63.0 to 1,019.9) | 35 (22 to 54) | -38.5% (-60.8 to -3.6) | 5,431 (2,708 to 8,957) | -7.0% (-59.2 to 96.9) |
| Eritrea | 4,035 (989 to 7,500) | 9.2% (-74.1 to 601.4) | 373 (266 to 509) | 6.6% (-21.8 to 38.6) | 30,121 (19,629 to 43,904) | -2.5% (-37.5 to 52.6) |
| Estonia | 526 (164 to 808) | 15.9% (-66.8 to 323.9) | 30 (24 to 35) | -47.9% (-58.5 to -34.9) | 2,532 (1,368 to 4,280) | -36.0% (-64.8 to 14.5) |
| Eswatini | 689 (161 to 1,221) | 21.5% (-74.5 to 581.7) | 34 (22 to 46) | -6.2% (-41.1 to 44.5) | 3,765 (2,243 to 5,795) | 6.5% (-43.9 to 100.2) |
| Ethiopia | 50,042 (26,329 to 75,113) | 10.0% (-44.1 to 187.2) | 5,212 (4,266 to 6,245) | -37.2% (-52.4 to -14.9) | 378,228 (303,894 to 478,535) | -33.4% (-49.1 to -10.0) |
| Fiji | 381 (111 to 616) | 7.7% (-66.1 to 369.4) | 17 (12 to 23) | -12.7% (-38.5 to 24.3) | 2,220 (1,217 to 3,480) | -3.7% (-48.5 to 86.2) |
| Finland | 2,532 (765 to 3,994) | 8.4% (-70.3 to 205.2) | 126 (110 to 140) | 16.7% (3.5 to 31.1) | 7,784 (4,342 to 14,023) | -3.8% (-56.3 to 112.4) |
| France | 47,359 (16,632 to 74,013) | 8.8% (-60.2 to 280.8) | 2,011 (1,743 to 2,243) | -2.4% (-12.6 to 6.7) | 116,702 (65,076 to 214,831) | -10.1% (-52.8 to 83.5) |
| Gabon | 1,566 (334 to 2,705) | 9.5% (-71.5 to 428.1) | 48 (33 to 69) | -27.9% (-48.2 to 0.2) | 6,638 (3,389 to 10,844) | -18.5% (-63.0 to 59.9) |
| Gambia | 1,352 (302 to 2,437) | 15.6% (-75.9 to 510.4) | 76 (53 to 103) | 21.2% (-20.6 to 85.7) | 6,840 (4,204 to 9,853) | 16.3% (-33.1 to 121.1) |
| Georgia | 1,345 (409 to 2,187) | -4.9% (-75.0 to 258.9) | 64 (50 to 76) | 5.6% (-16.6 to 28.5) | 6,456 (3,461 to 10,518) | -15.5% (-59.3 to 74.8) |
| Germany | 74,433 (22,871 to 112,811) | 14.9% (-62.9 to 218.6) | 3,147 (2,765 to 3,455) | 69.5% (52.8 to 83.7) | 185,643 (98,911 to 356,086) | 5.9% (-47.7 to 144.9) |
| Ghana | 20,839 (5,733 to 35,587) | 22.5% (-68.9 to 500.0) | 620 (416 to 817) | 11.4% (-32.4 to 66.5) | 78,351 (41,633 to 127,923) | 13.0% (-46.4 to 137.4) |
| Greece | 4,020 (1,041 to 6,328) | 1.7% (-73.8 to 225.6) | 181 (164 to 196) | 107.6% (89.2 to 127.2) | 10,648 (5,776 to 19,089) | 15.2% (-52.3 to 205.6) |
| Greenland | 30 (10 to 47) | -3.0% (-70.2 to 241.2) | 2 (1 to 2) | -33.4% (-47.7 to -17.4) | 151 (81 to 259) | -29.3% (-64.7 to 40.6) |
| Grenada | 62 (17 to 102) | 7.8% (-66.5 to 311.2) | 2 (2 to 3) | -39.5% (-49.9 to -27.8) | 254 (138 to 413) | -28.1% (-64.3 to 46.0) |
| Guam | 61 (21 to 100) | 3.4% (-65.7 to 282.0) | 1 (0 to 1) | 42.2% (-44.6 to 133.0) | 201 (76 to 378) | 3.1% (-61.8 to 219.0) |
| Guatemala | 10,197 (2,788 to 16,910) | 10.3% (-69.1 to 404.5) | 327 (277 to 384) | -39.3% (-48.7 to -28.9) | 45,501 (24,813 to 73,550) | -23.9% (-61.9 to 52.0) |
| Guinea | 8,030 (1,687 to 16,067) | 10.3% (-75.5 to 499.5) | 401 (264 to 539) | 16.0% (-30.1 to 82.8) | 40,224 (22,795 to 65,629) | 7.4% (-42.1 to 107.7) |

| | | | | | | |
|---|---|---|---|---|---|---|
| Guinea-Bissau | 1,243<br>(239 to 2,341) | -0.1%<br>(-81.8 to 547.0) | 76<br>(53 to 102) | 9.6%<br>(-28.1 to 62.6) | 7,169<br>(4,197 to 11,060) | 0.8%<br>(-43.1 to 82.9) |
| Guyana | 494<br>(143 to 810) | 14.1%<br>(-68.3 to 291.3) | 26<br>(20 to 34) | -10.8%<br>(-35.8 to 16.5) | 2,625<br>(1,518 to 3,892) | -3.4%<br>(-47.5 to 77.5) |
| Haiti | 6,452<br>(1,574 to 11,690) | -7.6%<br>(-80.4 to 366.7) | 461<br>(338 to 633) | -28.0%<br>(-47.3 to -4.4) | 41,456<br>(25,671 to 61,579) | -27.2%<br>(-56.0 to 22.2) |
| Honduras | 5,930<br>(1,308 to 10,386) | -3.7%<br>(-78.8 to 407.7) | 263<br>(182 to 362) | -28.2%<br>(-50.0 to -0.5) | 29,360<br>(16,232 to 45,500) | -27.9%<br>(-62.2 to 45.9) |
| Hungary | 3,177<br>(1,018 to 5,092) | -5.3%<br>(-72.6 to 253.4) | 174<br>(139 to 212) | -12.8%<br>(-32.5 to 6.6) | 15,020<br>(7,858 to 26,371) | -25.9%<br>(-68.1 to 63.4) |
| Iceland | 168<br>(47 to 260) | 14.7%<br>(-65.8 to 265.5) | 5<br>(4 to 5) | 43.1%<br>(26.7 to 62.3) | 396<br>(206 to 755) | 10.6%<br>(-51.1 to 130.0) |
| India | 457,420<br>(325,186 to 602,651) | 6.7%<br>(-23.6 to 54.5) | 31,176<br>(22,983 to 35,983) | -31.2%<br>(-42.5 to -15.2) | 2,646,968<br>(2,052,364 to 3,304,933) | -29.0%<br>(-41.5 to -10.1) |
| Indonesia | 92,528<br>(61,423 to 124,849) | 16.3%<br>(-20.4 to 73.3) | 905<br>(218 to 1,422) | -10.2%<br>(-31.6 to 20.3) | 248,916<br>(144,392 to 364,035) | 2.0%<br>(-26.9 to 47.2) |
| Iran | 39,827<br>(27,032 to 52,554) | 13.4%<br>(-19.7 to 58.6) | 604<br>(543 to 769) | -55.1%<br>(-61.6 to -44.3) | 98,687<br>(67,059 to 139,669) | -40.0%<br>(-56.1 to -17.8) |
| Iraq | 19,238<br>(5,800 to 32,017) | 12.8%<br>(-68.7 to 361.3) | 314<br>(240 to 409) | -48.9%<br>(-61.0 to -33.5) | 52,367<br>(25,909 to 87,928) | -32.9%<br>(-68.9 to 28.7) |
| Ireland | 2,888<br>(887 to 4,474) | 12.2%<br>(-65.1 to 288.6) | 85<br>(74 to 95) | 7.6%<br>(-6.6 to 22.5) | 6,728<br>(3,555 to 12,479) | -3.5%<br>(-57.0 to 125.6) |
| Israel | 5,001<br>(1,654 to 7,850) | 13.0%<br>(-60.1 to 312.0) | 125<br>(111 to 139) | 38.1%<br>(22.6 to 54.8) | 11,159<br>(5,713 to 21,197) | 7.3%<br>(-49.8 to 146.8) |
| Italy | 22,897<br>(15,348 to 31,205) | -3.3%<br>(-27.6 to 26.6) | 1,541<br>(1,248 to 1,738) | 113.1%<br>(74.0 to 138.2) | 69,514<br>(49,195 to 100,631) | 8.1%<br>(-16.8 to 36.9) |
| Jamaica | 1,569<br>(444 to 2,585) | 0.2%<br>(-70.9 to 261.5) | 63<br>(46 to 82) | -1.3%<br>(-27.8 to 32.7) | 6,090<br>(3,192 to 9,979) | -11.8%<br>(-56.9 to 73.2) |
| Japan | 47,554<br>(31,556 to 64,782) | 11.5%<br>(-8.4 to 33.6) | 1,705<br>(1,452 to 1,860) | 108.0%<br>(97.8 to 117.9) | 120,161<br>(79,423 to 190,673) | 16.5%<br>(-4.0 to 43.3) |
| Jordan | 5,515<br>(1,556 to 8,939) | 3.3%<br>(-69.2 to 288.1) | 77<br>(63 to 97) | -55.0%<br>(-65.3 to -42.4) | 13,622<br>(6,589 to 24,303) | -37.8%<br>(-70.6 to 25.1) |
| Kazakhstan | 9,430<br>(2,663 to 15,433) | 6.9%<br>(-73.5 to 308.4) | 369<br>(322 to 426) | 27.1%<br>(6.7 to 49.4) | 43,810<br>(24,202 to 73,500) | 0.7%<br>(-51.1 to 125.4) |
| Kenya | 35,642<br>(25,413 to 47,859) | 5.2%<br>(-20.5 to 47.4) | 2,037<br>(1,584 to 2,527) | -8.8%<br>(-32.4 to 23.4) | 178,110<br>(137,309 to 220,747) | -3.2%<br>(-21.4 to 20.1) |
| Kiribati | 46<br>(11 to 82) | -5.2%<br>(-79.6 to 300.2) | 3<br>(2 to 4) | -11.5%<br>(-33.9 to 24.1) | 314<br>(187 to 474) | -13.1%<br>(-52.9 to 59.4) |
| Kuwait | 2,206<br>(585 to 3,563) | -0.5%<br>(-73.5 to 292.5) | 23<br>(19 to 28) | -43.3%<br>(-54.4 to -29.4) | 4,884<br>(1,967 to 9,507) | -28.6%<br>(-74.8 to 82.5) |
| Kyrgyzstan | 3,245<br>(879 to 5,504) | -4.9%<br>(-78.3 to 349.3) | 204<br>(167 to 246) | -2.6%<br>(-23.6 to 26.4) | 19,728<br>(12,491 to 29,756) | -15.5%<br>(-54.4 to 54.9) |
| Laos | 2,532<br>(641 to 4,327) | 14.4%<br>(-75.3 to 711.5) | 40<br>(24 to 81) | -32.7%<br>(-51.8 to -11.8) | 9,119<br>(3,591 to 16,393) | -12.0%<br>(-68.9 to 141.9) |

| | | | | | | |
|---|---|---|---|---|---|---|
| Latvia | 652<br>(202 to 1,035) | 10.0%<br>(-67.8 to 353.4) | 36<br>(27 to 45) | -32.7%<br>(-49.8 to -11.6) | 3,248<br>(1,754 to 5,746) | -21.9%<br>(-60.1 to 40.1) |
| Lebanon | 2,373<br>(737 to 4,005) | 7.9%<br>(-69.6 to 384.7) | 101<br>(83 to 125) | -42.3%<br>(-56.6 to -22.9) | 8,159<br>(4,654 to 13,262) | -37.0%<br>(-67.6 to 15.7) |
| Lesotho | 967<br>(208 to 1,740) | 32.8%<br>(-73.0 to 471.7) | 51<br>(37 to 68) | 30.1%<br>(-18.4 to 111.6) | 5,424<br>(3,076 to 8,094) | 37.5%<br>(-27.6 to 166.0) |
| Liberia | 3,101<br>(645 to 5,819) | -4.8%<br>(-82.1 to 457.8) | 148<br>(91 to 215) | -19.1%<br>(-50.5 to 24.2) | 14,729<br>(8,273 to 22,413) | -23.9%<br>(-62.5 to 42.6) |
| Libya | 2,826<br>(810 to 4,571) | 3.6%<br>(-71.8 to 457.8) | 70<br>(46 to 100) | -34.4%<br>(-53.8 to -8.1) | 8,771<br>(4,553 to 14,560) | -28.7%<br>(-65.7 to 55.0) |
| Lithuania | 1,005<br>(325 to 1,556) | 5.6%<br>(-68.7 to 280.1) | 67<br>(54 to 80) | -29.5%<br>(-44.0 to -13.9) | 5,584<br>(3,236 to 9,039) | -22.2%<br>(-56.5 to 37.8) |
| Luxembourg | 421<br>(141 to 651) | 7.3%<br>(-66.1 to 259.8) | 15<br>(13 to 18) | 6.8%<br>(-7.9 to 24.9) | 1,096<br>(585 to 1,956) | -10.5%<br>(-58.3 to 77.3) |
| Madagascar | 16,688<br>(4,354 to 31,234) | -4.7%<br>(-78.7 to 328.3) | 1,090<br>(820 to 1,470) | -19.9%<br>(-39.8 to 1.6) | 99,907<br>(65,835 to 144,986) | -15.5%<br>(-50.0 to 35.7) |
| Malawi | 11,651<br>(2,718 to 21,237) | 0.7%<br>(-75.0 to 391.5) | 1,094<br>(795 to 1,408) | -19.0%<br>(-36.0 to 1.3) | 86,598<br>(58,299 to 124,396) | -13.1%<br>(-42.8 to 32.8) |
| Malaysia | 13,612<br>(3,531 to 22,125) | 14.0%<br>(-67.4 to 282.3) | 216<br>(186 to 261) | -31.7%<br>(-42.1 to -17.0) | 43,470<br>(17,072 to 78,362) | -11.9%<br>(-67.0 to 107.9) |
| Maldives | 202<br>(61 to 346) | 5.0%<br>(-66.4 to 438.5) | 4<br>(3 to 8) | -48.0%<br>(-60.5 to -27.9) | 756<br>(321 to 1,374) | -33.1%<br>(-72.3 to 78.0) |
| Mali | 12,226<br>(2,788 to 23,661) | 27.1%<br>(-69.7 to 616.7) | 738<br>(488 to 1,012) | 10.0%<br>(-25.5 to 61.2) | 64,680<br>(37,824 to 98,313) | 14.8%<br>(-32.3 to 96.2) |
| Malta | 195<br>(57 to 314) | 14.8%<br>(-64.9 to 308.5) | 6<br>(5 to 6) | 50.2%<br>(32.1 to 71.0) | 453<br>(227 to 852) | 11.8%<br>(-50.2 to 171.8) |
| Marshall Islands | 19<br>(5 to 35) | 3.7%<br>(-79.3 to 408.1) | 1<br>(1 to 1) | -4.0%<br>(-33.3 to 35.4) | 108<br>(57 to 167) | -3.4%<br>(-54.6 to 104.9) |
| Mauritania | 2,681<br>(760 to 4,600) | 4.9%<br>(-73.1 to 395.9) | 103<br>(72 to 135) | -4.3%<br>(-38.2 to 38.5) | 10,669<br>(5,982 to 17,029) | -10.4%<br>(-53.3 to 72.6) |
| Mauritius | 706<br>(238 to 1,124) | 20.4%<br>(-64.9 to 476.6) | 52<br>(47 to 57) | 17.9%<br>(4.7 to 32.2) | 4,443<br>(2,647 to 6,998) | 17.3%<br>(-42.0 to 147.5) |
| Mexico | 97,906<br>(66,968 to 130,110) | -5.4%<br>(-28.9 to 22.7) | 2,590<br>(2,171 to 3,010) | -24.0%<br>(-35.9 to -12.0) | 347,067<br>(248,049 to 468,840) | -27.2%<br>(-42.6 to -10.5) |
| Micronesia (Federated States of) | 36<br>(7 to 62) | -0.7%<br>(-77.8 to 294.4) | 1<br>(1 to 2) | -16.9%<br>(-41.3 to 19.1) | 193<br>(100 to 310) | -14.0%<br>(-55.8 to 63.7) |
| Monaco | 20<br>(6 to 31) | 5.3%<br>(-68.0 to 259.6) | 0<br>(0 to 0) | 31.0%<br>(-22.9 to 96.8) | 43<br>(17 to 88) | 1.3%<br>(-61.4 to 154.1) |
| Mongolia | 1,499<br>(319 to 2,592) | 13.0%<br>(-74.3 to 436.7) | 59<br>(46 to 73) | -34.3%<br>(-49.0 to -14.0) | 7,458<br>(3,924 to 12,135) | -20.8%<br>(-59.5 to 53.7) |
| Montenegro | 201<br>(56 to 327) | -1.4%<br>(-72.3 to 259.8) | 5<br>(4 to 7) | -19.6%<br>(-36.9 to 4.2) | 710<br>(302 to 1,357) | -16.6%<br>(-64.8 to 88.9) |
| Morocco | 15,675<br>(3,773 to 27,016) | 9.9%<br>(-71.9 to 348.6) | 617<br>(448 to 779) | -37.7%<br>(-50.9 to -19.9) | 61,406<br>(35,318 to 99,584) | -27.3%<br>(-60.6 to 31.5) |

| | | | | | | |
|---|---|---|---|---|---|---|
| Mozambique | 20,223<br>(3,815 to 37,370) | 21.9%<br>(-74.8 to 614.8) | 1,437<br>(1,060 to 2,033) | -17.9%<br>(-38.8 to 8.5) | 129,022<br>(76,503 to 190,701) | -5.5%<br>(-42.8 to 50.1) |
| Myanmar | 21,074<br>(5,879 to 37,540) | 13.2%<br>(-70.8 to 464.1) | 520<br>(301 to 712) | -33.4%<br>(-51.9 to -7.1) | 85,397<br>(37,180 to 147,646) | -11.2%<br>(-62.3 to 109.4) |
| Namibia | 1,347<br>(403 to 2,377) | 13.9%<br>(-68.7 to 375.6) | 60<br>(43 to 85) | -9.4%<br>(-36.7 to 28.2) | 6,555<br>(3,787 to 10,215) | -3.7%<br>(-52.1 to 76.5) |
| Nauru | 5<br>(1 to 8) | -6.3%<br>(-76.1 to 361.3) | 0<br>(0 to 0) | 3.7%<br>(-25.8 to 43.6) | 24<br>(12 to 39) | -14.5%<br>(-61.2 to 99.9) |
| Nepal | 11,066<br>(2,421 to 19,669) | 14.4%<br>(-75.3 to 555.0) | 1,253<br>(839 to 1,676) | -19.8%<br>(-40.4 to 11.9) | 84,578<br>(52,279 to 128,683) | -23.7%<br>(-55.3 to 38.0) |
| Netherlands | 8,766<br>(2,585 to 13,716) | 18.1%<br>(-64.7 to 325.2) | 373<br>(330 to 412) | 32.3%<br>(20.0 to 46.1) | 23,869<br>(13,540 to 43,365) | 7.6%<br>(-49.3 to 144.8) |
| New Zealand | 2,377<br>(936 to 3,587) | -9.9%<br>(-62.6 to 132.7) | 62<br>(57 to 68) | -33.9%<br>(-39.9 to -27.9) | 6,211<br>(3,583 to 10,253) | -31.2%<br>(-60.1 to 17.1) |
| Nicaragua | 3,500<br>(841 to 6,540) | -3.6%<br>(-77.1 to 362.6) | 80<br>(67 to 99) | -39.0%<br>(-48.1 to -28.6) | 11,403<br>(5,742 to 20,317) | -35.2%<br>(-71.2 to 40.0) |
| Niger | 12,533<br>(2,162 to 26,972) | -2.6%<br>(-81.1 to 459.6) | 683<br>(390 to 1,084) | 9.3%<br>(-32.5 to 68.0) | 63,410<br>(33,606 to 101,665) | -0.7%<br>(-47.4 to 78.7) |
| Nigeria | 170,702<br>(118,786 to 224,352) | 11.7%<br>(-14.3 to 56.5) | 4,878<br>(2,880 to 6,788) | -4.6%<br>(-43.9 to 50.9) | 608,585<br>(436,414 to 811,012) | -1.9%<br>(-26.5 to 29.1) |
| Niue | 1<br>(0 to 1) | 7.0%<br>(-64.0 to 326.4) | 0<br>(0 to 0) | 13.4%<br>(-20.8 to 57.6) | 3<br>(2 to 5) | 6.2%<br>(-44.4 to 140.1) |
| North Macedonia | 714<br>(206 to 1,131) | -2.6%<br>(-75.7 to 202.0) | 42<br>(31 to 55) | -4.7%<br>(-38.4 to 35.8) | 3,649<br>(2,136 to 6,009) | -19.2%<br>(-61.2 to 61.2) |
| Northern Mariana Islands | 17<br>(4 to 29) | -7.2%<br>(-78.0 to 291.0) | 0<br>(0 to 0) | 87.7%<br>(-15.8 to 240.8) | 62<br>(26 to 112) | -7.0%<br>(-66.4 to 229.9) |
| Norway | 3,339<br>(2,025 to 4,611) | -0.1%<br>(-29.8 to 36.6) | 107<br>(98 to 114) | -14.1%<br>(-19.4 to -8.7) | 8,411<br>(5,449 to 13,010) | -19.2%<br>(-38.1 to 6.2) |
| Oman | 2,297<br>(683 to 3,650) | 27.3%<br>(-61.5 to 513.2) | 15<br>(10 to 19) | -37.6%<br>(-55.3 to -11.4) | 4,681<br>(1,695 to 8,985) | -11.0%<br>(-69.0 to 161.9) |
| Pakistan | 91,541<br>(44,758 to 138,269) | 5.4%<br>(-45.3 to 144.1) | 7,867<br>(6,098 to 10,253) | -0.1%<br>(-22.9 to 29.7) | 702,774<br>(537,940 to 933,247) | -5.1%<br>(-31.5 to 36.9) |
| Palau | 7<br>(2 to 11) | 2.6%<br>(-72.2 to 344.4) | 0<br>(0 to 0) | -9.1%<br>(-32.7 to 22.2) | 42<br>(24 to 65) | -8.3%<br>(-50.9 to 72.3) |
| Palestine | 2,498<br>(774 to 4,442) | 8.6%<br>(-72.1 to 567.9) | 71<br>(60 to 92) | -41.9%<br>(-53.9 to -28.2) | 8,579<br>(4,988 to 13,509) | -32.0%<br>(-64.5 to 25.0) |
| Panama | 3,014<br>(847 to 4,866) | 21.0%<br>(-66.2 to 525.1) | 63<br>(50 to 76) | -14.8%<br>(-32.2 to 4.7) | 9,829<br>(4,550 to 17,396) | -0.2%<br>(-56.6 to 151.1) |
| Papua New Guinea | 3,172<br>(775 to 5,779) | 1.0%<br>(-76.1 to 360.8) | 176<br>(118 to 263) | 17.2%<br>(-16.2 to 70.7) | 19,696<br>(10,732 to 30,527) | 8.2%<br>(-42.6 to 125.8) |
| Paraguay | 3,808<br>(1,253 to 6,275) | 9.9%<br>(-66.7 to 312.6) | 102<br>(72 to 134) | -0.9%<br>(-34.9 to 39.6) | 13,853<br>(7,143 to 23,066) | -6.6%<br>(-54.6 to 98.4) |
| Peru | 24,466<br>(6,969 to 40,938) | 1.6%<br>(-70.6 to 339.6) | 294<br>(221 to 404) | -58.1%<br>(-70.1 to -36.3) | 69,128<br>(27,761 to 128,959) | -39.0%<br>(-77.4 to 57.8) |

| | | | | | | |
|---|---|---|---|---|---|---|
| Philippines | 41,855<br>(29,016 to 56,497) | 4.0%<br>(-16.4 to 31.0) | 383<br>(287 to 455) | -8.2%<br>(-28.1 to 9.7) | 116,068<br>(78,784 to 164,451) | -3.5%<br>(-20.3 to 19.0) |
| Poland | 12,308<br>(8,037 to 16,538) | 13.0%<br>(-17.3 to 51.0) | 1,161<br>(941 to 1,326) | 83.4%<br>(48.6 to 108.7) | 74,691<br>(55,929 to 98,330) | 18.0%<br>(-6.6 to 47.2) |
| Portugal | 4,592<br>(1,220 to 7,126) | 17.4%<br>(-68.2 to 359.6) | 326<br>(281 to 360) | 29.5%<br>(14.5 to 43.5) | 14,936<br>(9,267 to 24,385) | -4.0%<br>(-48.4 to 97.3) |
| Puerto Rico | 1,818<br>(531 to 2,911) | -6.6%<br>(-72.1 to 202.3) | 57<br>(47 to 69) | -53.4%<br>(-62.1 to -43.6) | 5,878<br>(2,724 to 10,579) | -39.6%<br>(-73.9 to 19.6) |
| Qatar | 1,477<br>(485 to 2,385) | -6.7%<br>(-74.4 to 201.3) | 13<br>(10 to 19) | -59.2%<br>(-70.6 to -35.8) | 3,127<br>(1,242 to 6,046) | -45.2%<br>(-81.5 to 28.4) |
| South Korea | 21,404<br>(5,486 to 32,888) | 0.1%<br>(-72.5 to 219.4) | 564<br>(422 to 642) | -45.6%<br>(-62.5 to -34.8) | 56,166<br>(26,890 to 106,019) | -40.7%<br>(-73.2 to 29.6) |
| Republic of Moldova | 977<br>(291 to 1,613) | -13.2%<br>(-75.8 to 218.1) | 72<br>(59 to 87) | -38.1%<br>(-50.4 to -24.2) | 5,543<br>(3,542 to 8,420) | -39.2%<br>(-65.0 to 5.3) |
| Romania | 6,526<br>(1,925 to 10,401) | -0.5%<br>(-69.7 to 258.1) | 416<br>(322 to 496) | -3.2%<br>(-25.1 to 18.2) | 35,052<br>(19,816 to 56,062) | -20.5%<br>(-60.5 to 56.5) |
| Russian | 41,005<br>(27,958 to 54,875) | -10.7%<br>(-26.0 to 3.0) | 838<br>(754 to 919) | -41.6%<br>(-47.0 to -36.6) | 108,613<br>(71,621 to 162,868) | -36.9%<br>(-49.4 to -24.9) |
| Rwanda | 7,911<br>(1,724 to 14,528) | -3.6%<br>(-80.4 to 383.3) | 636<br>(467 to 904) | -29.1%<br>(-47.8 to -2.9) | 51,636<br>(34,412 to 75,020) | -28.5%<br>(-56.4 to 13.3) |
| Saint Kitts and Nevis | 39<br>(12 to 63) | -9.4%<br>(-72.5 to 271.4) | 1<br>(1 to 2) | -53.0%<br>(-62.4 to -41.6) | 163<br>(88 to 270) | -44.5%<br>(-73.1 to 13.9) |
| Saint Lucia | 107<br>(29 to 178) | -2.6%<br>(-76.2 to 280.8) | 6<br>(5 to 7) | -40.2%<br>(-51.7 to -27.9) | 520<br>(299 to 853) | -31.7%<br>(-66.0 to 39.8) |
| Saint Vincent and the Grenadines | 69<br>(17 to 113) | 10.2%<br>(-70.1 to 441.0) | 4<br>(3 to 5) | -18.9%<br>(-32.8 to -3.0) | 352<br>(211 to 524) | -13.3%<br>(-53.9 to 55.1) |
| Samoa | 80<br>(24 to 136) | 5.2%<br>(-72.1 to 401.4) | 2<br>(2 to 3) | -14.1%<br>(-39.1 to 26.6) | 341<br>(175 to 566) | -8.0%<br>(-56.3 to 90.1) |
| San Marino | 13<br>(4 to 20) | 0.2%<br>(-70.3 to 236.2) | 0<br>(0 to 0) | -45.7%<br>(-63.5 to -23.9) | 22<br>(7 to 47) | -11.7%<br>(-75.0 to 189.1) |
| São Tomé and Príncipe | 141<br>(41 to 243) | 25.1%<br>(-68.8 to 533.5) | 6<br>(4 to 8) | 9.5%<br>(-24.9 to 71.1) | 604<br>(303 to 953) | 10.8%<br>(-42.8 to 128.2) |
| Saudi Arabia | 24,102<br>(6,271 to 39,558) | 25.9%<br>(-65.0 to 419.3) | 543<br>(329 to 731) | -39.1%<br>(-57.1 to -15.5) | 80,462<br>(37,741 to 144,059) | -19.4%<br>(-63.1 to 73.9) |
| Senegal | 9,736<br>(2,078 to 18,156) | 11.4%<br>(-77.0 to 682.6) | 539<br>(376 to 726) | 16.1%<br>(-20.8 to 68.1) | 50,423<br>(29,474 to 77,321) | 11.2%<br>(-42.6 to 121.5) |
| Serbia | 3,444<br>(980 to 5,513) | -6.4%<br>(-72.3 to 251.1) | 170<br>(123 to 209) | -32.9%<br>(-49.8 to -14.2) | 14,775<br>(7,750 to 26,085) | -32.6%<br>(-68.8 to 32.4) |
| Seychelles | 41<br>(12 to 70) | 10.5%<br>(-65.6 to 293.1) | 1<br>(1 to 1) | -40.0%<br>(-50.8 to -26.7) | 156<br>(73 to 265) | -18.5%<br>(-65.6 to 66.0) |
| Sierra Leone | 4,916<br>(974 to 9,070) | 4.5%<br>(-78.0 to 468.7) | 254<br>(156 to 360) | 11.0%<br>(-29.7 to 67.2) | 24,982<br>(13,071 to 38,676) | 3.6%<br>(-43.7 to 88.1) |
| Singapore | 2,099<br>(603 to 3,370) | 13.7%<br>(-71.2 to 324.3) | 29<br>(25 to 32) | 8.8%<br>(-4.3 to 22.6) | 4,420<br>(1,834 to 9,156) | -7.1%<br>(-66.6 to 145.7) |

| | | | | | | |
|---|---|---|---|---|---|---|
| Slovakia | 2,148 (623 to 3,532) | 6.7% (-69.6 to 233.2) | 136 (103 to 167) | -13.7% (-34.8 to 12.1) | 11,740 (6,646 to 19,713) | -13.9% (-56.8 to 81.3) |
| Slovenia | 718 (221 to 1,132) | -9.9% (-74.4 to 195.8) | 44 (31 to 56) | -2.2% (-31.4 to 25.7) | 3,202 (1,765 to 5,439) | -26.3% (-67.0 to 64.9) |
| Solomon Islands | 225 (51 to 410) | 4.1% (-76.7 to 396.3) | 11 (8 to 16) | 6.4% (-23.5 to 54.4) | 1,322 (743 to 2,091) | 1.6% (-46.0 to 116.3) |
| Somalia | 9,108 (1,372 to 19,866) | -6.9% (-83.8 to 585.8) | 1,291 (796 to 2,109) | -0.9% (-23.6 to 30.6) | 94,814 (56,205 to 146,348) | -1.9% (-35.3 to 39.5) |
| South Africa | 33,214 (20,311 to 46,183) | -1.3% (-35.7 to 53.6) | 1,179 (1,006 to 1,356) | -11.9% (-23.5 to 1.8) | 132,325 (96,364 to 178,256) | -16.4% (-36.9 to 10.9) |
| South Sudan | 5,622 (1,248 to 10,953) | -10.3% (-83.2 to 302.7) | 563 (395 to 772) | 0.8% (-23.7 to 40.2) | 44,306 (30,068 to 63,012) | 2.1% (-37.5 to 60.7) |
| Spain | 17,767 (5,790 to 28,706) | 5.4% (-66.2 to 322.0) | 818 (691 to 915) | 45.4% (27.1 to 62.3) | 46,938 (25,478 to 86,310) | -2.6% (-54.8 to 123.3) |
| Sri Lanka | 11,228 (2,760 to 18,557) | 5.5% (-76.4 to 333.5) | 347 (234 to 486) | -54.2% (-69.2 to -34.4) | 44,289 (19,810 to 77,271) | -33.9% (-72.6 to 39.7) |
| Sudan | 19,218 (5,103 to 33,204) | 12.3% (-71.6 to 455.5) | 623 (421 to 853) | -37.9% (-56.7 to -10.5) | 79,238 (48,142 to 122,131) | -27.5% (-60.8 to 30.9) |
| Suriname | 376 (122 to 606) | 10.3% (-64.8 to 439.2) | 16 (12 to 20) | -26.7% (-43.5 to -2.7) | 1,760 (1,025 to 2,766) | -15.8% (-56.3 to 70.4) |
| Sweden | 3,689 (1,573 to 5,654) | -11.2% (-60.0 to 110.0) | 118 (103 to 134) | -25.2% (-34.9 to -13.6) | 9,557 (5,508 to 16,370) | -26.5% (-59.4 to 34.6) |
| Switzerland | 4,415 (1,266 to 6,831) | -2.8% (-70.4 to 246.5) | 175 (147 to 203) | 3.7% (-20.2 to 25.4) | 11,013 (5,994 to 19,564) | -14.4% (-61.7 to 94.2) |
| Syrian Arab Republic | 5,147 (1,473 to 8,531) | 9.3% (-71.0 to 416.0) | 145 (100 to 201) | -15.6% (-43.6 to 24.4) | 17,116 (9,951 to 28,228) | -12.4% (-53.7 to 88.5) |
| Türkiye | 43,973 (13,647 to 72,076) | 15.7% (-67.1 to 350.2) | 1,479 (1,183 to 1,818) | -52.1% (-61.9 to -39.0) | 153,904 (87,880 to 251,337) | -45.5% (-70.7 to 0.1) |
| Taiwan (province of China) | 7,721 (2,127 to 11,774) | 11.6% (-67.9 to 312.4) | 299 (271 to 326) | 19.5% (7.6 to 31.1) | 32,140 (15,708 to 57,965) | -3.6% (-56.4 to 143.2) |
| Tajikistan | 4,869 (1,338 to 8,428) | -10.0% (-74.9 to 257.0) | 467 (344 to 621) | -4.1% (-28.8 to 30.9) | 42,944 (28,869 to 60,112) | -10.5% (-46.7 to 44.6) |
| Thailand | 25,162 (7,863 to 40,960) | 31.2% (-60.0 to 483.8) | 989 (755 to 1,241) | -18.0% (-42.8 to 10.6) | 103,216 (51,404 to 180,179) | 5.5% (-47.4 to 134.3) |
| Timor-Leste | 522 (145 to 918) | 16.4% (-67.2 to 567.4) | 7 (5 to 14) | -23.8% (-43.6 to 1.0) | 1,734 (668 to 3,199) | -5.2% (-62.4 to 145.5) |
| Togo | 5,184 (1,166 to 9,782) | -10.8% (-78.8 to 343.0) | 241 (153 to 335) | 11.6% (-29.4 to 65.1) | 24,271 (13,213 to 38,099) | -11.9% (-53.2 to 88.2) |
| Tokelau | 0 (0 to 1) | 5.6% (-77.2 to 384.7) | 0 (0 to 0) | 32.3% (-4.4 to 82.5) | 3 (2 to 4) | 19.7% (-34.8 to 124.9) |
| Tonga | 37 (11 to 60) | 6.7% (-72.6 to 333.4) | 1 (1 to 1) | -13.5% (-35.8 to 22.3) | 147 (78 to 240) | -4.0% (-51.2 to 104.7) |
| Trinidad and Tobago | 933 (265 to 1,537) | 3.5% (-68.7 to 296.9) | 38 (28 to 48) | -35.6% (-52.0 to -17.2) | 4,140 (2,231 to 7,015) | -22.3% (-60.9 to 60.4) |

| | | | | | | |
|---|---|---|---|---|---|---|
| Tunisia | 4,598 (1,332 to 7,796) | 17.8% (-70.6 to 373.4) | 113 (82 to 161) | -42.0% (-57.3 to -22.2) | 13,331 (6,992 to 23,207) | -31.4% (-66.7 to 35.5) |
| Turkmenistan | 2,504 (598 to 4,328) | 11.9% (-73.6 to 278.1) | 148 (100 to 206) | 47.3% (-2.8 to 114.8) | 16,092 (9,040 to 25,360) | 22.5% (-38.7 to 139.8) |
| Tuvalu | 4 (1 to 7) | 3.4% (-73.1 to 297.4) | 0 (0 to 0) | -18.5% (-36.8 to 4.4) | 21 (11 to 33) | -14.2% (-57.2 to 76.8) |
| Uganda | 32,443 (6,989 to 58,736) | 10.3% (-76.3 to 595.5) | 1,553 (1,163 to 2,046) | -13.4% (-38.5 to 21.9) | 156,048 (98,147 to 235,170) | -1.6% (-45.0 to 78.0) |
| Ukraine | 12,868 (4,047 to 20,913) | -11.2% (-72.0 to 199.8) | 425 (303 to 553) | -30.3% (-49.4 to -8.5) | 49,970 (26,971 to 82,168) | -26.6% (-66.1 to 46.6) |
| United Arab Emirates | 5,335 (1,735 to 8,863) | -3.0% (-68.4 to 228.4) | 48 (38 to 60) | -60.2% (-68.7 to -43.6) | 15,054 (5,805 to 28,385) | -41.8% (-76.3 to 31.5) |
| UK | 42,922 (29,133 to 57,869) | 5.5% (-10.0 to 20.0) | 1,067 (972 to 1,131) | -24.3% (-30.8 to -20.0) | 102,392 (70,294 to 151,734) | -17.3% (-30.3 to -4.9) |
| Tanzania | 40,002 (9,568 to 72,527) | 13.6% (-67.2 to 520.9) | 2,890 (2,209 to 3,715) | -24.9% (-46.0 to -3.1) | 254,132 (164,749 to 371,644) | -12.2% (-44.9 to 42.4) |
| USA | 131,613 (82,178 to 180,640) | 16.5% (-10.0 to 39.9) | 3,426 (3,214 to 3,570) | 29.3% (24.7 to 33.0) | 386,335 (245,396 to 600,739) | 9.0% (-11.8 to 34.8) |
| Virgin Islands | 50 (16 to 78) | 0.0% (-69.5 to 306.4) | 1 (1 to 2) | -18.5% (-40.2 to 8.9) | 193 (90 to 330) | -10.7% (-59.8 to 95.4) |
| Uruguay | 1,779 (421 to 2,893) | 14.5% (-72.9 to 309.8) | 73 (67 to 79) | 18.5% (6.4 to 30.4) | 6,417 (3,532 to 10,629) | 2.1% (-52.4 to 115.5) |
| Uzbekistan | 17,179 (4,639 to 27,932) | 14.9% (-65.8 to 391.2) | 902 (761 to 1,067) | 0.0% (-18.2 to 21.9) | 103,303 (65,442 to 160,865) | 3.2% (-41.1 to 91.0) |
| Vanuatu | 104 (23 to 181) | 4.0% (-75.5 to 419.2) | 5 (3 to 6) | 8.4% (-22.1 to 57.8) | 570 (299 to 892) | 4.6% (-47.9 to 112.0) |
| Venezuela | 16,559 (5,129 to 28,253) | -5.0% (-73.1 to 326.6) | 611 (452 to 776) | -4.5% (-29.9 to 20.1) | 70,432 (36,755 to 122,188) | -16.4% (-60.1 to 93.4) |
| Viet Nam | 32,978 (7,580 to 56,040) | 28.1% (-72.0 to 526.6) | 79 (15 to 326) | -20.6% (-34.2 to 10.2) | 77,438 (17,792 to 164,318) | 11.3% (-76.6 to 352.6) |
| Yemen | 12,930 (2,526 to 24,018) | 7.7% (-79.8 to 641.9) | 441 (274 to 612) | -26.0% (-46.2 to 3.7) | 54,109 (28,194 to 85,439) | -21.6% (-57.1 to 61.3) |
| Zambia | 16,067 (4,093 to 28,828) | 10.1% (-68.8 to 411.1) | 1,706 (1,215 to 2,358) | 45.1% (3.4 to 99.3) | 134,735 (88,958 to 188,670) | 34.5% (-15.7 to 133.5) |
| Zimbabwe | 8,260 (1,964 to 15,310) | 3.4% (-75.9 to 407.9) | 530 (341 to 773) | 13.8% (-24.9 to 61.2) | 50,411 (29,431 to 75,589) | 15.1% (-37.6 to 101.8) |

**Table 2. Age-standardised incidence, prevalence, death, and DALY rates of idiopathic epilepsy per 100,000 people by seven GBD super-regions, 21 GBD regions, and 204 countries/territories, both sexes, 2021**

| Region, country | Incidence rate (95% UI) | Prevalence rate (95% UI) | Death rate (95% UI) | DALYs rate (95% UI) |
|---|---|---|---|---|
| Global | 42.8 (31.2 to 53.7) | 307.4 (234.7 to 389.0) | 1.7 (1.5 to 1.9) | 177.8 (137.7 to 225.9) |
| High income region | 52.5 (34.8 to 71.7) | 343.7 (233.4 to 454.9) | 1.1 (1.0 to 1.1) | 119.1 (79.4 to 179.9) |
| High income Asia Pacific | 45.0 (27.4 to 62.6) | 276.7 (168.6 to 384.4) | 0.7 (0.7 to 0.7) | 92.9 (59.1 to 144.3) |
| High income North America | 42.2 (27.0 to 59.6) | 334.7 (218.9 to 460.7) | 0.8 (0.7 to 0.8) | 109.4 (69.2 to 168.0) |
| Southern Latin America | 51.8 (25.0 to 78.4) | 339.7 (161.0 to 515.6) | 1.0 (0.9 to 1.1) | 136.7 (80.7 to 216.4) |
| Western Europe | 65.3 (41.3 to 89.1) | 381.1 (238.1 to 502.5) | 1.4 (1.3 to 1.5) | 135.0 (91.1 to 204.0) |
| Australasia | 48.9 (19.8 to 75.9) | 316.1 (123.3 to 491.0) | 0.9 (0.9 to 1.0) | 111.6 (60.8 to 199.7) |
| Latin America and Caribbean | 63.2 (45.4 to 82.4) | 470.6 (346.3 to 607.6) | 1.7 (1.6 to 1.9) | 220.7 (164.8 to 296.9) |
| Central Latin America | 69.7 (48.4 to 92.2) | 537.4 (384.2 to 714.3) | 1.9 (1.7 to 2.2) | 250.7 (179.2 to 337.2) |
| Tropical Latin America | 55.2 (37.1 to 73.7) | 389.5 (262.4 to 529.0) | 1.5 (1.5 to 1.6) | 183.1 (130.2 to 256.0) |
| Andean Latin America | 71.7 (38.7 to 103.6) | 561.9 (309.6 to 821.7) | 1.4 (1.2 to 1.7) | 232.5 (144.5 to 347.5) |
| Caribbean | 52.5 (32.9 to 73.7) | 371.1 (236.3 to 507.4) | 2.0 (1.7 to 2.5) | 220.7 (159.6 to 303.9) |
| Sub-Saharan Africa | 56.8 (40.1 to 74.0) | 386.3 (286.7 to 491.3) | 4.9 (4.1 to 5.7) | 345.3 (274.3 to 426.0) |
| Southern sub-Saharan Africa | 56.6 (36.8 to 78.6) | 403.7 (271.2 to 545.3) | 2.5 (2.2 to 3.1) | 257.2 (195.2 to 330.5) |

| | | | | |
|---|---|---|---|---|
| **Western sub-Saharan Africa** | 59.3<br>(42.2 to 77.3) | 404.5<br>(293.9 to 526.1) | 3.6<br>(2.4 to 4.5) | 299.7<br>(216.0 to 386.8) |
| **Central sub-Saharan Africa** | 55.6<br>(22.4 to 88.9) | 394.3<br>(177.8 to 625.8) | 3.9<br>(2.9 to 5.4) | 334.5<br>(220.9 to 477.2) |
| **Eastern sub-Saharan Africa** | 54.0<br>(36.0 to 73.2) | 355.2<br>(242.0 to 477.8) | 7.5<br>(6.4 to 8.9) | 423.5<br>(345.1 to 519.7) |
| **North Africa and Middle East** | 47.4<br>(32.3 to 66.7) | 316.2<br>(217.5 to 430.7) | 1.3<br>(1.1 to 1.5) | 158.6<br>(113.9 to 219.8) |
| **Southeast Asia, east Asia, and Oceania** | 31.5<br>(22.0 to 40.9) | 232.2<br>(168.0 to 298.2) | 0.7<br>(0.6 to 0.8) | 104.6<br>(74.9 to 142.6) |
| **Southeast Asia** | 37.1<br>(25.4 to 50.8) | 263.0<br>(186.3 to 349.7) | 0.6<br>(0.4 to 0.7) | 110.5<br>(75.1 to 155.6) |
| **Oceania** | 29.7<br>(11.9 to 48.4) | 248.8<br>(111.2 to 390.5) | 1.7<br>(1.3 to 2.5) | 187.9<br>(116.0 to 276.4) |
| **East Asia** | 28.2<br>(19.2 to 37.4) | 216.1<br>(149.9 to 279.2) | 0.8<br>(0.7 to 1.0) | 102.0<br>(74.1 to 138.7) |
| **Central Europe, eastern Europe, and central Asia** | 39.4<br>(27.1 to 52.7) | 326.3<br>(228.0 to 425.3) | 1.5<br>(1.3 to 1.6) | 163.0<br>(125.0 to 213.4) |
| **Central Asia** | 47.4<br>(27.5 to 66.2) | 449.7<br>(262.4 to 619.7) | 2.6<br>(2.3 to 2.9) | 280.7<br>(214.2 to 367.4) |
| **Eastern Europe** | 33.2<br>(22.0 to 45.3) | 226.5<br>(152.2 to 303.9) | 0.6<br>(0.5 to 0.7) | 87.1<br>(58.1 to 124.7) |
| **Central Europe** | 41.9<br>(28.0 to 59.0) | 387.9<br>(266.4 to 516.7) | 1.8<br>(1.5 to 2.0) | 174.6<br>(127.6 to 239.8) |
| **South Asia** | 33.1<br>(23.4 to 42.5) | 259.7<br>(190.7 to 329.9) | 2.6<br>(1.9 to 2.9) | 199.2<br>(156.4 to 246.2) |
| | | | | |
| **Countries** | | | | |
| Afghanistan | 43.9<br>(10.2 to 83.9) | 317.9<br>(70.6 to 594.2) | 3.3<br>(2.5 to 4.3) | 293.1<br>(182.3 to 448.9) |
| Albania | 43.6<br>(11.1 to 70.7) | 437.1<br>(113.6 to 705.2) | 2.0<br>(1.5 to 2.9) | 215.2<br>(123.5 to 367.3) |

| | | | | |
|---|---|---|---|---|
| Algeria | 43.9 (11.8 to 74.7) | 284.4 (72.0 to 471.3) | 1.1 (0.9 to 1.5) | 139.9 (73.0 to 230.3) |
| American Samoa | 40.3 (10.5 to 65.5) | 366.2 (92.7 to 594.3) | 1.4 (1.0 to 1.7) | 195.8 (104.3 to 317.1) |
| Andorra | 59.6 (17.2 to 92.1) | 348.7 (100.7 to 530.1) | 0.7 (0.4 to 0.9) | 103.6 (46.1 to 201.8) |
| Angola | 69.3 (18.7 to 119.9) | 542.0 (142.7 to 917.0) | 3.9 (2.8 to 5.1) | 388.6 (208.2 to 604.8) |
| Antigua and Barbuda | 79.1 (21.1 to 126.7) | 662.3 (195.6 to 1,027.4) | 3.2 (3.0 to 3.5) | 322.8 (184.9 to 523.2) |
| Argentina | 45.6 (11.4 to 77.2) | 277.7 (71.5 to 454.7) | 0.8 (0.7 to 0.8) | 113.5 (53.0 to 205.4) |
| Armenia | 38.0 (12.3 to 64.0) | 296.5 (95.5 to 496.2) | 0.8 (0.6 to 1.0) | 120.5 (57.7 to 211.4) |
| Australia | 48.3 (13.7 to 79.7) | 316.5 (100.8 to 512.0) | 0.9 (0.8 to 1.0) | 109.4 (55.7 to 209.2) |
| Austria | 57.4 (16.7 to 90.4) | 332.7 (99.5 to 511.7) | 1.1 (1.0 to 1.1) | 112.0 (53.4 to 207.7) |
| Azerbaijan | 47.1 (14.0 to 76.8) | 449.6 (133.3 to 745.2) | 2.4 (1.7 to 3.3) | 263.8 (150.0 to 417.9) |
| Bahamas | 65.5 (20.6 to 105.0) | 493.1 (155.9 to 765.5) | 1.5 (1.2 to 1.9) | 219.1 (101.5 to 367.3) |
| Bahrain | 59.1 (17.2 to 97.8) | 417.2 (115.7 to 663.3) | 1.6 (1.3 to 2.0) | 178.6 (92.2 to 310.9) |
| Bangladesh | 25.7 (7.4 to 45.5) | 199.3 (59.0 to 356.3) | 1.1 (0.8 to 1.4) | 108.0 (57.9 to 184.0) |
| Barbados | 63.1 (19.1 to 102.5) | 463.0 (148.0 to 739.6) | 1.7 (1.3 to 2.1) | 199.8 (105.0 to 335.7) |
| Belarus | 31.1 (8.6 to 51.6) | 234.2 (65.1 to 384.3) | 0.9 (0.7 to 1.1) | 99.0 (52.2 to 175.6) |
| Belgium | 66.7 (19.5 to 102.2) | 425.6 (125.9 to 656.7) | 1.9 (1.7 to 2.1) | 164.6 (90.4 to 293.7) |
| Belize | 55.8 (15.7 to 97.3) | 401.3 (116.3 to 686.1) | 1.8 (1.5 to 2.1) | 210.0 (107.5 to 348.7) |

| | | | | |
|---|---|---|---|---|
| Benin | 55.0 (13.0 to 100.6) | 391.2 (96.6 to 719.4) | 4.0 (2.6 to 5.5) | 316.6 (180.3 to 492.2) |
| Bermuda | 62.6 (19.6 to 102.6) | 452.6 (139.4 to 709.8) | 0.9 (0.8 to 1.1) | 144.5 (60.9 to 281.6) |
| Bhutan | 31.8 (8.5 to 57.8) | 297.6 (78.9 to 525.4) | 2.7 (1.8 to 3.9) | 222.5 (127.0 to 348.0) |
| Bolivia | 48.6 (12.5 to 82.0) | 421.1 (105.8 to 704.0) | 2.0 (1.4 to 2.8) | 242.8 (126.1 to 392.9) |
| Bosnia and Herzegovina | 41.0 (11.2 to 69.1) | 379.1 (107.0 to 617.1) | 1.8 (1.2 to 2.3) | 176.7 (97.1 to 293.3) |
| Botswana | 63.4 (18.2 to 106.3) | 513.1 (142.2 to 844.8) | 2.9 (2.1 to 3.7) | 318.3 (176.7 to 482.5) |
| Brazil | 55.3 (37.3 to 74.2) | 388.9 (262.2 to 526.5) | 1.5 (1.5 to 1.6) | 182.6 (129.9 to 254.6) |
| Brunei | 56.4 (17.5 to 88.9) | 418.3 (132.5 to 644.5) | 1.2 (1.0 to 1.4) | 174.6 (85.5 to 292.4) |
| Bulgaria | 43.5 (12.0 to 71.1) | 429.1 (118.8 to 682.0) | 2.0 (1.5 to 2.6) | 209.0 (115.4 to 339.5) |
| Burkina Faso | 50.8 (9.2 to 99.0) | 362.1 (66.6 to 692.2) | 4.4 (2.7 to 6.1) | 315.9 (165.2 to 491.8) |
| Burundi | 47.7 (9.2 to 93.7) | 306.8 (57.7 to 602.2) | 8.5 (6.4 to 11.4) | 427.1 (289.8 to 607.8) |
| Côte d’Ivoire | 60.7 (14.5 to 109.1) | 446.6 (109.5 to 797.9) | 4.1 (2.5 to 5.6) | 336.5 (187.6 to 530.1) |
| Cabo Verde | 66.9 (18.0 to 115.6) | 500.8 (125.8 to 858.8) | 3.3 (2.5 to 4.2) | 287.7 (153.5 to 471.7) |
| Cambodia | 32.1 (10.0 to 58.7) | 233.4 (73.5 to 429.0) | 0.7 (0.4 to 1.2) | 114.4 (50.0 to 195.9) |
| Cameroon | 57.8 (14.9 to 102.3) | 402.8 (99.8 to 694.6) | 3.9 (2.3 to 5.5) | 313.9 (176.3 to 472.1) |
| Canada | 35.3 (9.0 to 57.5) | 272.3 (76.4 to 430.2) | 0.8 (0.7 to 0.8) | 93.1 (46.6 to 173.7) |
| Central African Republic | 47.9 (9.9 to 89.4) | 325.7 (65.0 to 600.6) | 5.6 (4.0 to 7.8) | 414.5 (267.6 to 602.2) |

| | | | | |
|---|---|---|---|---|
| Chad | 49.3 (8.7 to 97.3) | 342.1 (60.3 to 649.1) | 5.1 (3.4 to 7.0) | 350.9 (206.9 to 522.6) |
| Chile | 66.3 (19.9 to 107.2) | 478.8 (148.1 to 773.6) | 1.4 (1.3 to 1.6) | 183.6 (92.4 to 331.9) |
| China | 28.2 (19.0 to 37.9) | 214.7 (150.1 to 278.6) | 0.8 (0.7 to 1.0) | 101.4 (72.5 to 139.4) |
| Colombia | 64.4 (16.2 to 110.3) | 504.4 (135.2 to 858.9) | 1.4 (1.2 to 1.7) | 206.3 (98.7 to 371.2) |
| Comoros | 57.1 (17.1 to 101.0) | 378.8 (108.6 to 661.6) | 7.2 (5.3 to 10.2) | 419.9 (273.3 to 596.3) |
| Congo | 67.6 (18.9 to 115.0) | 515.4 (144.1 to 887.6) | 3.6 (2.5 to 4.8) | 356.2 (194.6 to 544.2) |
| Cook Islands | 41.0 (12.9 to 66.5) | 364.6 (115.3 to 578.1) | 0.6 (0.5 to 0.9) | 136.0 (62.4 to 247.2) |
| Costa Rica | 63.5 (18.8 to 104.7) | 495.7 (150.0 to 804.0) | 1.7 (1.5 to 1.9) | 203.7 (107.1 to 344.6) |
| Croatia | 45.3 (11.9 to 73.5) | 443.8 (122.6 to 705.1) | 1.6 (1.1 to 2.0) | 172.1 (83.8 to 318.5) |
| Cuba | 44.0 (12.2 to 75.4) | 270.1 (78.5 to 445.2) | 0.8 (0.7 to 0.9) | 103.6 (47.7 to 190.5) |
| Cyprus | 51.1 (14.0 to 82.3) | 287.6 (81.0 to 450.5) | 0.7 (0.6 to 0.8) | 87.6 (39.4 to 164.6) |
| Czechia | 46.1 (13.6 to 75.7) | 450.3 (126.9 to 694.6) | 1.7 (1.3 to 2.1) | 175.1 (90.6 to 312.6) |
| North Korea | 21.7 (5.9 to 38.7) | 177.8 (47.7 to 304.9) | 0.9 (0.7 to 1.5) | 110.0 (60.7 to 183.0) |
| Democratic Republic of the Congo | 49.3 (10.4 to 91.1) | 327.0 (73.1 to 584.1) | 3.9 (2.7 to 5.7) | 307.6 (184.2 to 474.2) |
| Denmark | 42.6 (11.1 to 66.9) | 266.1 (74.9 to 413.4) | 1.4 (1.2 to 1.5) | 107.0 (60.6 to 189.6) |
| Djibouti | 61.9 (16.5 to 110.3) | 429.9 (113.8 to 738.0) | 6.8 (5.0 to 9.5) | 415.5 (266.6 to 596.9) |
| Dominica | 77.6 (27.2 to 123.4) | 665.6 (224.1 to 1,029.3) | 3.6 (2.8 to 4.7) | 386.8 (225.0 to 604.4) |

| | | | | |
|---|---|---|---|---|
| Dominican Republic | 58.0<br>(16.4 to 98.5) | 423.0<br>(129.2 to 696.3) | 1.4<br>(1.1 to 1.9) | 207.0<br>(99.5 to 358.3) |
| Ecuador | 94.9<br>(29.9 to 160.5) | 710.8<br>(226.2 to 1,140.8) | 2.2<br>(1.5 to 2.9) | 313.9<br>(155.0 to 524.7) |
| Egypt | 46.3<br>(12.0 to 77.7) | 303.1<br>(81.0 to 484.3) | 0.8<br>(0.6 to 1.0) | 132.7<br>(59.7 to 240.4) |
| El Salvador | 55.0<br>(13.2 to 92.6) | 401.8<br>(98.6 to 658.9) | 1.0<br>(0.8 to 1.3) | 165.9<br>(74.2 to 292.8) |
| Equatorial Guinea | 84.9<br>(20.6 to 140.9) | 706.0<br>(172.0 to 1,150.4) | 2.9<br>(1.9 to 4.4) | 370.1<br>(185.0 to 625.8) |
| Eritrea | 55.8<br>(13.9 to 102.5) | 380.1<br>(98.2 to 676.6) | 8.6<br>(6.4 to 11.4) | 486.4<br>(329.2 to 688.8) |
| Estonia | 44.9<br>(13.7 to 69.5) | 420.7<br>(126.8 to 632.4) | 1.7<br>(1.4 to 2.1) | 177.5<br>(97.6 to 298.5) |
| Eswatini | 56.6<br>(13.6 to 98.5) | 449.0<br>(107.6 to 751.3) | 3.4<br>(2.3 to 4.6) | 329.0<br>(195.4 to 508.7) |
| Ethiopia | 41.4<br>(21.9 to 62.3) | 257.5<br>(139.1 to 372.4) | 7.6<br>(6.2 to 9.2) | 372.6<br>(302.9 to 464.0) |
| Fiji | 41.2<br>(12.1 to 66.5) | 381.3<br>(111.3 to 598.0) | 1.9<br>(1.4 to 2.6) | 239.8<br>(130.8 to 376.7) |
| Finland | 52.4<br>(15.7 to 84.1) | 336.3<br>(91.5 to 529.4) | 1.3<br>(1.2 to 1.5) | 126.4<br>(67.9 to 229.2) |
| France | 76.2<br>(26.3 to 120.9) | 426.9<br>(155.3 to 661.3) | 1.7<br>(1.5 to 1.8) | 152.6<br>(83.6 to 292.8) |
| Gabon | 82.8<br>(17.7 to 140.9) | 688.2<br>(146.3 to 1,158.0) | 3.1<br>(2.2 to 4.3) | 374.9<br>(194.3 to 615.7) |
| Gambia | 51.0<br>(11.7 to 90.3) | 334.6<br>(77.4 to 578.0) | 4.5<br>(3.1 to 6.1) | 316.9<br>(201.2 to 452.6) |
| Georgia | 41.5<br>(12.5 to 68.4) | 357.5<br>(110.3 to 573.4) | 1.5<br>(1.2 to 1.8) | 177.8<br>(96.1 to 291.4) |
| Germany | 91.8<br>(27.5 to 140.5) | 538.6<br>(163.0 to 815.6) | 1.9<br>(1.7 to 2.0) | 178.8<br>(88.8 to 355.0) |
| Ghana | 56.4<br>(15.1 to 95.6) | 387.6<br>(104.4 to 634.9) | 2.4<br>(1.6 to 3.1) | 239.2<br>(129.5 to 383.3) |

| | | | | |
|---|---|---|---|---|
| Greece | 47.9 (12.4 to 76.1) | 267.3 (68.7 to 422.8) | 0.9 (0.9 to 1.0) | 95.4 (48.2 to 179.7) |
| Greenland | 53.8 (17.9 to 86.2) | 554.6 (167.4 to 870.9) | 2.4 (1.6 to 3.1) | 250.9 (135.4 to 429.7) |
| Grenada | 63.1 (17.4 to 104.4) | 477.2 (131.8 to 790.3) | 2.2 (1.9 to 2.5) | 240.1 (128.5 to 393.0) |
| Guam | 39.9 (13.6 to 65.1) | 351.4 (123.2 to 567.2) | 0.4 (0.2 to 0.5) | 125.5 (47.9 to 237.0) |
| Guatemala | 61.5 (17.0 to 101.7) | 505.2 (140.3 to 844.9) | 2.2 (1.9 to 2.6) | 284.5 (156.4 to 453.6) |
| Guinea | 52.7 (11.5 to 103.7) | 374.1 (81.7 to 702.2) | 4.5 (3.0 to 6.1) | 335.4 (201.5 to 527.5) |
| Guinea-Bissau | 54.3 (10.4 to 100.7) | 385.9 (71.8 to 712.7) | 5.5 (3.8 to 7.2) | 392.5 (240.0 to 587.9) |
| Guyana | 63.8 (18.5 to 104.9) | 502.4 (138.6 to 804.2) | 3.6 (2.7 to 4.6) | 340.8 (197.2 to 506.7) |
| Haiti | 47.1 (11.6 to 85.2) | 324.3 (80.6 to 567.8) | 4.2 (3.1 to 5.8) | 323.5 (202.6 to 482.0) |
| Honduras | 56.6 (12.4 to 97.7) | 437.9 (96.9 to 757.0) | 3.0 (2.1 to 4.0) | 292.5 (164.0 to 456.7) |
| Hungary | 41.1 (12.8 to 68.0) | 375.0 (118.2 to 588.8) | 1.3 (1.0 to 1.6) | 148.5 (74.7 to 266.4) |
| Iceland | 51.4 (14.7 to 80.3) | 308.1 (88.0 to 484.8) | 1.0 (0.9 to 1.1) | 106.2 (53.8 to 205.1) |
| India | 33.2 (23.6 to 43.2) | 256.2 (185.6 to 325.7) | 2.5 (1.8 to 2.8) | 191.6 (146.9 to 237.7) |
| Indonesia | 34.9 (22.9 to 46.8) | 219.0 (147.8 to 294.1) | 0.4 (0.1 to 0.6) | 92.7 (54.1 to 136.0) |
| Iran | 50.0 (33.8 to 65.7) | 305.4 (211.4 to 392.6) | 0.7 (0.7 to 1.0) | 121.5 (81.7 to 172.7) |
| Iraq | 44.2 (13.3 to 73.4) | 278.9 (83.4 to 449.8) | 0.9 (0.7 to 1.1) | 124.7 (62.6 to 208.7) |
| Ireland | 63.8 (19.4 to 100.7) | 382.9 (113.0 to 589.7) | 1.3 (1.1 to 1.4) | 129.3 (66.8 to 242.3) |

| | | | | |
|---|---|---|---|---|
| Israel | 52.9 (17.4 to 83.2) | 307.6 (105.5 to 466.1) | 1.1 (1.0 to 1.2) | 113.4 (57.5 to 216.4) |
| Italy | 43.1 (28.7 to 59.7) | 253.4 (174.1 to 344.3) | 1.2 (1.0 to 1.4) | 97.1 (65.6 to 148.3) |
| Jamaica | 58.5 (17.0 to 95.4) | 426.3 (124.7 to 693.1) | 2.1 (1.5 to 2.7) | 211.9 (109.3 to 348.6) |
| Japan | 44.0 (28.3 to 61.0) | 261.6 (169.0 to 352.3) | 0.7 (0.6 to 0.7) | 87.0 (56.7 to 135.8) |
| Jordan | 43.5 (12.3 to 70.0) | 268.8 (74.0 to 436.0) | 0.7 (0.6 to 0.9) | 109.2 (53.4 to 193.0) |
| Kazakhstan | 49.8 (14.1 to 82.0) | 475.8 (136.7 to 769.9) | 1.9 (1.7 to 2.2) | 230.3 (126.4 to 386.6) |
| Kenya | 66.2 (48.5 to 87.0) | 411.9 (308.0 to 527.6) | 6.1 (4.7 to 7.5) | 377.9 (295.6 to 465.8) |
| Kiribati | 35.6 (8.8 to 63.9) | 317.8 (79.1 to 551.9) | 2.3 (1.7 to 3.1) | 253.8 (151.0 to 385.7) |
| Kuwait | 53.9 (14.7 to 86.6) | 358.1 (95.0 to 569.3) | 0.6 (0.5 to 0.7) | 117.9 (46.4 to 231.6) |
| Kyrgyzstan | 45.0 (12.2 to 76.1) | 416.9 (112.5 to 660.2) | 3.1 (2.5 to 3.7) | 284.3 (179.2 to 428.9) |
| Laos | 33.4 (8.5 to 56.8) | 252.3 (60.5 to 427.9) | 0.6 (0.4 to 1.3) | 124.1 (48.5 to 221.7) |
| Latvia | 40.9 (12.4 to 65.2) | 362.8 (117.0 to 571.2) | 1.5 (1.1 to 1.9) | 161.8 (86.2 to 286.3) |
| Lebanon | 45.6 (14.3 to 76.6) | 289.8 (93.5 to 476.6) | 1.7 (1.4 to 2.0) | 149.2 (83.7 to 244.1) |
| Lesotho | 49.1 (10.3 to 86.5) | 360.1 (79.7 to 602.7) | 3.2 (2.2 to 4.2) | 290.4 (165.7 to 427.6) |
| Liberia | 52.0 (10.5 to 97.4) | 365.3 (72.6 to 657.7) | 3.8 (2.3 to 5.5) | 290.5 (165.4 to 431.6) |
| Libya | 43.5 (12.8 to 69.9) | 266.7 (80.2 to 428.6) | 1.1 (0.7 to 1.5) | 131.7 (66.9 to 218.5) |
| Lithuania | 42.8 (13.8 to 67.3) | 389.2 (123.9 to 586.2) | 1.9 (1.5 to 2.3) | 190.1 (110.8 to 303.0) |

| | | | | |
|---|---|---|---|---|
| Luxembourg | 70.2 (23.9 to 109.7) | 439.9 (139.5 to 663.0) | 1.6 (1.4 to 1.9) | 153.5 (78.4 to 278.8) |
| Madagascar | 52.1 (13.4 to 96.6) | 337.3 (88.4 to 620.0) | 5.7 (4.4 to 7.7) | 363.1 (241.6 to 514.9) |
| Malawi | 52.1 (12.3 to 94.2) | 338.2 (75.6 to 610.6) | 8.5 (6.4 to 10.7) | 468.8 (329.8 to 652.8) |
| Malaysia | 44.6 (11.8 to 72.7) | 353.4 (94.8 to 559.1) | 0.7 (0.6 to 0.9) | 138.7 (54.2 to 247.8) |
| Maldives | 43.6 (13.0 to 75.8) | 376.5 (120.1 to 631.7) | 1.0 (0.8 to 1.4) | 154.0 (67.0 to 275.2) |
| Mali | 43.6 (10.2 to 84.1) | 288.3 (64.0 to 547.2) | 5.0 (3.3 to 6.9) | 321.2 (194.4 to 462.6) |
| Malta | 51.4 (15.2 to 84.2) | 290.5 (82.7 to 458.4) | 0.8 (0.7 to 0.9) | 96.5 (46.9 to 187.8) |
| Marshall Islands | 32.8 (8.3 to 60.2) | 278.6 (67.4 to 492.3) | 1.5 (1.2 to 2.0) | 186.3 (97.2 to 290.2) |
| Mauritania | 55.8 (15.7 to 95.1) | 384.2 (106.1 to 649.1) | 3.3 (2.3 to 4.3) | 269.7 (159.3 to 421.7) |
| Mauritius | 61.4 (21.2 to 99.5) | 630.3 (207.5 to 981.0) | 3.5 (3.1 to 3.8) | 339.8 (202.7 to 542.7) |
| Mexico | 77.0 (52.8 to 101.6) | 582.8 (400.6 to 753.8) | 2.0 (1.7 to 2.3) | 268.5 (192.0 to 360.9) |
| Micronesia (Federated States of) | 34.1 (7.2 to 58.6) | 289.1 (62.6 to 488.8) | 1.5 (1.1 to 1.9) | 184.8 (96.1 to 298.9) |
| Monaco | 60.0 (18.9 to 96.1) | 356.4 (121.8 to 564.0) | 0.6 (0.4 to 0.8) | 103.8 (42.2 to 209.9) |
| Mongolia | 43.2 (9.2 to 74.0) | 403.0 (82.2 to 672.8) | 1.8 (1.4 to 2.3) | 223.4 (116.4 to 364.4) |
| Montenegro | 38.2 (10.4 to 62.6) | 333.3 (89.4 to 532.7) | 0.7 (0.6 to 0.9) | 113.9 (46.1 to 221.3) |
| Morocco | 43.2 (10.5 to 74.4) | 281.1 (69.5 to 485.0) | 1.8 (1.3 to 2.2) | 168.5 (97.3 to 273.8) |
| Mozambique | 57.0 (11.1 to 104.6) | 409.6 (77.4 to 763.8) | 7.9 (5.9 to 10.6) | 455.7 (293.1 to 651.5) |

| | | | | |
|---|---|---|---|---|
| Myanmar | 37.5 (10.5 to 66.7) | 292.2 (84.4 to 518.6) | 1.0 (0.6 to 1.3) | 150.7 (65.5 to 260.5) |
| Namibia | 53.4 (15.7 to 92.2) | 405.5 (123.2 to 688.0) | 2.9 (2.1 to 3.9) | 274.7 (159.1 to 429.9) |
| Nauru | 39.4 (10.4 to 65.5) | 376.9 (99.3 to 611.3) | 1.7 (1.3 to 2.2) | 217.3 (111.1 to 348.7) |
| Nepal | 35.8 (7.8 to 63.6) | 339.5 (68.8 to 609.1) | 5.5 (3.1 to 7.6) | 290.5 (185.4 to 435.2) |
| Netherlands | 56.4 (16.1 to 91.4) | 347.3 (99.4 to 537.7) | 1.4 (1.2 to 1.5) | 126.6 (70.1 to 231.5) |
| New Zealand | 51.5 (20.6 to 77.9) | 312.9 (122.1 to 471.4) | 1.0 (1.0 to 1.1) | 121.5 (70.5 to 199.0) |
| Nicaragua | 51.3 (12.6 to 95.6) | 364.8 (85.9 to 645.8) | 1.3 (1.1 to 1.6) | 170.5 (85.6 to 302.2) |
| Niger | 41.0 (7.1 to 85.1) | 264.6 (48.4 to 540.1) | 4.8 (2.8 to 7.6) | 305.8 (174.6 to 484.5) |
| Nigeria | 66.4 (47.6 to 86.1) | 443.4 (326.7 to 580.9) | 3.2 (1.9 to 4.3) | 288.5 (205.5 to 374.5) |
| Niue | 39.8 (11.9 to 64.7) | 347.9 (108.6 to 551.5) | 1.4 (1.1 to 1.7) | 198.0 (120.5 to 310.1) |
| North Macedonia | 38.7 (11.3 to 62.8) | 341.9 (96.4 to 539.1) | 1.6 (1.1 to 2.1) | 158.7 (89.2 to 267.0) |
| Northern Mariana Islands | 38.2 (9.0 to 62.7) | 326.8 (80.8 to 516.1) | 0.6 (0.4 to 0.7) | 129.3 (53.0 to 234.5) |
| Norway | 67.4 (40.5 to 93.2) | 413.3 (247.4 to 567.6) | 1.4 (1.3 to 1.4) | 142.9 (91.5 to 227.9) |
| Oman | 49.9 (14.8 to 79.5) | 321.8 (89.0 to 497.8) | 0.4 (0.3 to 0.5) | 106.0 (39.3 to 202.5) |
| Pakistan | 36.1 (18.0 to 54.0) | 308.5 (157.1 to 447.3) | 4.4 (3.1 to 5.8) | 298.4 (227.7 to 395.6) |
| Palau | 43.0 (11.8 to 69.7) | 403.1 (115.7 to 631.4) | 1.9 (1.5 to 2.5) | 237.9 (134.2 to 361.8) |
| Palestine | 44.2 (13.9 to 77.6) | 279.0 (87.4 to 468.9) | 1.7 (1.5 to 2.2) | 161.7 (95.1 to 252.5) |

| | | | | |
|---|---|---|---|---|
| Panama | 70.8 (19.9 to 114.0) | 571.5 (170.7 to 897.7) | 1.5 (1.2 to 1.7) | 228.3 (105.9 to 403.1) |
| Papua New Guinea | 27.9 (6.6 to 50.8) | 227.3 (54.7 to 402.6) | 1.8 (1.2 to 2.7) | 183.7 (98.5 to 286.2) |
| Paraguay | 52.4 (17.3 to 86.1) | 399.9 (124.0 to 668.6) | 1.5 (1.1 to 2.0) | 192.5 (99.5 to 319.1) |
| Peru | 67.8 (18.9 to 113.0) | 534.4 (155.1 to 856.1) | 0.8 (0.6 to 1.1) | 189.3 (75.8 to 353.7) |
| Philippines | 36.4 (25.5 to 48.6) | 243.5 (175.2 to 319.5) | 0.4 (0.3 to 0.5) | 103.3 (70.5 to 144.9) |
| Poland | 39.1 (26.1 to 53.7) | 350.7 (236.8 to 458.2) | 2.2 (1.7 to 2.5) | 174.3 (126.5 to 233.6) |
| Portugal | 47.7 (12.7 to 76.4) | 269.2 (73.8 to 420.7) | 1.6 (1.4 to 1.8) | 119.2 (71.0 to 197.1) |
| Puerto Rico | 63.0 (18.4 to 100.1) | 460.9 (129.5 to 727.0) | 1.2 (1.0 to 1.4) | 164.6 (74.3 to 304.3) |
| Qatar | 55.4 (17.9 to 87.5) | 381.3 (122.2 to 587.3) | 0.6 (0.5 to 0.8) | 119.1 (48.4 to 232.5) |
| South Korea | 47.7 (12.1 to 74.3) | 315.4 (84.6 to 484.7) | 0.8 (0.7 to 1.0) | 108.7 (51.9 to 201.4) |
| Republic of Moldova | 32.2 (9.4 to 54.4) | 247.5 (76.3 to 407.6) | 1.7 (1.3 to 2.0) | 149.3 (93.5 to 228.1) |
| Romania | 42.1 (12.6 to 68.1) | 398.1 (121.7 to 617.6) | 1.7 (1.3 to 2.0) | 180.1 (100.7 to 290.7) |
| Russia | 32.0 (21.6 to 43.9) | 211.2 (143.6 to 281.8) | 0.5 (0.4 to 0.5) | 72.8 (48.2 to 110.6) |
| Rwanda | 54.7 (11.9 to 98.7) | 360.5 (77.4 to 634.1) | 6.8 (5.1 to 9.7) | 405.8 (276.0 to 585.1) |
| Saint Kitts and Nevis | 71.6 (22.5 to 116.3) | 564.3 (166.3 to 909.6) | 2.3 (1.9 to 2.8) | 269.3 (142.6 to 448.3) |
| Saint Lucia | 65.7 (17.1 to 111.3) | 519.3 (139.4 to 852.3) | 2.8 (2.3 to 3.3) | 279.8 (158.3 to 458.5) |
| Saint Vincent and the Grenadines | 63.9 (15.7 to 104.4) | 494.2 (132.8 to 812.4) | 3.2 (2.7 to 3.6) | 301.5 (179.4 to 451.7) |

| | | | | |
|---|---|---|---|---|
| Samoa | 35.2 (11.0 to 59.4) | 295.3 (90.6 to 476.0) | 1.2 (0.9 to 1.6) | 163.7 (85.1 to 268.3) |
| San Marino | 50.9 (14.5 to 78.5) | 284.9 (78.7 to 448.8) | 0.1 (0.1 to 0.2) | 67.0 (20.3 to 146.5) |
| São Tomé and Príncipe | 61.3 (17.8 to 104.1) | 439.0 (133.7 to 723.0) | 3.4 (2.4 to 4.5) | 292.2 (152.3 to 457.6) |
| Saudi Arabia | 67.1 (17.6 to 107.4) | 518.0 (145.1 to 805.9) | 1.5 (1.1 to 1.9) | 211.7 (99.0 to 386.4) |
| Senegal | 56.3 (12.0 to 104.7) | 400.8 (89.7 to 717.9) | 4.6 (3.2 to 6.2) | 347.6 (211.6 to 520.4) |
| Serbia | 45.0 (12.5 to 73.0) | 371.2 (109.1 to 567.6) | 1.4 (1.1 to 1.7) | 153.2 (81.0 to 270.2) |
| Seychelles | 42.0 (12.3 to 72.0) | 331.8 (94.3 to 560.3) | 1.0 (0.8 to 1.2) | 146.4 (65.4 to 254.5) |
| Sierra Leone | 49.8 (10.0 to 92.5) | 346.4 (73.4 to 636.3) | 4.0 (2.5 to 5.7) | 304.2 (166.8 to 454.2) |
| Singapore | 43.0 (12.2 to 69.1) | 271.3 (78.8 to 431.8) | 0.5 (0.4 to 0.5) | 82.0 (34.9 to 166.2) |
| Slovakia | 46.0 (13.5 to 76.6) | 458.3 (136.9 to 727.4) | 1.9 (1.5 to 2.4) | 199.8 (110.2 to 338.9) |
| Slovenia | 41.9 (13.0 to 68.1) | 376.7 (120.6 to 590.9) | 1.4 (1.0 to 1.8) | 140.9 (74.9 to 247.0) |
| Solomon Islands | 30.4 (7.0 to 55.0) | 256.7 (61.9 to 458.6) | 1.8 (1.2 to 2.6) | 193.8 (108.0 to 306.8) |
| Somalia | 35.5 (5.5 to 76.2) | 211.5 (34.2 to 459.8) | 10.3 (6.7 to 16.9) | 505.5 (305.5 to 770.1) |
| South Africa | 58.7 (35.8 to 81.5) | 407.3 (266.3 to 544.6) | 2.1 (1.8 to 2.4) | 231.0 (168.2 to 311.5) |
| South Sudan | 50.6 (11.3 to 97.9) | 334.8 (76.4 to 623.4) | 9.0 (6.5 to 12.5) | 490.1 (339.6 to 688.2) |
| Spain | 44.0 (13.7 to 71.8) | 269.3 (85.1 to 429.5) | 1.0 (0.8 to 1.1) | 92.3 (47.3 to 176.5) |
| Sri Lanka | 52.8 (12.8 to 86.9) | 476.5 (117.2 to 777.7) | 1.5 (1.0 to 2.1) | 197.1 (87.7 to 345.7) |

| | | | | |
|---|---|---|---|---|
| Sudan | 39.1 (10.3 to 67.1) | 242.3 (64.5 to 411.8) | 1.6 (1.1 to 2.1) | 167.7 (101.5 to 256.7) |
| Suriname | 66.6 (21.6 to 107.7) | 542.9 (184.4 to 868.1) | 2.6 (2.0 to 3.3) | 301.8 (175.6 to 474.2) |
| Sweden | 39.9 (16.9 to 62.9) | 245.2 (105.3 to 363.5) | 0.8 (0.7 to 0.9) | 87.4 (49.7 to 149.7) |
| Switzerland | 54.1 (14.9 to 85.3) | 314.1 (92.6 to 495.1) | 1.2 (1.0 to 1.3) | 109.8 (57.3 to 204.0) |
| Syria | 38.0 (10.6 to 62.4) | 229.3 (61.2 to 381.0) | 1.2 (0.8 to 1.6) | 127.8 (76.3 to 205.8) |
| Turkey | 57.0 (17.8 to 93.3) | 405.8 (127.1 to 642.6) | 1.8 (1.4 to 2.2) | 193.5 (111.2 to 316.2) |
| Taiwan (province of China) | 35.0 (9.3 to 55.4) | 334.2 (84.6 to 509.7) | 1.0 (0.9 to 1.1) | 126.7 (61.9 to 232.0) |
| Tajikistan | 45.0 (12.2 to 77.2) | 417.8 (113.0 to 698.8) | 4.6 (3.4 to 6.1) | 406.9 (269.1 to 572.0) |
| Thailand | 43.0 (13.9 to 71.2) | 344.2 (111.1 to 559.4) | 1.2 (0.9 to 1.5) | 152.3 (74.8 to 270.9) |
| Timor-Leste | 34.2 (9.7 to 60.3) | 251.7 (69.0 to 428.9) | 0.6 (0.4 to 1.3) | 121.1 (45.6 to 221.0) |
| Togo | 57.0 (12.8 to 105.2) | 399.6 (86.9 to 733.2) | 3.9 (2.5 to 5.4) | 312.9 (177.8 to 479.9) |
| Tokelau | 33.1 (7.7 to 59.4) | 268.2 (64.5 to 454.5) | 2.0 (1.7 to 2.6) | 216.8 (140.8 to 311.5) |
| Tonga | 32.9 (10.2 to 52.8) | 261.6 (79.9 to 422.3) | 1.0 (0.7 to 1.4) | 141.7 (75.7 to 229.2) |
| Trinidad and Tobago | 72.3 (20.5 to 119.0) | 593.4 (168.6 to 955.2) | 2.4 (1.8 to 3.0) | 287.4 (153.6 to 486.5) |
| Tunisia | 42.1 (12.1 to 71.3) | 257.9 (75.8 to 429.9) | 1.0 (0.7 to 1.4) | 118.1 (61.6 to 205.2) |
| Turkmenistan | 47.5 (11.3 to 81.9) | 459.8 (115.9 to 748.7) | 2.8 (1.9 to 3.9) | 305.2 (170.1 to 480.8) |
| Tuvalu | 33.2 (9.5 to 56.6) | 272.2 (74.7 to 438.5) | 1.3 (1.0 to 1.7) | 168.3 (91.6 to 261.9) |

| | | | | |
|---|---|---|---|---|
| Uganda | 64.1 (13.4 to 115.2) | 404.7 (85.8 to 722.6) | 5.7 (4.4 to 7.4) | 375.7 (247.1 to 555.6) |
| Ukraine | 36.5 (11.2 to 59.4) | 253.1 (77.6 to 403.7) | 0.9 (0.6 to 1.1) | 115.8 (62.0 to 194.0) |
| United Arab Emirates | 64.4 (22.0 to 105.6) | 482.4 (148.1 to 748.3) | 0.9 (0.6 to 1.1) | 177.4 (74.6 to 330.1) |
| UK | 72.0 (49.0 to 97.8) | 413.6 (276.6 to 554.0) | 1.2 (1.1 to 1.3) | 148.2 (101.8 to 221.1) |
| Tanzania | 60.7 (14.6 to 108.5) | 425.8 (103.0 to 749.0) | 7.0 (5.5 to 9.2) | 445.3 (294.8 to 641.6) |
| USA | 42.9 (27.1 to 60.7) | 341.6 (217.9 to 466.9) | 0.8 (0.7 to 0.8) | 111.2 (70.0 to 173.7) |
| Virgin Islands | 66.4 (20.2 to 107.6) | 500.4 (152.1 to 777.4) | 1.6 (1.1 to 2.2) | 231.8 (118.4 to 389.8) |
| Uruguay | 56.3 (13.4 to 91.5) | 394.4 (95.3 to 636.9) | 1.8 (1.6 to 1.9) | 183.7 (100.5 to 305.5) |
| Uzbekistan | 49.4 (13.6 to 79.9) | 479.2 (142.1 to 765.4) | 2.6 (2.2 to 3.1) | 297.9 (186.3 to 466.5) |
| Vanuatu | 30.8 (7.0 to 53.4) | 254.8 (56.8 to 433.2) | 1.6 (1.2 to 2.1) | 182.2 (96.2 to 284.9) |
| Venezuela | 64.6 (20.0 to 110.9) | 520.8 (149.0 to 889.5) | 2.2 (1.6 to 2.8) | 265.1 (138.8 to 462.2) |
| Viet Nam | 34.6 (8.0 to 58.9) | 246.7 (56.2 to 409.7) | 0.1 (0.0 to 0.3) | 79.1 (18.1 to 169.2) |
| Yemen | 33.3 (6.5 to 61.4) | 197.4 (36.9 to 352.4) | 1.5 (1.0 to 2.0) | 148.2 (79.9 to 235.6) |
| Zambia | 73.1 (18.7 to 129.0) | 547.3 (147.9 to 944.1) | 12.9 (9.5 to 17.1) | 746.4 (505.8 to 1,031.4) |
| Zimbabwe | 50.2 (11.8 to 91.6) | 380.2 (89.8 to 677.6) | 4.5 (3.0 to 6.3) | 346.5 (205.5 to 517.0) |

**Table 3. Age-standardised incidence, deaths, and DALYs of idiopathic epilepsy per 100,000 people (with 95% UI) in 2021 by sex and World Bank country income level**

| | Incidence | | | Death | | | DALYs | | |
|---|---|---|---|---|---|---|---|---|---|
| | Males | Females | Both sexes | Males | Females | Both sexes | Males | Females | Both sexes |
| **Global** | 45.1 (33.1 to 56.4) | 40.5 (29.4 to 51.0) | 42.8 (31.2 to 53.7) | 2.1 (1.8 to 2.4) | 1.4 (1.0 to 1.5) | 1.7 (1.5 to 1.9) | 201.3 (157.9 to 252.7) | 154.2 (114.7 to 201.8) | 177.8 (137.7 to 225.9) |
| **World Bank country income level** | | | | | | | | | |
| HICs | 55.5 (37.5 to 74.1) | 49.5 (33.1 to 67.3) | 52.5 (35.4 to 70.7) | 1.4 (1.3 to 1.4) | 1.0 (0.9 to 1.0) | 1.2 (1.1 to 1.2) | 142.5 (98.1 to 205.8) | 115.9 (77.5 to 173.1) | 129.2 (87.7 to 189.7) |
| UMICs | 42.0 (29.6 to 54.6) | 39.4 (27.5 to 51.1) | 40.7 (28.6 to 52.9) | 1.3 (1.1 to 1.5) | 0.8 (0.6 to 0.9) | 1.0 (0.9 to 1.1) | 152.5 (113.1 to 200.6) | 119.3 (82.8 to 163.9) | 136.1 (97.8 to 182.2) |
| LMICs | 42.2 (30.5 to 53.3) | 37.9 (27.5 to 48.4) | 40.1 (29.0 to 50.6) | 2.6 (2.0 to 3.0) | 1.9 (1.1 to 2.2) | 2.2 (1.7 to 2.5) | 218.5 (170.1 to 269.5) | 175.0 (129.5 to 224.0) | 196.9 (153.4 to 245.5) |
| LICs | 50.4 (34.0 to 68.0) | 42.8 (28.6 to 58.9) | 46.5 (31.1 to 63.5) | 7.0 (5.6 to 8.6) | 2.6 (1.9 to 3.5) | 4.6 (3.9 to 5.6) | 402.9 (321.9 to 501.7) | 230.9 (166.9 to 302.7) | 313.6 (248.0 to 392.3) |

## Figures

**Appendix Figure 1. Age-standardised prevalence of epilepsy from idiopathic and secondary epilepsy combined per 100,000 people (with 95% UI) by World Bank country income level and Socio-demographic Index (SDI) quintiles, both sexes, 1990–2021**

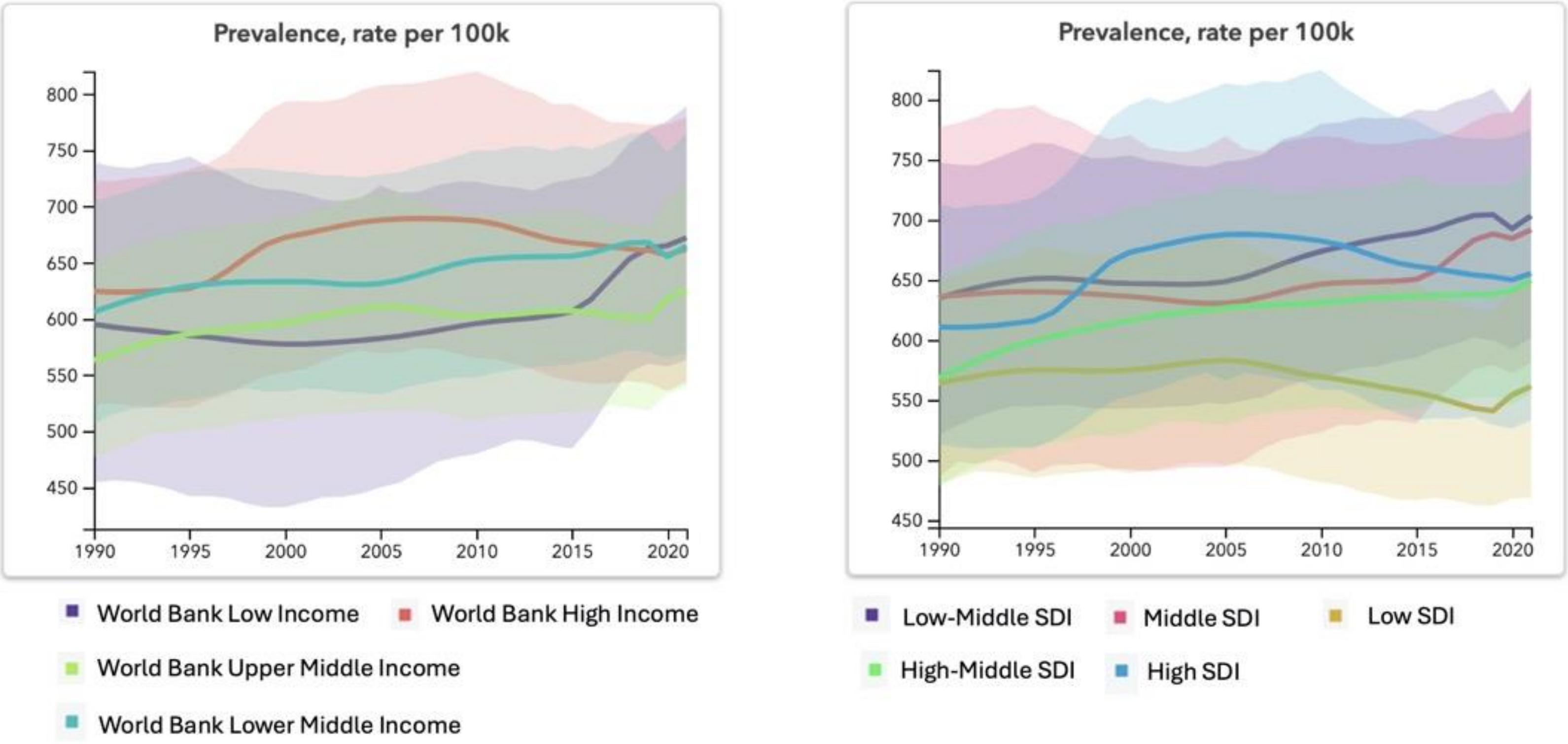

**Figure 2. Age-standardised incidence, prevalence, death, and DALY rates of idiopathic epilepsy per 100,000 people in the world by sex, 1990–2021**

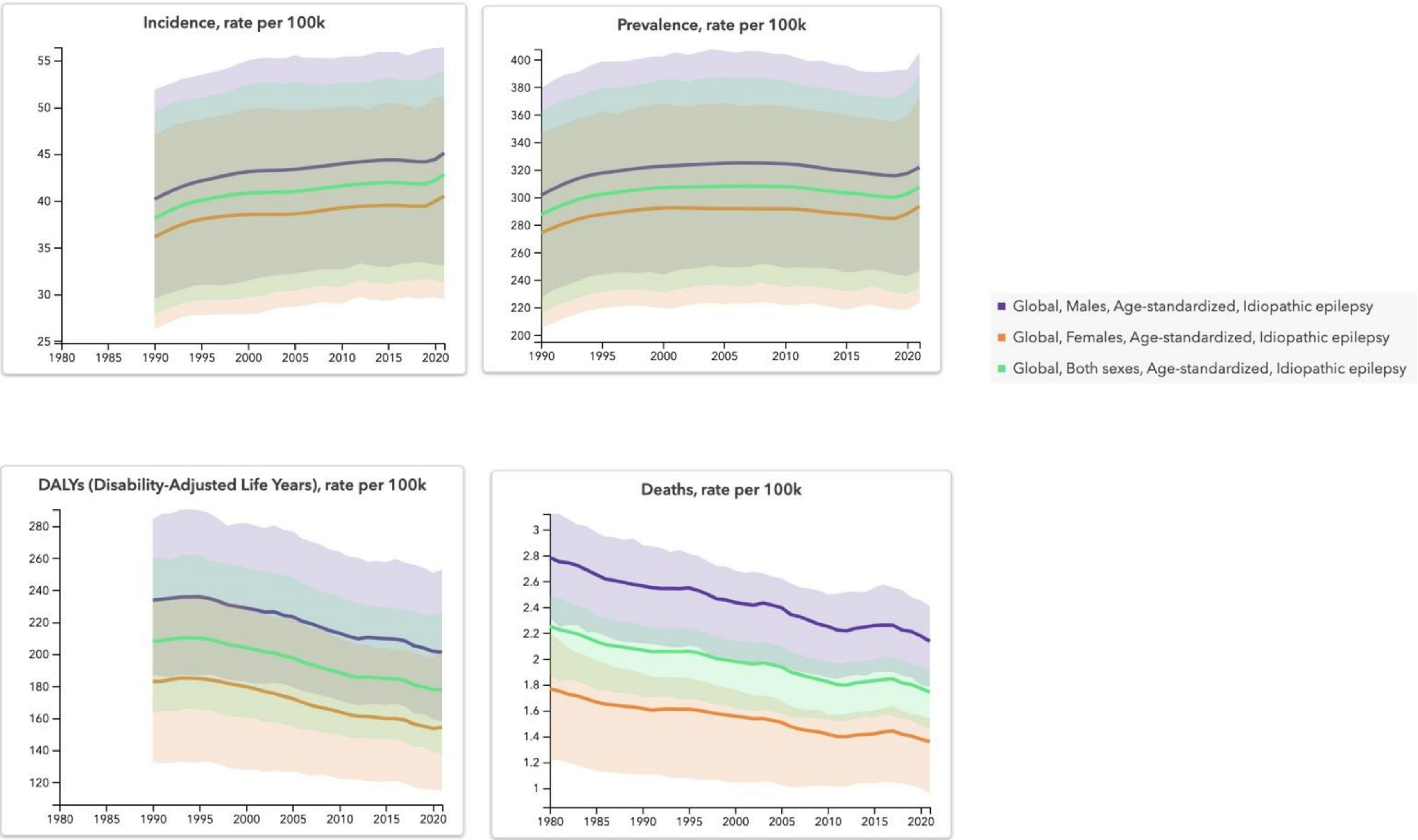

**Figure 3. Age-standardised idiopathic epilepsy incidence, death, and DALY rates per 100,000 people (with 95% UI) by World Bank country income level from 1990 to 2021, both sexes**

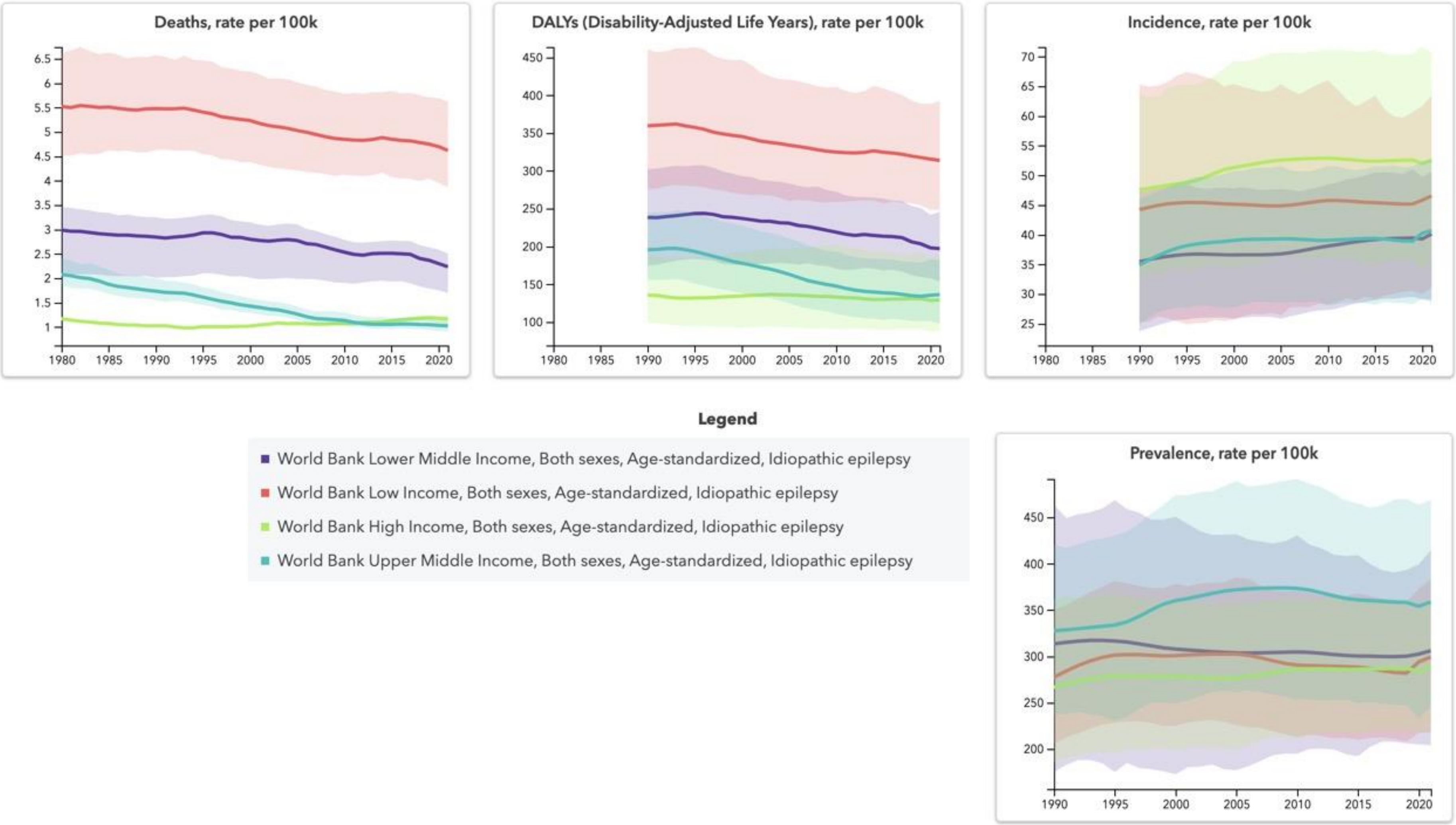

**Figure 4. Age-standardised idiopathic epilepsy incidence, death, and DALY rates per 100,000 people (with 95% UI) by Socio-demographic Index quintile from 1990 to 2021, both sexes**

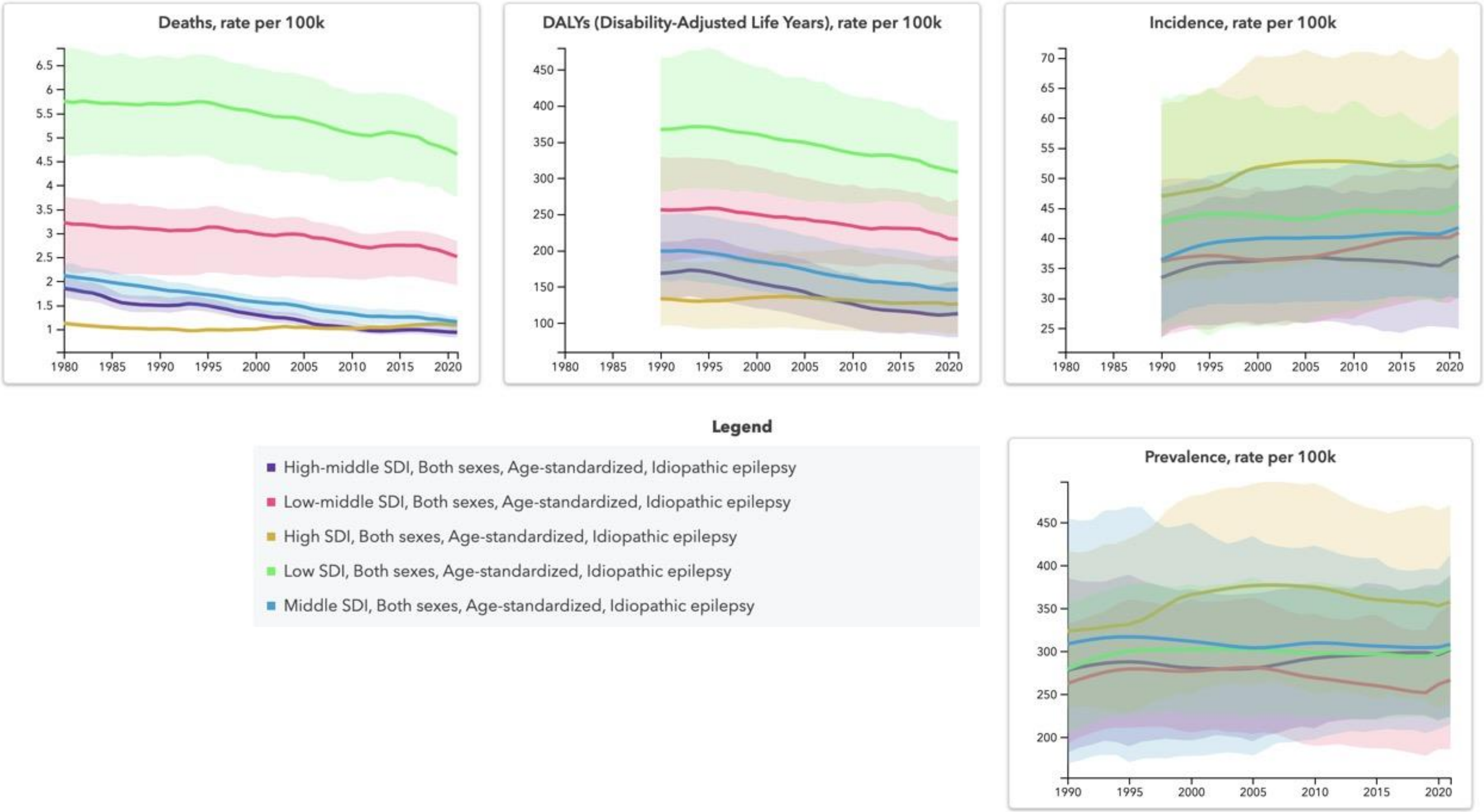

## References


1. Murray CJL, Aravkin AY, Zheng P, Abbafati C, Abbas KM, Abbasi-Kangevari M, . . . Lim SS. Global burden of 87 risk factors in 204 countries and territories, 1990-2019: a systematic analysis for the Global Burden of Disease Study 2019. *The Lancet*. 2020;396:1223-1249. doi: 10.1016/S0140-6736(20)30752-2
2. Steinmetz JD, Seeher KM, Schiess N, Nichols E, Cao B, Servili C, . . . Dua T. Global, regional, and national burden of disorders affecting the nervous system, 1990–2021: a systematic analysis for the Global Burden of Disease Study 2021. *The Lancet Neurology*. 2024;23:344-381. doi: https://doi.org/10.1016/S1474-4422(24)00038-3
3. Stanaway JD, Afshin A, Gakidou E, Lim SS, Abate D, Abate KH, . . . Collaborators GBDRF. Global, regional, and national comparative risk assessment of 84 behavioural, environmental and occupational, and metabolic risks or clusters of risks for 195 countries and territories, 1990-2017: A systematic analysis for the Global Burden of Disease Study 2017. *The Lancet*. 2018;392:1923-1994. doi: 10.1016/S0140-6736(18)32225-6
4. Roth GA, Abate D, Abate KH, Abay SM, Abbafati C, Abbasi N, . . . Murray CJL. Global, regional, and national age-sex-specific mortality for 282 causes of death in 195 countries and territories, 1980-2017: a systematic analysis for the Global Burden of Disease Study 2017. *The Lancet*. 2018;392:1736-1788. doi: 10.1016/S0140-6736(18)32203-7
5. United Nations Department of Economics and Social Affairs Population Division. World Population Prospects: The 2012 Revision. https://population.un.org/wpp/ Accessed 5 April 2024.
6. Collaborators GBDM. Global, regional, and national under-5 mortality, adult mortality, age-specific mortality, and life expectancy, 1970–2016: a systematic analysis for the Global Burden of Disease Study 2016. *Lancet (London, England)*. 2017;390:1084-1150. doi: 10.1016/S0140-6736(17)31833-0
7. Fisher RS, Acevedo C, Arzimanoglou A, Bogacz A, Cross JH, Elger CE, . . . Wiebe S. ILAE Official Report: A practical clinical definition of epilepsy. *Epilepsia*. 2014;55:475-482. doi: https://doi.org/10.1111/epi.12550
8. Guidelines for epidemiologic studies on epilepsy. Commission on Epidemiology and Prognosis, International League Against Epilepsy. *Epilepsia*. 1993;34:592-596. doi: 10.1111/j.1528-1157.1993.tb00433.x
9. Zheng P, Barber R, Sorensen RJD, Murray CJL, Aravkin AY. Trimmed Constrained Mixed Effects Models: Formulations and Algorithms. *Journal of Computational and Graphical Statistics*. 2021;30:544-556. doi: 10.1080/10618600.2020.1868303
10. Ferrari AJ, Santomauro DF, Aali A, Abate YH, Abbafati C, Abbastabar H, . . . Murray CJL. Global incidence, prevalence, years lived with disability (YLDs), disability-adjusted life-years (DALYs), and healthy life expectancy (HALE) for 371 diseases and injuries in 204 countries and territories and 811 subnational locations, 1990-2021: a systematic analysis for the Global Burden of Disease Study 2021. *The Lancet*. 2024;403:2133-2161. doi: 10.1016/S0140-6736(24)00757-8
11. Biolková V, Kolka Z, Biolek D. Algorithmic utilization of LDI transform for discrete-time filter design. Paper/Poster presented at: 2008 Proceedings of the Mosharaka International Conference on Communications, Signals and Coding, MIC-CSC 2008; 2008; https://www.scopus.com/inward/record.uri?eid=2-s2.0-70349812104&partnerID=40&md5=b356c608ef67cf4516e8bec4682ed951 [link].
12. Naghavi M, Ong KL, Aali A, Ababneh HS, Abate YH, Abbafati C, . . . Murray CJL. Global burden of 288 causes of death and life expectancy decomposition in 204 countries and territories

and 811 subnational locations, 1990-2021: a systematic analysis for the Global Burden of Disease Study 2021. *The Lancet*. 2024;403:2100-2132. doi: 10.1016/S0140-6736(24)00367-2
13. Global burden and strength of evidence for 88 risk factors in 204 countries and 811 subnational locations, 1990-2021: a systematic analysis for the Global Burden of Disease Study 2021. *Lancet*. 2024;403:2162-2203. doi: 10.1016/s0140-6736(24)00933-4
